# Ethnomedicinal Uses, Phytochemistry, Pharmacological Activities, and Toxicology of the Subfamily Gomphrenoideae (Amaranthaceae): A Comprehensive Review

**DOI:** 10.1002/cbdv.202500530

**Published:** 2025-05-24

**Authors:** Dayanna Isabel Araque Gelves, Giulia Cristina de Andreoli Souza, Alvaro Jose Hernandez Tasco, Marcos José Salvador

**Affiliations:** ^1^ Department of Plant Biology University of Campinas (UNICAMP) São Paulo Brazil; ^2^ Department of Industrial Microbiology Universidad de Santander (UDES) Bucaramanga Colombia

**Keywords:** bioactivity, gomphrenoideae, phytochemistry, toxicology, traditional use

## Abstract

The subfamily Gomphrenoideae is composed of about 480 accepted species, many of which have been historically used as medicinal plants, reason why they have been studied in terms of chemical profile, biological activity, and safety. This review consolidates the advances in research on this subfamily over the past 47 years, emphasizing its promising biotechnological potential and justifying the development of research in species that remain unstudied; additionally, it presents new perspectives based on the current knowledge, including the study of in vitro cultures and co‐cultures of the members of this subfamily as a sustainable approach to standardizing their chemical profiles and, consequently, enhancing their biotechnological potential. The information was collected from scientific databases such as Wiley Online Library, PubMed, Springer Link, Scielo, and Nature Research for 4 years. Verification of the scientific names and affiliations of the plants was carried out using the databases Global Biodiversity Information Facility (www.gbif.org), Plants of the World Online (www.plantsoftheworldonline.org), and The Plant List (www.theplantlist.org). To date, 512 chemical compounds have been reported for this subfamily, evidencing a wide diversity of chemical structures. It was also shown that the extracts, fractions, isolated pure compounds, and nanoparticles of this subfamily present antimicrobial, antioxidant, anticancer, anti‐inflammatory, antidiabetic, and antihyperglycemic activity, among others. Likewise, it is evident that the members of this subfamily do not present toxicity.

## Graphical abstract

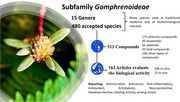

**Abbreviations**
%*A*
: percentage of cell apoptosis
%*I*
: percentage of inhibition
%*L*
: percentage of lethality
%*R*
: percentage of reduction
%*S*
: percentage of scavenging effect
%St: percentage of stimulation
%*V*
: percentage of viability
%ILS: percentage increase in life span
%TAC: total antioxidant activity
[]: concentration
=: same
↑: increase or higher or superior
↓: decrease or reduction or reduced
←: regression or reverse
1K1C1: kidney 1 clip
2K1C2: kidneys 1 clip
AAE: ascorbic acid equivalents
ABTS: 2,2‐azino‐bis(3‐ethylbenzothiazoline‐6‐sulfonic acid)
AcE: acetone extract
ACE: angiotensin‐converting enzyme
ACh: acetylcholine
AChE: acetylcholinesterase
AF: aqueous fraction
AgNPs: silver nanoparticles
ALP: alkaline phosphatase
ALT: alanine aminotransferase
AO/BE: acridine orange and ethidium bromide
AO/PI: acridine orange and propidium iodide
aPTT: activated partial thromboplastin
AqE: aqueous extract
AST: aspartate aminotransferase
AuNPs: gold nanoparticles
B‐16: melanoma cells
B16‐F10: murine melanoma
Bax Bcl‐2: associated X protein
BCG: bacillus Calmette–Guérin
BChE: butyrylcholinesterase
Bcl‐2: B‐cell lymphoma
BE: butanolic extract
BHT: butylated hydroxytoluene
BrdU: 5‐bromo‐2′‐deoxyuridine
BSA: bovine serum albumin
BT: total bilirubin
BthTX‐I: bothropstoxin I
BthTX‐II: bothropstoxin II
BuF: butanolic fraction
BUN: blood urea nitrogen
BW: body weight
Ca: calcium
CAA: cellular antioxidant activity
CAM: chloramphenicol
CAT: catalase
CB: conjugated bilirubin
CC_50_
: 50% cytotoxic concentration
CCB: calcium channel blocking
CCh: carbachol
Cdc2: cell division cycle protein 2 homolog
CF: chloroform fraction
CFA: Freund's complete adjuvant
CK‐MB: creatine kinase
ClE: chloroform extract
CM: chorioallantoic membrane
COX‐1: cycloxygenase‐1
COX‐2: cycloxygenase‐2
CREB: cAMP response element‐binding protein
CRP: C reactive protein
Cur: curcumin
D: time of death of worms
DAPI: 40,60‐diamidino‐2‐phenylindole
DBP: diastolic blood pressure
DCFH‐DA: 6‐carboxy‐2′,7′‐dichlorodihydrofluorescein diacetate
DCM: dichloromethane
DF: dichloromethane fraction
DOX: hydroxydaunomycin hydrochloride (doxorubicin)
DPPH: 1, 1 diphenyl‐2‐picrylhydrazyl
EAC: Ehrlich ascites carcinoma
EaE: ethyl acetate extract
EB: extract of betacyanins
EC50: values of the antiglucosidase activity: Sample concentration required to achieve 50% antiglucosidase activity
EC50: values of the anti‐inflammatory activity: Sample concentration providing 50% of inhibition in the production of NO
EC50: values of the antioxidant activity: Sample concentration providing 50% of the antioxidant activity or 0.5 of absorbance in the reducing power
ED_50_
: 50% inhibition of growth
EE: ethanolic extracts
Ehr Ca: ehrlich's carcinoma
ELISA: enzyme‐linked immunosorbent assay
EqE100: concentrations of the extract that stimulated the cell proliferation equivalent 100 pM 17β‐estradiol
ESR: erythrocyte sedimentation rate
FD: fraction of DCM
FEA: fraction of ethyl acetate
FEaMc: fraction of ethyl acetate: methylene chloride
FEaMcM: fraction of ethyl acetate: methylene chloride: methanol
FEtAc: fraction of ethanol: acetone
FF: flavonoid fraction
FH: fraction of hexane
FHa: fraction of hydroalcohol
FMW: fraction of methanol: water
FnB: fraction of *n*‐butanol
FnH: fraction of hexane
FST: forced swimming test
FTC: ferric thiocyanate
G1: phase cell growth
GC: glucocorticoids
GI50: value of hepatoprotective activity: sample concentration providing 50% of inhibition of the net cell growth
GLB: glibenclamide
HaE: hydroalcoholic extract
Hb: hemoglobin
HBV: hepatitis b virus
HCMV: human cytomegalovirus
HCT‐8: human colon carcinoma
HCTZ: hydrochlorothiazide
HdE: hydrophilic extracts
HDL‐C: high‐density lipoprotein cholesterol
HE: hydromethanolic extracts
HeE: hexanic extracts
HepG2: human hepatocellular carcinoma cells
HF: 20‐hydroxyecdysone‐enriched fraction
HG: high glucose
HL60: human leukemia cell
HSV‐1: herpes simplex virus Type 1
HSV‐2: herpes simplex virus Type 2
IC: inhibitory concentration
IDF: international diabetes federation
IL‐6: interleukin‐6
IMD: index of mucosal damage
J774: cancerous macrophage cell lines from BALB/C mouse
KB cell: tumor line was derived from a human epidermid carcinoma of the nasopharynx
Km: substrate concentration that yields a half‐maximal velocity
LA: LDH activity
LC50: lethal concentration 50
LC90: lethal concentration 90
LD50: lethal media dose
LDH: lactate deshydrogenase
MABP: mean arterial blood pressure
MAP: mean arterial pressure
MBC: minimal bactericidal concentration
MCF‐7: human breast cancer cell line
MCW: made with cold water
MDA: malondialdehyde
MDA‐MB‐435: melanoma
ME: methanolic extracts
MFC: minimal fungicidal concentrations
MHW: made with hot water
MI‐: microorganisms not irradiated with laser irradiation
MI+: microorganisms irradiated with laser irradiation
MIC: minimum inhibitory concentration
MO: microorganism
MPO: myeloperoxidase
MWMT: Morris water maze task
NA: not applicable
NAG: *N*‐acetylglucosaminidase
ND: not detected
Nd: not determined
NDNS: numerical data not shown
NE: no effect
*n*HE: *n*‐hexane extracts
NO: nitric oxide
NORT: Novel Object Recognition Test
NP‐SG: non‐protein sulfhydryl groups
NT: nitrotyrosine
OGTT: oral glucose tolerance test
OSTT: oral starch tolerance test
OVX: ovariectomized
P: times of paralysis
PARP: poly (ADP‐ribose) polymerase
PBMN: peripheral blood mononuclear cells
PC3: human prostate cancer cell line
PCNA: proliferating cell nuclear antigen
PE: phenylephrine
PEE: petroleum ether extracts
PEF: petroleum ether fraction
PFC: plaque forming cells
PGE2: prostaglandin E2
Phe: phenylephrine
PLP2: porcine liver primary cells
PMNLs: polymorphonuclear leukocytes
PT: preventive therapy
PTP1B: protein tyrosine phosphatase 1B
PTT: prothrombin time
PTZ: pentylenetetrazole
qRT‐PCR: Quantitative Reverse‐Transcription PCR
RBCs: red blood cells
ROS: reactive oxygen species
RTE: relative Trolox equivalent
RT‐PCR: real‐time quantitative polymerase chain reaction
RW: relative weight
S180: sarcoma 180
SBP: systolic blood pressure
SC50: scavenging concentration
SEM: scanning electron microscope
SF‐295: glioblastoma
SGOT: serum glutamic oxaloacetate transaminase
SGPT: serum glutamic pyruvate transaminase
SIs: selectivity indices
SK‐N‐SH: human neuroblastoma cell line
SNP: sodium nitroprusside
SOD: superoxide dismutase
SPF: sun protection factor
SRB: sulforhodamine B
SRC: spontaneous rhythmic contraction
STZ: streptozotocin
T: trace of inhibition
TAA: total antibacterial activity
TC: the cure
TE: mmol Trolox equivalents
TEAC: Trolox equivalent antioxidant capacity
TNBS: trinitrobenzenesulphonic acid
TNF‐α: tumor necrosis factor alpha
TP: total protein
TST: tail suspension test
UNa: urine sodium
*V*_max_
: maximum velocity
WBCs: white blood cells
WST‐1: water‐soluble tetrazolium salt
ZI: zone of inhibition
ZnONPs: zinc oxide nanoparticles
ΔΨm: mitochondrial membrane potential

## 1 Introduction

Amaranthaceae is an important family of plants and includes species of economic interest; many are marketed as ornamental plants or to be used as food or healthcare based on traditional medicinal knowledge. However, several species are also known as invasive or parasitic plants and are even listed among the worst weeds. Amaranthaceae is placed in the order Caryophyllales Juss. ex Bercht. & J. Presl. and comprises about 163–195 genera and approximately 2215–3805 species, according to The Plant List database (www.theplantlist.org), including those formerly treated as the family Chenopodiaceae [1, 2, 3, 4, 5, 6, 7, 8, 9].

The Amaranthaceae family has recently become the subject of intensive systematics research. Results of the molecular genetic studies suggest that the traditional classification based on morphological and anatomical characters often did not reflect phylogenetic relationships. The family Amaranthaceae (in their narrow circumscription) is classified into two subfamilies, Amaranthoideae and Gomphrenoideae, and contains about 65 genera and 900 species [1, 7].

**SCHEME 1:**
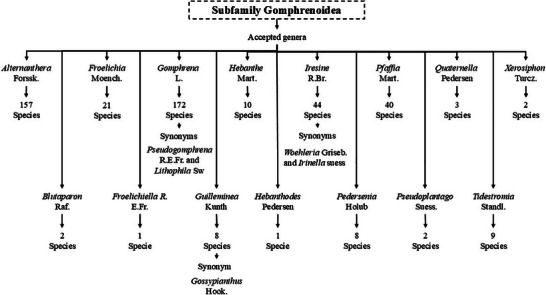
Members of the subfamily Gomphrenoideae. The subfamily Gomphrenoideae has 15 accepted genera and additionally has the genus *Pseudogomphrena* R.E.Fr. and *Lithophila* Sw. that are synonyms of the genus *Gomphrena* L.; *Woehleria* Griseb. and *Irinella* suess that are synonymous with *Iresine*; and *Gossypianthus* Hook. that is synonymous with *Guilleminea* Kunth. In terms of species, this subfamily has 480.

The subfamily Gomphrenoideae comprises about 480 accepted species distributed in 15 genera (Scheme 1) (GBIF), with the majority of its members being annual and perennial herbs, with some shrubs or small trees and climbing plants that have adapted to salty soils, arid environments, and human settlements [4, 6, 8].

Members of the Gomphrenoideae subfamily are widely used in traditional medicine in Asia, America, and Africa, making them a focus of interest for researchers. Scientists seek to verify their medicinal properties through studies of the chemical and pharmacological profile, with the goal of finding new chemical compounds that could lead to the development of new, more efficient, and safer drugs.

This article provides a comprehensive review that consolidates all available information on members of this subfamily. A wide range of topics are covered, including traditional uses, reported phytochemical profiles, biological activities of interest, and safety and/or toxicity of the extracts studied up until April 2024. Additionally, it concludes with a comparison of traditional uses and activities verified under laboratory conditions and perspectives and research directions.

This review aims to highlight the biotechnological potential of the members of this subfamily, proposing them as a promising source of bioactive molecules. It also emphasizes the importance of studying the relationship among traditional uses, chemical profiles, biological activities, and safety; additionally, it seeks to demonstrate the effect of biotic and abiotic factors on the chemical profile, among which it can mention location, climatic conditions, available nutrients, exposure to UV light, interaction with other living beings, and even the plant genotype; this highlights the need for new research strategies that allow for controlled growth conditions, enabling the optimization and standardization of metabolite production in plants. As a sustainable alternative, the use of in vitro plant tissue cultures is suggested.

## 2 Methodology

**SCHEME 2:**
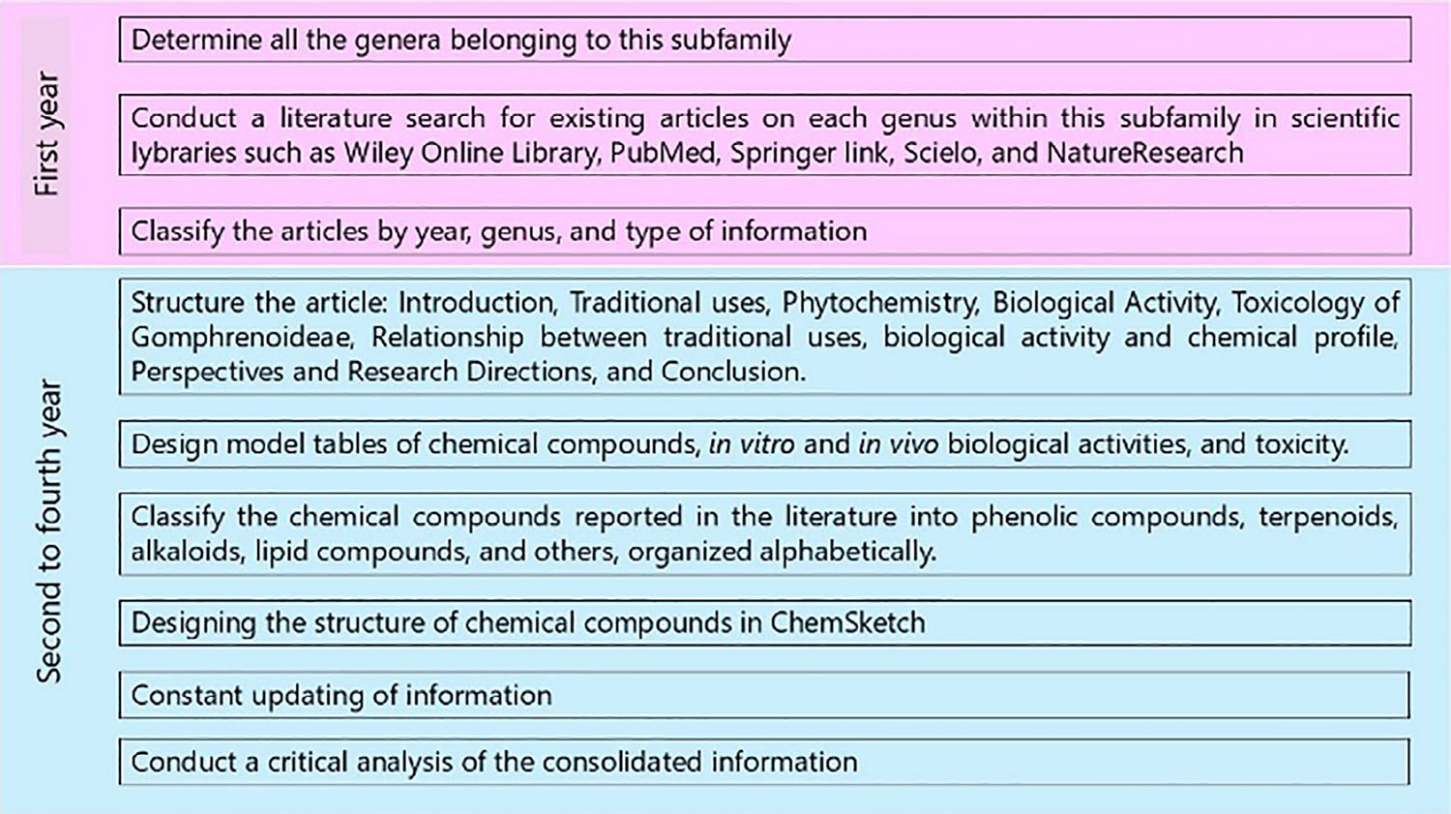
Methodology and work plan. The literature review was conducted in several stages, including a comprehensive bibliographic search, article structuring, table and chemical structure design, and concluding with a critical analysis.

To gather relevant literature, a comprehensive search was conducted using widely recognized scientific libraries. The search focused on keywords such as names of accepted genera or their synonyms, and the literature search was limited to sources in English. Chemical structures were drawn using ChemSketch, and their names, structures, and classifications were confirmed via the PubChem and ChemSpider websites. The information was summarized in different sections in the form of tables and figures for a better understanding. Scheme 2 outlines the methodology and work plan followed to develop this review. All plant names were consulted in “Global Biodiversity Information Facility” (www.gbif.org), “Plants of the World Online” (www.plantsoftheworldonline.org), “The Plant List” (www.theplantlist.org), “The World Flora Online” (http://www.worldfloraonline.org), MPNS (http://mpns.kew.org), on July 14, 2023 and May 20, 2024.

## 3 Traditional Medicinal Uses

Ethnomedicine plays a significant role in both research and society, with 80% of the population relying on traditional medicine for healthcare. The evidence of the pharmaceutical potential of commonly used plants has increased since 2013. By 2020, the WHO indicated that over 20 000 species of plants are utilized in medicine, with 13 000 plants having been studied. Furthermore, various sources indicate that 25%–50% of modern medicine is based on compounds derived from plants [10, 11, 12, 13, 14, 15].

Currently, many drug development studies are based on traditional medicine, among which can be cited aspirin, atropine, curare alkaloids, ephedrine, cortisone, digoxin, morphine, penicillin, and tubocurarine [11, 15, 16].

In Africa, America, Asia, Europe, and Antarctica, different members of this subfamily have been used to treat a wide variety of conditions, including chronic diseases, infectious diseases, skin diseases, respiratory issues, gastrointestinal disorders and sexually transmitted diseases, urinary disorders, malaria, diabetes, cancer, hypertension, burns, wounds, snake bites, and scorpion stings, among others. The main uses of some of the members of this subfamily are summarized in Table 1. It is important to note that some plants listed in Table 1 are included in Ayurveda, Unani, Siddha, Homeopathy, Chinese Pharmacopoeia, and “Zhonghua Bencao.”

**TABLE 1:** Uses in traditional medicine for major species of the Gomphrenoideae subfamily.

Accepted names	Vernacular names and/or synonyms	Geographical location	Medicinal part	Medicinal condition treated	References
*Alternanthera bettzickiana*	Baptist plant, border plant, joyweed, Matiti ya ba temoins de Jéhovah, nanthara, and red calico plant	Pakistan, South America, Thailand	Whole plant, leaves	Treatment of arthritis, gastrointestinal discomfort, menstrual pain, prevention of dementia, and its use as a mild laxative. Additionally, it is characterized by having anti‐Alzheimer's, anti‐inflammatory, antimicrobial, antioxidant, antipyretic, blood purifying, cytotoxic, diuretic, healing, hemolytic, and mutagenic properties. It is also used to promote lactation (as a galactagogue) and to provide nourishment	[17, 18, 19, 20, 21]
*Alternanthera brasiliana*	Brazilian joyweed, Carrapichinho, Doril, Novalgina, Lancetilla macho, Penicillin, Perpétua, Perpétua do mato, perpetuate of the bush, Tetracycline, Terramycin	Australia, Central America (e.g., Belize, Guatemala, Honduras, Nicaragua) French Guiana French Guiana, India, North America (Mexico, United States), South America (e.g., Argentina, Bolivia, Brazil, Colombia, Ecuador, Guyana, Peru, Suriname, Venezuela)	Leaves, whole plant	Treatment of asthma, bronchitis, cancer, cough, cold, diarrhea, discharge, fever, flu, headache, infections, inflammation, influenza, skin injuries, and wound healing. It is also used as an abortifacient, analgesic, antinociceptive, anticonvulsant, antitumoral, antiviral, anxiolytic, cholagogue, diuretic, galactagogue, and immunomodulator	[22, 23, 24, 25, 26, 27, 28, 29, 30, 31, 32]
*Alternanthera caracasana* HBK	Tianguis, tianguistumina, tianguispepetla, tlalpetate	Mexico	Stems, leaves, flowers, and roots	Treatment of dysentery, diarrhea, fever, and other conditions	[33]
*Alternanthera flavescens*	Lancetilla hembra			Treatment of fever and wound healing	[31]
*Alternanthera littoralis* P. Beauv.	*Alternanthera maritima* (Mart.) St. Hil.	Brazil		Treatment of infectious and inflammatory diseases	[8, 34]
*Alternanthera paronychioides*		Central and South America		Treatment of hyperuricemia, gout, rheumatic arthritis, nephritis, cystitis, uremia, diabetes, and systemic neuralgia	[35]
*Alternanthera philoxeroides*	Alligator weed, haicha shak, Phak Pet	Australia, Asia (e.g., Bangladesh, China, India), South America		Treatment of acute brain fever, anemia, diabetes, diarrhea, dysentery, encephalitis, hazy vision, herpes zoster, inflammation, influenza, malaria, measles, night blindness, pain, postnatal complaints, postnatal depression, puerperal fever, and viral infectious diseases. It is also used as an antipyretic, diuretic, and dressing for wounds and ulcers	[36, 37, 38, 39, 40, 41, 42]
*Alternanthera porrigens*	Sanguinaria, Moradilla, Lancetilla	Peru	Whole plant	Cleansing the womb after childbirth	[43]
*Alternanthera pungens*	Kakishak and Motsweetswe	Bangladesh, Limpopo Province	Tuber and whole plant	Treatment of mouth ulcers, cough, fever, gonorrhea (drop), kidney problems, and malaria	[16, 44, 45]
*Alternanthera repens*	Tianquis, tianquiz, or tianguispepetla	Mexico		Treatment of gastrointestinal ailments, such as diarrhea, inflammation, and stomach ache, as well as for the treatment of typhus fever. It is also used as diaphoretic, diuretic, and astringent agent	[46, 47]
*Alternanthera sessilis*	*Alternanthera triandra*, *Alternanthera repens*, Abisrana, amaranth, Angelica, Bhiringi jhar, Brede chevrette, bunga‐bunga, Carpet weed, Chanchi, Chanchi shak, Daun tolod, Dwarf copperleaf, Gandal, Gudrisag, Hong Tian Wu, Haicha, Honagone, Honugonesoppu, Hong Tian Wu, Horng‐tyan‐wu, Kachari, keremek, keremak merah, kermak putih, Lian zi cao, Lilonchi, Lupo, Matyakshika, Matikanduri, Matsyaksi, Minannani, Mukunuwenna, Phak ped khao, Phak pet daeng, Ponnagantikura, ponnankannikkirai, ponnandan, ponnanganni, ponnannani, pudoh, rumput aoh, red sessile joyweed, Sachi‐shak, serapat, Sessile Joy weed, water Dwarf Copperleaf	Africa, Argentina, Australia, Bangladesh, Bhutan, Brazil, Cameroon, Chad, China (e.g., Huanjiang), Ecuador, Egypt, Gambia, India, Indonesia, Iran, Kenya, Malaysia, Micronesia, Nepal, New Zealand, Nigeria, Pakistan, Philippines, Saudi Arabia, Singapore, Soloman Islands, Sri Lanka, Taiwan, Uganda, United States, Zambia, and Zaire	Whole plant, leaves, roots, and shoots	Treatment of anemia, aphthous ulcer, asthma, blood dysentery, bone fractures, bronchitis, burning sensations, chickenpox, cough, cuts, diabetes, diarrhea, dysentery, dyspepsia, eczema, eye diseases, fever, flatulence, gonorrhea, hemorrhoids, headache, helminthiasis, hepatitis, hernia, hypertension, indigestion, kidney diseases, leucorrhea, liver and spleen diseases, low sperm count, lung diseases, malaria, measles, menstrual disorder, nausea, neuralgia, night blindness, ophthalmia, post‐natal depression, pruritis, rheumatism, severe pain, skin diseases, splenomegaly, sprains, tight chest, ulcers, venereal disease, vertigo, vomiting, vomiting blood, and wound healing. It is also used as an abortifacient, analgesic, anti‐inflammatory, antioxidant, antidote to snakebite and scorpion sting, antimicrobial, for bleeding control, as a cholagogue, diuretic, galactagogue, and for refreshing of eyes and body. Additionally, it is used as a poultice for boils, to relieve neuritis, and to remove tiredness, laziness, and sleepiness	[38, 48, 49, 50, 51, 52, 53, 54, 55, 56, 57, 58, 59, 60, 61, 62, 63, 64, 65]
*Alternanthera tenella*	Anador, Enxuga, Joyweed, melhoral, Meracilina, pérpetua do mato, and quebra panela	Australia, India, and South America (e.g., Brazil)	Leaves and roots	Treatment of bronchitis, bruises, cough, diabetes, diarrhea, dysentery, fevers, flatulence, genital inflammation, headache, inflammation, infections, itches, nausea, pain, swelling, vomiting, and wounds. It is also used as a diuretic	[29, 66, 67, 68, 69, 70]
*Blutaparon portulacoides*	Capotiraguá, pirrixiu, or bredo‐de‐praia	Brazil		Treatment of leukorrhea and vulvovaginitis	[71]
*Froelichia*	Cottonweed, snake‐cotton, and roadside weed	From the southern extremes of Canada to Northern Argentina and Uruguay	NA	To date, no traditional uses have been reported	[72]
*Froelichia floridana* (Nuttall)	Florida snake‐cotton and plains snake‐cotton	North America, West Indies of the Caribbean, and Australia	NA	To date, no traditional uses have been reported	[72]
*Gomphrena*	Bachelor Button, Globe Amaranth	Americas (particularly in South America), Antarctica, and Indo‐Malaysia		Treatment of asthma, infant flu, body wounds, bronchial disorders, cooling, cough, diarrhea, fever, gastrointestinal and respiratory disorders, high cholesterol, infectious diseases, jaundice, kidney disorders, liver disease, malaria, oliguria, throat disorders, and urinary problems. It is also utilized as an analgesic, tonic, and carminative	[2, 3, 73]
*Gomphrena arborescens* L.	Paratudo, Paratudinho, Perpétua raiz do padre	Brazil	Leaves, flowers, and tuberous roots	Treatment of colitis, fevers, intermittent fevers, malaria, mental fatigue, and weakness. It is also utilized as an antidiarrheal, antithermal, antitoxic, aromatic, emmenagogue, eupeptic, protector, and tonic	[74]
*Gomphrena boliviana*		Argentina	Leaves and roots	Treatment of gastrointestinal disorders, infections, stomachache, and traumatic injuries	[75]
*Gomphrena celosioides*	*Gomphrena serrata*, *Gomphrena decumbens*, adukowé, amegantaxe, bachelor's button, brava, perdudilla, perpétua, pkaa Toum Hou, prostrate globe‐amaranth, soft khaki weed, and white‑eye	Americas (Argentina, Benin, Brazil, Paraguay, and Uruguay), Africa, Australia, Cambodia, East and West Vietnam, India, Indo‐Malaysia, Nigeria, Togo, and Zimbabwe	Leaves, roots, whole plant	Treatment of asthma, bronchitis, wound healing, coughs, cold, dermatological problems, diabetes, diarrhea, dysmenorrhea, fever, gastrointestinal diseases, hay fever, hypertension, kidney infections, jaundice, kidney stones, lithiasic problems, liver diseases (e.g., viral hepatitis A and C, liver damage), malaria, renal disorders, respiratory diseases, sexually transmitted diseases, skin infections/diseases/problems, infectious diseases, urinary tract disorders, vulvovaginitis, and worms. It is also used as an abortive, analgesic, antifungal, antibiotic, diuretic, immunostimulant, and tonic/carminative	[3, 76, 77, 78, 79, 80, 81, 82, 83, 84, 85, 86, 87, 88, 89, 90, 91, 92]
*Gomphrena globosa*	Bachelor button, Botamphul, Globe amaranth, Meilingper, Perpétua, Perpétuas‐roxas, Qianrihong, Trochiek Toun Say, and White bachelor button	Argentina, Bangladesh, Belize, Bolivia, Brazil, Cambodia, Canada, China (Huanjiang), Colombia, Costa Rica, Ecuador, French Guiana, Guatemala, Guyana, Honduras, India, Mexico, Panama, Peru, Portugal, South Africa, Suriname, United States, Trinidad, Tobago, Venezuela	Leaves, inflorescence, flowers, rhizome, and whole plant	Treatment of bronchial asthma, bronchitis, cough, diabetes, diarrhea, gallstones, gangrenous wounds, giddiness, hemorrhage, headache, heat and indigestion, hemoptysis, hoarseness, hypertension, indigestion, jaundice, kidney and prostate problems, oliguria, reproductive problems, respiratory diseases, urinary retention, tuberculosis, urinary system conditions, uterine infection, and whooping cough. It is also used as an antimicrobial, antioxidant, and expectorant	[3, 25, 29, 44, 79, 93, 94, 95, 96, 97, 98]
*Gomphrena macrocephala*		Brazil	Roots	It is used as a stimulant and a tonic	[99]
*Gomphrena martiana*	Solo and yerba de pollo	South America (e.g., Argentina)	Leaves and roots	Treatment of liver, kidney, urinary tract, and gastrointestinal disorders; infections; stomachache; and traumatic injuries. It is also used as a diuretic and blood purifier	[75, 100]
*Gomphrena virgata*	Cangussu‐branco	Brazil		Treatment of pain, inflammation, and infection. It is also used as an anti‐lethargic	[101]
*Guilleminea densa*	Sanguinaria			Treatment of gastric ulcers and menstrual cramps. It is also used as an antihemorrhagic	[102]
*Iresine angustifolia*	Hierba del arlomo	Mexico		Treatment of insect bites	[103]
*Iresine diffusa*	*Iresine celosia*, *Iresine celosioides*, herb of the Mayas, Paja Blanca, and Sangrinaria	Central and South America (e.g., Mexico, Peru), the West Indies, and the Southeastern United States	Whole plant	Treatment of anorexia, cancer, fever, inflammation, malaria, menstrual symptoms in adolescents, mouth sores, oral infections, prostate and urethra ailments, rash, skin problems, swelling, and typhoid fever	[43, 104, 105]
*Iresine herbstii* Hook	Bloodleaf, cimora senorita, chicken gizzard, beefsteak plant, herbst's bloodleaf, Mussurú, and Phak phaeo daeng	The entire world	Whole plant, aerial part, leaves, and stem	Treatment of anemia, broken bones, cancer, candidiasis, burns, eczema, wound healing, inflammatory bowel diseases, peptic ulcer, pimples, and sores. It is also used as antipyretic, skin depurative, and tonic	[106, 107, 58, 59, 108, 109, 110, 111]
*Pfaffia glomerata*	Acônito, Brazil ginseng, corango sempre‐viva, dipyrone, fáfia, paratudo, and novalgina	Brazil and Ecuador	Roots	Treatment of cancer, cholesterol, diabetes, flu, gastritis, impotence, inflammatory disorders, memory lapses, local pain, palpitations, rheumatism, stomach problems, and stress. It is also used as antioxidants, aphrodisiac, stimulant, tonic, and for wound healing. As well as it is utilized for restoring vital functions, increasing physical strength and mental equilibrium, and protecting the gastric mucosa from injury	[25, 112, 113, 114, 115, 116, 117, 118, 119]
*Pfaffia paniculata*	*Hebanthe eriantha*, *Hebanthe paniculata*, *Gomphrena paniculata*, *Gomphrena eriantha*, *Iresine erianthos*, *Iresine paniculata*, *Iresine tenuis*, *Pfaffia eriantha*, *Pfaffia virgata*, *Xeraea paniculata*, Brazilian ginseng, paratudo, suma	Brazil, Ecuador, Panama, Peru, and Venezuela	Roots	Treatment of arthritis, diabetes, cancer, rheumatism, and ulcers. It is also used as an analgesic, anti‐inflammatory, antistress, antitumor, aphrodisiac, invigorating, memory booster, and tonic	[120, 121, 122, 123, 124, 125, 126]
*Pfaffia townsendii*	Brazilian ginseng	Brazil		It is used as an anti‐inflammatory, tonic, analgesic, and antidiabetic agent	[127]
*Tidestromia oblongifolia*		United States, Mexico		Treatment of headache and foot pain	[128]

**SCHEME 3:**
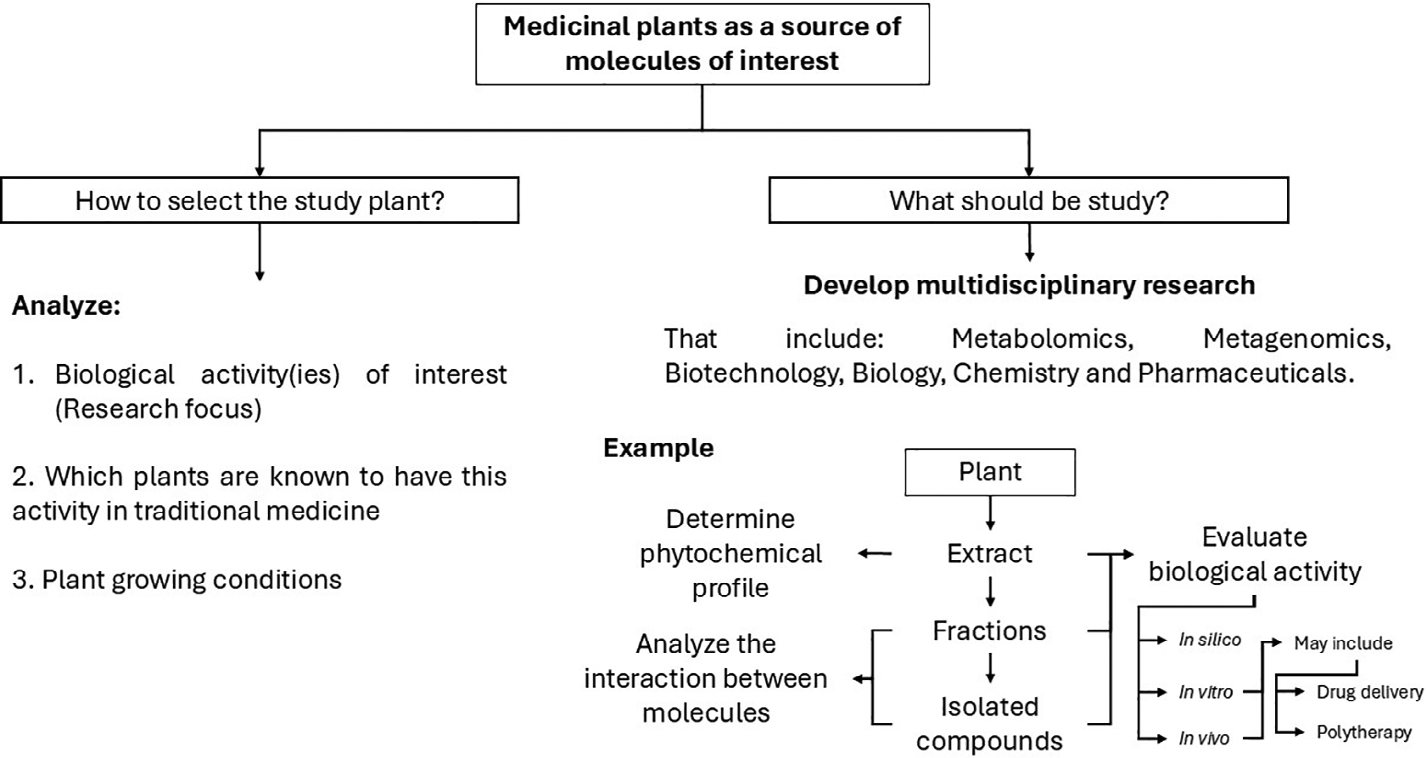
Medicinal plants as a source of molecules of interest. Plants utilized in traditional medicine are excellent candidates for bioprospecting studies; their choice should be based on the objective of the research and uses in traditional medicine, and previous reports, if any. Once the plant material is chosen, it is important to determine the most suitable solvents for extraction. Following this, analyses of chemical profile and biological activity should be conducted. Additionally, it may be beneficial to consider other factors such as drug delivery systems and polytherapy.

It is noteworthy that among the 15 genera of this subfamily, the traditional use of only eight (8) has been documented in the literature, accounting for 53.33%. Most of these plants have been used to treat infections, inflammation, pain and gastrointestinal, respiratory, and skin diseases. In this regard, conducting multidisciplinary research to verify whether these plants really have the potential to treat these conditions, as shown in Scheme 3. To date, only some species belonging to the subfamily have been studied in terms of chemical profile and biotechnological potential, as described in detail in the following sections.

## 4 Phytochemistry

Medicinal plants produce secondary metabolites or phytochemicals, which are responsible for their biological and pharmacological activity [12, 85, 88].

The production, quality, and quantity of phytochemical compounds are influenced by the biotic and abiotic factors present in the environment. Consequently, the phytochemical profile of a plant can vary significantly based on the location and growing conditions.

Furthermore, the concentration of secondary metabolites differs among the different parts of the plant, with the leaves typically exhibiting the highest concentration of phytochemicals [16, 129, 130].

Different natural products and their derivatives have been studied, revealing therapeutic potential for various diseases with fewer side effects than synthetic drugs [14, 131]. In this context, 109 compounds identified within this subfamily have been evaluated for various biological activities, demonstrating significant activity in most instances. Notably, phenolic compounds are the most widely studied, with antimicrobial activity being the most assessed.

In the case of this subfamily until 2024, 512 compounds have been reported, including phenolic compounds, terpenoids, alkaloids, lipid compounds, and other minor compounds, demonstrating the wide chemical diversity present in the members of this subfamily.

A comprehensive review of the bioactive secondary metabolites isolated from Gomphrenoideae, including their sources, structures, and biological properties, is presented below and summarized in Tables 2, 3, 4, 5, 6 and Figure 1. The structures of compounds **63**, **286**, **287**, and **334** are not provided, as there is no available literature presenting them, nor sufficient information to determine them.

**TABLE 2:** Phenolic compounds isolated from the Gomphrenoideae subfamily.

No.	Compound	Species	Parts of plant	References
**Flavonoids**				
**Flavan3‐ols**				
1	Catechin	*Alternanthera bettzickiana*	Aerial parts	[19]
		*Alternanthera philoxeroides*	Whole plant	[132]
		*Alternanthera sessilis*	Whole plant	[57, 133]
		*Gomphrena celosioides* Mart.	Aerial parts	[81]
		*Gomphrena perennis*	Aerial parts	[134]
2	Epigallocatechin	*Alternanthera sessilis*	—	[57]
**Flavones**				
3	Apigenin	*Alternanthera brasiliana*	Leaves	[26]
		*Alternanthera sessilis*	Leaves	[55, 57]
4	Demethyltorosaflavone B	*Alternanthera philoxeroides*	Aerial parts	[38]
5	Demethyltorosaflavone D	*Alternanthera philoxeroides*	Aerial parts and whole plant	[38, 135, 136]
6	5,7‐Dihydroxy‐3,6‐dimethoxyflavone	*Gomphrena boliviana*	Whole plant	[75]
		*Gomphrena martiana*	Whole plant	[75, 100, 137]
7	5,7‐Dihydroxy‐6‐methoxyflavone (oroxilin A)	*Gomphrena boliviana*	Whole plant	[75]
		*Gomphrena martiana*	Whole plant	[75, 137]
8	Dimethoxy‐flavone	*Gomphrena celosioides* Mart.	Aerial parts	[81]
9	5,6‐Dimethoxy‐7‐hydroxyflavone (baicalein 5,6‐dimethyl ether)	*Gomphrena boliviana*	Whole plant	[75]
		*Gomphrena martiana*	Whole plant	[75, 137]
10	3,5‐Dimethoxy‐6,7‐methylenedioxyflavone	*Gomphrena boliviana*	Whole plant	[75]
		*Gomphrena martiana*	Whole plant	[75, 137]
11	Diosmetin	*Froelichia floridana*	Whole plants	[72]
12	Isoorientin	*Alternanthera sessilis*	Leaves	[137]
13	Isorhamnetin	*Alternanthera maritima*	Aerial parts	[138]
		*Gomphrena agrestis*	Roots and leaves	[139]
		*Gomphrena claussenii*	Whole plant	[137]
		*Gomphrena globosa*	Inflorescence	[94, 98]
14	Isovitexin	*Alternanthera maritima*	Aerial parts	[137]
		*Alternanthera sessilis*	Whole plant	[140]
		*Gomphrena perennis*	Aerial parts	[134]
15	Luteolin	*Alternanthera brasiliana*	Leaves	[26]
		*Gomphrena agrestis*	Roots and leaves	[139]
16	Orientin	*Alternanthera brasiliana*	Leaves	[26]
		*Alternanthera sessilis*	Leaves	[137]
17	3,5,6,7‐Tetramethoxyflavone	*Gomphrena boliviana*	Whole plant	[75]
		*Gomphrena martiana*	Whole plant	[75, 137]
**Flavonol**				
18	Galangin triOMe	*Gomphrena martiana*	Whole plant	[137]
19	Gomphrenol	*Blutaparon portulacoides*	Stems	[71]
		*Gomphrena celosioides* Mart.	—	[84]
		*Gomphrena claussenii*	Whole plant	[137]
		*Gomphrena globosa*	Leaves	[137]
20	Kaempferol	*Alternanthera brasiliana*	Leaves, stems, and whole plant	[23, 28]
		*Alternanthera maritima*	Aerial parts	[34, 137]
		*Alternanthera philoxeroides*	Leaves	[38, 141]
		*Alternanthera tenella* Colla	Leaves, stems, and whole plant	[28, 142, 143]
		*Gomphrena agrestis*	Roots and leaves	[139]
		*Gomphrena claussenii*	Whole plant	[137]
		*Gomphrena globosa*	Inflorescence and leaves	[94, 98, 136, 144]
		*Iresine angustifolia*	Whole plant	[103]
21	Kaempferol monosulfate	*Alternanthera sessilis*	Stems	[56]
22	Myricetin	*Alternanthera sessilis*	—	[55]
23	Patuletin	*Gomphrena claussenii*	Whole plant	[137]
24	Quercetin	*Alternanthera bettzickiana*	Aerial parts	[19]
		*Alternanthera brasiliana*	Leaves, stems, and whole plant	[23, 26, 28]
		*Alternanthera maritima*	Aerial parts	[34, 137, 138, 145]
		*Alternanthera paronychioides*	—	[35]
		*Alternanthera philoxeroides*	Leaves	[42, 136]
		*Alternanthera sessilis*	Leaves and whole plant	[55, 133, 137]
		*Alternanthera tenella* Colla	Leaves, stems, and whole plant	[28, 142, 143]
		*Gomphrena agrestis*	Roots and leaves	[139]
		*Gomphrena celosioides*	Roots	[146]
		*Gomphrena claussenii*	Whole plant	[137]
		*Gomphrena globosa*	Inflorescence and leaves	[94, 98, 137]
		*Iresine angustifolia*	Whole plant	[103]
25	Quercetin 3‐methyl ether (3‐methoxy quercetin)	*Alternanthera maritima*	Aerial parts	[138, 145]
		*Alternanthera tenella* Colla	Whole plant	[142]
26	Quercetin‐3‐*O*‐methyl ester	*Alternanthera maritima*	Aerial parts	[34]
27	3,5,3′,4′‐Tetrahydroxy‐6,7‐methylenedioxy flavone	*Gomphrena globosa*	Inflorescence	[94, 98]
**Isoflavone**				
28	Daidzein	*Alternanthera sessilis*	Stem	[56, 57]
29	Daidzin	*Froelichia floridana*	Whole plants	[72]
30	2′,5‐Dimethoxy‐6,7‐methylenedioxyisoflavon (tlatlancuayin)	*Iresine celosioides*	Whole plant	[137]
		*Iresine herbstii*	Aerial parts	[111, 147]
31	Irisone B	*Blutaparon portulacoides*	Aerial parts	[148]
		*Gomphrena celosioides* Mart.	Aerial parts	[81]
32	2′,2,5‐Trimethoxy‐6,7‐methylenedioxyisoflavanone	*Iresine herbstii*	Aerial parts	[111]
**Aurone**				
33	(*E*)‐3′‐*O*‐β‐d‐glucopyranosyl‐4,5,6,4′‐tetrahydroxy‐7,2′‐dimethoxyaurone	*Gomphrena agrestis*	Whole plant	[2]
**Flavonoid glycosides**				
**Flavone glycosides**				
34	Acacetin 8‐C‐[α‐l‐rhamnopyranosyl‐(1 → 2)‐β‐d‐glucopyranoside	*Alternanthera maritima*	Aerial parts	[34, 138, 145]
		*Alternanthera tenella* Colla	Whole plant	[142, 143]
35	Alternanthin	*Alternanthera philoxeroides*	Aerial parts, stems, leaves, and whole plant	[36, 38, 135, 137]
36	Alternanthin B	*Alternanthera philoxeroides*	Aerial parts and whole plant	[36, 38, 135, 136]
37	Apigenin‐6,8‐di‐C‐β‐d‐glucopyranoside	*Alternanthera sessilis*	Stems and whole plant	[56, 149]
38	Chrysin 7‐*O*‐glucuronide	*Gomphrena martiana*	Whole plant	[137]
39	Chrysoeriol‐6‐C‐β‐d‐boivinopyranoside	*Alternanthera philoxeroides*	—	[40]
40	Chrysoeriol‐6‐C‐β‐d‐Boivinopyranosyl‐4′‐*O*‐β‐d‐glucopyranoside	*Alternanthera philoxeroides*	—	[40]
41	Chrysoeriol 7‐*O*‐rhamnoside or chrysoeriol 7‐rhamnoside	*Alternanthera philoxeroides*	Whole plant and aerial parts	[38, 135, 136]
42	Glucopyranosyl‐vitexin	*Alternanthera tenella* Colla	Leaves and stems	[28]
43	2″‐*O*‐β‐d‐glucopyranosyl‐vitexin	*Alternanthera maritima*	Aerial parts	[34, 138, 145]
		*Alternanthera tenella* Colla	Whole plant	[142, 143]
44	Isorhamnetin 3‐*O*‐α‐l‐rhamnosyl‐(1 → 6)‐β‐d‐galactopyranoside	*Alternanthera maritima*	Aerial parts	[34, 138]
45	Isorhamnetin 3‐*O*‐α‐l‐rhamnosyl‐(1 → 6)‐β‐d‐glucopyranoside	*Alternanthera maritima*	Aerial parts	[34]
46	Isorhamnetin‐3‐hexoside	*Gomphrena globosa*	Inflorescence	[73, 94, 98]
		*Gomphrena* sp.	Flower	[73]
47	Isorhamnetin‐3‐(pentosyl)hexoside	*Gomphrena globosa*	Inflorescence	[94, 98]
48	Isorhamnetin‐3‐(6‐rhamnosyl)hexoside	*Gomphrena globosa*	Inflorescence	[94, 98]
49	Isorhamnetin 3‐*O*‐[α‐rhamnopyranosyl‐(1 → 6)‐β‐d‐glucopyranoside]	*Alternanthera maritima*	Aerial parts	[138]
		*Gomphrena celosioides*	Aerial parts	[92]
50	Isorhamnetin 3‐*O*‐α‐l‐rhamnosyl‐(1 → 6)‐β‐d‐glucopyranoside	*Alternanthera maritima*	Aerial parts	[145]
		*Gomphrena globosa* L.	Flower	[144]
51	Isorhamnetin‐3‐*O*‐β‐d‐apiofuranosyl‐(1 → 2)‐β‐d‐glucopyranoside	*Gomphrena globosa* L.	Flower	[144]
52	Isorhamnetin 3‐*O*‐glucoside	*Gomphrena globosa* var. *albiflora*	Flower	[73, 150]
		*Gomphrena* sp.	Flower	[73]
53	Isorhamnetin 3‐*O*‐robinobioside	*Alternanthera maritima*	Aerial parts	[137]
		*Gomphrena martiana*	Whole plant	[137]
54	Isorhamnetin 3‐*O*‐β‐robinobioside	*Gomphrena boliviana*	Whole plant	[75]
		*Gomphrena martiana*	Whole plant	[75]
55	Isorhamnetin 3‐*O*‐rutinoside	*Alternanthera brasiliana*	Leaves and stems	[28]
		*Alternanthera maritima*	Aerial parts	[137]
		*Alternanthera tenella* Colla	Leaves and stems	[28]
		*Gomphrena agrestis*	Roots and leaves	[139]
		*Gomphrena globosa*	Flower	[150]
56	Isorhamnetin‐*O*‐glucuronyl‐deoxyhexosyl‐hexoside	*Gomphrena haageana* K.	Flower	[73]
57	Isorhamnetin‐*O*‐glucuronyl‐hexoside	*Gomphrena haageana* K.	Flower	[73]
58	Luteolin‐6‐C‐β‐d‐boivinopyranoside	*Alternanthera philoxeroides*	—	[40]
59	Luteolin‐6‐C‐β‐d‐boivinopyranosyl‐3′‐*O*‐β‐d‐glucopyranoside	*Alternanthera philoxeroides*	—	[40]
60	Luteolin‐6‐C‐β‐d‐boivinopyranosyl‐4′‐*O*‐β‐d‐glucopyranoside	*Alternanthera philoxeroides*	—	[40]
61	Luteolin 8‐C‐*E*‐propenoic acid	*Alternanthera philoxeroides*	Aerial parts	[38]
62	Luteolin‐8‐C‐rhamnosylglucoside	*Alternanthera brasiliana*	Aerial parts	[22]
63	Methoxy‐trihydroxymethylenedioxyflavone *O*‐glucuronyl‐hexoside	*Gomphrena haageana* K.	Flower	[73]
64	Nepetin 3‐*O*‐rhamnoside	*Alternanthera philoxeroides*	Leaves	[137]
65	Patuletin *O*‐deoxyhexosyl‐hexoside	*Gomphrena haageana* K.	Flower	[73]
66	Patuletin 3‐*O*‐glucoside	*Gomphrena claussenii*	Whole plant	[137]
67	Patuletin 3‐*O*‐β‐d‐glucopyranoside	*Pfaffia townsendii*	Whole plant	[127]
68	Patuletin *O*‐hexoside	*Gomphrena haageana* K.	Flower	[73]
69	2″‐*O*‐pentosyl‐6‐C‐hexosyl‐apigenin (2″‐*O*‐pentosyl‐isovitexin)	*Alternanthera brasiliana*	Leaves	[28]
		*Alternanthera tenella* Colla	Leaves and stems	[28]
70	2″‐*O*‐pentosyl‐8‐C‐hexosyl‐apigenin (2″‐*O*‐pentosyl‐vitexin)	*Alternanthera brasiliana*	Leaves and stems	[28]
		*Alternanthera tenella* Colla	Stems	[28]
71	Potentilin A	*Gomphrena globosa* L.	Flower	[144]
72.	2″‐*O*‐Rhamnopyranosyl‐vitexin	*Alternanthera brasiliana*	Leaves	[28]
		*Alternanthera maritima*	Aerial part	[34, 138, 145]
		*Alternanthera tenella* Colla	Leaves and whole plant	[28, 143]
73	2″‐*O*‐rhamnosyl‐6‐C‐glucosil methylluteolin	*Alternanthera brasiliana*	Aerial parts	[22]
74	2″‐*O*‐rhamnosylvitexin	*Alternanthera brasiliana*	Aerial parts	[22]
		*Alternanthera sessilis*	Stems	[56]
75	2″‐*O*‐Rhamnosylswertisin	*Alternanthera brasiliana*	Aerial parts	[22]
76	3,5,3′,4′‐Tetrahydroxy‐6,7‐methylenedioxyflavone‐3‐*O*‐deoxyhexosyl‐hexoside	*Gomphrena haageana* K.	Flower	[73]
77	3,5,3′,4′‐tetrahydroxy‐6,7‐methylenedioxyflavone‐3‐hexoside	*Gomphrena globosa*	Inflorescence	[94, 98]
		*Gomphrena haageana* K.	Flower	[73]
78	7,3′,4′,5′‐Tetrahydroxy‐flavanone 7‐*O*‐glucoside	*Alternanthera sessilis*	Leaves	[137]
79	Torosaflavone E	*Alternanthera philoxeroides*	Aerial parts and whole plant	[38, 145]
80	3,5,3′‐Trihydroxy‐4′‐methoxy‐6,7‐methylenedioxyflavone	*Blutaparon portulacoides*	Aerial parts, stem, and whole plant	[71, 148, 151, 152]
81	3,5,3′‐Trihydroxy‐4′‐methoxy‐6,7‐methylenedioxy‐favone‐glucosilated	*Blutaparon portulacoides*	Stems and whole plant	[71, 152]
82	3′,4′,7‐Trihydroxy‐6‐methoxyflavone	*Iresine herbstii*	—	[111]
83	3,5,4′‐Trihydroxy‐6,7‐methylenedioxyflavone‐3‐(6‐acetyl)hexoside or Gomphrenol 3‐*O*‐(6‐acetyl)hexoside	*Gomphrena globosa*	Inflorescence	[73, 94, 98, 150]
		*Gomphrena* sp.	Flower	[73]
84	3,5,4′‐Trihydroxy‐6,7‐methylenedioxyflavone‐3‐hexoside or gomphrenol 3‐*O*‐hexoside	*Gomphrena globosa*	Inflorescence	[73, 94, 98, 150]
				
		*Gomphrena* sp	Flower	[73]
85	3,5,4′‐trihydroxy‐6,7‐methylenedioxyflavone‐3‐(2‐pentosyl) hexoside or gomphrenol 3‐*O*‐(2‐pentosyl)‐hexoside	*Gomphrena globosa*	Inflorescence	[73, 94, 98]
		*Gomphrena* sp.	Flower	[73]
86	3,5,4′‐Trihydroxy‐6,7‐methylenedioxyflavone‐3‐(2‐pentosyl, 6‐acetyl)hexoside or gomphrenol 3‐*O*‐(2‐pentosyl, 6‐acetyl)‐hexoside	*Gomphrena globosa*	Inflorescence	[73, 94, 98]
		*Gomphrena* sp.	Flower	[73]
87	3,5,4′‐Trihydroxy‐6,7‐methylenedioxyflavone‐3‐(6‐rhamnosyl)hexoside	*Gomphrena globosa*	Inflorescence	[94, 98]
88	Vitexin	*Alternanthera brasiliana*	Leaves and whole plant	[23, 26, 28]
		*Alternanthera maritima*	Aerial parts	[34, 137, 138]
		*Alternanthera tenella* Colla	Leaves, stems, and whole plant	[28, 142, 143]
89	2″ Vitexin‐*O*‐glucoside	*Alternanthera maritima*	Aerial parts	[137]
90	2″ Vitexin‐*O*‐rhamnoside	*Alternanthera maritima*	Aerial parts	[137]
**Flavonol glycosides**				
91	Gomphrenol‐3‐glucoside	*Blutaparon portulacoides*	Stems and whole plant	[71, 152]
92	Gomphrenol‐3‐*O*‐β‐d‐glucopyranoside	*Gomphrena globosa* L.	Flower	[144]
93	Gomphrenol‐3‐*O*‐β‐d‐glucopyranosyl‐(1 → 6)‐β‐d‐glucopyranoside	*Gomphrena globosa* L.	Flower	[144]
94	Gomphrenol‐3‐*O*‐β‐d‐xylopyranosyl‐(1 → 2)‐β‐d‐glucopyranoside	*Gomphrena globosa* L.	Flower	[144]
95	3′‐Hydroxygomphrenol‐3‐*O*‐β‐d‐glucopyranoside	*Gomphrena globosa* L.	Flower	[144]
96	Kaempferol glucoside	*Alternanthera brasiliana*	Leaves and stems	[28]
		*Alternanthera tenella* Colla	Leaves and stems	[28]
97	Kaempferol‐3‐(2‐pentosyl)hexoside	*Gomphrena globosa*	Inflorescence	[94, 98]
98	Kaempferol‐3‐(2‐pentosyl, 6‐rhamnosyl)hexoside	*Gomphrena globosa*	Inflorescence	[94, 98]
99	Kaempferol‐3‐(6‐rhamnosyl)hexoside	*Gomphrena globosa*	Inflorescence	[94, 98]
100	Kaempferol‐3‐*O*‐α‐l‐rhamnopyranosyl‐(1 → 6)‐β‐dglucopyranoside	*Gomphrena globosa* L.	Flower	[144]
101	Kaempferol‐3‐*O*‐β‐dglucopyranoside	*Gomphrena globosa* L.	Flower	[144]
102	Kaempferol *O*‐acetylhexoside	*Gomphrena globosa* var. *albiflora*	Flower	[73, 150]
		*Gomphrena* sp.	Flower	[73]
103	Kaempferol 3‐*O*‐glucoside	*Alternanthera philoxeroides*	Whole plant	[136]
		*Gomphrena claussenii*	Whole plant	[137]
		*Gomphrena globosa* var. *albiflora*	Flower	[73, 94, 98, 150, 153]
		*Gomphrena* sp.	Flower	[73]
		*Pfaffia glomerata*	Inflorescences	[154]
104	Kaempferol *O*‐glucuronide‐*O*‐hexoside	*Gomphrena globosa*	Flower	[150]
105	Kaempferol 3‐*O*‐(2‐pentosyl)‐hexoside	*Gomphrena globosa* var. *albiflora*	Flower	[73, 150]
		*Gomphrena* sp.	Flower	[73]
106	Kaempferol 3‐*O*‐β‐d‐(6‐*O*‐*p*‐*E*‐coumaroyl)‐glucopyranoside (tiliroside) or kaempferol‐3‐*O*‐(6‐*p*‐coumaroyl)‐glucoside	*Froelichia floridana*	Whole plants	[72]
		*Gomphrena agrestis*	Whole plant	[2]
		*Pfaffia glomerata*	Inflorescences	[154]
		*Pfaffia townsendii*	Whole plant	[127]
107	Kaempferol‐3‐*O*‐(6″‐*O*‐(*E*)‐*p*‐coumaroyl)‐β‐d‐glucopyranoside	*Gomphrena globosa* L.	Flower	[144]
108	Kaempferol‐3‐*O*‐(6″‐*O*‐(*Z*)‐*p*‐coumaroyl)‐β‐d‐glucopyranoside	*Gomphrena globosa* L.	Flower	[144]
109	Kaempferol 3‐*O*‐β‐d‐(6″‐feruloylglucopyranoside)	*Gomphrena globosa* L.	Flower	[144]
110	Kaempferol 3‐*O*‐(2‐pentosyl, 6‐*O*‐rhamnosyl)‐hexoside	*Gomphrena globosa* var. *albiflora*	Flower	[73, 150]
		*Gomphrena* sp.	Flower	[73]
111	Kaempferol‐rhamnosyl‐rhamnosyl‐glycoside	*Alternanthera brasiliana*	Leaves and stems	[28]
		*Alternanthera tenella* Colla	Stems	[28]
112	Kaempferol 3‐*O*‐(2‐rhamnosyl)‐hexoside	*Gomphrena globosa*	Flower	[150]
113	Kaempferol 3‐*O*‐(6‐rhamnosyl)‐hexoside	*Gomphrena globosa* var. *albiflora*	Flower	[73]
		*Gomphrena* sp.	Flower	[73]
114	Kaempferol *O*‐rhamnosyl‐pentoside	*Gomphrena globosa* var. *albiflora*	Flower	[73]
		*Gomphrena* sp.	Flower	[73]
115	Kaempferol 3‐*O*‐robinobioside	*Alternanthera brasiliana*	—	[28]
116	Kaempferol rutinoside or kaempferol 3‐*O*‐rutinoside or kaempferol 3‐rutinoside	*Alternanthera brasiliana*	Leaves and stems	[28]
		*Alternanthera tenella* Colla	Leaves and stems	[28]
		*Gomphrena agrestis*	Roots and leaves	[139]
		*Gomphrena celosioides*	Aerial parts	[92]
		*Gomphrena globosa* var. *albiflora*	Flower	[73, 150, 153]
		*Gomphrena* sp.	Flower	[73]
117	Laricitin 3‐*O*‐β‐d‐glucopyranoside	*Froelichia floridana*	Whole plants	[72]
118	8,8‴‐methylene bis(spinacetin 3‐*O*‐robinobioside)	*Blutaparon portulacoides*	Leaves	[155]
119	Quercetin‐glucoside	*Alternanthera brasiliana*	Leaves and stems	[28]
		*Alternanthera tenella* Colla	Leaves and stems	[28]
120	Quercetin‐3‐hexoside	*Gomphrena globosa*	Inflorescence	[94, 98]
121	Quercetin‐3‐(pentosyl)hexoside	*Gomphrena globosa*	Inflorescence	[94, 98]
122	Quercetin‐3‐(2‐pentosyl)hexoside	*Gomphrena globosa*	Inflorescence	[94, 98]
123	Quercetin‐3‐(2‐pentosyl, 6‐rhamnosyl)hexoside	*Gomphrena globosa*	Inflorescence	[94, 98]
124	Quercetin‐3‐(6‐rhamnosyl)hexoside	*Gomphrena globosa*	Inflorescence	[94, 98]
125	Quercetin 3‐*O*‐(2‐pentosyl, 6‐rhamnosyl)‐hexoside	*Gomphrena globosa* var. *albiflora*	Flower	[73]
		*Gomphrena* sp.	Flower	[73]
126	Quercetin 3‐*O*‐glucoside	*Gomphrena globosa* var. *albiflora*	Flower	[73, 150]
		*Gomphrena haageana* K.	Flower	[73]
		*Gomphrena* sp.	Flower	[73]
		*Pfaffia glomerata*	Inflorescences	[154]
127	Quercetin 3‐OMe	*Alternanthera maritima*	Aerial parts	[137]
128	Quercetin 3‐*O*‐(6‐pentosyl)‐hexoside	*Gomphrena globosa* var. *albiflora*	Flower	[73, 150]
		*Gomphrena* sp.	Flower	[73]
129	Quercetin 3‐*O*‐α‐l‐rhamnosyl‐(1 → 6)‐β‐d‐glucopyranoside	*Alternanthera maritima*	Aerial parts	[145]
130	Quercetin 3‐*O*‐rutinoside (rutin)	*Alternanthera brasiliana*	Aerial parts and stems	[22, 28]
		*Alternanthera maritima*	Aerial parts	[34, 137, 138]
		*Alternanthera sessilis*	Leaves and whole plant	[55, 133]
		*Alternanthera tenella* Colla	Leaves and stems	[28]
		*Gomphrena globosa* var. *albiflora*	Flower	[73, 150, 153]
		*Gomphrena haageana* K.	Flower	[73]
		*Gomphrena* sp.	Flower	[73]
131	Quercetin‐*O*‐acetylhexoside	*Gomphrena* sp.	Flower	[73]
132	Quercetin *O*‐glucuronide‐*O*‐hexoside	*Gomphrena globosa* var. *albiflora*	Flower	[73, 150]
		*Gomphrena* sp.	Flower	[73]
133	Spinacetin 3‐*O*‐robinobioside	*Blutaparon portulacoides*	Leave and whole plant	[152, 155]
**Non‐flavonoid phenolic compounds**				
**Benzoic acids**				
134	Dihydroxybenzoic acid glucoside	*Alternanthera brasiliana*	Leaves and stems	[28]
		*Alternanthera tenella* Colla	Leaves and stems	[28]
135	3,4‐Dimethoxybenzoic acid	*Gomphrena elegans* Mart.	Leaves	[156]
136	3,4‐Dimethylbenzoic acid	*Gomphrena globosa*	—	[156]
137	Ethyl gallate	*Alternanthera sessilis*	—	[57]
138	Gallic acid	*Alternanthera bettzickiana*	Aerial parts	[19]
		*Alternanthera brasiliana*	Leaves	[26]
		*Alternanthera philoxeroides*	Whole plant	[132]
		*Alternanthera sessilis*	—	[55]
		*Gomphrena agrestis*	Roots and leaves	[139]
		*Gomphrena globosa*	Flowers	[153]
		*Gomphrena perennis*	Aerial parts	[134]
		*Iresine angustifolia*	Whole plant	[103]
139	Gentisic acid	*Alternanthera brasiliana*	Leaves and stems	[28]
		*Alternanthera tenella* Colla	Leaves and stems	[28]
140	4‐Hydroxibenzoic acid or *p*‐hydroxybenzoic acid	*Alternanthera brasiliana*	Leaves and stems	[28]
		*Alternanthera philoxeroides*	Aerial parts	[38]
		*Alternanthera sessilis*	Stems	[55, 56, 57]
		*Alternanthera tenella* Colla	Leaves and stems	[28]
		*Gomphrena agrestis*	Roots and leaves	[139]
		*Gomphrena celosioides* Mart.	Aerial parts	[78, 157]
		*Gomphrena elegans* Mart.	Leaves	[156]
141	*p*‐Methoxybenzoic acid	*Gomphrena elegans* Mart.	Leaves	[156]
142	Protocatechuic acid	*Alternanthera sessilis*	Stems	[56]
**Hydroxycinnamic acids–phenolic acids**				
143	Caffeic acid	*Alternanthera brasiliana*	Leaves	[26]
		*Blutaparon portulacoides*	Stems and whole plant	[71, 152]
		*Gomphrena celosioides* Mart.	Aerial parts	[81]
		*Gomphrena globosa*	Flowers	[153]
		*Iresine angustifolia*	Whole plant	[103]
144	Caffeoyl‐glucose	*Gomphrena celosioides* Mart.	Aerial parts	[81]
145	Chlorogenic acid	*Alternanthera bettzickiana*	Aerial parts	[19]
		*Alternanthera brasiliana*	Leaves and stems	[26, 28]
		*Alternanthera philoxeroides*	Leaves	[38, 141]
		*Alternanthera sessilis*	—	[57]
		*Iresine angustifolia*	Whole plant	[103]
146	Cinnamic acid	*Iresine angustifolia*	Whole plant	[103]
147	Coumaric acid	*Alternanthera brasiliana*	Leaves and stems	[28]
		*Alternanthera tenella* Colla	Leaves and stems	[28]
148	*cis*‐*p*‐Coumaric acid	*Gomphrena globosa*	Flowers	
149	Ferulic acid or *trans*‐ferulic acid	*Alternanthera brasiliana*	Leaves and stems	[28]
		*Alternanthera paronychioides*	—	[35]
		*Alternanthera philoxeroides*	Leaves	[38, 141]
		*Alternanthera sessilis*	Leaves	[55, 57]
		*Alternanthera tenella* Colla	Leaves and stems	[28]
		*Blutaparon portulacoides*	Stems and whole plant	[71, 152]
		*Gomphrena celosioides* Mart.	Aerial parts	[81]
		*Gomphrena globosa*	Inflorescence	[94, 98, 150]
		*Iresine angustifolia*	Whole plant	[103]
150	*cis*‐Ferulic acid	*Gomphrena globosa*	Flowers	[150]
151	*cis*‐Ferulic acid hexoside	*Gomphrena globosa*	Flowers	[150]
152	*trans*‐Ferulic acid hexoside	*Gomphrena globosa*	Flowers	[150]
153	Isoferulic acid	*Gomphrena elegans* Mart.	Leaves	
154	*p*‐Coumaric acid or *trans*‐*p*‐coumaric acid	*Gomphrena agrestis*	Roots and leaves	[139]
		*Gomphrena globosa*	Inflorescence	[94, 98, 150]
		*Gomphrena perennis*	Aerial parts	[134]
		*Gomphrena haageana* K.	Flower	[73]
155	Sinapic acid	*Alternanthera bettzickiana*	Aerial parts	[19]
		*Iresine angustifolia*	Whole plant	[103]
156	Vanillic acid	*Alternanthera philoxeroides*	Aerial parts and whole plant	[38, 132]
		*Alternanthera sessilis*	—	[57]
		*Blutaparon portulacoides*	Stems, roots, and whole plant	[71, 148, 152]
		*Gomphrena agrestis*	Roots and leaves	[139]
		*Gomphrena celosioides* Mart.	Aerial parts	[78, 81, 157]
		*Gomphrena elegans* Mart.	Leaves	[156]
**Gallic acid derivatives**				
157	Ellagic acid	*Alternanthera sessilis*	Whole plant	[133]
158	Syringic acid	*Alternanthera philoxeroides*	Leaves	[38, 141]
**Coumarins**				
159	7‐Methoxycoumarin	*Alternanthera caracasana*	Aerial parts	[33]
**Lignans**				
160	Pinoresinol‐4″‐*O*‐β‐d‐glucopyranoside	*Gomphrena celosioides*	Aerial parts	[92]
161	Tortoside A	*Gomphrena celosioides*	Aerial parts	[92]
**Coumarinolignoids**				
162	Cleomiscosin A	*Gomphrena celosioides* Mart.	Leaves	[84]
**Phenylpropanoid**				
163	3,4‐Dihydroxyphenyl caffeate	*Froelichia floridana*	Whole plants	[72]
164	Safrole	*Alternanthera philoxeroides*	Leaves	[141]
**Phenylpropanoid glycosides**				
165	β‐d‐(1‐*O*‐acetyl‐3,6‐*O*‐*p*‐*E*‐dicoumaroyl)‐fructofuranosyl‐α‐d‐(4′‐*O*‐acetyl‐2′‐*O*‐*p*‐*E*‐coumaroyl)‐glucopyranoside	*Froelichia floridana*	Whole plants	[72]
**Sesquiterpene phenol**				
166	Dictyoceratin C	*Gomphrena celosioides* Mart.	Leaves	[84]
**Other phenolic compounds**				
167	2‐Ethyl‐4,5‐dimethylphenol	*Alternanthera sessilis*	Stems	[57]
168	Hydrangeifolin I	*Gomphrena celosioides*	Aerial parts	[92]
169	Hydroxytyrosol	*Alternanthera littoralis* P. Beauv.	Aerial parts	[8]
170	2‐Phenylethyl β‐primeveroside	*Gomphrena celosioides*	Aerial parts	[92]
171	2‐Phenylethyl β‐rutinoside	*Gomphrena celosioides*	Aerial parts	[92]
172	Salicylic acid	*Alternanthera philoxeroides*	Leaves	[38, 141]
		*Gomphrena agrestis*	Roots and leaves	[139]
173	Tannic acid	*Alternanthera philoxeroides*	Whole plant	[132]

**TABLE 3:** Terpenoids compounds isolated from the Gomphrenoideae subfamily.

No.	Compound	Species	Parts of plant	References
**Monoterpenes**				
174	Linalool	*Gomphrena virgata*	Whole plant	[101]
175	(−)‐Loliolide	*Alternanthera brasiliana*	Aerial parts	[22]
176	Myrcene	*Alternanthera philoxeroides*	Leaves	[141]
177	Neryl acetone	*Alternanthera brasiliana*	Aerial parts	[22]
**Monoterpene glycoside**				
178	(+)‐Angelicoidenol‐2‐*O*‐β‐d‐glucopyranoside	*Pfaffia paniculata* Kuntze	Roots	[123]
**Sesquiterpenes**				
179	11,12 Acetonide of 11,12,13‐trihydroxydrimene	*Tidestromia oblongifolia*	Aerial parts	[128]
180	α‐Amorphene	*Gomphrena virgata*	Whole plant	[101]
181	Aromadendrene	*Gomphrena virgata*	Whole plant	[101]
182	δ‐Cadinene	*Gomphrena virgata*	Whole plant	[101]
183	α‐Cadinol	*Gomphrena virgata*	Whole plant	[101]
184	*cis*‐Calamenene	*Gomphrena virgata*	Whole plant	[101]
185	β‐Caryophillene	*Gomphrena virgata*	Whole plant	[101]
186	β‐Elemene	*Gomphrena virgata*	Whole plant	[101]
187	Ilimaquinone	*Gomphrena celosioides* Mart.	Leaves	[84]
188	α‐Ionone	*Alternanthera sessilis*	Leaves	[63]
189	α‐Muurolene	*Gomphrena virgata*	Whole plant	[101]
190	Neodactyloquinone	*Gomphrena celosioides* Mart.	Leaves	[84]
191	Nerolidol	*Gomphrena virgata*	Whole plant	[101]
192	Polygodial	*Tidestromia oblongifolia*	Aerial parts	[128]
193	β‐Selinene	*Gomphrena virgata*	Whole plant	[101]
194	11,12,13‐Trihydroxydrimene	*Tidestromia oblongifolia*	Aerial parts	[128]
195	3β,7α,14‐Trihydroxy‐Δ^8,9^‐drimen‐11,12‐olide	*Iresine diffusa*	Aerial parts	[105]
196	3β,7β,14‐Trihidroxy‐Δ^8,9^‐drimen‐11,12‐olide	*Iresine diffusa*	Aerial parts	[105]
197	6,10,14‐Trimetil‐2‐pentadecanone or phytone	*Alternanthera sessilis*	Stems	[57]
		*Gomphrena virgata*	Whole plant	[101]
**Diterpenoids**				
198	Fitone	*Alternanthera brasiliana*	Aerial parts	[22]
		*Alternanthera sessilis*	Leaf	[158]
199	Gibberellin	*Alternanthera sessilis*	Stems	[56]
200	Jatropone	*Gomphrena elegans* Mart.	Leaves	[156]
201	Neophytadiene	*Alternanthera brasiliana*	Aerial parts	[22]
		*Alternanthera sessilis*	Stems	[57, 158]
202	Phytol	*Alternanthera bettzickiana*	—	[19]
		*Alternanthera brasiliana*	Aerial parts	[22]
		*Alternanthera philoxeroides*	—	[41]
		*Alternanthera sessilis*	Stems and leaves	[57, 158]
**Triterpenes**				
203	α‐Amyrin	*Alternanthera brasiliana*	Aerial parts and whole plant	[22, 23]
		*Alternanthera maritima*	Aerial parts	[138]
204	α‐Amyrin acetate	*Alternanthera brasiliana*	Whole plant	[23]
		*Alternanthera maritima*	Aerial parts	[138]
205	α‐Amyrin‐3‐*O*‐β‐d‐glucopyranoside	*Iresine diffusa*	Aerial parts	[105]
206	β‐Amyrin	*Alternanthera brasiliana*	Aerial parts and whole plant	[22, 23]
		*Alternanthera maritima*	Aerial parts	[138]
207	β‐Amyrin‐3‐*O*‐β‐d‐glucopyranoside	*Iresine diffusa*	Aerial parts	[105]
208	Azadirachtin	*Alternanthera sessilis*	Whole plant	[149]
209	Calenduloside *E* 6′‐methyl ester	*Pfaffia paniculata* Kuntze	Roots	[123]
210	Epitaraxerol	*Gomphrena globosa*	—	[156]
211	Friedelin	*Alternanthera brasiliana*	Whole plant	[23]
		*Alternanthera maritima*	Aerial parts	[138]
212	Glomeric acid	*Pfaffia glomerata*	Roots	[154]
		*Pfaffia paniculata*	—	[159]
213	Gypsogenic acid	*Pfaffia glomerata*	Roots	[115]
214	Handianol	*Alternanthera sessilis*	—	[64]
215	16β‐Hydroxyl‐3‐oxo‐akebonoic acid	*Pfaffia glomerata*	Roots	[115]
216	16β‐Hydroxyl‐3‐oxo‐akebonoic acid 28‐*O*‐β‐d‐glucopyranoside	*Pfaffia glomerata*	Roots	[115]
217	Lupeol	*Alternanthera sessilis*	Leaves	[62, 158]
218	Lupeol acetate	*Alternanthera brasiliana*	Whole plant	[23]
		*Alternanthera maritima*	Aerial parts	[138]
219	Mesembryanthemoidigenic acid	*Pfaffia paniculata* Kuntze	Roots	[123]
220	24‐Methylenecycloartanol	*Alternanthera philoxeroides*	—	[41]
		*Alternanthera sessilis*	—	[64]
221	Oleanolic acid	*Alternanthera philoxeroides*	—	[41]
		*Alternanthera sessilis*	—	[64]
		*Pfaffia glomerata*	Roots	[115, 160]
		*Pfaffia paniculata*	—	[159]
222	Oleanolic acid 28‐*O*‐β‐d‐glucopyranoside	*Pfaffia paniculata* Kuntze	Roots	[123]
223	Oleanonic acid	*Alternanthera philoxeroides*	Aerial parts	[38]
		*Pfaffia glomerata*	Inflorescences	[154]
224	3‐Oxo‐akebonoic acid	*Pfaffia glomerata*	Roots	[115]
225	Pfaffianol A	*Pfaffia glomerata*	Roots	[161]
226	Pfaffic acid	*Hebanthe eriantha*	Roots	[122]
		*Hebanthe paniculata*	—	[121]
		*Pfaffia glomerata*	Roots	[112]
		*Pfaffia paniculata* Kuntze	Roots	[123, 125]
227	Pfaffine A	*Pfaffia paniculata* Kuntze	Roots	[123]
		*Pfaffia glomerata*	Roots and aerial parts	[162]
228	Pfaffine B	*Pfaffia paniculata* Kuntze	Roots and aerial parts	[4, 123]
229	Pfameric acid	*Pfaffia glomerata*	Roots	[154]
		*Pfaffia paniculata* Kuntze	Roots	[123]
230	Serratagenic acid	*Pfaffia glomerata*	Roots	[115]
231	Squalene	*Gomphrena agrestis*	Roots and leaves	[139]
		*Gomphrena elegans* Mart.	Leaves and stem	[156]
232	Taraxerone	*Gomphrena globosa*	—	[156]
233	Taraxerol	*Gomphrena globosa*	—	[156]
**Triterpenoid saponins**				
234	Akebonoic acid	*Pfaffia glomerata*	Roots	[161]
235	Boussingoside A_2_	*Pfaffia glomerata*	Aerial parts and roots	[4 ]
236	Calenduloside E	*Alternanthera philoxeroides*	Whole plant	[38, 41]
		*Pfaffia glomerata*	Roots	
237	Chikusetsusaponin IV	*Pfaffia glomerata*	Inflorescences	[154]
238	Chikusetsusaponin IVa	*Alternanthera philoxeroides*	Aerial parts and whole plant	[4, 41]
		*Pfaffia glomerata*	Roots	[115, 163]
239	2α,3β‐Dihydroxyurs‐12,20(30)‐diene‐28‐oic acid‐3‐α‐l‐arabinopyranosyl‐(1 → 2)‐[β‐d‐xylopyranosyl‐(1 → 3)]‐β‐d‐glucopyranosyl	*Alternanthera repens*	Aerial parts	[4]
240	2α,3β‐Dihydroxyurs‐12,20(30)‐diene‐28‐oic acid‐3‐β‐d‐glucopyranosyl‐(1 → 2)‐α‐l‐arabinopyranosyl‐(1 → 2)‐[β‐d‐xylopyranosyl‐(1 → 3)]‐β‐d‐glucuronopuranoside	*Alternanthera repens*	Aerial parts	[4]
241	2α,3β‐Dihydroxyurs‐12,20(30)‐diene‐28‐oic acid‐3‐β‐d‐quinovopyranosyl‐(1 → 2)‐α‐l‐arabinopyranosyl‐(1 → 2)‐[β‐d‐xylopyranosyl‐(1 → 3)]‐β‐d‐glucopyranosyl	*Alternanthera repens*	Aerial parts	[4]
242	2α,3β‐Dihydroxyurs‐12,20(30)‐diene‐28‐oic acid‐3‐β‐d‐xylopyranosyl‐(1 → 3)‐β‐d‐glucopyranosyl	*Alternanthera repens*	Aerial parts	[4]
243	11α,12α‐epoxy‐3β‐[(*O*‐β‐d‐galactopyranosyl‐(1 → 3)‐*O*‐[β‐d‐glucopyranosyl‐(1 → 2)]‐β‐d‐glucuronopyranosyl)‐oxy]olean‐28,13‐olide	*Gomphrena macrocephala*	Roots	[99]
244	11α,‐12α‐Epoxy‐3β‐[(*O*‐β‐d‐glucuronopyranosyl)oxy]olean‐28,13‐olide	*Gomphrena macrocephala*	Roots	[99]
245	11α,‐12α‐Epoxy‐3β‐[(*O*‐β‐d‐glucuronopyranosyl)oxy]taraxer‐14‐en‐28‐oic acid β‐d‐glucopyranosyl ester	*Gomphrena macrocephala*	Roots	[99]
246	11α,12α‐Epoxy‐3β‐hydroxyolean‐28,13‐olide	*Gomphrena macrocephala*	Roots	[99]
247	11α,12α‐Epoxy‐3β‐hydroxytaraxer‐14‐en‐28‐oic acid	*Gomphrena macrocephala*	Roots	[99]
248	Ginsenoside R_0_	*Pfaffia glomerata*	Inflorescences	[154]
249	3β‐*O*‐(β‐d glucopyranosyluronic acid) 28‐*O*‐β‐d‐Glucopyranosyl oleanolic acid	*Alternanthera sessilis*	—	[64]
250	Gomphrenoside	*Gomphrena globosa*	Aerial parts	[4]
251	Oleanolic acid‐3‐β‐d‐glucopyranosyl	*Alternanthera philoxeroides*	Aerial parts	[4]
252	Oleanolic acid 3‐*O*‐β‐d‐glucuronopyranoside	*Alternanthera philoxeroides*	—	[39]
253	Pfaffiaglycoside A	*Pfaffia glomerata*	Aerial parts and roots	[4, 161]
254	Pfaffiaglycosides B	*Pfaffia glomerata*	Aerial parts and roots	[4, 161, 163]
255	Pfaffoside A	*Hebanthe eriantha*	Roots	[126]
		*Hebanthe paniculata*	Roots	[121]
		*Pfaffia glomerata*	Roots	[112]
		*Pfaffia paniculata*	Roots	[125]
256	Pfaffoside B	*Hebanthe eriantha*	Roots	[126]
		*Hebanthe paniculata*	Roots	[121]
		*Pfaffia glomerata*	Roots	[112]
		*Pfaffia paniculata*	Roots	[125]
257	Pfaffoside C	*Hebanthe eriantha*	Roots	[126]
		*Hebanthe paniculata*	Roots	[121]
		*Pfaffia glomerata*	Roots	[161]
		*Pfaffia paniculata*	Roots	[125]
258	Pfaffoside D	*Hebanthe eriantha*	Roots	[126]
		*Hebanthe paniculata*	Roots	[121]
		*Pfaffia glomerata*	Roots	[112]
		*Pfaffia paniculata*	Roots	[164]
259	Pfaffoside E	*Hebanthe eriantha*	Roots	[126]
		*Hebanthe paniculata*	Roots	[121]
		*Pfaffia glomerata*	Roots	[112]
		*Pfaffia paniculata*	Roots	[164]
260	Pfaffoside F	*Hebanthe eriantha*	Roots	[126]
		*Hebanthe paniculata*	Roots	[121]
		*Pfaffia glomerata*	Roots	[112]
		*Pfaffia paniculata*	Roots	[164]
261	Philoxeroideside A	*Alternanthera philoxeroides*	Aerial parts	[37]
262	Philoxeroideside B	*Alternanthera philoxeroides*	Aerial parts	[37]
263	Philoxeroideside C	*Alternanthera philoxeroides*	Aerial parts	[37]
264	Philoxeroideside D	*Alternanthera philoxeroides*	Aerial parts	[37]
**Carotenoids**				
265	Astaxanthin	*Alternanthera sessilis*	Whole plant	[149]
266	β‐Carotene	*Alternanthera sessilis*	—	[64]
267	Dihydroactinidiolide	*Alternanthera brasiliana*	Aerial parts	[22]
		*Alternanthera sessilis*	Leaf	[158]
		*Gomphrena elegans* Mart.	Leaves	[156]
**Drimene**				
268	3β,14‐Dihydroxy‐Δ^7,8^‐drimen‐11,12‐acetonide	*Iresine diffusa*	Aerial parts	[105]

**TABLE 4:** Alkaloid compounds isolated from the Gomphrenoideae subfamily.

No.	Compound	Species	Parts of plant	References
**Guanidine alkaloids**				
269	Celosiadine A	*Iresine diffusa*	Aerial parts	[165]
270	Celosiadine B	*Iresine diffusa*	Aerial parts	[165]
**Indole alkaloid**				
271	Bruceolline F	*Gomphrena celosioides* Mart.	Leaves	[84]
272	β‐Carboline	*Alternanthera philoxeroides*	Leaves	[42]
**Pyridine alkaloids**				
273	Trigonelline	*Iresine herbstii*	—	[111]
**Alkaloids with phenethylamine nucleus**				
274	Alternamide A (7,8‐dihydroxy‐1,2,4,5‐tetrahydro‐3*H*‐1,5‐ethano[c]azepin‐3‐one)	*Alternanthera littoralis* P. Beauv.	Aerial parts	[8]
275	Alternamide B (6,7‐dihydroxy‐3,4‐dihydroquinoline‐1‐one)	*Alternanthera littoralis* P. Beauv.	Aerial parts	[8]
**Betalains**				
**Amaranthin group (betacyanins)**				
276	Amaranthine (previously named amaranthin)	*Alternanthera bettzickiana*	Leaves	[166]
		*Alternanthera brasiliana*	Leaves	[27, 28]
				
		*Alternanthera ficoidea*	Leaves	[166]
		*Alternanthera tenella*	Leaves and stems	[28]
		Colla		
		*Gomphrena globosa*	Petals and inflorescences	[29, 166, 167, 168]
				
		*Iresine herbstii*	Leaves	[110, 111]
		*Iresine lindenii*	Leaves	[169]
				
277	Celosianin I	*Alternanthera bettzickiana*	Leaves	[166]
278	Celosianin II or celosianin	*Alternanthera bettzickiana*	Leaves	[166]
		*Gomphrena globosa*	Red petals and red flowers	[29, 167, 170]
		*Iresine herbstii*	Leaves	[110]
		*Iresine lindenii*	Leaves	[169]
279	17‐Decarboxy‐amaranthin	*Gomphrena globosa*	Red petals and red flowers	[29, 150, 167, 171]
280	17‐Decarboxy‐isoamaranthine	*Gomphrena globosa*	Red inflorescences	[29, 170]
281	2‴‐*O*‐*E*‐Feruloyl‐iresinin or (2‴‐*O*‐*E*‐feruloyl)‐iresinin I	*Iresine herbstii*	Leaves	[110, 169]
		*Iresine lindenii*	Leaves	[169]
282	2‴‐*O*‐*E*‐Feruloyl‐isoiresinin or (2‴‐*O*‐*E*‐Feruloyl)‐isoiresinin I	*Iresine herbstii*	Leaves	[110]
		*Iresine lindenii*	Leaves	[169]
283	Iresin	*Iresine celosia*	Aerial parts	[104]
		*Iresine diffusa*	Aerial parts	[105]
284	Iresinin previously named iresinin I	*Alternanthera brasiliana*	Leaves	[27]
		*Iresine herbstii*	Leaves	[110, 111, 166, 172]
		*Iresine lindenii*	Leaves	[169]
285	Iresinin II (=isoiresinine I)	*Iresine herbstii*	Leaves	[110, 111, 166]
		*Iresine lindenii*	Leaves	[169]
286	Iresinin III	*Iresine herbstii*	—	[111]
287	Iresinin IV	*Iresine herbstii*	—	[111]
288	Isoamaranthine (Isoamaranthin)	*Alternanthera bettzickiana*	Leaves	[166]
		*Alternanthera brasiliana*	Leaves	[27, 28]
		*Alternanthera ficoidea*	Leaves	[166]
		*Alternanthera tenella* Colla	Leaves and stems	[28]
		*Gomphrena globosa*	Red and orange petals	[29, 167]
		*Iresine herbstii*	Leaves	[110, 111, 166]
		*Iresine lindenii*	Leaves	[169]
289	Isocelosianin or isocelosianin II or (2″‐*O*‐*E*‐sinapoyl)‐amaranthine or lindenin	*Alternanthera bettzickiana*	Leaves	[166]
		*Gomphrena globosa*	Red petals and red flowers	[29, 170 ]
		*Iresine herbstii*	Leaves	[110]
		*Iresine lindenii*	Leaves	[169]
290	Sinapoyl‐amaranthin	*Gomphrena globosa*	Red petals and red flowers	[29, 167, 170]
291	2‴‐*O*‐*E*‐sinapoyl‐iresinin or (2‴‐*O*‐*E*‐sinapoyl)‐iresinin I	*Iresine herbstii*	Leaves	[110]
		*Iresine lindenii*	Leaves	[169]
292	(2″‐*O*‐*E*‐Sinapoyl)‐isoamaranthine or Isolindenin	*Iresine herbstii*	Leaves	[110]
		*Iresine lindenii*	Leaves	[169]
293	2‴‐*O*‐*E*‐sinapoyl‐isoiresinin or (2‴‐*O*‐*E*‐sinapoyl)‐isoiresinin I	*Iresine herbstii*	Leaves	[110]
		*Iresine lindenii*	Leaves	[169]
**Betanin group (betacyanins)**				
294	Betanidin	*Gomphrena globosa*	Red and purple petals	[29]
295	Betanin	*Alternanthera bettzickiana*	Leaves	[166]
		*Alternanthera brasiliana*	Leaves and stems	[27, 28]
		*Alternanthera ficoidea*	Leaves	[166]
		*Alternanthera tenella* Colla	Leaves and stems	[28]
		*Gomphrena globosa*	Red petals	[29, 167]
		*Iresine herbstii*	Leaves	[110]
		*Iresine lindenii*	Leaves	[169]
296	Isobetanidin	*Gomphrena globosa*	Purple petals	[29, 167]
297	Isobetanin	*Alternanthera bettzickiana*	Leaves	[166]
		*Alternanthera brasiliana*	Leaves and stems	[28]
		*Alternanthera ficoidea*	Leaves	[166]
		*Alternanthera tenella* Colla	Leaves and stems	[28]
		*Gomphrena globosa*	Red petals	[29, 167]
		*Iresine herbstii*	Leaves	[110]
		*Iresine lindenii*	Leaves	[169]
**Gomphrenin group (betacyanins)**				
298	*cis*‐Isomer of gomphrenin II	*Gomphrena globosa*	Purple flower	[167, 168, 173]
299	*cis*‐Isomer of gomphrenin III	*Gomphrena globosa*	Pigmented floral parts from the inflorescences	[167, 168, 173, 174]
300	*cis*‐Isomer of isogomphrenin II	*Gomphrena globosa*	Purple petals	[167]
301	*cis*‐Isomer of isogomphrenin III	*Gomphrena globosa*	Purple petals	[167]
302	Gomphrenin	*Gomphrena celosioides*	—	[84]
		*Gomphrena globosa*	Flowers, bract, and bracteoles	[29, 175]
303	Gomphrenin I	*Gomphrena globosa*	Pigmented floral parts from the inflorescences	[94, 98, 166, 168, 173, 174, 176]
304	Gomphrenin II or globosin	*Gomphrena globosa*	Pigmented floral parts from the inflorescences	[94, 98, 150, 166, 167, 168, 173, 174, 176]
		*Iresine herbstii*	Leaves	[110]
305	Gomphrenin III or basellin	*Gomphrena globosa*	Pigmented floral parts from the inflorescences	[94, 98 150, 166, 167, 168, 173, 174, 176]
		*Iresine herbstii*	Leaves	[110]
		*Iresine lindenii*	Leaves	[169]
306	Gomphrenin IV	*Gomphrena globosa*	Inflorescences	[175]
307	Isogomphrenin	*Gomphrena globosa*	Flowers	[175]
308	Isogomphrenin I	*Gomphrena globosa*	Pigmented floral parts from the inflorescences	[94, 98, 166, 168, 173, 174, 176]
309	Isogomphrenin II or Isoglobosin	*Gomphrena globosa*	Inflorescence	[29, 94, 98, 150, 166, 167, 168, 176]
		*Iresine herbstii*	Leaves	[110]
310	Isogomphrenin III or isobasellin	*Gomphrena globosa*	Pigmented floral parts from the inflorescences, bract, and bracteoles	[29, 94, 98, 150, 166, 167, 168, 173, 174, 176–]
		*Iresine herbstii*	Leaves	[110]
		*Iresine lindenii*	Leaves	[169]
311	Isosinapoyl‐gomphrenin I or isogandolin	*Gomphrena globosa*	Purple flowers	[173]
		*Iresine herbstii*	Leaves	[110]
312	Sinapoyl‐gomphrenin I or gandolin	*Gomphrena globosa*	Inflorescence	[94, 98, 167, 168, 173]
		*Iresine herbstii*	Leaves	[110]
313	Sinapoyl‐isogomphrenin I	*Gomphrena globosa*	Inflorescence	[94, 98, 167, 168]
**Other betalains**				
314	Hylocerenin	*Iresine herbstii*	Leaves	[110]
		*Iresine lindenii*	Leaves	[169]
315	Isohylocerenin	*Iresine lindenii*	Leaves	[169]
**Betaxanthins**				
316	Arginine‐betaxanthin	*Gomphrena globosa*	Red petals	[29, 167]
317	Dopamine‐betaxanthine	*Alternanthera brasiliana*	Leaves and stems	[28]
		*Alternanthera tenella Colla*	Leaves and stems	[28]
318	Glutamine‐betaxanthin (vulgaxanthin I)	*Gomphrena globosa*	Red petals	[29, 167]
319	Histidine‐betaxanthin (muscaarin VII)	*Gomphrena globosa*	Red petals	[29, 167]
320	Isoleucine‐betaxanthin	*Gomphrena globosa*	Red petals	[29, 167]
321	Lysine‐betaxanthin	*Gomphrena globosa*	Red petals	[29, 167]
322	3‐Methoxytyramine‐betaxanthin	*Alternanthera brasiliana*	Leaves and stems	[28]
		*Alternanthera tenella* Colla	Leaves and stems	[28]
323	Tryptophan‐betaxanthin	*Gomphrena globosa*	Red petals	[29, 167]
**Other alkaloids**				
324	Alternamine A ((*R*)‐1‐(3,4‐dihydroxyphenyl)‐1,2,3,4‐tetrahydroisoquinoline‐6,7‐diol)	*Alternanthera littoralis* P. Beauv.	Aerial parts	[8]
325	Alternamine B (4‐(2‐aminoethyl)benzene‐1,2‐diol‐4‐(2‐aminoethyl)benzene‐1,2‐diol‐b‐d‐glucopyranose)	*Alternanthera littoralis* P. Beauv.	Aerial parts	[8]
326	Aurantiamide	*Gomphrena celosioides* Mart.	Whole plant	[83–85, 89]
				
327	Aurantiamide acetate	*Gomphrena agrestis*	Whole plant	[2]
		*Gomphrena celosioides* Mart.	Whole plant	[83, 85, 89]
328	*N*‐(3,4‐dihydroxyphenethyl)formamide	*Alternanthera littoralis* P. Beauv.	Aerial parts	[8]
329	*N*‐feruloyl‐tyramine	*Iresine herbstii*	—	[111]
330	Pyrimidine‐2,4 (1*H*, 3*H*)‐dione (uracil)	*Gomphrena elegans* Mart.	Leaves, roots, and stem	[156]

**TABLE 5:** Lipid compounds isolated from Gomphrenoideae subfamily.

No.	Compound	Species	Parts of plant	References
**Fatty acids**				
331	Arachidonic acid	*Alternanthera sessilis*	Whole plant	[149]
332	Butyl hexadecanoate	*Gomphrena elegans* Mart.	Leaves	[156]
333	Ethyl linolenate	*Gomphrena elegans* Mart.	Leaves, root, and stem	[156]
334	Ethyl linoleolate	*Gomphrena elegans* Mart.	Leaves, root, and stem	[156]
335	Ethyl palmitate or ethyl hexadecanoate	*Alternanthera brasiliana*	Whole plant	[23]
		*Alternanthera sessilis*	Stems	[56]
		*Gomphrena elegans* Mart.	Leaves, root, and stem	[156]
		*Pfaffia glomerata*	Roots	[177]
336	Hexadecanoate	*Alternanthera sessilis*	Stems	[56]
337	(8*E*)‐10‐Hydroxy‐8‐octadecenoic acid	*Alternanthera brasiliana*	Stems	[32]
338	(10*E*)‐9‐Hydroxy‐10‐octadecenoic	*Alternanthera brasiliana*	Stems	[32]
339	(8*E*,12*Z*)‐10‐Hydroxy 8,12‐octadecadienoic acid	*Alternanthera brasiliana*	Stems	[32]
340	(9*Z*,11*E*)‐13‐Hydroxy‐9,11‐octadecadienoic acid	*Alternanthera brasiliana*	Stems	[32]
341	(9*Z*,11*E*,15*Z*)‐13‐Hydroxy‐9,11,15‐octadecatrienoic acid	*Alternanthera brasiliana*	Stems	[32]
342	(9*Z*,12*Z*,14*E*)‐16‐Hydroxy‐9,12,14‐octadecatrienoic acid	*Alternanthera brasiliana*	Stems	[32]
343	(9*Z*,13*E*)‐12‐Hydroxy‐9,13‐octadecadienoic acid	*Alternanthera brasiliana*	Stems	[32]
344	(9*Z*,13*E*,15*Z*)‐12‐Hydroxy‐9,13,15‐octadecatrienoic acid	*Alternanthera brasiliana*	Stems	[32]
345	(10*E*,12*E*)‐9‐Hydroxy‐10,12‐Octadecadienoic acid	*Alternanthera brasiliana*	Stems	[32]
346	(10*E*,12*Z*)‐9‐Hydroxy‐10,12‐octadecadienoic acid	*Alternanthera brasiliana*	Stems	[32]
347	Linoleic acid	*Alternanthera bettzickiana*	—	[19]
		*Alternanthera brasiliana*	Aerial parts	[22]
348	Linoleic acid ethyl ester	*Alternanthera brasiliana*	Aerial parts	[22]
349	Methyl linoleate or linoleic acid methyl ester	*Alternanthera sessilis*	Stems	[56]
		*Gomphrena elegans* Mart.	Leaves and stem	[156]
350	Methyl linolenate	*Gomphrena elegans* Mart.	Leaves and stem	[156]
351	Methyl palmitate or methyl hexadecanoate	*Gomphrena celosioides*	Roots	[157]
		*Gomphrena elegans* Mart.	Leaves, stem	[156]
		*Alternanthera sessilis*	Leaves	[158]
352	Methyl stearate	*Gomphrena elegans* Mart.	—	[156]
353	Miristic acid	*Alternanthera brasiliana*	Aerial parts	[22]
		*Gomphrena agrestis*	Roots and leaves	[139]
354	(9*Z*)‐9‐octadecenedioic acid	*Alternanthera brasiliana*	Stems	[32]
355	Oleic acid	*Alternanthera brasiliana*	Whole plant	[23]
356	(7*E*)‐9‐oxo‐7‐octadecenoic acid	*Alternanthera brasiliana*	Stems	[32]
357	(8*E*)‐10‐oxo‐8‐octadecenoic acid	*Alternanthera brasiliana*	Stems	[32]
358	(9*E*,11*E*)‐13‐oxo‐9,11‐octadecadienoic acid	*Alternanthera brasiliana*	Stems	[32]
359	(9*Z*,11*E*)‐13‐oxo‐9,11‐octadecadienoic acid	*Alternanthera brasiliana*	Stems	[32]
360	(10*E*,12*E*)‐9‐oxo‐10,12‐octadecadienoic acid	*Alternanthera brasiliana*	Stems	[32]
361	(10*E*,12*Z*)‐9‐oxo‐10,12‐octadecadienoic	*Alternanthera brasiliana*	Stems	[32]
362	Palmitic acid	*Alternanthera brasiliana*	Aerial parts and whole plant	[22, 23]
		*Alternanthera sessilis*	Stems, leaves, and whole plant	[57, 149, 158]
		*Gomphrena elegans* Mart.	Leaves	[156]
363	Stearic acid	*Alternanthera brasiliana*	Aerial parts and whole plant	[22, 23]
**Fatty alcohol**				
364	1‐Hexadecanol	*Gomphrena elegans* Mart.	Leaves and stem	[156]
**Fatty amide**				
365	Erucamide	*Alternanthera brasiliana*	Aerial parts	[22]

**TABLE 6:** Other compounds isolated from the Gomphrenoideae subfamily.

No.	Compound	Species	Parts of plant	References
**Phytoecdysones**				
366	Ecdysone	*Pfaffia glomerata*	Inflorescences, roots, and aerial parts	[154, 162, 163]
		*Pfaffia paniculata*	Roots	[123]
367	β‐Ecdysone or 1α,20*R*‐dihydroxyecdysone or ecdysterone or 20‐hydroxyecdysone	*Froelichia floridana*	Seeds and whole plant	[72]
		*Gomphrena celosioides*	Roots and aerial parts	[133 ]
		*Gomphrena virgata*	Roots	[101]
		*Pfaffia glomerata*	Inflorescences, stems, roots, and aerial parts	[154, 159, 161, 162, 179, 180, 181, 182, 183, 184]
		*Pfaffia paniculata* Kuntze	Roots	[123]
**Phytoecdysteroids**				
368	Achyranthesterone A	*Froelichia floridana*	Whole plants	[72]
369	Blechnoside B	*Froelichia floridana*	Whole plants	[72]
370.	2‐Dehydro‐3‐epi‐20‐hydroxyecdysone	*Froelichia floridana*	Seeds	[182]
371	2,22‐Dideoxyecdysone 25‐*O*‐β‐d‐glucopyranoside	*Froelichia floridana*	Whole plants	[72]
372	2,22‐Dideoxyecdysone 25‐*O*‐β‐d‐glucopyranosyl‐(1 → 2)‐β‐d‐glucopyranoside	*Froelichia floridana*	Whole plants	[72]
373	(5α)‐2,22‐Dideoxyecdysone 25‐*O*‐β‐d‐glucopyranosyl‐(1 → 2)‐β‐d‐glucopyranoside	*Froelichia floridana*	Whole plants	[72]
374	2,22‐Dideoxy‐20‐hydroxyecdysone 25‐*O*‐β‐d‐glucopyranoside	*Froelichia floridana*	Whole plants	[72]
375	2,22‐dideoxy‐5β‐hydroxyecdysone 25‐*O*‐β‐d‐glucopyranosyl‐(1 → 2)‐β‐d‐glucopyranoside	*Froelichia floridana*	Whole plants	[72]
376	β‐Glucopyranosil oleanolate	*Pfaffia paniculata*	—	[159]
377	22‐Oxo‐20‐hydroxyecdysone	*Pfaffia glomerata*	Roots	[161]
378	Pfaffiaglycoside C	*Pfaffia glomerata*	Roots	[161]
379	Pfaffiaglycoside D	*Pfaffia glomerata*	Roots	[161]
380	Pfaffiaglycoside E	*Pfaffia glomerata*	Roots	[161]
381	Pterosterone	*Pfaffia glomerata*	Roots	[161]
		*Pfaffia paniculata* Kuntze	Roots	[123]
382	Rapisterone	*Pfaffia paniculata* Kuntze	Roots	[123]
383	Rubrosterone	*Pfaffia glomerata*	Roots, aerial parts, and roots	[154, 162]
		*Pfaffia paniculata*	—	[159]
384	Taxisterone	*Pfaffia glomerata*	Roots	[161]
385	2β,3β,14α,17β‐Tetrahydroxy‐5β‐androst‐7‐en‐6‐one	*Pfaffia glomerata*	Roots	[161]
**Phytosterols**				
386	Campesterol	*Alternanthera brasiliana*	Whole plant	[23]
		*Alternanthera maritima*	Aerial parts	[138]
		*Alternanthera sessilis*	—	[62]
		*Alternanthera tenella* Colla	Whole plant	[142]
		*Blutaparon portulacoides*	Aerial parts	[148]
		*Gomphrena celosioides* Mart.	Aerial Parts	[78, 157]
		*Gomphrena globosa*	—	[156]
		*Pfaffia glomerata*	Roots	[177]
387	Campestrol	*Gomphrena celosioides* Mart.	Aerial parts	[81]
388	Cycloeucalenol	*Alternanthera philoxeroides*	—	[41]
		*Alternanthera sessilis*	—	[64]
389	3‐β‐Hydroxystigmast‐5‐en‐7‐one	*Alternanthera philoxeroides*	—	[41]
390	Sitostenone or β‐sitostenone	*Alternanthera brasiliana*	Aerial parts	[22]
		*Gomphrena elegans* Mart.	Leaves	[156]
391	Sitosterol or β‐sitosterol	*Alternanthera brasiliana*	Whole plant and leaves	[23, 28]
		*Alternanthera maritima*	Aerial parts	[138]
		*Alternanthera philoxeroides*	—	[41]
		*Alternanthera sessilis*	—	[62]
		*Alternanthera tenella* Colla	Whole plant	[142]
		*Blutaparon portulacoides*	Aerial parts	[148]
		*Gomphrena agrestis*	Roots and leaves	[139]
		*Gomphrena celosioides* Mart.	Aerial parts	[78, 81, 157]
		*Gomphrena elegans* Mart.	Leaves, roots, and stem	[156]
		*Gomphrena globosa*	—	[156]
		*Hebanthe paniculata*	—	[121]
		*Iresine diffusa*	Aerial parts	[105]
		*Pfaffia glomerata*	Roots	[112, 177]
		*Pfaffia paniculata*	Roots	[183]
		*Tidestromia oblongifolia*	Aerial parts	[128]
392	β‐Sitosteryl‐β‐*O*‐d‐glucopyranoside	*Iresine diffusa*	Aerial parts	[105]
393	γ‐Sitosterol	*Alternanthera brasiliana*	Aerial parts	[22]
394.	Sitosterol glycoside or 3‐*O*‐β‐d‐glucopyranosyl β‐sitosterol	*Alternanthera brasiliana*	—	[32]
		*Alternanthera tenella* Colla	Whole plant	[142]
395	Sitosteryl	*Blutaparon portulacoides*	Roots	[148, 151]
396	Spinasterol or α‐spinasterol	*Alternanthera brasiliana*	Leaves and whole plant	[23, 28]
		*Alternanthera maritima*	Aerial parts	[138]
		*Alternanthera philoxeroides*	—	[41]
		*Alternanthera tenella* Colla	Whole plant	[142]
		*Alternanthera sessilis*	—	[64]
		*Pfaffia glomerata*	Roots	[177]
397	Δ^7^‐Spinasterol	*Pfaffia glomerata*	Roots	[177]
398	β‐Spinasterol	*Alternanthera sessilis*	—	[64]
399	Spinasteryl β‐d‐glucopyranoside	*Blutaparon portulacoides*	Roots	[148]
400	5α‐Stigmasta‐7,22‐dien‐3β‐ol	*Gomphrena elegans* Mart.	Leaves, roots, and stem	[156]
401	5α‐Stigmasta‐enol	*Alternanthera sessilis*	—	[64]
402	5α‐Stigmasta‐7‐enol	*Alternanthera sessilis*	—	[184]
403	Stigmasta 4,6,22 trien‐3‐α‐ol	*Alternanthera brasiliana*	Whole plant	[23]
404	Stigmasta 4,7,22 trien‐3‐β‐ol	*Alternanthera brasiliana*	Whole plant	[23]
405	Δ^7^‐Stigmastenol	*Pfaffia glomerata*	Roots	[177]
406	Stigmast‐7‐en‐3‐ol	*Alternanthera brasiliana*	Aerial parts	[22]
407	Stigmast‐7en‐3β‐ol	*Alternanthera brasiliana*	Whole plant	[23]
		*Alternanthera maritima*	Aerial parts	[138]
408	Stigmast‐22‐en‐3β‐ol	*Tidestromia oblongifolia*	Aerial parts	[128]
409	Stigmast‐7‐enyl	*Blutaparon portulacoides*	Roots	[148]
410	Stigmasterol	*Alternanthera brasiliana*	Leaves and whole plant	[23, 28]
		*Alternanthera maritima*	Aerial parts	[138]
		*Alternanthera sessilis*	—	[62]
		*Alternanthera tenella* Colla	Whole plant	[142]
		*Blutaparon portulacoides*	Aerial parts	[148]
		*Gomphrena agrestis*	Roots and leaves	[139]
		*Gomphrena celosioides* Mart.	Aerial parts and roots	[78, 81, 157]
		*Gomphrena globosa*	—	[156]
		*Hebanthe paniculata*	—	[121]
		*Iresine diffusa*	Aerial parts	[105]
		*Pfaffia glomerata*	Roots	[177]
		*Pfaffia paniculata*	Roots	[183]
		*Tidestromia oblongifolia*	Aerial parts	[128]
411	Δ^7^‐Stigmasterol	*Alternanthera tenella* Colla	Whole plant	[142]
412	Stigmasteryl 3‐β‐*O*‐glucoside 6′‐*O*‐palmitate	*Blutaparon portulacoides*	Roots	[151]
**Phytosteroids**				
413	4,6 Cholestadien‐3‐beta‐ol	*Alternanthera brasiliana*	Whole plant	[23]
**Saponins**				
414	3‐*O*‐β‐d‐glucopyranosyl spinasterol	*Alternanthera tenella* Colla	Whole plant	[142]
415	3‐*O*‐β‐d‐glucopyranosyl stigmasterol	*Alternanthera tenella* Colla	Whole plant	[142]
416	3‐*O*‐β‐d‐glucopyranosyl Δ^7^‐stigmasterol	*Alternanthera tenella* Colla	Whole plant	[142]
417	Stigmast‐6‐en‐3‐*O*‐β‐(d‐glicopiranoside)	*Gomphrena celosioides*	Roots	[157]
**Aliphatic alcohols**				
418	Triacontanol	*Iresine diffusa*	Aerial parts	[105]
**Aliphatic hydrocarbons**				
419	*cis*‐Jasmone	*Gomphrena virgata*	Whole plant	[101]
420	Docosane	*Pfaffia glomerata*	Roots	[177]
421	Docosano	*Gomphrena elegans* Mart.	Roots and stem	[156]
422	Docoseno	*Gomphrena elegans* Mart.	Stem, root and leaves	[156]
423	Eicosene	*Gomphrena elegans* Mart.	Roots and stem	[156]
424	3‐Eicosene	*Gomphrena elegans* Mart.	—	[156]
425	9‐Eicosene	*Gomphrena elegans* Mart.	—	[156]
426	Heptacosane	*Gomphrena elegans* Mart.	Leaves	[156]
427	Heptadecane	*Gomphrena elegans* Mart.	Roots and stem	[156]
428	Hexacosane	*Pfaffia glomerata*	Roots	[177]
429	1‐Hexacosene	*Gomphrena elegans* Mart.	Roots	[156]
430	Hexacosano	*Gomphrena elegans* Mart.	Roots	[156]
431	Hexadecane	*Gomphrena elegans* Mart.	Roots and stem	[156]
432	Nonadecane	*Gomphrena elegans* Mart.	Roots and stem	[156]
		*Pfaffia glomerata*	Roots	[177]
433	Octadecane	*Gomphrena elegans* Mart.	Roots and stem	[156]
		*Pfaffia glomerata*	Roots	[177]
434	1‐Octadecene	*Gomphrena elegans* Mart.	Roots and stem	[156]
435	Pentacosane	*Pfaffia glomerata*	Roots	[177]
436	Pentacosano	*Gomphrena elegans* Mart.	Roots and stem	[156]
437	Pentadecane	*Gomphrena elegans* Mart.	Leaves and stem	[156]
438	Tetracosane	*Pfaffia glomerata*	Roots	[177]
439	Tetracosano	*Gomphrena elegans* Mart.	Roots and stem	[156]
440	7,11,15‐Trimethyl‐3‐methylenehexadec‐1‐ene	*Gomphrena elegans* Mart.	Leaves and stem	[156]
**Alkane**				
441	Eicosane	*Pfaffia glomerata*	Roots	[177]
442	16‐Hentriacontane	*Alternanthera sessilis*	—	[64]
443	Heptadecane	*Gomphrena virgata*	Whole plant	[101]
444	Pentadecane	*Gomphrena virgata*	Whole plant	[101]
445	Tetradecane	*Gomphrena virgata*	Whole plant	[101]
446	2,6,10‐Trimethyldodecane	*Gomphrena virgata*	Whole plant	[101]
**Carboxylic acid**				
447	Citric acid	*Gomphrena agrestis*	Roots and leaves	[139]
		*Gomphrena globosa*	—	[97]
448	Fumaric acid	*Gomphrena agrestis*	Roots and leaves	[139]
		*Gomphrena globosa*	—	[97]
449	3‐(4‐Hydroxyphenyl) methylpropenoate	*Gomphrena celosioides*	Whole plant	[82]
450	Malic acid	*Gomphrena agrestis*	Roots and leaves	[139]
		*Gomphrena globosa*	—	[97]
451	Methylsalicylate	*Gomphrena virgata*	Whole plant	[101]
452	Oxalic acid	*Gomphrena globosa*	—	[97]
453	Quinic acid	*Gomphrena globosa*	Flowers	[153]
**Feruloyl tyramine**				
454	*N*‐*cis*‐feruloyl tyramine	*Alternanthera philoxeroides*	Aerial parts	[36]
455	*N*‐*trans*‐feruloyl tyramine	*Alternanthera philoxeroides*	Aerial parts	[36]
456	*N*‐*trans*‐feruloyl‐3,5‐dimethoxytyramine	*Alternanthera philoxeroides*	Aerial parts	[36]
457	*N*‐*trans*‐feruloyl‐3‐methyldopamine	*Alternanthera philoxeroides*	Aerial parts	[36]
**Heterocyclic compounds**				
458	4*H*‐Pyran‐4‐one,2,3‐dihydro‐3,5‐dihydroxy‐6‐methyl	*Alternanthera sessilis*	Whole plant	[149]
459	2‐Methoxy‐3‐isopropylpyrazine	*Gomphrena virgata*	Whole plant	[101]
**Hydrocarbon**				
460	α‐Copaene	*Gomphrena virgata*	Whole plant	[101]
461	Nonacosane	*Alternanthera sessilis*	—	[64]
		*Gomphrena elegans* Mart.	Leaves	[156]
462	Tricosano	*Gomphrena elegans* Mart.	Roots	[156]
**Organic acid**				
463	Gluconic acid	*Pfaffia glomerata*	Inflorescences and aerial parts	[154, 162]
**Tocopherols**				
464	α‐Tocopherol	*Alternanthera bettzickiana*	Aerial parts	[19]
		*Alternanthera brasiliana*	Aerial parts	[22]
		*Gomphrena agrestis*	Roots and leaves	[139]
		*Gomphrena globosa*	—	[97]
465	α‐Tocopherol acetate	*Alternanthera brasiliana*	Aerial parts	[22]
466	γ‐Tocopherol	*Alternanthera bettzickiana*	Aerial parts	[19]
		*Gomphrena globosa*	—	[97]
467	δ‐Tocopherol	*Gomphrena agrestis*	Roots and leaves	[139]
		*Gomphrena globosa*	—	[97]
**Vitamins**				
468	Riboflavin	*Alternanthera brasiliana*	—	[185]
469	Niacin	*Alternanthera brasiliana*	—	[185]
**Other types of compounds**				
470	Allantoin	*Hebanthe paniculata*	—	[121]
		*Pfaffia paniculata*	Roots	[183]
471	Benzophenone‐4	*Alternanthera sessilis*	Stem	[56]
472	Butyrolactone	*Alternanthera sessilis*	Stems	[57]
473	Choline	*Alternanthera sessilis*	—	[64]
474	2‐Decenal	*Alternanthera brasiliana*	Whole plant	[23]
475	Diisobutil Phthalate	*Gomphrena elegans* Mart.	Stem	[156]
476	7,9‐di‐ter‐butyl‐oxaspiro [4, 5] deca‐6,9‐dien‐2,8‐dione	*Gomphrena elegans* Mart.	Roots	[156]
477	2,4‐Dihydroxy‐2,5‐dimethyl‐3(2*H*)‐furan‐3‐one	*Alternanthera sessilis*	Stems	[57]
478	11‐(3‐Ethenylcyclopentyl)undec‐10‐enoic acid, ethyl ester	*Alternanthera sessilis*	Stems	[57]
479	3‐Ethyl‐5‐(2‐ethylbutyl)‐octadecane	*Gomphrena elegans* Mart.	Roots	[156]
480	Ethyl 9‐octadecenoate	*Gomphrena elegans* Mart.	Leaves, root, and stem	[156]
481	Formic acid, 2‐propenyl ester	*Alternanthera sessilis*	Stems	[57]
482	Furfural	*Alternanthera sessilis*	Stems	[57]
483	β‐Glucopyranosyl oleanolate	*Pfaffia glomerata*	Roots	[154]
484	l‐Glutamic acid	*Alternanthera sessilis*	Stems	[57]
485	Glutamine, L‐	*Alternanthera sessilis*	Stems	[57]
486	Glycinebetaine	*Iresine herbstii*	—	[111]
487	Heneicosane	*Pfaffia glomerata*	Roots	[177]
488	1‐Hexadecene	*Gomphrena elegans* Mart.	—	[156]
489	(*E*)‐hexyl 2‐methylbut‐2‐enoate	*Gomphrena virgata*	Whole plant	[101]
490	*p*‐Hydroxycinnamoyl moiety	*Alternanthera sessilis*	Stems	[56]
491	Indole‐3‐carbaldehyde	*Alternanthera philoxeroides*	Aerial parts	[38]
492.	Inulin	*Pfaffia glomerata*	—	[186]
493	Laurenan‐2‐ona	*Gomphrena elegans* Mart.	Leaves	[156]
494	Levan	*Gomphrena marginata*	—	[186]
495	8‐Methyl‐1‐decene	*Gomphrena elegans* Mart.	Stem	[156]
496	Methyl octadecanoate	*Gomphrena elegans* Mart.	Leaves	[156]
		*Alternanthera sessilis*	Leaves	[158]
497	Methyl 6‐octadecenoate	*Gomphrena elegans* Mart.	Stem	[156]
498	Methyl 8‐octadecenoate	*Gomphrena elegans* Mart.	Leaves	[156]
499	3‐Methyl‐5‐propylnonane	*Alternanthera brasiliana*	Aerial parts	[22]
500	Nonanal	*Gomphrena virgata*	Whole plant	[101]
501	*Z*‐3,17‐Octadecadien‐1‐ol acetate	*Alternanthera sessilis*	Stems	[57]
502	Palmitic acid ethyl ester	*Alternanthera brasiliana*	Aerial parts	[22]
503	Pentanal	*Alternanthera sessilis*	Stems	[57]
504	Phaeophytin a	*Alternanthera philoxeroides*	—	[41]
505	Phenylacetaldehyde	*Alternanthera sessilis*	Stems	[57]
506	Pheophytin a′	*Alternanthera philoxeroides*	—	[41]
507	Sebacic acid, bis(2‐ethylhexyl) ester	*Alternanthera sessilis*	Stems	[57]
508	6,10,14‐Trimethyl‐2‐pentadecanone	*Gomphrena elegans* Mart.	Leaves and roots	[156]
509	(5*Z*)‐2,6,10‐Trimethyl‐1,5,9‐undecatriene	*Alternanthera brasiliana*	Aerial parts	[22]
510	1,2,4‐Trioxolane,3‐phenyl‐	*Alternanthera sessilis*	Stems	[57]
511	Uridine	*Gomphrena elegans* Mart.	Leaves	[156]
512	Umbellatosides B	*Gomphrena celosioides*	Aerial parts	[178]

FIGURE 1Chemical compounds identified in the subfamily Gomphrenoideae.

**Figure:**
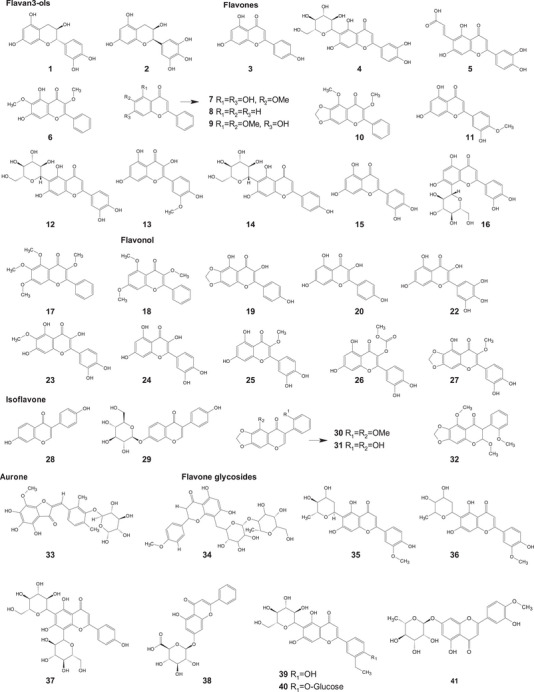

**Figure:**
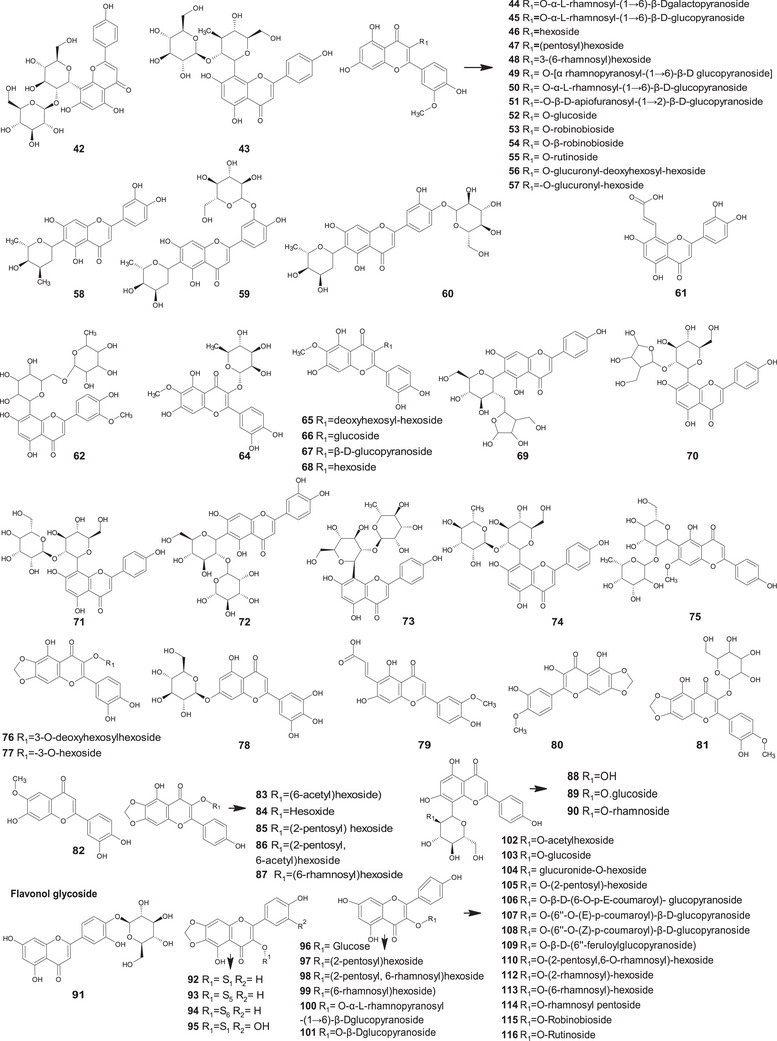

**Figure:**
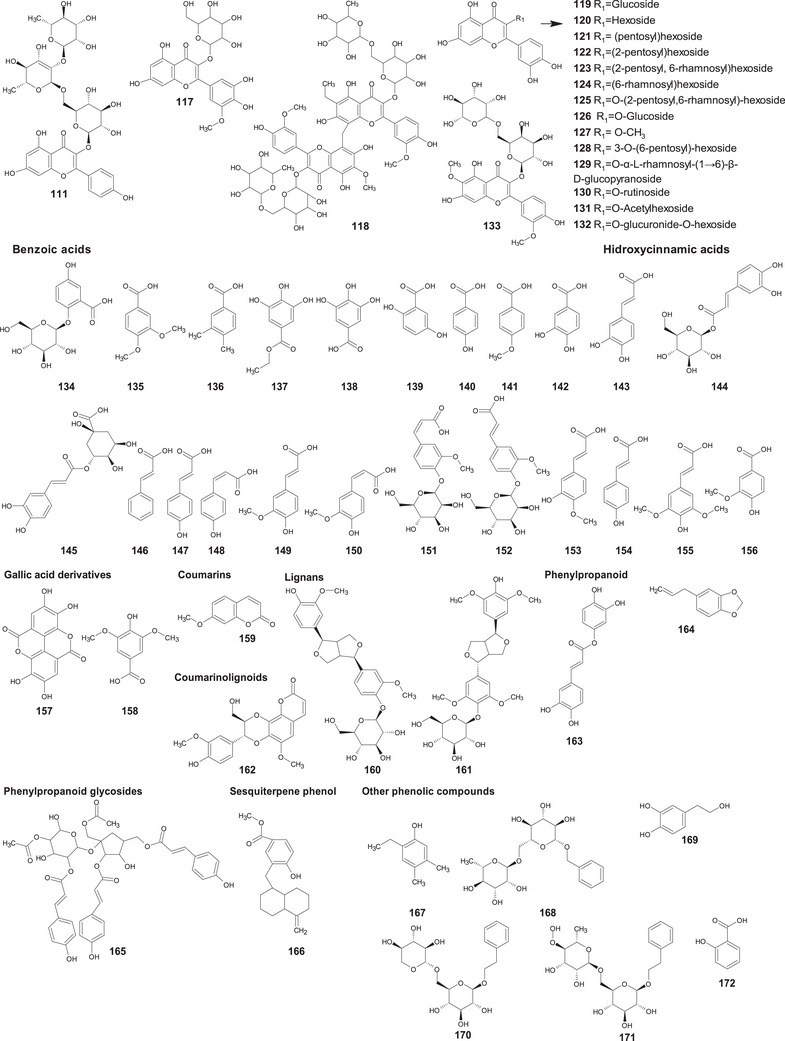

**Figure:**
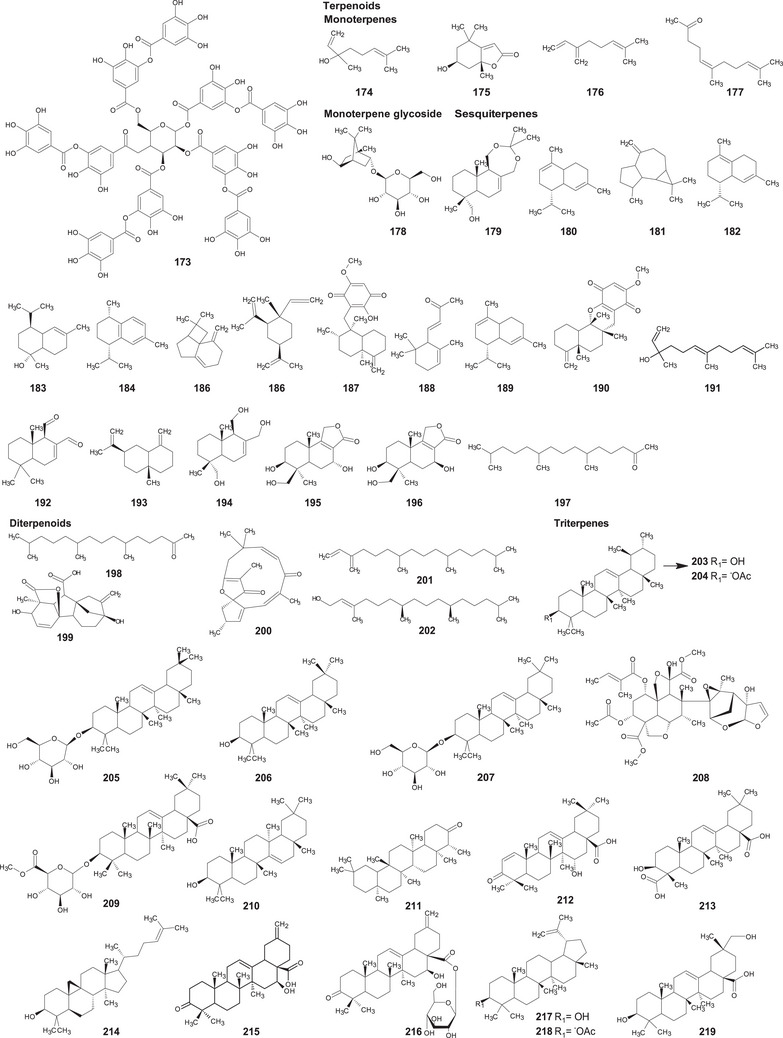

**Figure:**
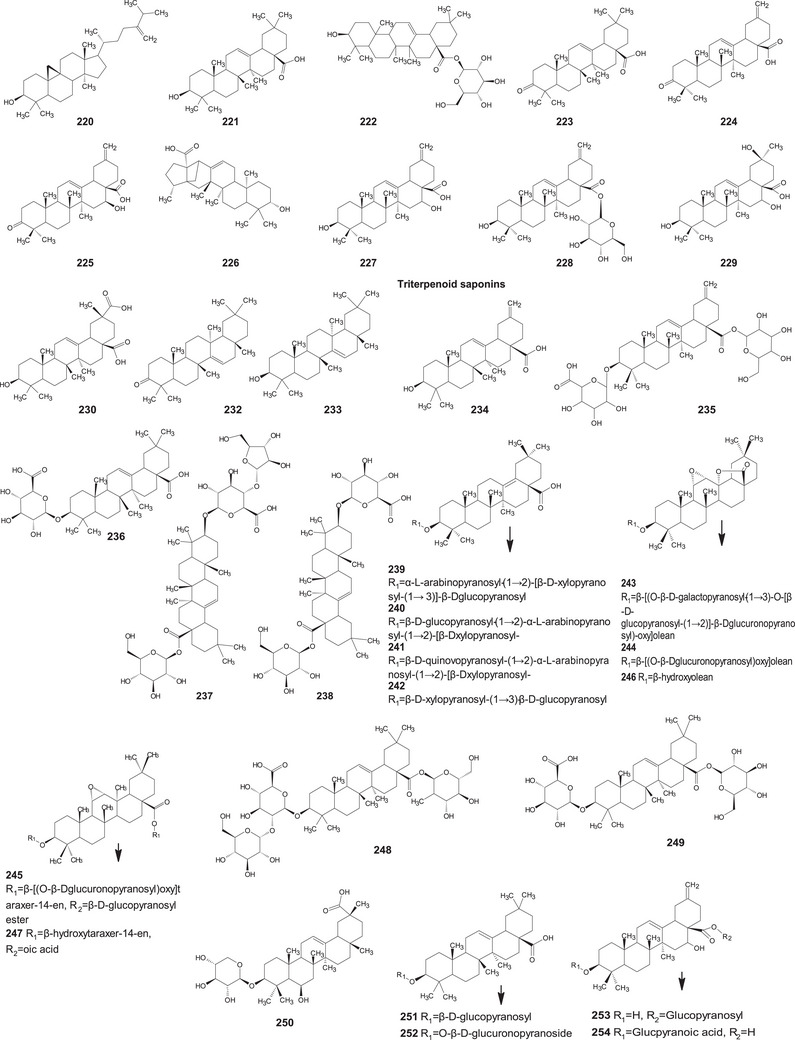

**Figure:**
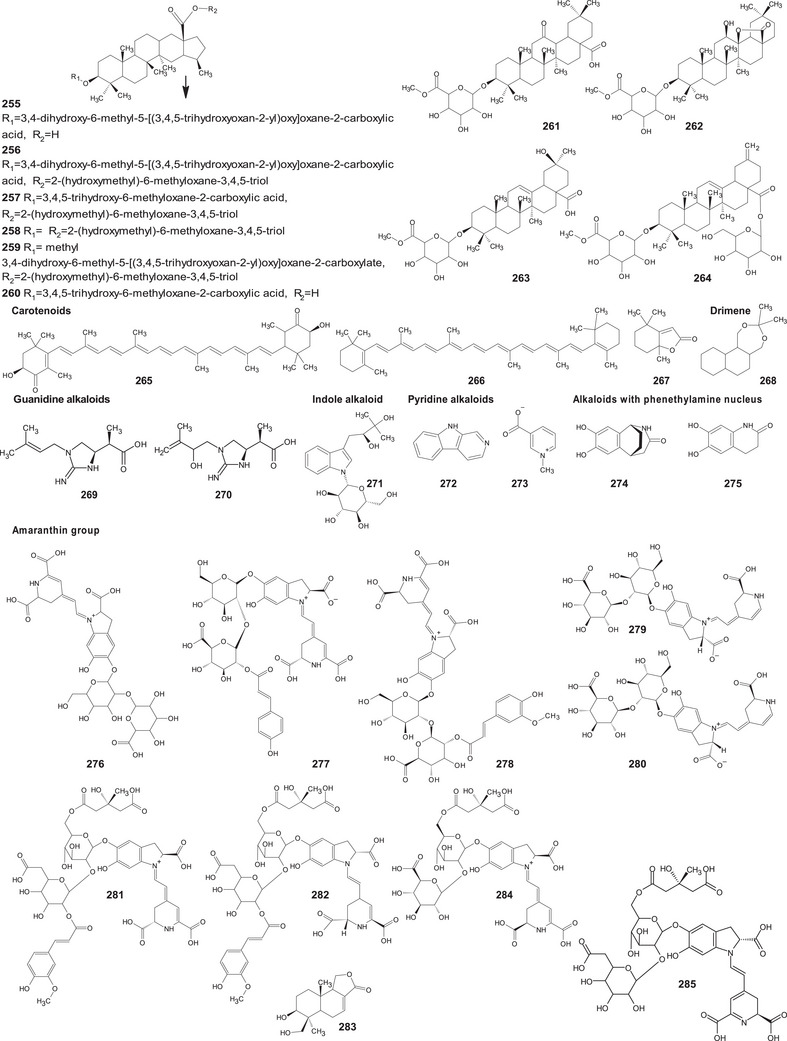

**Figure:**
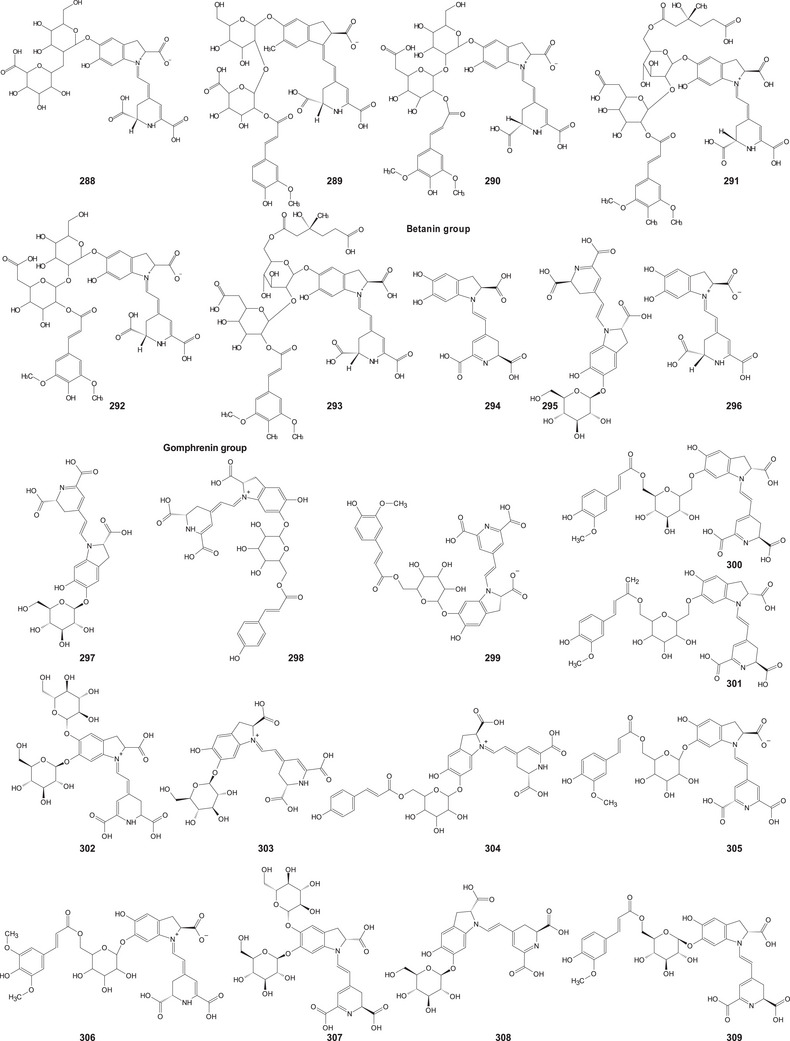

**Figure:**
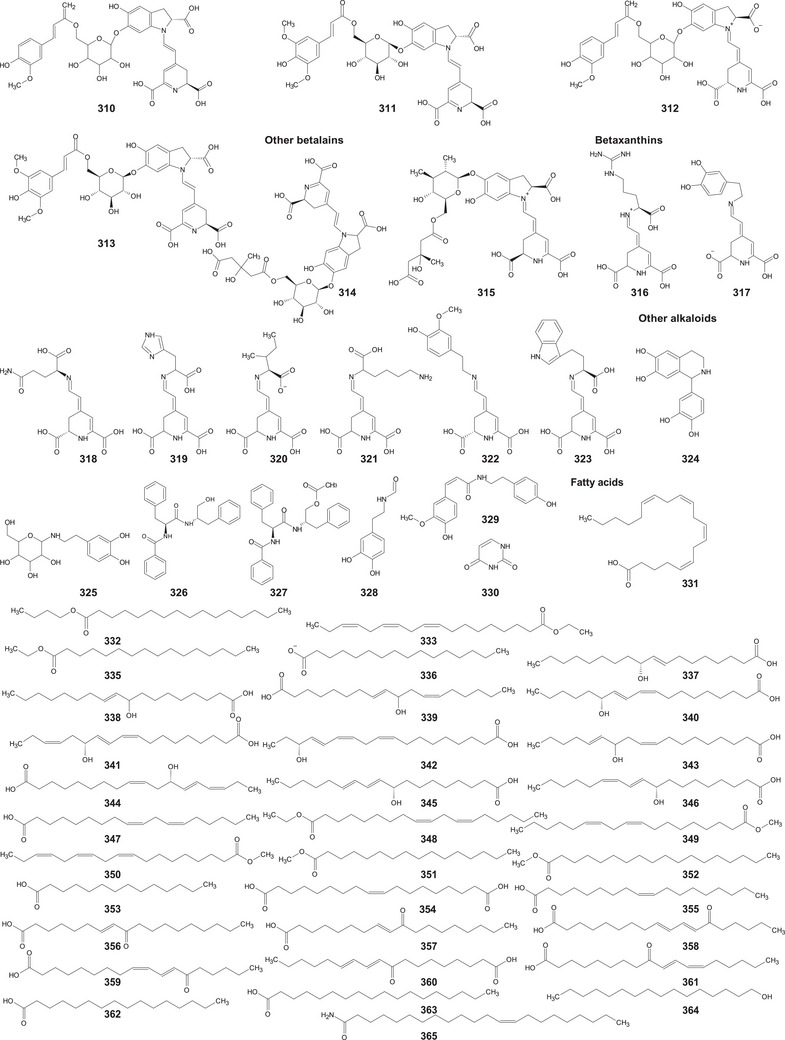

**Figure:**
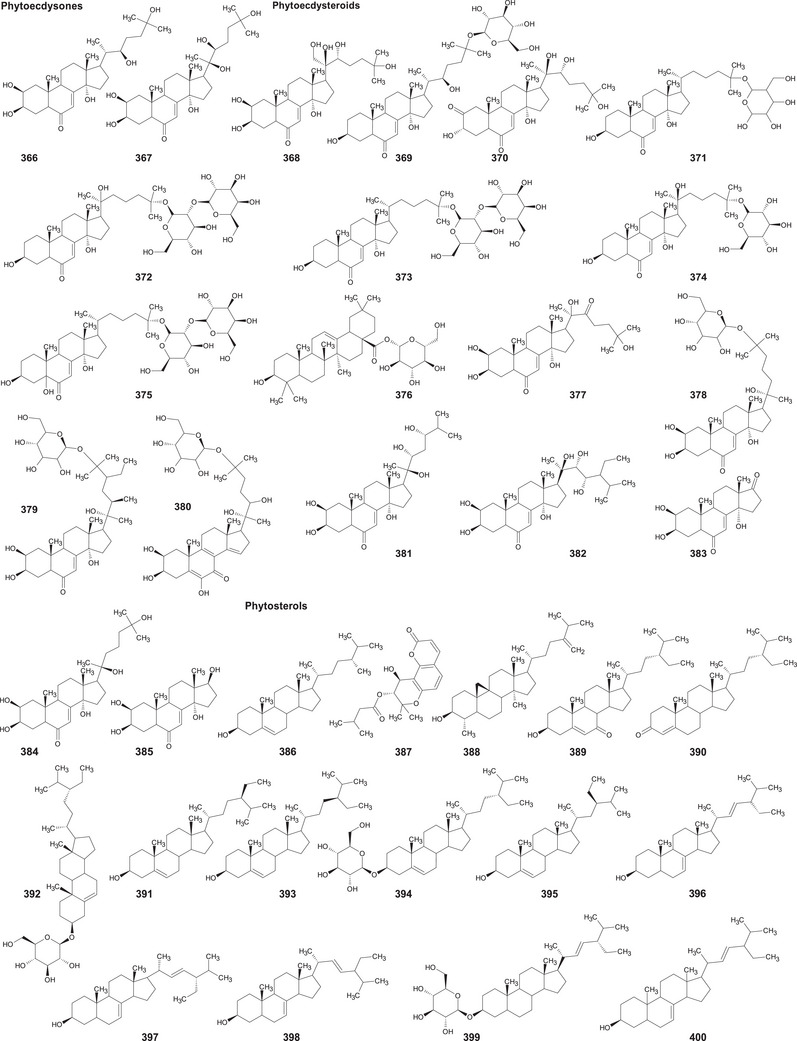

**Figure:**
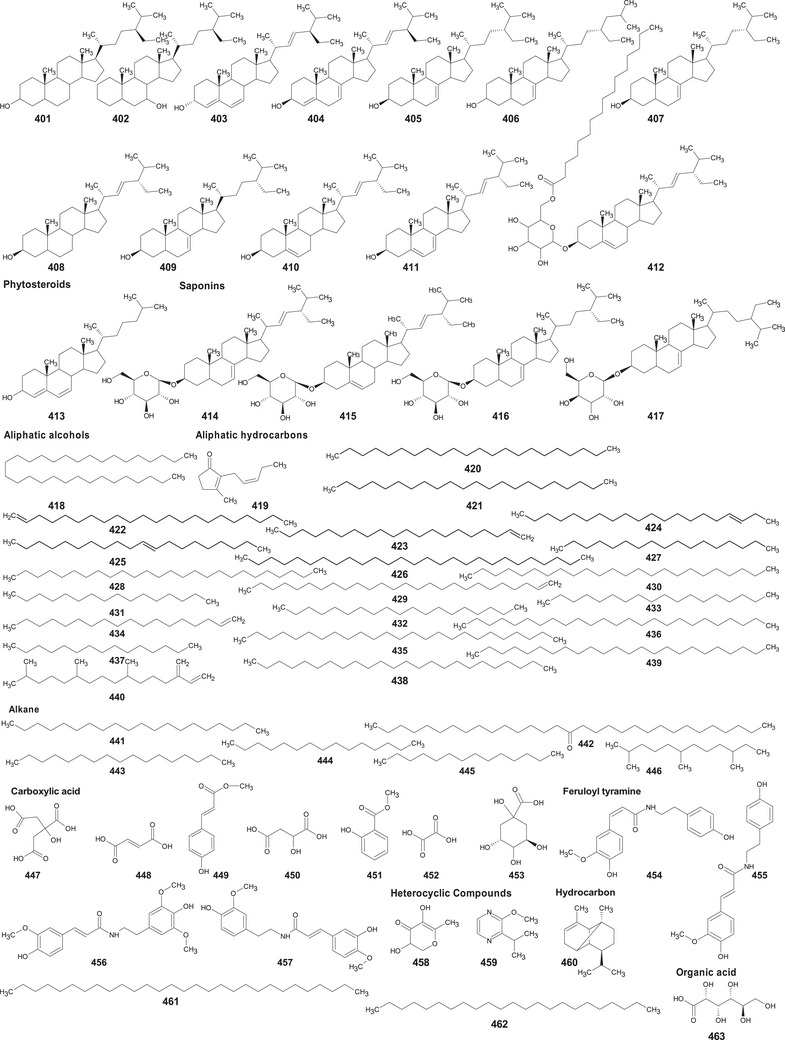

**Figure:**
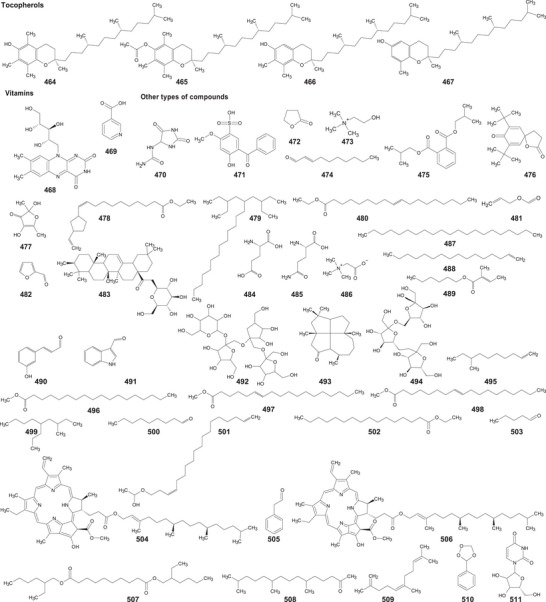

Currently, only 8.22% of the members of Gomphrenoideae have a documented phytochemical profile. Notably, *Gomphrena globosa* L. has been found to contain 107 compounds, representing 20.94% of the total compounds reported in Gomphrenoideae, including phenolic compounds, terpenoids, alkaloids, lipid compounds, carboxylic acids, and tocopherols. Similarly, *Alternanthera brasiliana* (L.) Kuntze exhibits a remarkable phytochemical profile with 91 secondary metabolites, including phenolic compounds, terpenoids, alkaloids, lipid compounds, tocopherols, vitamins, among other compounds. In contrast, for other species, fewer than 10 chemical compounds have been reported, examples include *Alternanthera paronychioides* A.St.‐Hil. (**24**, **149**), *Alternanthera repens* (Synm. *Alternanthera caracasana* Kunth) (**159**, **239**–**242**), *Gomphrena boliviana* Moq. (**6**, **7**, **9**, **10**, **17**, **54**), *Gomphrena claussenii* Moq. (**13**, **19**, **20**, **23**–**24**, **66**, **96**), *Gomphrena macrocephala* A.St.‐Hil. (**243**–**247**), *Gomphrena marginata* Seub. (**494**), *Gomphrena martiana* Gillies ex Moq. (**6**–**7**, **9**–**10**, **17**–**18**, **38**, **53**–**54**), *Pfaffia townsendii* Pedersen (67, 106), and *Tidestromia oblongifolia* (S. Watson) Standl. (Synm. *Tidestromia suffruticosa* var. oblongifolia (S. Watson) Sánch.Pino & Flores Olv.) (**179**, **192**, **194**, **391**, **408**, **410**).

However, no studies have been conducted on the phytochemical profile of species within the genera *Froelichiella*, *Guilleminea*, *Hebanthodes*, *Pedersenia*, *Pseudoplantago*, *Quaternella*, and *Xerosiphon*, leaving them chemically unexplored.

Determining the chemical profile of a plant is crucial, as it allows for the deduction of its biological activity, safety, and toxicity. Additionally, it facilitates the study of how internal and external factors influence the production of secondary metabolites and, consequently, biological activity. Research has demonstrated that plants of the same species collected from different locations or at different times, as well as subspecies, can exhibit different phytochemical profiles.

In accordance with the above, it is also worth mentioning that in many cases, the compounds identified in the chemical profile of a plant are produced by its endophytic microorganisms or by the interaction plant‐microorganisms. This association has become a new area of multidisciplinary research of high biotechnological interest.

It is important to emphasize that, to date, there are no studies that evaluate the effect of biotic and abiotic factors on the chemical profile of the members of this subfamily. Additionally, there are no reports on the endophytic communities present in these plants and their effect on the production of secondary metabolites.

### 4.1 Phenolic Compounds

Phenolic compounds are the most numerous and ubiquitously distributed group of secondary plant metabolites [73]. This group includes all substances that contain phenolic functions linked to aromatic or aliphatic structures [187].

These compounds exhibit a broad range of biological effects mainly related to their antioxidant capacity due to the presence of hydrogen‐donating hydroxyl groups [73, 77, 90, 188]. Previous studies have reported that these compounds inhibit cellular DNA mutagenicity and possess antimicrobial and anti‐inflammatory activities [73, 85, 188].

To date, 173 phenolic compounds have been isolated from the Gomphrenoideae subfamily. In this review, these compounds have been classified into two categories: flavonoids and their glycosides (**1**–**133**) and non‐flavonoid phenolic compounds (**134**–**173**), as detailed in Sections 4.1.1 and 4.1.2, Table 2, and Figure 1.

Some of these compounds have been subjected to in vivo and in vitro studies to evaluate their biological activity, aiming to propose them as potential therapeutic agents for various diseases.

In terms of antimicrobial activity, compounds 5,7‐dihydroxy‐3,6‐dimethoxyflavone (**6**), oroxilin A (**7**), baicalein 5,6‐dimethyl ether (**9**), 3,5‐dimethoxy‐6,7‐methylenedioxyflavone (**10**), and 3,5,6,7‐tetramethoxyflavone (**17**) were evaluated against *Mycobacterium phlei*, *Staphylococcus aureus* ATTC 12600, and *Enterococcus faecalis* ATCC 19433. All were more active against *M. phlei*, with compounds **6** and **7** presenting the highest activity, with an MIC of 15 µg/mL [75]. Likewise, these compounds were evaluated against *Aspergillus niger*, *Candida albicans*, and *Saccharomyces cerevisiae* at concentrations of 125–4000 µg/mL and showed MICs ranging from 250 to 1000 µg/mL [75]. Compounds kaempferol (**20**), quercetin (**24**), quercetin 3‐methyl ether (3‐methoxy quercetin) (**25**), acacetin 8‐C‐[α‐l‐rhamnopyranosyl‐(1 → 2)‐β‐d‐glucopyranoside (**34**), 2″‐*O*‐β‐d‐glucopyranosyl‐vitexin (**43**), 2″‐*O*‐rhamnopyranosyl‐vitexin (**72**), and vitexin (**88**) were assessed against 15 gram‐positive and 4 gram‐negative bacteria, 7 yeast, and 4 dermatophytes at concentrations of 50–500 µg/mL. All these compounds showed activity against at least 3 microorganisms, with compound **24** showing the broadest spectrum, exhibiting activity against 19 of the 30 microorganisms evaluated [142].

Compounds (*E*)‐3′‐*O*‐β‐d‐glucopyranosyl‐4,5,6,4′‐tetrahydroxy‐7,2′‐dimethoxyaurone (**33**) and kaempferol 3‐*O*‐β‐d‐(6‐*O*‐*p*‐*E*‐coumaroyl)‐glucopyranoside (tiliroside) or kaempferol‐3‐*O*‐(6‐*p*‐coumaroyl)‐glucoside (**106**) were tested against 19 bacteria but only showed activity against four microorganisms in a concentration range of 0.02–0.5 mg/mL [2]. Compound 3,5,3′‐trihydroxy‐4′‐methoxy‐6,7‐methylenedioxyflavone (**80**) was evaluated against 15 microorganisms and showed activity against 7 microorganisms, with the lowest MIC observed against *Streptococcus mutans* 11.22.1 (20 µg/mL) and the highest in *S. aureus* ATCC 6538 (1250 µg/mL) [151]. Compounds kaempferol (**20**), kaempferol‐3‐*O*‐(6″‐*O*‐(*E*)‐*p*‐coumaroyl)‐β‐d‐glucopyranoside (**107**), kaempferol‐3‐*O*‐(6″‐*O*‐(*Z*)‐*p*‐coumaroyl)‐β‐d‐glucopyranoside (**108**), and kaempferol 3‐*O*‐β‐d‐(6″‐feruloylglucopyranoside) (**109**) were evaluated against *Pseudomonas aeruginosa*. Among these, compound **20** showed the highest activity with an MIC of 0.008 mg/mL, which was lower than that of ceftriaxone sodium [144].

The compound vanillic acid (**156**) was evaluated against five (5) bacteria at a concentration of 0.2 mg/disc and showed activity against two (2) of these microorganisms [157]. The compound 7‐methoxycoumarin (**159**), identified in *A. caracasana*, was evaluated in vitro for antimicrobial activity against *Bacillus subtilis*, *S. aureus* ATCC 12398, *Staphylococcus epidermidis*, *Sarcina lutea*, and *Vibrio cholerae* No. 01 ATCC 35971, showing MIC between 0.5 and 0.75 mg/mL, highlighting that this compound demonstrated an MCB of 1 mg/mL against *V. cholerae* No. 01 ATCC 35971 [33].

Compounds chrysoeriol‐6‐C‐β‐d‐boivinopyranoside (**39**), chrysoeriol‐6‐C‐β‐d‐boivinopyranosyl‐4′‐*O*‐β‐d‐glucopyranoside (**40**), luteolin‐6‐C‐β‐d‐boivinopyranoside (**58**), luteolin‐6‐C‐β‐d‐boivinopyranosyl‐3′‐*O*‐β‐d‐glucopyranoside (**59**), and luteolin‐6‐C‐β‐d‐boivinopyranosyl‐4′‐*O*‐β‐d‐glucopyranoside (**60**) were evaluated for antiviral activity. It was evident that compounds **40**, **59**, and **60** blocked the secretion of HBsAg, as detailed in subsequent sections [40]. The compound (**80**) was evaluated as an antiparasitic against *Trypanosoma cruzi* and *Leishmania amazonensis* at concentrations between 4 and 500 µg/mL, but they showed a low reduction in viability [151].

The results described show that the phenolic compounds exhibit antimicrobial activity at the laboratory level. Most were evaluated against bacteria, followed by yeasts, fungi, viruses, and, to a lesser extent, parasites, highlighting the potential of these compounds as a promising source of antimicrobials.

The isolated phenolic compounds of this subfamily have also been assessed for antioxidant activity. Compounds patuletin 3‐*O*‐β‐d‐glucopyranoside (**67**) and kaempferol‐3‐*O*‐(6″‐*O*‐(*E*)‐*p*‐coumaroyl)‐β‐d‐glucopyranoside (**106**), as well as a mixture of these two, were evaluated using the DPPH and ORAC assays, both compound **67** and the mixture exhibited relevant activity [127]. Compounds quercetin (**24**), quercetin 3‐methyl ether (3‐methoxy quercetin) (**25**), acacetin 8‐C‐[α‐l‐rhamnopyranosyl‐(1 → 2)‐β‐d‐glucopyranoside (**34**), 2″‐*O*‐β‐d‐glucopyranosyl‐vitexin (**43**), isorhamnetin 3‐*O*‐α‐l‐rhamnosyl‐(1 → 6)‐β‐d‐glucopyranoside (**50**), 2″‐*O*‐rhamnopyranosyl‐vitexin (**72**), and quercetin 3‐*O*‐α‐l‐rhamnosyl‐(1 → 6)‐β‐d‐glucopyranoside (**129**) were evaluated using chemiluminescent assays, showing activity, with compounds **24** and **25** exhibiting greater activity [145]. Similarly, compounds **34**, **43**, **70**, and **88** were evaluated using ORAC, demonstrating their antioxidant potential, where compound **88** showed the best activity with 0.96 relative TE [143]. Although there have been few studies focused on studying the antioxidant potential of the phenolic compounds identified and isolated from this subfamily, the research conducted to date demonstrates that they are a promising source of antioxidant compounds.

Regarding cytotoxic activity against cancer cell lines, the mixture of compounds 5,7‐dihydroxy‐3,6‐dimethoxyflavone (**6**), oroxilin A (**7**), 3,5‐dimethoxy‐6,7‐methylenedioxyflavone (**10**), and 3,5,6,7‐tetramethoxyflavone (**17**) presented an ED_50_: 27.5 µg/mL against KB cell [100]. The compounds alternanthin (**35**) and alternanthin B (**36**) were evaluated against HeLa cells at concentrations of 10 and 30 µg/mL, showing inhibition percentages between 8.9% and 55.9% [36]. Likewise, the mixture of flavonoids (**6**, **7**, **10**, and **17**) was evaluated in vivo, resulting in an increase in the survival of the mice and a reduction in the size of the tumor [100].

Furthermore, compounds quercetin (**24**), quercetin 3‐methyl ether (3‐methoxy quercetin) (**25**), acacetin 8‐C‐[α‐l‐rhamnopyranosyl‐(1 → 2)‐β‐d‐glucopyranoside (**34**), 2″‐*O*‐β‐d‐glucopyranosyl‐vitexin (**43**), isorhamnetin 3‐*O*‐α‐l‐rhamnosyl‐(1 → 6)‐β‐d‐glucopyranoside (**50**), 2″‐*O*‐rhamnopyranosyl‐vitexin (**72**), and quercetin 3‐*O*‐α‐l‐rhamnosyl‐(1 → 6)‐β‐d‐glucopyranoside (**129**) were evaluated as immunomodulators, and it was evidenced that at 50 µmol/L, they do not induce significant release of LDH and are not cytotoxic against PMNL [145].

For some compounds, the effect on the nervous system has been evaluated. Compounds demethyltorosaflavone D (**5**), alternanthin (**35**), alternanthin B (**36**), chrysoeriol 7‐*O*‐rhamnoside or chrysoeriol 7‐rhamnoside (**41**), and torosaflavone E (**79**) were evaluated as antidepressants and antidementia agents. In the first case, the inhibition of MAO‐A and MAO‐B enzymes was evaluated, whereas in the second, the reduction in β‐amyloid (Aβ) aggregation was assessed. The authors report that the compounds were able to inhibit MAO‐A and MAO‐B, as well as reduce the formation of the Aβ‐aggregation, highlighting that compound **36** presents the greatest inhibition of toxic Aβ plaques [38, 135]. These results show that the phenolic compounds isolated from this family also have promising activity in the nervous system.

Additionally, the analgesic and anti‐inflammatory activity in vivo was evaluated for compounds 2″‐*O*‐β‐d‐glucopyranosyl‐vitexin (**43**) and 2″‐*O*‐rhamnopyranosyl‐vitexin (**72**). In both cases, the compounds inhibited hyperalgesia, edema formation, and reduced leukocyte migration [34, 67]. Likewise, compounds patuletin 3‐*O*‐β‐d‐glucopyranoside (**67**) and tiliroside (**106**) were evaluated for anti‐inflammatory activity in vivo, demonstrating a reduction in the edema formation and leukocyte migration [127]. These results show that phenolic compounds have potential as analgesic compounds.

Hypoglycemic activity was also evaluated in cleomiscosin A (**162**) and dictyoceratin C (**166**), but their activity was low [84].

Besides the above, it should be noted that some phenolic compounds such as caffeic acid (**132**) and ferulic acid (**137**), which have been isolated from members of this subfamily, can become candidates for drugs with dual activity (anti‐inflammatory and antimicrobial activity), which would provide an advantage from the pharmacoeconomic point of view [71].

A total of 36 out of 173 phenolic compounds identified in this subfamily have been researched in terms of biological activity, showing promising effects such as antimicrobial, antiviral, antioxidant, anticancer, immunomodulatory, antidepressant, antidementia, analgesic, and anti‐inflammatory. Compounds **43** and **72** are particularly noteworthy, as they have demonstrated antimicrobial, immunomodulatory, anti‐inflammatory, and analgesic activity, highlighting their biotechnological potential.

#### 4.1.1 Flavonoids and Their Glycosides

Flavonoids are phenolic compounds commonly found in nature, known for their diverse biological activities. They exhibit antioxidant, analgesic, anti‐inflammatory, anticancer, antipyretic, antiallergic, antidiabetes, antiulcer, antimicrobial, antiprotozoal, antiplatelet, antiatherogenic, antiestrogenic, cardioprotective, neuroprotective, hepatoprotective, and radioprotective activities [28, 77, 85, 90, 187, 188, 189, 190].

Currently, 133 flavonoids (Table 2) have been reported in members of Gomphrenoideae (Table 2 and Figure 1), consisting of 57 flavone glycosides (**34–90**), 43 flavonol glycosides (**91–133**), 15 flavones (**3–17**), 10 flavonols (**18–27**), 5 iso‐flavones (**28–32**), 2 flavan‐3‐ols (**1–2**), and an aurone (**33**). It should be noted that quercetin (**24**), kaempferol (**20**), and rutin (**130**) are the most commonly isolated flavonoids from these plants.

Some important aspects of the discovery of flavonoids are described below. In 1992, Pomilio reported the occurrence of an isorhamnetin glycoside (**54**) for the first time in *G. boliviana* Moq. [75]. Later, in 2003, Oliveira first reported the isolation of the symmetrically glycosylated methylene bioflavonoid 8,8‴‐methylene bis(spinacetin 3‐*O*‐robinobioside) (**118**) from the ethanol extract of *Blutaparon portulacoides* (A.St.‐Hil.) Mears leaves. In 2004, Ferreira discovered a new hepta‐substituted (*E*)‐aurone glucoside (**33**) in the ethanol extract of *Gomphrena agrestis* Mart. [2]. In 2011, Valentová isolated a new isoflavone (**32**) from the aerial parts of *Iresine diffusa* f. *herbstii* (Hook.) Pedersen, characterized by the presence of a methoxy group in position two of the isoflavanone skeleton [111].

In 2011, Ferreres reported a tetrahydroxymethylenedioxyflavone derivative (**77**) for the first time *in natura* [94]. In 2014, Felipe reported the presence of flavonoids (**103, 106, 126**) in the inflorescences of *Pfaffia glomerata* (Spreng.) Pedersen for the first time [154]. In 2017, Deladino reported the isolation of pentosyl‐vitexin (**70**) and pentosyl‐isovitexin (**69**) in *A. brasiliana* and *Alternanthera tenella* (Synm. *Alternanthera sessilis* (L.) R.Br. ex DC. and *Alternanthera pungens* Kunth) for the first time [28].

Quercetin (**24**) has been reported in various species, including *Alternanthera bettzickiana* (Regel) G. Nicholson, *A. brasiliana*, *Alternanthera maritima* (Mart.) A.St.‐Hil. (Synm. *Alternanthera littoralis* Beauv. ex Moq.), *A. paronychioide*s, *Alternanthera philoxeroides* (Mart.) Griseb., *A. sessilis* (L.) R.Br. ex DC., *A. tenella* Moq. (Synm. *A. sessilis* (L.) DC. and *Alternanthera ficoidea* (L.) P. Beauv.), *G. agrestis*, *Gomphrena celosioides* Mart., *G. claussenii*, *Gomphrena globosa*, and *Iresine angustifolia* Euphrasén. Quercetin is one of the most abundant flavonoids in the nature and is known for its therapeutic application in allergies, cancer, inflammation, obesity, arthritis, asthma, diabetes, prostate adenocarcinoma, immunity, and infections, as well as its gastroprotective and analgesic properties [189].

On the other hand, it is noteworthy that *G. globosa*, with 50 flavonoids (**13, 19–20, 24, 27, 46–48, 50–52, 55, 71, 77, 83–87, 92–95, 97–105, 107–110, 112–114, 116, 120–126, 128, 130, 132**), is the plant with the highest number of flavonoid‐type compounds reported to date.

#### 4.1.2 Non‐Flavonoid Phenolic Compounds

Currently, only 40 non‐flavonoid phenolic compounds have been reported. These include 14 hydroxycinnamic acids (**143–156**), 9 benzoic acids (**134–142**), 2 derivatives of gallic acid (**157–158**), 2 lignans (**160–161**), 2 phenylpropanoids (**163–164**), a coumarin (**159**), a coumarinolignoid (**162**), a glycosylated phenylpropionate (**165**), a sesquiterpene phenol (**166**), and 7 other phenolic compounds (**167–173**) (Table 2 and Figure 1).

*G. celosioides* has the highest number of non‐flavonoid phenolic constituents, with 12 compounds (**140**, **143–144**, **149**, **156**, **160–162**, **166**, **168**, **170–171**). Ferulic acid (**149**) is the most abundant non‐flavonoid phenolic compound distributed among the species of Gomphrenoideae, having been reported in *A. brasiliana*, *A. paronychioides*, *A. philoxeroides*, *A. sessilis*, *A. tenella*, *B. portulacoides*, *G. celosioides* Mart, *G. globosa*, and *I. angustifolia*.

### 4.2 Terpenoids

Among the terpenes are found monoterpenes, triterpenes, sesquiterpenes, and triterpenoid saponins. These compounds exhibit different biological activities, including antioxidant, antimicrobial, antimalarial, anti‐HIV, anti‐inflammatory, antitumor, antimutagenic, anticancer, antipruritic, antidiabetic, anti‐atherosclerotic, antiallergic, cytotoxic, hemolytic, hypotensive, hepatoprotective, immunomodulatory, and nutraceutical activities, as well as inhibition of cardio‐cerebral vascular diseases, playing an important role in the pharmaceutical industry [4, 9, 77, 131, 191].

To date, the production of 95 different terpenes in this subfamily has been reported (Table 3 and Figure 1), including 31 triterpenoid saponins (**234–264**), 31 triterpenes (**203–233**), 19 sesquiterpenes (**179–197**), 5 diterpenoids (**198–202**), 4 monoterpenes (**174–177**), 3 carotenoids (**265–267**), a glycoside monoterpene (**178**), and a drimene (**268**).

Phytol (**202**), oleanolic acid (**221**), pfaffic acid (**226**), and pfaffosides A–F (**255**–**260**) are the most abundant terpenoid compounds distributed among the species of Gomphrenoideae.

In 1984, Nishimoto was the first to isolate the nortriterpene glucuronides called pfaffosides A–C (**255–257**) from the roots of *Pfaffia paniculata* (Mart.) Kuntze (Synm. *Hebanthe eriantha* (Poir.) Pedersen) [125]. That same year, Nakai reported the isolation of three additional nortriterpenoids, pfaffosides D–F (**258–260**), from the roots of *P. paniculata* [164]. In 2005, Rios identified three new drimenes (**268, 195, 196**) from the acetone extract of aerial parts of *I. diffusa* Humb. & Bonpl. ex Willd. [105]. In 2006, Kuroda reported for the first time the isolation of a taraxane glycoside (**245**) from a natural source, the roots of *G. macrocephala* [99].

In 2008, Chaudhary reported the presence of drimanes in the Amaranthaceae family for the first time, with the isolation of 11,12‐acetonide of 11,12,13‐trihydroxydrimene (**179**) and 11,12,13‐trihydroxydrimene (**194**) from the acetone extract of the aerial parts of *T. oblongifolia* (S. Watson) Standl. (Synm. *T. suffruticosa* var. oblongifolia (S. Watson) Sánch.Pino & Flores Olv.). Notably, this remains the only phytochemical study conducted on any *Tidestromia* species [128]. In 2010, 26 years after Nishimoto's discovery, Li reported the isolation of two new nortriterpenoids, pfaffine A and B (**227–228**), from the root of *P. paniculata* [123]. In 2018, Han reported the isolation of three new norolean‐type triterpenes (**215, 216, 224**) from the root of *P. glomerata* [115].

With 24 compounds (**212–213, 215–216, 221, 223–226, 229–230, 234–235, 237–238, 248, 253–260**), *P. glomerata* has the highest number of terpenoid compounds. It is worth noting that in 2014, Felipe was the first to report the presence of glomeric acid (**212**), oleanonic acid (**223**), pfameric acid (**229**), chikusetsusaponin IV (**237**), and ginsenoside R_0_ (**248**) in this plant [154].

As mentioned, terpenes exhibit various pharmaceutical activities, which has led to research on compounds isolated from this subfamily. However, their antimicrobial activity (antiparasitic, antibacterial, and antifungal activity) was not extensively studied. To date, only pfaffic acid (**226**) has been evaluated against *T. cruzi*, showing an IC_50_ of 44.78 ± 7.83 µg/mL [177].

Antiviral activity has been assessed solely for chikusetsusaponin IVa (**238**) against 11 viruses, demonstrating efficacy against enveloped viruses (Table S1), and this compound was also evaluated in vivo in mice infected with HSV‐2, revealing a reduction in viral titer, symptom alleviation, and increased survival (Table 10) [41].

The cytotoxicity of pfaffoside A (**255**), pfaffoside C (**257**), pfaffoside D (**258**), pfaffoside E (**259**), and pfaffoside F (**260**) was evaluated against B‐16 cells, with inhibitory concentrations between 30 and 120 µg/mL as seen in Table S2 [125, 164]. Philoxeroideside A (**261**), philoxeroideside B (**262**), philoxeroideside C (**263**), and philoxeroideside D (**264**) were evaluated against HL60 and SK‐N‐SH cell lines, with IC_50_ values ranging from 37.29 to 271.45 µg/mL. Compound **264** exhibited the highest activity, with IC_50_ values of 45.93 and 37.29 µg/mL, respectively [37].

Additionally, compounds 11α,12α‐epoxy‐3β‐[(*O*‐β‐d‐galactopyranosyl‐(1 → 3)‐*O*‐[β‐d‐glucopyranosyl‐(1 → 2)]‐β‐d‐glucuronopyranosyl)‐oxy]olean‐28,13‐olide **(243)**, 11α,‐12α‐epoxy‐3β‐[(*O*‐β‐d‐glucuronopyranosyl)oxy]olean‐28,13‐olide (**244)**, 11α,‐12α‐epoxy‐3β‐[(*O*‐β‐d‐glucuronopyranosyl)oxy]taraxer‐14‐en‐28‐oic acid β‐d‐glucopyranosyl ester (**245**), 11α,12α‐epoxy‐3β‐hydroxyolean‐28,13‐olide (**246**), and 11α,12α‐epoxy‐3β‐hydroxytaraxer‐14‐en‐28‐oic acid (**247**) were evaluated against HSC‐2, but only compounds **246** and **247** showed activity, with IC_50_ values of 20 µM [99]. These results confirm the potential of most terpenoid‐type compounds as anticancer agents.

Hypoglycemic activity was evaluated in ilimaquinone (**187**) and neodactyloquinone (**190**), showing different levels of activity, with the compound **187** showing the highest efficacy [84].

Pfaffianol A (**225**), boussingoside A_2_ (**235**), pfaffiaglycosides B (**254**), and pfaffoside C (**257**) were evaluated for their effects on melanogenesis inhibition. The results indicated that only compounds **225** and **257** had a significant effect, even greater than that of arbutin [161], as shown in Table 9.

The studies described above provide evidence of antiparasitic, antiviral, and cytotoxic activity against carcinogenic cell lines, as well as hypoglycemic activity and antimelanosis properties in 21 out of the 95 compounds identified in this family. This suggests that the members of this subfamily serve as a reservoir of terpenoid compounds with pharmacological activity.

### 4.3 Alkaloids

Alkaloids possess a wide range of biological activities, including inhibition of malignant cell growth and proliferation, as well as antioxidant, anti‐inflammatory, antiviral, antibacterial, and immunomodulatory effects [77, 84, 151].

To date, 62 alkaloids have been isolated (Table 4 and Figure 1), including 48 betalains (**276–323**), 2 guanidine alkaloids (**269–270**), 2 indole alkaloids (**271–272**), 2 tricyclic alkaloids (**274–275**), a pyridine alkaloid (**273**), and other alkaloids (**324–330**). Among these, the betacyanins amaranthine (**276**), isoamaranthine (**288**), betanin (**295**), and isobetanin (**297**) are the most abundant alkaloids found in species of Gomphrenoideae, being reported all in *A. bettzickiana*, *A. brasiliana*, *A. ficoidea* Griseb. (Synm. *A. littoralis* Beauv. ex Moq.), *A. tenella*, *G. globosa*, *I. herbstii*, and *Iresine lindenii* Van Houtte.

*G. globosa* has the highest number of alkaloid compounds, with 33 compounds (**276, 278–280, 288–290, 294–313, 316, 318–321, 323**).

In 2020, Killian reported the isolation of two new, unusual guanidine alkaloids (**269–270**) from the ethanol extract of aerial parts of *I. diffusa* [165], being the first time that this type of compound was isolated from a Gomphrenoideae species.

On the other hand, aurantiamide (**326**) has been found to possess antioxidant, anti‐inflammatory, antiviral, antibacterial, and immunomodulating properties [84]. This compound has been reported in the extract of the whole plant *G. celosioides* [83–85, 89]. The presence of betalains in this subfamily is noteworthy, as these compounds are exclusively produced by plants belonging to the order Caryophyllales and have demonstrated significant biological activity.

Of the 62 identified alkaloids, 16 have been studied for their biological activity. For instance, the antimicrobial activity of compound aurantiamide acetate (**327**) was evaluated against 19 bacteria and 3 yeasts, showing activity against only 5 bacteria, as detailed in Table S1 [2]. In 2018, Spórna‐Kucab evaluated extracts, seven fractions (mixtures of alkaloids), and individual alkaloid‐type compounds for antimicrobial activity (bacteria and yeast). The study demonstrated that the isolated compounds exhibited better activity than the fractions, whereas the fractions showed better activity than the extracts. The compounds evaluated included *cis*‐isomer of gomphrenin II (**298**), *cis*‐isomer of gomphrenin III (**299**), *cis*‐isomer of isogomphrenin II (**300**), *cis*‐isomer of isogomphrenin III (**301**), gomphrenin II (**304**), gomphrenin III (**305**), isogomphrenin II (**309**), isogomphrenin III (**310**), isosinapoyl‐gomphrenin I (**311**), and sinapoyl‐isogomphrenin I (**313**) [173]. These findings are intriguing, as they suggest that the combination of these compounds may produce an antagonistic effect on antibacterial activity, which is not always the case. In many instances, fractions and extracts exhibit greater activity than isolated compounds due to compound synergism. This research highlights the importance of studying extracts, fractions, and isolated compounds in the search for substances of biotechnological interest.

Additionally, antiparasitic and antioxidant activities were evaluated for compounds alternamide A (7,8‐dihydroxy‐1,2,4,5‐tetrahydro‐3*H*‐1,5‐ethano[c]azepin‐3‐one) (**274**), alternamide B (6,7‐dihydroxy‐3,4‐dihydroquinoline‐1‐one) (**275**), alternamine A ((*R*)‐1‐(3,4‐dihydroxyphenyl)‐1,2,3,4‐tetrahydroisoquinoline‐6,7‐diol) (**324**), and alternamine B (4‐(2‐aminoethyl)benzene‐1,2‐diol‐4‐(2‐aminoethyl)benzene‐1,2‐diol‐b‐d‐glucopyranose) (**325**). These compounds showed different degrees of activity, as detailed in Tables S1 and S2, with compound **324** exhibiting promising antiparasitic activity [8].

Regarding anticancer activity, only compounds celosiadine A (**269**) and celosiadine B (**270**) have been evaluated in vitro, showing activity against LNCaP but not PC3 cells. Hypoglycemic activity was evaluated in bruceolline F (**271**), but it showed no activity [84].

The studies evaluating the bioactivity of alkaloid compounds from this subfamily have confirmed their antimicrobial, antioxidant, anticancer, and hypoglycemic properties, demonstrating the potential of this subfamily as a source of biologically active compounds. However, there is still a vast area for investigation, as there have been limited studies to ascertain the biotechnological potential of the betalains identified in this subfamily.

#### 4.3.1 Betalains

Betalains are classified into betacyanins (red‐violet) and betaxanthins (yellow) based on their structure. In plants, they serve protective functions against pathogens and various environmental conditions, aiding in plant propagation. Once isolated, betalains can be utilized as colorants and exhibit a wide range of biological activities, including antioxidants, anticancer, radioprotective, antilipidemic, antihypoglycemic, anti‐inflammatory, and antimicrobial (antibacterial, antifungal, and antiviral) effects. They are also used in the treatment of hypertension, diabetes, anemia, thalassemia, high cholesterol levels, calcium deficiency disorders, and liver‐related issues. Additionally, betalains demonstrate chemopreventive properties and have positive effects on metabolism, cardiovascular health, and gastrointestinal health in humans. Importantly, betalains are non‐toxic and do not exhibit mutagenic or allergic reactions [29, 96, 130, 174].

To date, 48 types of betalains have been reported in this subfamily. These include 18 belonging to the amaranthin group (**276–293**), 16 to the gomphrenin group (**298–313**), 8 types of betaxanthine (**316–323**), 4 belonging to the betanin group (**294–297**), and 2 other betalains (**314–315**). Deladino's 2017 study indicates that it was the first report of amaranthine (**276**), isoamaranthine (**288**), betanin (**295**), and isobetanin (**297**) in *Alternanthera* species, although these compounds are commonly isolated from *Amaranthus* species [28]. However, it is important to note that these compounds were previously reported by Cai in *A. bettzickiana* and *A. ficoidea*, making Cai's study the first to report these compounds [166].

As mentioned above, *G. globosa* showed the broadest phytochemical profile in terms of betalains. It is also worth noting that 22 betalains (**276, 277, 281–282, 284–289, 291–293, 295, 297, 304–305, 309–312, 314**) have been reported in *I. herbstii* and 17 (**276, 278, 281–282, 284–285, 288–289, 291–293, 295, 297, 305, 310, 314–315**) have been reported in *I. lindenii*. Among the compounds isolated in this subfamily, Betanin (**295**) is known to combat oxidative stress and reduce tumors in the lung, skin, colon, liver, and esophageal in various animal models. It also shows activity against tumors in the prostate, breast, and pancreas in humans and inhibits the proliferation of various human cancer cell lines [130]. Additionally, gomphrenin (**302**) has been found to have chemopreventive activity, whereas celosianin (**278**) and iresin (**283**) exhibit antioxidant potential [130].

Spórna‐Kucab in 2020 proposed new tribal names for some betalains. Among the proposed changes was renaming gomphrenina II (**304**) to globosin, gomphrenina III (**305**) to basellin (due to its presence in *Basella alba*), 2″‐OE‐sinapoyl‐amaranthin (**289**) to lindenin (because of its presence in *I. lindenii*), sinapoyl‐gomphrenin I (**312**) to gandolin (due to its presence in *Gandola nigra*), celosianin I (**277**) to argentianin, and celosianin II (**278**) to celosianin [110].

### 4.4 Compounds of Lipid Nature

Currently, 35 compounds have been reported in members of this subfamily. These include 33 fatty acids (**331–363**), a fatty alcohol (**364**), and a fatty amide (**365**) (Table 5 and Figure 1). *A. brasiliana* has the largest spectrum of lipid compounds (**335, 337–348, 353–363, 365**).

Regarding the evaluation of biological activity, only the antimicrobial and antimelanosis activities have been evaluated as described below. Compounds (8*E*)‐10‐hydroxy‐8‐octadecenoic acid (**337**), (10*E*)‐9‐hydroxy‐10‐octadecenoic (**338**), (8*E*,12*Z*)‐10‐hydroxy 8,12‐octadecadienoic acid (**339**), (9*Z*,11*E*)‐13‐hydroxy‐9,11‐octadecadienoic acid (**340**), (9*Z*,11*E*,15*Z*)‐13‐hydroxy‐9,11,15‐octadecatrienoic acid (**341**), (9*Z*,12*Z*,14*E*)‐16‐hydroxy‐9,12,14‐octadecatrienoic acid (**342**), (9*Z*,13*E*)‐12‐hydroxy‐9,13‐octadecadienoic acid (**343**), (9*Z*,13*E*,15*Z*)‐12‐hydroxy‐9,13,15‐octadecatrienoic acid (**344**), (10*E*,12*E*)‐9‐hydroxy‐10,12‐octadecadienoic acid (**345**), (10*E*,12*Z*)‐9‐hydroxy‐10,12‐octadecadienoic acid (**346**), (9*Z*)‐9‐octadecenedioic acid (**354**), (7*E*)‐9‐oxo‐7‐octadecenoic acid (**356**), (8*E*)‐10‐oxo‐8‐octadecenoic acid (**357**), (9*E*,11*E*)‐13‐oxo‐9,11‐octadecadienoic acid (**358**), (9*Z*,11*E*)‐13‐oxo‐9,11‐octadecadienoic acid (**359**), (10*E*,12*E*)‐9‐oxo‐10,12‐octadecadienoic acid (**360**), and (10*E*,12*Z*)‐9‐oxo‐10,12‐octadecadienoic (**361**) were evaluated against three bacteria, but only nine of them showed activity as detailed in Table S1 [32].

Of the 35 compounds identified, only 17 have been evaluated for biological activity, highlighting a significant research opportunity.

### 4.5 Other Compounds

A total of 146 other compounds were reported, which consisted of 26 phytosterols (**386–412**), 22 aliphatic hydrocarbons (**419–440**), 20 phytoecdysteroids (**368–385**), 7 carboxylic acids (**447–443**), 6 alkanes (**441–446**), 4 saponins (**414–417**), 4 feruloyl tyramine derivatives (**454–457**), 4 tocopherols (**464–467**), 3 hydrocarbons (**460–462**), 2 phytoecdysones (**366–367**), 2 vitamins (**468–469**), 2 heterocyclic compounds (**458–459**), 1 phytosteroid (**413**), 1 aliphatic alcohol (**418**), 1 organic acid (**463**), and 43 compounds that were not classified (**470–512**) (Table 6 and Figure 1). With 30 compounds, *Gomphrena elegans M*art. had the highest number of secondary metabolites of another type (**421–427, 429–434, 436–437, 439–440, 461–462, 475–476, 479–480, 488, 493, 495–498, 508, 511**), highlighting that 77.27% of aliphatic hydrocarbons were reported in *G. elegans M*art. Additionally, it can be seen from Table 6 that feruloyl tyramine‐type compounds were only reported in *A. philoxeroides*.

In 1998, Sarker was the first to report the phytoecdysteroid 2‐dehydro‐3‐epi‐20‐hydroxyecdysone (**370**) in seeds of *Froelichia floridana* (Nutt.) Moq. [192]. Another important aspect was that Roriz, in 2014, reported tocopherols (**464, 466, 467**) for the first time in *G. globosa* [97].

The biological activity of these compounds has been studied little. Compounds Δ^7^‐stigmasterol (**411**) and 3‐*O*‐β‐d‐glucopyranosyl Δ^7^‐stigmasterol (**416**), and the following mixtures **411** and campesterol (**386**); spinasterol (**396**) and **411**; 3‐*O*‐β‐d‐glucopyranosyl stigmasterol (**415**) and **416**; sitosterol glycoside (**394**) and 3‐*O*‐β‐d‐glucopyranosyl spinasterol (**414**), were evaluated against bacteria, yeast, and fungi, showing activity to varying degrees, as detailed in Table S1 [142]. Likewise, the mixture of compounds sitosteryl (**395**) and stigmasteryl 3‐β‐*O*‐glucoside 6′‐*O*‐palmitate (**412**) was evaluated for antimicrobial activity against 15 microorganisms but showed activity against 6 (Table S1) [151]. Compound stigmast‐6‐en‐3‐*O*‐β‐(d‐glicopyranoside) (**417**) was evaluated against five microorganisms but only had activity against two of them (Table S1) [157]. On the other hand, the mixture of compounds **395** and **412** was evaluated against *T. cruzi* and *L. amazonensis* and showed activity (Table S1) [151]. Compound (**449**) was tested against six bacteria and showed activity against all of them, although the activity was lower than that of the extracts tested (Table S1) [82].

Additionally, compounds β‐ecdysone (**367**), 22‐oxo‐20‐hydroxyecdysone (**377**), pterosterone (**381**), taxisterone (**384**), and 2β,3β,14α,17β‐tetrahydroxy‐5β‐androst‐7‐en‐6‐one (**385**) were evaluated as melanogenesis inhibitors, but none showed activity. Notably, terpenoid compounds were also assessed as melanogenesis inhibitors and demonstrated significant activity (see Table 9) [161]. Compounds (**455**), (**456**), and (**457**) were evaluated against HeLa cells, and two of them showed significant effects, as evidenced in Table S2 [36].

## 5 Pharmacological Activities

To date, 162 articles have evaluated biological activity, both in vitro (108 articles) and in vivo (73 articles), of 30 species within the Gomphrenoideae subfamily. This research shows that only 6.10% of the members of this subfamily have been studied in terms of biological activity. Furthermore, of the 15 genera, only 6 have been studied. This indicates that the remaining genera, *Froelichia*, *Froelichiella*, *Hebanthodes*, *Pedersenia*, *Pseudoplantago*, *Quaternella*, *Tidestromia*, and *Xerosiphon*, remain unexplored in terms of biological activity.

On the other hand, it should be noted that the pharmacological investigations of different extracts and isolated compounds from members of this subfamily confirm various biological activities. These include antioxidant, antimicrobial, anticancer, anti‐inflammatory, antidiabetic, antihyperglycemic, antiarthritic, antihypertensive, analgesic, immunomodulatory, neuroprotective, cardioprotective, gastroprotective, hepatoprotective, diuretic, and wound‐healing properties.

It is important to emphasize that when consolidating and analyzing the biological activity data of the isolated compounds, fractions, extracts, and extract‐based nanoparticles, a notable variability becomes evident. In some cases, isolated compounds exhibit greater activity compared to extracts and fractions, whereas in others, the opposite occurs. This variability can be attributed to synergistic and antagonistic interactions between the compounds, as well as the specific concentrations of each compound present in the extract, which can influence the overall activity.

In this sense, the importance of conducting studies focused on examining extracts, fractions, isolated compounds, and compound mixtures is evident, as this could lead to the discovery of phytotherapeutics that are beneficial for the pharmaceutical industry and the community. Additionally, it is crucial to conduct studies that evaluate the potential of free extracts and drug delivery systems, such as nanoparticles, because the latter have shown that they can enhance the biological activity of the extract in many cases.

Another important observation, which is discussed in the following sections, is that extracts from the same species exhibited different results for the same biological activity. This variability can be attributed to the fact that the study plants were sourced from different geographical locations and times, resulting in distinct phytochemical profiles and consequently varying biological activities. These findings underscore the importance of conducting studies that consider different varieties of the same species, plants collected at different times and locations, and various environmental and stress conditions, as well as the use of in vitro systems and greenhouses to assess the stability of the phytochemical profile and biological activity. Such studies can help determine the optimal conditions for producing bioactive substances of interest.

The significance of the studies that lead to the discovery of new phytotherapeutic compounds lies in their lower cost, greater availability, reduced adverse effects, biodegradability, and environmental safety compared to synthetic drugs [14, 16, 129, 192].

**SCHEME 4:**
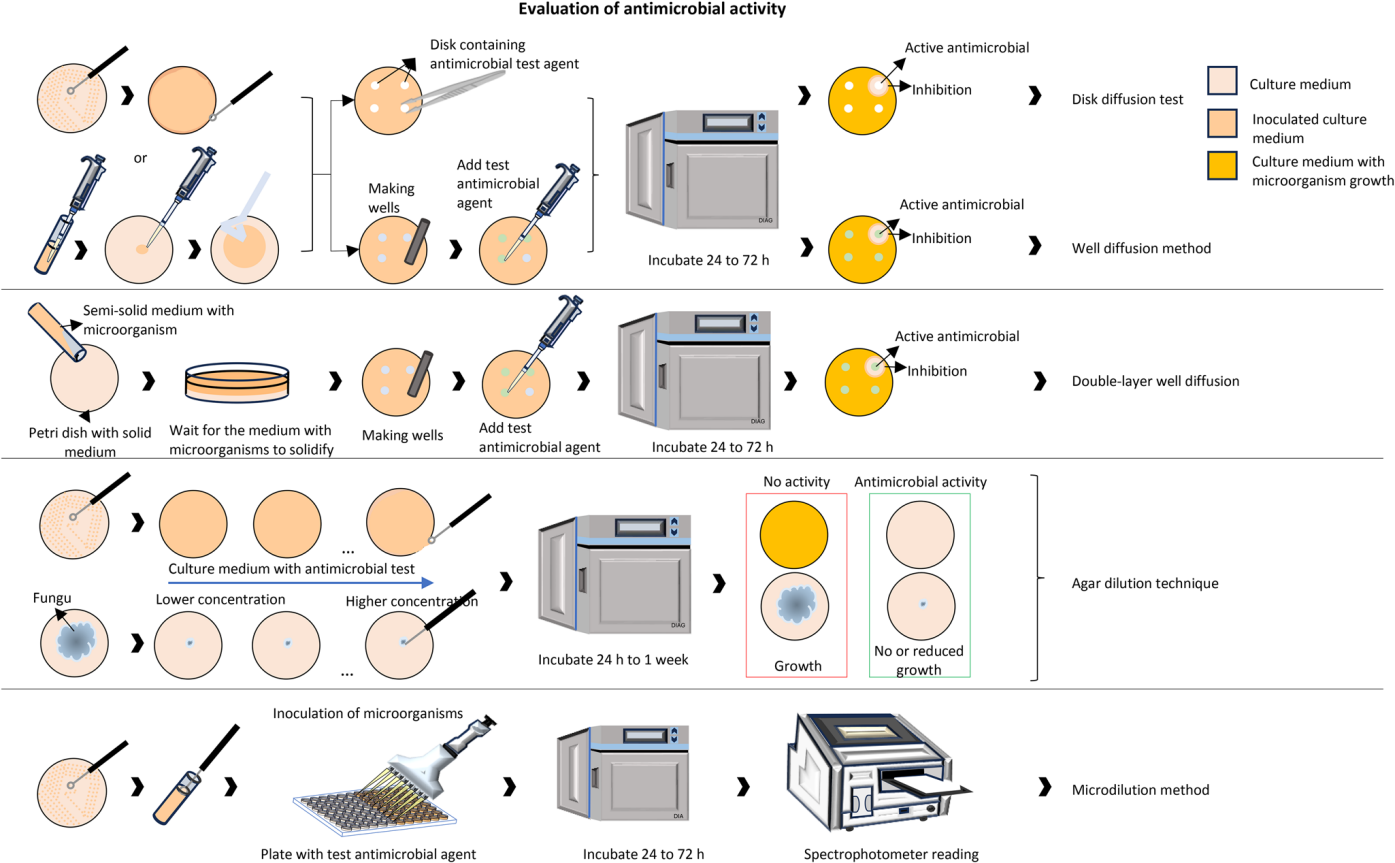
Antimicrobial activity assays. The tests presented are used to evaluate bacteria, yeasts, and fungi. All tests are standardized in terms of culture medium, temperature, and incubation period, according to the evaluated microorganism. Each assay includes a negative control, positive control (bacteria: ampicillin, amoxicillin, bacitracin, chloramphenicol, ceftriaxone sodium, clarithromycin, ciprofloxacin, erythromycin, gentamicin, isoniazid, kanamycin, norfloxacin, streptomycin, streptomycin sulfate, and tetracycline; yeast: amphotericin B, chloramphenicol, ketoconazole, nystatin, and tioconazole; fungi: fluconazole, ketoconazole, tioconazole), and different concentrations of the tested agent (0.5–12 500 µg/mL).

**SCHEME 5:**
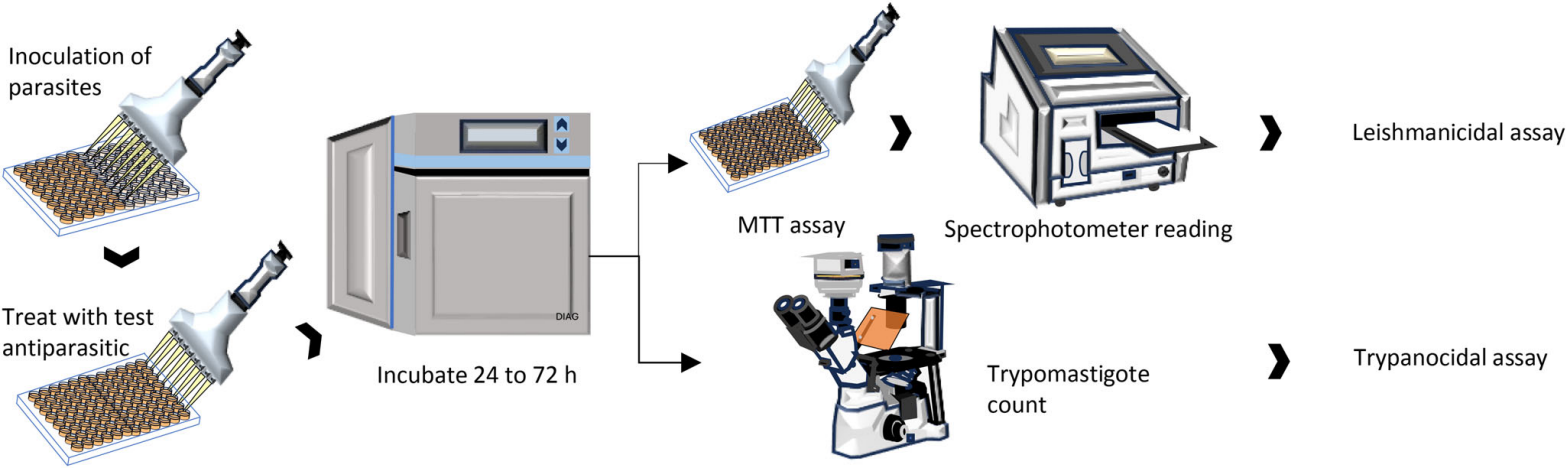
Antiparasitic activity assays. The tests presented are used to evaluate the antiparasitic potential. They are standardized in terms of culture medium, temperature, and incubation period according to the evaluated organism. The assay includes a negative control, a positive control (*Trypanosoma cruzi*: crystal violet and gentian violet; *Leishmania amazonensis*: amphotericin B), and different concentrations of the agent (1–4000 µg/mL).

**SCHEME 6:**
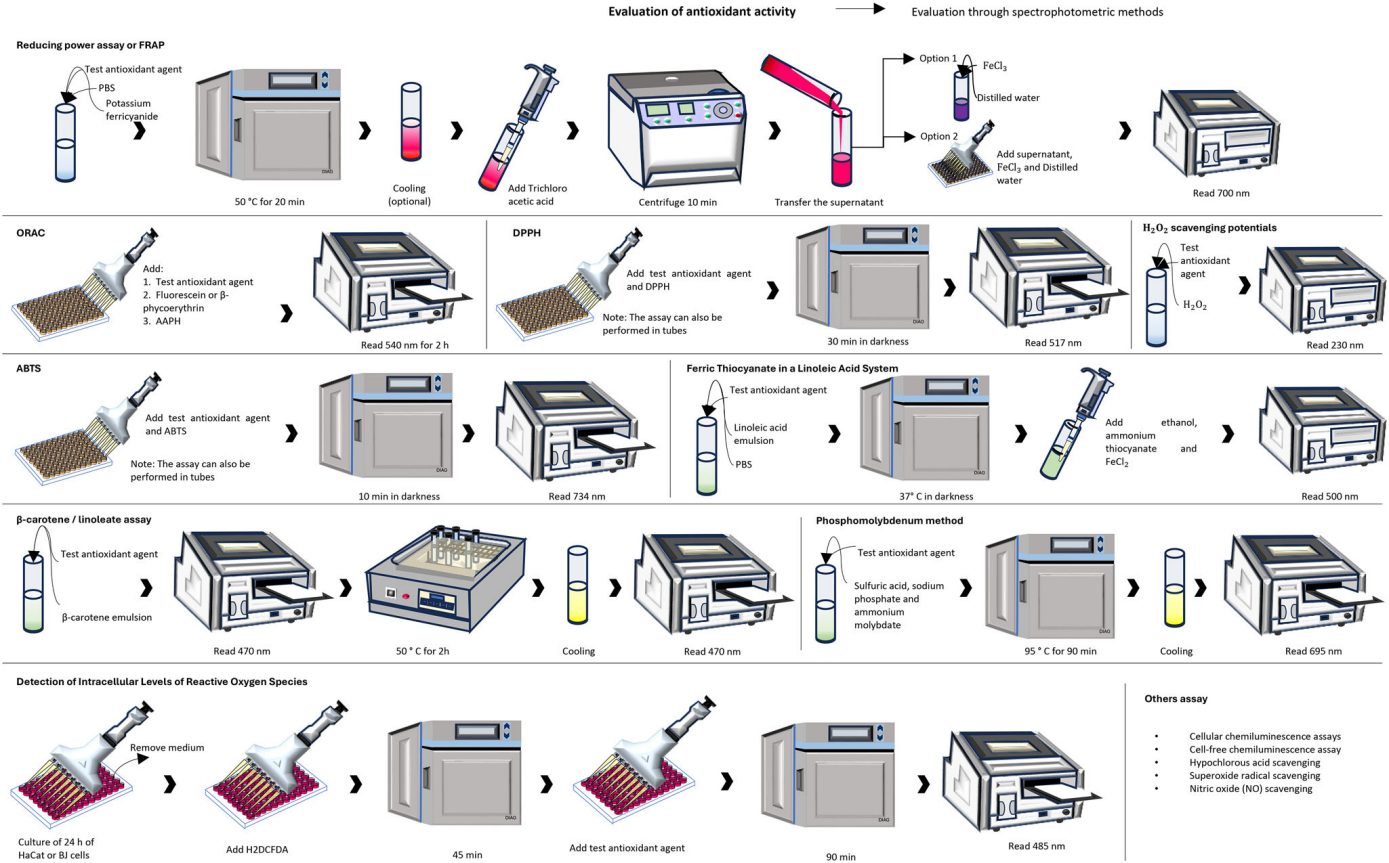
Antioxidant activity assays. All tests are standardized and include negative control, positive control, and different concentrations of the test agent.

An overview of the modern pharmacological studies conducted on different extracts and isolated compounds is described in the following subsections, Tables 7, 8, 9, 10, and Schemes 4, 5, 6, 7. In general, the reported studies used standardized and/or similar techniques and concentrations, allowing comparisons between the reported results, thus generating a more comprehensive analysis.

**TABLE 7:** Anti‐inflammatory activity of the Gomphrenoideae subfamily.

Species	Extract(s)/Compounds	Assay method	Model	Dose	Positive control	Activity		References
						Values	Analysis	
*Alternanthera brasiliana*	HaE of leaves	ELISA IL‐6	RAW 264.7 cells	10.0, 50.0, and 100.0 µg/mL	Gallic acid and quercetin	↓ Production of IL‐6	↓ The production of IL‐6 and TNF‐α in a dose‐dependent manner Significantly ↓ free radical (NO and O2^•−^) The HaE of leaves exhibited anti‐inflammatory activity	[193]
		ELISA TNF‐α	RAW 264.7 cells	10.0, 50.0, and 100.0 µg/mL	Gallic acid and quercetin	↓ Production of TNF‐α		
		NO releasing	RAW 264.7 cells	10.0, 50.0, and 100.0 µg/mL	Gallic acid and quercetin	IC_50_: 79.2 ± 5.7 µg/mL		
		Inhibition of O_2_^•−^ production	RAW 264.7 cells	10.0, 50.0, and 100.0 µg/mL	Gallic acid and quercetin	IC_50_: 72.1 ± 6.0 µg/mL		
*Alternanthera sessilis*	EE of stems	Cell viability (MTT method)	RAW 264.7 cells	25, 50, 100, 200, 300, 400, and 500 µg/mL	—	500 µg/mL ↓ cell viability by 80% ↓ NO levels in a dose‐dependent manner Significantly suppresses the production of PGE_2_, IL‐6, IL‐1β, and TNF‐α Inhibit the translocation of the NF‐κB subunit p65 to the nucleus ↓ The expression of iNOS and COX‐2 Prevents phosphorylation of IκBα and, consequently, activation of NF‐κB p65		[56]
		NO releasing	RAW 264.7 cells	50, 100, and 200 µg/mL	Dexamethasone			
		ELISA	RAW 264.7 cells	50, 100, and 200 µg/mL	Dexamethasone			
		Immunocytochemistry	RAW 264.7 cells	50, 100, and 200 µg/mL	—			
		Protein expression analysis	RAW 264.7 cells	50, 100, and 200 µg/mL	BSA			
*Gomphrena celosioides*	Extract	NO releasing	RAW 264.7 cells	10 mg/mL	—	%*I*: 31.1	Anti‐inflammatory effect	[195]
		Determination of COX‐2	RAW 264.7 cells	Uninformed	Celecoxib	%*I*: 59.4		
*Gomphrena celosioides*	367	NO determination	RAW 264.7 cells	Uninformed	L‐NMMA	NDNS	512 showed the greatest activity in inhibiting nitric oxide activity	[178]
	512					IC_50_: 19.55 ± 0.61 µM		
	20‐Hydroxyecdysone‐20,22‐monoacetonide					IC_50_: 97.35 ± 1.14 µM		
*Gomphrena globosa*	AqE of inflorescences	Cell viability (MTT and LDH assays)	RAW 264.7 cells	Uninformed	Dexamethasone	NDNS	Extracts do not significantly affect cell viability All the extracts decrease nitric oxide levels, observing the highest activity in commercial preparation	[98]
		NO determination				NDNS		
	EB of inflorescences	Cell viability (MTT and LDH assays)	RAW 264.7 cells	Uninformed	Dexamethasone	NDNS		
		NO determination				NDNS		
	Commercial preparation of inflorescences	Cell viability (MTT and LDH assays)	RAW 264.7 cells	Uninformed	Dexamethasone	NDNS		
		NO determination				NDNS		
*Gomphrena globosa* var. *albiflora* (white amaranth)	HE of flowers	NO determination	RAW 264.7 cells	Uninformed	Dexamethasone	EC_50_ (µg/mL): 198 ± 5	The HE from flowers of the three *Gomphrena* species exhibited dose‐dependent activity and has potential as an anti‐inflammatory agent	[73]
*Gomphrena haageana* K. (red amaranth)	HE of flowers	NO determination	RAW 264.7 cells	Uninformed	Dexamethasone	EC_50_ (µg/mL): 136 ± 4		
*Gomphrena* sp. (pink globe amaranth)	HE of flowers	NO determination	RAW 264.7 cells	Uninformed	Dexamethasone	EC_50_ (µg/mL): 133 ± 7		
*Pfaffia glomerata*	FD of roots	NO determination	BMDM (bone marrow‐derived macrophage)	250, 25, 2.5, and 0.25 µg/mL	—	NDNS	The highest concentrations evaluated (250 and 25 µg/mL) of the two fractions ↓ NO production. Only the FD of roots at a concentration of 0.25 µg/mL was able to ↓ the NO production of cells stimulated with LPS	[162]
	FD of aerial part					NDNS		
								

Abbreviations: EE, ethanolic extracts; ELISA, enzyme‐linked immunosorbent assay; FD, fraction of DCM; HaE, hydroalcoholic extract; HE, hydromethanolic extracts; IL‐6, interleukin‐6; LDH, lactate deshydrogenase; NDNS, numerical data not shown; TNF‐α, tumor necrosis factor alpha.

**TABLE 8:** Antidiabetic and antihyperglycemic activity of the Gomphrenoideae subfamily.

Species	Extract(s)/Compounds	Assay method	Model	Dose	Positive control	Activity		References
*Alternanthera paronychioides*	ME	CAA assay	HepG2	Uninformed	—	EE presented the highest antioxidant activity (175.8 ± 1.7 µM QE/g) EE did not show a cytotoxic effect, on the contrary, inhibited 90% of the cytotoxicity induced by HG in HIT‐T15 cells EE inhibits: HG‐induced ROS production, cell accumulation in sub‐G1, cell apoptosis by up to 14%, activation and activity of caspases 3 and 9, cleavage of PARP EE ↑ ΔΨm and attenuates the ↑ in Bax/ Bcl‐2 ratio EE ← PDX1 translocation in RIN‐m5F cells and ↑ the level of insulin secretion		[35]
	EE	CAA assay	HepG2	Uninformed	—			
		MTT assay	HIT‐T15	10–100 µg/mL	Ferulic acid and quercetin			
			RIN‐m5F					
		Staining with PI and DAPI	HIT‐T15	20 and 50 µg/mL	Quercetin			
		ΔΨm analysis	HIT‐T15	50 µg/mL	Quercetin			
		DCFH‐DA	HIT‐T15	20 and 50 µg/mL	Quercetin			
			RIN‐m5F				
		Western blot analysis	HIT‐T15	20 and 50 µg/mL	Quercetin			
		ELISA	RIN‐m5F	20 and 50 µg/mL	Quercetin			
	AqE	CAA assay	HepG2	Uninformed	—			
*Alternanthera philoxeroides*	Fraction X of ME of leaves	α‐Glucosidase inhibition assay	—	20, 40, and 60 µg/mL	Luteolin	%*I*_60 µg/mL_: 58.2 and IC_50_: 52.41 ± 5.22		[141]
*Alternanthera sessilis*	Green leaf juice	Prevention of Hb glycation	—	200 µL	—	NE	Potent α‐glucosidase inhibitor	[195]
		α‐Amylase inhibition assay	Pancreatic α‑amylase	—	—	NE		
		α‐Glucosidase inhibition assay	Rat intestinal α‑glucosidase	20 µL	—	IC_50_: 0.22 ± 0.0 mg/mL acarbose equivalent		
*Alternanthera sessilis*	FH of ME of leaves	α‐Glucosidase inhibition assay	—	Uninformed	Acarbose	EC_50_: 6.31 ± 1.70 mg/mL	Callus fractions exhibited higher antiglucosidase activity than leaf fractions The FEA of leaves, and FH, CF, and FEA of callus showed an EC_50_ lower than acarbose. In contrast, the AF of leaves, and BuF and AF of callus exhibited glucosidase‐stimulating activity The FEA of leaves and callus have been identified As noncompetitive and competitive α‐glucosidase inhibitors, respectively	[196]
	CF of ME of leaves	α‐Glucosidase inhibition assay	—	Uninformed	Acarbose	EC_50_: 4.89 ± 1.67 mg/mL		
	FEA of ME of leaves	α‐Glucosidase inhibition assay	—	Uninformed	Acarbose	EC_50_: 0.55 mg/mL		
		Lineweaver–Burk plot analysis	—	0, 550, and 825 µg/mL		*K_m_*: 2.406 ± 0.070 and *V*_max_: 0.018 ± 0.001 for 550 µg/mL		
	BuF of ME of leaves	α‐Glucosidase inhibition assay	—	Uninformed	Acarbose	EC_50_: 2.95 ± 0.31 mg/mL		
	AF of ME of leaves	α‐Glucosidase inhibition assay	—	Uninformed	Acarbose	EC_50_: Nd		
	FH of ME of callus	α‐Glucosidase inhibition assay	—	Uninformed	Acarbose	EC_50_: 0.67 ± 0.05 mg/mL		
	CF of ME of callus	α‐Glucosidase inhibition assay	—	Uninformed	Acarbose	EC_50_: 0.90 ± 0.11 mg/mL		
	FEA of ME of callus	α‐Glucosidase inhibition assay	—	Uninformed	Acarbose	EC_50_: 0.25 ± 0.01 mg/mL		
		Lineweaver–Burk plot analysis	—	0, 250, and 375 µg/mL		*K_m_*: 3.706 ± 0.283 and *V*_max_: 0.063 ± 0.009 for 375 µg/mL		
	BuF of ME of callus	α‐Glucosidase inhibition assay	—	Uninformed	Acarbose	EC_50_: Nd		
	AF of ME of callus	α‐Glucosidase inhibition assay	—	Uninformed	Acarbose	EC_50_: Nd		
*Gomphrena celosioides*	271	PTP1B inhibition assay	—	50 mM	Ursolic acid	40.21% ± 1.69%		[84]
		Measuring glucose uptake	3T3‐L1 adipocytes	20 µM	Insulin	% 2‐NBDG absorbance: ≅125		
	162	PTP1B inhibition assay	—	50 mM	Ursolic acid	17.89% ± 2.76%		
		Measuring glucose uptake	3T3‐L1 adipocytes	20 µM	Insulin	% 2‐NBDG absorbance: ≅118		
	166	PTP1B inhibition assay	—	50 mM	Ursolic acid	8.10% ± 5.19%		
		Measuring glucose uptake	3T3‐L1 adipocytes	20 µM	Insulin	% 2‐NBDG absorbance: 100		
	187	PTP1B inhibition assay	—	50 mM	Ursolic acid	28.64% ± 8.01%		
		Measuring glucose uptake	3T3‐L1 adipocytes	20 µM	Insulin	% 2‐NBDG absorbance: ≅122		
	190	PTP1B inhibition assay	—	50 mM	Ursolic acid	80.39% ± 6.88%		
		Measuring glucose uptake	3T3‐L1 adipocytes	20 µM	Insulin	% 2‐NBDG absorbance: ≅138		

Abbreviations: AF, aqueous fraction; CAA, cellular antioxidant activity; CF, chloroform fraction; DCFH‐DA, 6‐carboxy‐2′,7′‐dichlorodihydrofluorescein diacetate; EE, ethanolic extracts; ELISA, enzyme‐linked immunosorbent assay; FEA, fraction of ethyl acetate; FH, fraction of hexane; ME, methanolic extracts.

**TABLE 9:** Other activities of the Gomphrenoideae subfamily.

Species	Extract(s)/Compounds	Assay method	Model	Dose	Positive control	Activity			References
						Values	Analysis		
**Activity in blood cells**									
*Alternanthera bettzickiana*	AqE of leaves	Emmel test	Sickle cell blood	0, 5, 10, 15, and 20 µg/mL	Parahydroxybenzoic acid	% of normalization: 86 for 11 µg/mL	It has an antisickling effect since the RBCs maintain a circular (biconcave) and normal shape		[18]
*Pfaffia paniculata*	Commercial capsule powder	Ektacytometric studies	RBCs	0–0.5 mg/mL		↑ The deformity index indicates improvement in cellular hydration, which is independent of density. Also ↑ the mean corpuscular volume, Na^+^, and ↓ the mean cell hemoglobin []			[239]
*Pfaffia paniculata*	Extract of commercial powder	Hematological and RBC deformability measurement	RBCs	0.2 or 0.5 mg/mL		0.5 mg/mL ↑ the deformity of falciform cells Had no effect on the RBC's aggregation properties			[240]
		RBC aggregation	RBCs	0.2 or 0.5 mg/mL					
**Activity related to the urinary system**									
*Gomphrena celosioides*	367	Xanthine oxidase inhibitory activity	—	Uninformed	Allopurinol	IC_50_: 81.04 ± 0.49 µM	The inhibitory action of xanthine oxidase was most evident in compound **512**		[183]
	512			Uninformed	Allopurinol	IC_50_: 33.78 ± 0.49 µM			
	20‐Hydroxyecdysone‐20,22‐monoacetonide			Uninformed	Allopurinol	IC_50_: 101.15 ± 0.48 µM			
**Anti‐aging activities**									
*Gomphrena globosa*	HaE of flowers	Determination of anti‐elastase activity	Neutrophil elastase	100 and 250 µg/mL	SPCK	NDNS	Elastase and collagenase inhibitory activity were [] dependent HaE has significant anti‐collagenase properties		[156]
		Determination of anti‐collagenase activity	Collagenase	100 and 250 µg/mL	1,10‐Phenanthroline	%*I*_250 µg/mL_ about 40%			
**Anti‐allergy activity**									
*Alternanthera sessilis*	EE of aerial parts	Measurement of LDH release	RBL‐2H3 cells	25, 50, and 100 µg/mL	—	NE	↓ The release of β‐hexosaminidase in a []‐dependent manner ↓ Intracellular Ca^2+^ levels Suppresses the release of IL‐6, TNF‐α, IL‐4, and IL‐13 ↓ Degradation of IkBα and nuclear translocation of p65 NF‐kB Suppresses antigen‐induced degranulation of RBL‐2H3 cell		[241]
		β‐Hexosaminidase secretion assay	RBL‐2H3 cells sensitized with DNP‐specific IgE	25, 50, and 100 µg/mL	—	NDNS			
		Ca^2+^ measurement	RBL‐2H3 cells sensitized with DNP‐specific IgE	25, 50, and 100 µg/mL	—	NDNS			
		Measurement of IL‐6, TNF‐α, IL‐13, and IL‐4 (ELISA=	RBL‐2H3 cells sensitized with DNP‐specific IgE	25, 50, and 100 µg/mL	—	NDNS			
		Western Blot Analysis	RBL‐2H3 cells sensitized with DNP‐specific IgE	25, 50, and 100 µg/mL	—	NDNS			
**Antiarthritic activity**									
*Alternanthera bettzickiana*	EE of aerial parts	Egg albumin denaturation inhibition	—	50, 100, 200, 400, 800, 1600, 3200, and 6400 µg/mL	Diclofenac sodium	%*I*_6400 µg/mL_: 94.23%	Dose‐dependent activity The results of the EE were better than those of the standard drug		[19]
		Protein denaturation using BSA				%*I*_6400 µg/mL_: 97.43 ± 0.70			
**Anticoagulant activity**									
*Alternanthera philoxeroides*	ME of whole plant	PTT test		250, 500, and 1000 µg/mL	—	To 1000 µg/mL: 13.26	Showed significant anticoagulant activity of ME According to PT, vanillic acid exhibited the highest anticoagulant activity, whereas in aPTT, tannic acid had the highest activity		[138]
		aPTT tests			—	To 1000 µg/mL: 66.28			
	Tannic acid	PTT test		10 µg/mL	—	12.62			
		aPTT tests		10 µg/mL	—	57.54			
	Gallic acid	PTT test		10 µg/mL	—	13.33			
		aPTT tests		10 µg/mL	—	56.26			
	Catechin	PTT test		10 µg/mL	—	11.71			
		aPTT tests		10 µg/mL	—	56.87			
	Vanillic acid	PTT test		10 µg/mL	—	15.91			
		aPTT tests		10 µg/mL	—	56.14			
**Antidepressant activity**									
*Alternanthera philoxeroides*	EE of whole plant	Estrogenic activity (cell‐based assay)	MCF‐7	1–100 µg/mL	17β‐Estradiol	EqE_100_ = 1.68 µg/mL	1.68 µg/mL is equally effective as 100 pM of 17β‐estradiol		[38]
**Anti‐diarrhea and/or anti‐dysentery activity**									
*Alternanthera repens*	AqE of leaves	[]–response curves to CaCl_2_	Ileum of rats	0.56, 1.09, or 2.09 mg/mL	—	IC_50_: 0.25 ± 0.03 mM at a [] of 0.56 mg/mL	AqE, ME, and F2 to F4 have a spasmolytic effect on the CaCl_2_‐induced contractions, with ME showing the highest activity ME, AqE, and F2 to F4 inhibit the stimulatory effect of KCl; therefore, their mechanism of action is CCB All extracts and F2 to F4 inhibited 5‐HT‐induced ileum contractions, presenting a spasmolytic effect		[47]
		Relaxant effect on K^+^‐C	Ileum of rats	0.56, 1.09, or 2.09 mg/mL	—	IC_50_: 0.82 ± 0.01 mM at a [] of 0.56 mg/mL			
		Inhibition of dose–response curves to 5‐HT	Ileum of rats	0.56, 1.09, or 2.09 mg/mL	—	IC_50_: 7.19 ± 0.04 × 10^−8^ M at a [] of 0.56 mg/mL			
		[]–response curves to ACh	Ileum of rats	2.09 mg/mL	—	%*I*: 58.6 IC_50_: 72.68 ± 0.08 × 10^−6^ M			
	HeE of leaves	[]–response curves to CaCl_2_	Ileum of rats	0.24, 0.47, or 0.91 mg/mL	—	NE			
		Relaxant effect on K^+^‐C	Ileum of rats	0.24, 0.47, or 0.91 mg/mL	—	NE			
		Inhibition of dose–response curves to 5‐HT	Ileum of rats	0.24, 0.47, or 0.91 mg/mL	—	IC_50_: 5.44 ± 0.08 × 10^−6^ M at a [] of 0.24 mg/mL			
	ME of leaves	[]–response curves to CaCl_2_	Ileum of rats	0.24, 0.47, or 0.91 mg/mL	—	IC_50_: 0.18 ± 0.061 mM at a [] of 0.24 mg/mL			
		Relaxant effect on K^+^‐C	Ileum of rats	0.24, 0.47, or 0.91 mg/mL	—	IC_50_: 0.043 ± 0.001 mM at a [] of 0.24 mg/mL			
		Inhibition of dose–response curves to 5‐HT	Ileum of rats	0.24, 0.47, or 0.91 mg/mL	—	IC_50_: 2.24 ± 0.06 × 10^−7^ M at a [] of 0.24 mg/mL			
	Six DF of ME	[]–response curves to CaCl_2_	Ileum of rats	0.66 mg/mL	—	NDNS			
		Relaxant effect on K^+^‐C	Ileum of rats	0.66 mg/mL	—	NDNS			
		Inhibition of dose–response curves to 5‐HT	Ileum of rats	0.24, 0.47, or 0.91 mg/mL	—	NDNS			
*Alternanthera sessilis*	EE of whole plant	Relaxant and/or contractile	Jejunum of rabbit	Uninformed	Verapamil	EC_50_: 0.26 mg/mL SRC EC_50_: 0.08 mg/mL K^+^‐SC	EE and DF relax SRC and Kþ‐SC, but AF only has a spasmolytic effect on CRS EE acts as CCB and generates the repolarization of the ΔΨm DF showed greater CCB activity than FA		[64]
		Evaluation of CCB	Jejunum of rabbit	0.1–0.3 mg/mL	Verapamil				
	DF of EE	Relaxant and/or contractile	Jejunum of rabbit	Uninformed	Verapamil	EC_50_: 0.02 mg/mL SRC EC_50_: 0.04 mg/mL K^+^‐SC			
		Evaluation of CCB	Jejunum of rabbit	Uninformed	Verapamil				
	AF of EE	Relaxant and/or contractile	Jejunum of rabbit	Uninformed	Verapamil	EC_50_: 0.36 mg/mL SRC EC_50_: NE			
		Evaluation of CCB	Jejunum of rabbit	Uninformed	Verapamil				
**Antihypertensive effect**									
*Alternanthera sessilis*	EE of whole plant	Vasorelaxant activity	Aorta of rabbit constricted by PE or K^+^	Uninformed	Verapamil	EC_50_ (mg/mL): 2.03 and 0.34 for PE and K^+^ induced contraction, respectively	DF exhibited the highest vasorelaxant effect EE and DF reinforce the presence of the CCB mechanism		[64]
	DF of EE	Vasorelaxant activity	Aorta of rabbit constricted by PE or K^+^	Uninformed	Verapamil	EC_50_ (mg/mL): 0.32 and 0.15 for PE and K^+^ induced contraction, respectively			
	AF of EE	Vasorelaxant activity	Aorta of rabbit constricted by PE or K^+^	Uninformed	Verapamil	NE			
**Antiparasitic activity**									
						P (min for 10 mg/mL)	D (min for 10 mg/mL)		
*Gomphrena celosioides*	EaE of whole plant	Anthelmintic assay (P and D)	*F. gigantica*	10, 20, 30, 40, 50, 70, 80, and 100 mg/mL	Piperazine citrate	20 ± 0.8	35 ± 0.5	ME was more potent in causing paralysis of *P. posthuma* than EaE and the positive control	[82]
			*P. posthuma*			40 ± 0.2	>60		
			*T. solium*			15 ± 0.4	40 ± 0.3		
	ME of whole plant		*F. gigantica*	10, 20, 30, 40, 50, 70, 80, and 100 mg/mL	Piperazine citrate	40 ± 0.5	45 ± 0.1		
			*P. posthuma*			8 ± 0.9	60 ± 0.5		
			*T. solium*			37 ± 0.3	42 ± 0.3		
**Antitumor activity**									
*Alternanthera pungens*	AqE of whole plant	Potato disc method	*A. tumefaciens*	10, 100, 1000, and 10 000 ppm	Vincristine	IC_50_ (ppm): 1800		Dose‐dependent activity	[16]
	ME of whole plant	Potato disc method	*A. tumefaciens*	10, 100, 1000, and 10 000 ppm	Vincristine	IC_50_ (ppm): 11			
	*n*HE of whole plant	Potato disc method	*A. tumefaciens*	10, 100, 1000, and 10 000 ppm	Vincristine	IC_50_ (ppm): 90			
**Cardioprotective activity**									
*Alternanthera philoxeroides*	ME of leaves	MTT assay	Rat cardiac H9c2 cells with DOX‐induced apoptosis	10, 20, 40, 80, and 160 mg/mL	—	%*V*_10 mg/mL_: 38.43 ± 11.5 %*V*_20 mg/mL_: 66.33 ± 6.03 %*V*_40 mg/mL_: 79.00 ± 3.6 %*V*_80 mg/mL_: 84.33 ± 5.5 %*V*_160 mg/mL_: 83.16 ± 8.12		↑ Cell viability, indicating a protective effect against DOX‐mediated cytotoxicity ↓ The cell apoptosis induced by DOX	[42]
		Annexin V‐FITC/PI staining assay	Rat cardiac H9c2 cells with DOX‐induced apoptosis	10, 20, 40, 80, and 160 mg/mL	—	%*A*_10 mg/mL_: 51.18 %*A*_20 mg/mL_: 42.5 %*A*_40 mg/mL_: 33.18 %*A*_80 mg/mL_: 25.2 %*A*_160 mg/mL_: 23.46			
**Endothelial activation**									
*Alternanthera sessilis*	EE of whole plant	MTT assay	HAECs cells	25, 50, 100, 200, 400, and 800 µg/mL	—	6.25–200 µg/mL of EE does not affect the viability of HAECs 200 µg/mL of EE ↓ the ↑ permeability stimulated by TNF‐α EE does not alter the secretion of sVCAM‐1 induced by TNF‐α 200 µg/mL of EE suppresses the release of intracellular ROS stimulated by TNF‐α EE does not ↓ the elevated production of H_2_O_2_ induced by TNF‐α EE ↑ SOD activity in a dose‐dependent manner EE enhances CAT activity in cells exposed to by H_2_O_2_			[153]
		FITC‐dextran permeability assay	HAECs cells	25–200 µg/mL	Simvastatin				
		sVCAM‐1 production assay	HAECs cells	25, 50, 100, and 200 µg/mL	NAC				
		ROS quantitative assay	HAECs cells	25, 50, 100, and 200 µg/mL	—				
		H_2_O_2_	HAECs cells	25, 50, 100, and 200 µg/mL	Simvastatin				
		SOD activity	HAECs cells	25, 50, 100, and 200 µg/mL	Simvastatin, NAC, or dexamethasone				
		CAT activity	HAECs cells	25, 50, 100, and 200 µg/mL	Simvastatin				
**Hemolytic activity**									
*Pfaffia glomerata*	FD of roots	Hemolysis assay	Blood from 6‐ to 8‐week‐old male C57BL/6 mice	250, 25, 2.5, and 0.25 µg/mL	Saponin from *Quillaja* sp.	NDNS	The two FDs did not cause hemolytic effects		[167]
	FD of aerial part								
**Inhibition of melanogenesis**									
*Pfaffia glomerata*	225	Melanogenesis assay	Murine B16 melanoma 4A5 cells RCB0557	0, 1, 3, 10, 30, and 100 µM	Arbutin	%*I*_100 µM_: 80.0 ± 2.5 IC_50_: 44 µM	Pfaffianol A (**225**) and pfaffoside C (**257**) substantially inhibited melanogenesis without cytotoxic effects, showing stronger effects than arbutin The other compounds showed no effect		[166]
		Viability assay							
	254	Melanogenesis assay	Murine B16 melanoma 4A5 cells RCB0557	0, 1, 3, 10, 30, and 100 µM	Arbutin	%*I*_10 µM_: −1.2 ± 1.6			
	235	Melanogenesis assay	Murine B16 melanoma 4A5 cells RCB0557	0, 1, 3, 10, 30, and 100 µM	Arbutin	%*I*_100 µM_: 22.1 ± 2.4			
	367	Melanogenesis assay	Murine B16 melanoma 4A5 cells RCB0557	0, 1, 3, 10, 30, and 100 µM	Arbutin	%*I*_100 µM_: −6.1 ± 7.6			
	384	Melanogenesis assay	Murine B16 melanoma 4A5 cells RCB0557	0, 1, 3, 10, 30, and 100 µM	Arbutin	%*I*_30 µM_: 1.9 ± 3.5			
	381	Melanogenesis assay	Murine B16 melanoma 4A5 cells RCB0557	0, 1, 3, 10, 30, and 100 µM	Arbutin	%*I*_100 µM_: −3.3 ± 3.0			
	377	Melanogenesis assay	Murine B16 melanoma 4A5 cells RCB0557	0, 1, 3, 10, 30, and 100 µM	Arbutin	%*I*_30 µM_: 7.4 ± 2.2			
	385	Melanogenesis assay	Murine B16 melanoma 4A5 cells RCB0557	0, 1, 3, 10, 30, and 100 µM	Arbutin	%*I*_100 µM_: 2.3 ± 4.2			
	257	Melanogenesis assay	Murine B16 melanoma 4A5 cells RCB0557	0, 1, 3, 10, 30, and 100 µM	Arbutin	%*I*_100 µM_: 51.4 ± 1.2 IC_50_: 92 µM			
**Immunomodulatory activity**									
*Alternanthera brasiliana*	AqE of leaves	Proliferation assay	PBMN stimulated with PHA	0–200 µg/mL	—	Aqueous and ethanol extracts inhibit the proliferative response of lymphocytes to PHA 10 µg/mL of FEA completely inhibits lymphocyte proliferation			[242]
	EE of leaves	Proliferation assay	PBMN stimulated with PHA	0–200 µg/mL	—				
*Alternanthera maritima*	AqE of leaves	Proliferation assay	PBMN stimulated with PHA	0–200 µg/mL	—				
	EE of leaves	Proliferation assay	PBMN stimulated with PHA	0–200 µg/mL	—				
*Alternanthera tenella*	AqE of leaves	Proliferation assay	PBMN stimulated with PHA	0–200 µg/mL	—				
	EE of leaves	Proliferation assay	PBMN stimulated with PHA	0–200 µg/mL	—				
	FEA of AqE of leaves	Proliferation assay	PBMN stimulated with PHA	0–200 µg/mL	—				
*Alternanthera maritima*	EE of aerial parts	Cytotoxicity and LDH assay	PMNLs	100 µg/mL	Total cell lysis	%*V*: 78.50 ± 1.50 and LA _(IU*1000)_: 1.39 *ð* 0.07	EE, BuF, and the seven isolated compounds do not induce significant LDH release, nor do they exhibit cytotoxicity against human PMNL		[149]
	BuF of EE of aerial parts	Cytotoxicity and LDH assay	PMNLs	100 µg/mL	Total cell lysis	%*V*: 82.00 ± 1.00 and LA _(IU*1000)_: 0.76 ± 0.17			
	25	Cytotoxicity and LDH assay	PMNLs	50 µmol/L	Total cell lysis	%*V*: 97.75 ± 0.35 and LA _(IU*1000)_: 5.22 ± 0.27			
	24	Cytotoxicity and LDH assay	PMNLs	50 µmol/L	Total cell lysis	%*V*: 91.25 ± 1.77 and LA _(IU*1000)_: 6.29 ± 3.20			
	129	Cytotoxicity and LDH assay	PMNLs	50 µmol/L	Total cell lysis	%*V*: 94.50 ± 0.71 and LA _(IU*1000)_: 5.63 ± 1.01			
	50	Cytotoxicity and LDH assay	PMNLs	50 µmol/L	Total cell lysis	%*V*: 87.00 ± 2.83 and LA _(IU*1000)_: 4.13 ± 1.46			
	43	Cytotoxicity and LDH assay	PMNLs	50 µmol/L	Total cell lysis	%*V*: 91.50 ± 3.54 and LA _(IU*1000)_: 4.08 ± 1.93			
	72	Cytotoxicity and LDH assay	PMNLs	50 µmol/L	Total cell lysis	%*V*: 87.50 ± 0.71 and LA _(IU*1000)_: 5.81 ± 1.64			
	34	Cytotoxicity and LDH assay	PMNLs	50 µmol/L	Total cell lysis	%*V*: 93.50 ± 1.41 and LA _(IU*1000)_: 5.74 ± 2.27			
*Gomphrena celosioides*	Extract	Proliferation assay (MTT)	RAW 264.7	0–100 µg/mL	—	IC_50_: >100	It has no cytotoxic effect against RAW 264.7 cells, nor does it enhance phagocytosis Significantly inhibits TNF‐α production		[213]
		Phagocytosis assay	RAW 264.7	10 µg/mL	—	NDNS			
		Measurement of TNF‐α	RAW 264.7	10 µg/mL	—	%*I*: 32.5			
*Gomphrena virgata*	AqE of roots	Viability assay (trypan blue)	PBMN	1, 0.5, and 0.025 mg/mL	Cadmium chloride	%*V*_1 mg/mL_: 95.86 ± 7.12 (24 h) and %*V*_1 mg/mL_: 69.14 ± 26.69 (7 days)	c.		[101]
**Insecticidal activity**									
*Gomphrena elegans* Mart.	AqE, ClE, HeE, and ME of leaves	Insecticidal activity tests	Larvae of *Aedes aegypti*	10 mg/L	Bt	None of the extracts exhibited insecticidal activity.			[159]
**Larvicidal activity**									
*Alternanthera sessilis*	AgNPs of leaves	Mosquito culture and larvicidal bioassays	*Aedes aegypti*	Uninformed	Silver nitrate	LC_50_: 7.2 (24 h), 4.63 (48 h), and 2.93 (72 h)	For the three larval models, both the AgNPs complex and the different extracts exhibited a dose‐dependent effect HeE stands out as having the greatest larvicidal effect compared to the others		[163]
			*Culex quinquefasciatus*			LC_50_: 9.28 (24 h), 5.43 (48 h), and 2.82 (72 h)			
			*Anopheles stephensi*			LC_50_: 12.92 (24 h), 3.29 (48 h), and 2.9 (72 h)			
	ME of leaves	Mosquito culture and larvicidal bioassays	*Aedes aegypti*	Uninformed	Silver nitrate	LC_50_: 88.75 (24 h), 82.21 (48 h), and 71.74 (72 h)			
			*Culex quinquefasciatus*			LC_50_: 32.32 (24 h), 26.59 (48 h), and 20.63 (72 h)			
			*Anopheles stephensi*			LC_50_: 40.13 (24 h), 23.98 (48 h), and 17.26 (72 h)			
	HeE of leaves	Mosquito culture and larvicidal bioassays	*Aedes aegypti*	Uninformed	Silver Nitrate	LC_50_: 24.41 (24 h), 20.72 (48 h). and 17.03 (72 h)			
			*Culex quinquefasciatus*			LC_50_: 26.25 (24 h), 20.63 (48 h). and 16.03 (72 h)			
			*Anopheles stephensi*			LC_50_: 47.48 (24 h), 39.64 (48 h). and 30.23 (72 h)			
	ClE of leaves	Mosquito culture and larvicidal bioassays	*Aedes aegypti*	Uninformed	Silver nitrate	LC_50_: 31.90 (24 h), 26.24 (48 h), and 18.83 (72 h)			
			*Culex quinquefasciatus*			LC_50_: 49.19 (24 h), 35.22 (48 h), and 22.47 (72 h)			
			*Anopheles stephensi*			LC_50_: 45.97 (24 h), 34.16 (48 h), and 26.28 (72 h)			
	AcE of leaves	Mosquito culture and larvicidal bioassays	*Aedes aegypti*	Uninformed	Silver nitrate	LC_50_: 85.35 (24 h), 81.67 (48 h), and 67.43 (72 h)			
			*Culex quinquefasciatus*			LC_50_: 39.85 (24 h), 28.38 (48 h), and 20.73 (72 h)			
			*Anopheles stephensi*			LC_50_: 29.75 (24 h), 23.98 (48 h), and 19.02 (72 h)			
	PEE of leaves	Mosquito culture and larvicidal bioassays	*Aedes aegypti*	Uninformed	Silver nitrate	LC_50_: 156.87 (24 h), 150.22 (48 h), and 119.38 (72 h)			
			*Culex quinquefasciatus*			LC_50_: 37.79 (24 h), 30.23 (48 h), and 18.83 (72 h)			
			*Anopheles stephensi*			LC_50_: 59.0 (24 h), 49.21 (48 h), and 37.93 (72 h)			
**Neuroprotective or neurological activity**									
*Alternanthera philoxeroides*	EE of whole plant	Inhibitory assay	MAO‐A	Uninformed	Clorgyline	IC_50_: 252.9 ± 0.02 µM	EE and isolated compounds inhibit MAO‐A and MAO‐B EE and isolated compounds, except for compound **35**, show partial selectivity for MAO‐B. Alternatin exhibits partial selectivity by MAO‐A		[38]
			MAO‐B		Deprenyl	IC_50_: 90.69 ± 0.02 µM			
	35	Inhibitory assay	MAO‐A	Uninformed	Clorgyline	IC_50_: 0.00046 ± 0.04 µM			
			MAO‐B		Deprenyl	IC_50_: 0.00060 ± 0.12 µM			
	36	Inhibitory assay	MAO‐A	Uninformed	Clorgyline	IC_50_: 0.00206 ± 0.04 µM			
			MAO‐B		Deprenyl	IC_50_: 0.00022 ± 0.12 µM			
	79	Inhibitory assay	MAO‐A	Uninformed	Clorgyline	IC_50_: 18.37 ± 1.47 µM			
			MAO‐B		Deprenyl	IC_50_: 0.6748 ± 0.46 µM			
	5	Inhibitory assay	MAO‐A	Uninformed	Clorgyline	IC_50_: 0.0541 ± 0.01 µM			
			MAO‐B		Deprenyl	IC_50_: 0.1293 ± 0.42 µM			
	41	Inhibitory assay	MAO‐A	Uninformed	Clorgyline	IC_50_: 3.051 ± 0.35 µM			
			MAO‐B		Deprenyl	IC_50_: 0.5441 ± 0.33 µM			
*Alternanthera philoxeroides*	EE of whole plant	Thioflavin‐T assay	Aβ1‐42 solution	100 µg/mL	Cur	%*I*: 83.25 ± 4.25	Among the isolated compounds, compound **36** exhibits the greatest inhibitory activity against the formation of toxic Aβ plaques in the brain, surpassing that of Cur		[141]
	35	Thioflavin‐T assay	Aβ1‐42 solution	100 µM	Cur	NDNS			
	36	Thioflavin‐T assay	Aβ1‐42 solution	100 µM	Cur	%*I*: 81.96 ± 2.14			
	41	Thioflavin‐T assay	Aβ1‐42 solution	100 µM	Cur	NDNS			
	79	Thioflavin‐T assay	Aβ1‐42 solution	100 µM	Cur	NDNS			
	5	Thioflavin‐T assay	Aβ1‐42 solution	100 µM	Cur	NDNS			
*Alternanthera philoxeroides*	EE of whole plant	Ellman's method	AChE	Uninformed	Tacrine	IC_50_:2.06 ± 0.016 mg/mL	The SIs of the EE was 1.60, indicating its partial selectivity toward AChE		[141]
			BChE			IC_50_: 3.27 ± 0.011 mg/mL			
*Gomphrena globosa* L.	AqE or EB or commercial preparation of inflorescences	Ellman's method	AChE	Uninformed	—	NE	None of the extracts is capable of inhibiting AChE		[98]
*Iresine celosia*	EE of aerial part	WST‐1 assay	Mouse BV2 microglial cells stimulated with/without LPS	1, 10, or 100 µg/mL	Quercetin	No cytotoxic activity was observed against BV2 cells. 100 µg/mL ↓ NO production and PGE_2_ levels Significantly ↓ MAPK phosphorylation, without affecting total protein levels of MAPK factors 100 µg/mL significantly ↓ transcriptional activity of NF‐κB Significantly suppresses the phosphorylation of p65 and its translocation to the nucleus Can inhibit LPS‐induced neuroinflammation without cytotoxicity in BV2 cells, as well as suppress the expression of proinflammatory mediators mediated by the MAPKs/NF‐κB signaling pathway 30 and 100 µM of Iresin (283) significantly ↓ NO production			[104]
		Measurement of NO and PGE_2_	Mouse BV2 microglial cells stimulated with LPS	1, 10, or 100 µg/mL	Quercetin				
		qRT‐PCR	Mouse BV2 microglial cells	1, 10, or 100 µg/mL					
		Western blotting	Mouse BV2 microglial cells stimulated with LPS	1, 10, or 100 µg/mL					
		Luciferase assay	Mouse BV2 microglial cells stimulated with LPS	100 µg/mL					
	283	Measurement of NO and PGE_2_	Mouse BV2 microglial cells stimulated with LPS	1, 10, 30, and 100 µM	Quercetin				
*Iresine herbstii*	AqE of aerial part	5‐HT_1A_ serotoninergic assay	Cerebral cortex of rats	25, 50, 75, 100, and 125 µg/mL	8‐OH‐DPAT	%*I*: 13.51	AqE and ME show no affinity for 5‐HT_2A_ receptor, and additionally, the ME did not show affinity against the D2 receptor AqE and ME have an effect on the CNS similar to that observed with some psychotropic agents		[108]
		5‐HT_2C_ serotoninergic assay	Frontal cortical regions of rats	25, 50, 75, 100, and 125 µg/mL	Mesulergine	%*I*: 22.13			
		D1 dopaminergic assay	Corpora striata	25, 50, 75, 100, and 125 µg/mL	Spiroperidol	%*I*: 48.32			
		D2 dopaminergic assay	Corpora striata	25, 50, 75, 100, and 125 µg/mL	Spiroperidol	%*I*: 88.82 IC_50_: 32.08 ± 0.52			
		α_1_‐Adrenergic binding assay	Brain cortex	25, 50, 75, 100, and 125 µg/mL	Prazosin	%*I*: 13.51			
	ME of aerial part	5‐HT_1A_ serotoninergic assay	Cerebral cortex of rats	25, 50, 75, 100, and 125 µg/mL	8‐OH‐DPAT	%*I*: 22.44			
		5‐HT_2C_ serotoninergic assay	Frontal cortical regions of rats	25, 50, 75, 100, and 125 µg/mL	Mesulergine	%*I*: 92.46 IC_50_: 34.78 ± 1.80			
		D1 dopaminergic assay	Corpora striata	25, 50, 75, 100, and 125 µg/mL	Spiroperidol	%*I*: 90.52 IC_50_: 19.63 ± 2.10			
		α_1_‐Adrenergic binding assay	Brain cortex	25, 50, 75, 100, and 125 µg/mL	Prazosin	%*I*: 11.76			
*Iresine herbstii*	AqE of aerial part	5‐HT_1A_ serotoninergic assay	Cerebral cortex of rats	7.8–125 µg/mL	—	%*I*_125 µg/mL_: 13.51	AqE and ME have no affinity for the 5‐HT_2A_ receptor. Additionally, ME did not exhibit affinity for the D2 and α_2_ receptors, while AqE showed no affinity for the α_1_ receptor AqE and ME interact with 5‐HT receptors, indicating an effect on the CNS		[243]
		5‐HT_2C_ serotoninergic assay	Frontal cortical regions of rats	7.8–125 µg/mL	—	%*I*_125 µg/mL_: 22.13			
		D1 dopaminergic assay	Corpora striata	7.8–125 µg/mL	—	%*I*_125 µg/mL_: 48.32			
		D2 dopaminergic assay	Corpora striata	7.8–125 µg/mL	—	IC_50_: 2.99 ± 0.02 µg/mL			
		α_2_‐Adrenergic binding assay	Brain cortex	7.8–125 µg/mL	—	%*I*_125 µg/mL_: 25.73			
	ME of aerial part	5‐HT_1A_ serotoninergic assay	Cerebral cortex of rats	7.8–125 µg/mL	—	%*I*_125 µg/mL_: 22.44			
		5‐HT_2C_ serotoninergic assay	Frontal cortical regions of rats	7.8–125 µg/mL	—	IC_50_: 60.27 ± 5.59 µg/mL			
		D1 dopaminergic assay	Corpora striata	7.8–125 µg/mL	—	IC_50_: 1.39 ± 1.70 µg/mL			
		α_1_‐Adrenergic binding assay	Brain cortex	7.8–125 µg/mL	—	%*I*_125 µg/mL_: 11.76			
*Pfaffia glomerata*	ME of roots	Sharma and Bhat method	AChE	10 mg/mL	Galantamine	NE	Only the FD of aerial part showed activity	[167]	
	ME of aerial part	Sharma and Bhat method	AChE	10 mg/mL	Galantamine	NE			
	FnH of roots	Sharma and Bhat method	AChE	10 mg/mL	Galantamine	NE			
	FnH of aerial part	Sharma and Bhat method	AChE	10 mg/mL	Galantamine	NE			
	FD of roots	Sharma and Bhat method	AChE	10 mg/mL	Galantamine	NE			
	FD of aerial part	Sharma and Bhat method	AChE	10 mg/mL	Galantamine	IC_50_: 2.7 µg/mL			
	FEA of roots	Sharma and Bhat method	AChE	10 mg/mL	Galantamine	NE			
	FEA of aerial part	Sharma and Bhat method	AChE	10 mg/mL	Galantamine	NE			
	FnB of roots	Sharma and Bhat method	AChE	10 mg/mL	Galantamine	NE			
	FnB of aerial part	Sharma and Bhat method	AChE	10 mg/mL	Galantamine	NE			
	AF of roots	Sharma and Bhat method	AChE	10 mg/mL	Galantamine	NE			
	AF of aerial part	Sharma and Bhat method	AChE	10 mg/mL	Galantamine	NE			
**Sun protective effect**									
*Gomphrena globosa*	HaE of flowers	Determination of SPF	—	50 µg/mL	—	SPF of about 20			[156]
**Treatment of respiratory diseases**									
*Alternanthera sessilis*	EE of whole plant	Assessment of bronchodilator activity	Tracheal tissue of rabbit induced with CCh or K^+^	Uninformed	Verapamil	EC_50_: 0.22 and 0.18 mg/mL for CCh and K^+^ induced contraction, respectively	EE and DF decrease the maximum contractile effect similar to verapamil through CCB It should be noted that CCBs are beneficial as bronchodilators		[64]
	DF of EE	Assessment of bronchodilator activity	Tracheal tissue of rabbit induced with CCh or K^+^	Uninformed	Verapamil	EC_50_: 0.04 and 0.03 mg/mL for CCh and K^+^ induced contraction, respectively			
	AF of EE	Assessment of bronchodilator activity	Tracheal tissue of rabbit induced with CCh or K^+^	Uninformed	Verapamil	NE			
**Wound‐healing activity**									
*Alternanthera brasiliana*	ME of leaves	CM model	Embryonated chicken eggs	200 and 400 µg	—	ME has dose‐dependent angiogenetic activity, ranging from mild (200 µg) to marked (400 µg)			[244]
*Alternanthera sessilis*	EE of stem	Scratch assay	NHDF cells	12.5, 25, and 50 µg/mL	Allantoin	↑ Migration _50_ _µg/mL_: 86%	Dose‐dependent activity was observed in all cell lines		[57]
		Scratch assay	HDF‐D cells	12.5, 25, and 50 µg/mL	Allantoin	↑ Migration 50 µg/mL: 65%			
		Scratch assay	HaCaT cells	12.5, 25, and 50 µg/mL	Allantoin	↑ Migration _50_ _µg/mL_: 99%			
*Iresine herbstii*	EE of leaves	NF‐κB electrophoretic mobility shift assay	Jurkat T cells ACC No. 282	100 µg/mL	Parthenolide	%*I*: <30	The extracts exhibited moderate migration and filling in the damaged area EE inhibits elastase release, while HeE alters elastase activity EE did not show a cytotoxic effect, while HeE exhibited cytotoxic effect in a Jurkat T cells HeE has moderate caspase activity		[109]
		p38α assay	—	100 µg/mL		%*I*: 30.27 ± 0.67			
		Elastase assay	PAF‐stimulated neutrophils	10, 50, and 100 µg/mL	Resveratrol, GW311616A	Release_100 µg/mL_: 42.62 ± 1.66 Inhibition_100 µg/mL_: 13.02 ± 0.92			
		Scratch assay	Mouse fibroblasts	10 µg/mL	PDGF‐BB	%St: 34.33 ± 2.92			
		MTT assay	Jurkat T cells	50 and 100 µg/mL	Parthenolide	%*I*_100 µg/mL_: 7 ± 0.7			
	HeE of leaves	NF‐κB electrophoretic mobility shift assay	Jurkat T cells ACC No. 282	100 µg/mL	Parthenolide	%*I*: <30			
		p38α assay	—	100 µg/mL		%*I*: 74.14 ± 6.33			
		Elastase assay	PAF‐stimulated neutrophils	10, 50, and 100 µg/mL	Resveratrol, GW311616A	Release_100 µg/mL_: 39.22 ± 0.77 Inhibition_100 µg/mL_: 59.68 ± 0.60			
		Scratch assay	Mouse fibroblasts	10 µg/mL	PDGF‐BB	%St: 28.26 ± 2.41			
		Caspase‐3‐like assay	Jurkat T cells	50 µg/mL	Actinomycin D	Relative fluorescence unit of 1.07			
		MTT assay	Jurkat T cells	50 and 100 µg/mL	Parthenolide	%*I*_50 µg/mL_: 12 ± 1.4 and %*I*_100 µg/mL_: 61 ± 6.4			

Abbreviations: aPTT, activated partial thromboplastin; CCB, calcium channel blocking; ClE, chloroform extract; DF, dichloromethane fraction; EE, ethanolic extracts; FD, fraction of DCM; FEA, fraction of ethyl acetate; HeE, hexanic extracts; IL‐6, interleukin‐6; LDH, lactate deshydrogenase; ME, methanolic extracts; NDNS, numerical data not shown; NE, no effect; PBMN, peripheral blood mononuclear cells; PMNLs, polymorphonuclear leukocytes; PTT, prothrombin time; RBCs, red blood cells; ROS, reactive oxygen species; TNF‐α, tumor necrosis factor alpha.

**TABLE 10:** Pharmacological effects of crude extracts and compounds of Gomphrenoideae subfamily.

Species	Extract (s)/Compounds	Models	Assay method	Dose	Positive control	Effects/Mechanisms	References
**Antimicrobial activity**							
*Gomphrena globosa*	**20**	Male Kunming mice	Murine model of superficial skin infection	30 mg/kg	Ceftriaxone sodium	↓ The number of colonies in the wound Antibacterial efficacy was better than positive control	[136]
**Activity related to the sexual system**							
*Pfaffia glomerata*	HaE of roots	C57BL/6J	Histomorphometric studies	600 and 1000 mg/kg	—	Does not act as an endocrine disruptor and has no antiandrogenic activity	[247]
*Pfaffia glomerata*	HaE of roots	Swiss mice (male)	Histological analysis	100, 200, and 400 mg/kg for 42 days	Sildenafil citrate	400 mg/kg ↑ the weight of the testes and Leydig somatic index 200 mg/kg administered intermittently ↑ the weight of the testes and parenchyma HaE ↑ the proportions of interstitium, Leydig cells, lymphatic vessels, and NO 400 mg/kg of HaE causes DNA damage and cell death in Leydig cells, whereas lower concentrations and sildenafil citrate do not show cytotoxicity 200 and 400 mg/kg ↓ plasma testosterone and ↑ 17β‐estradiol levels At 400 mg/kg, the amount of connective tissue in the penis ↓, whereas type I collagen ↑ ↓ The amount of type III collagen and the % of smooth muscle	[113]
			Volumetric proportions and interstitium analysis				
			Leydig cell morphometry				
			Leydig cell viability				
			Hormone assay				
			Collagen and smooth muscle quantification				
			NO assay				
*Pfaffia glomerata*	HaE of roots	SPF ICR mice	Sexual behavior experiment	150, 750, and 1500 mg/kg for 28 days	Paroxetine	The behavioral study indicated that HaE improved sexual performance in mice HaE helps mitigate the damage caused by paroxetine to the testes and has the potential to enhance sexual function HaE concentrations ↑ the levels of T, FSH, and E2, restore NO and cGMP content, and significantly reduced PDE5 activity, with results comparable to the control group. HaE effectively reduced PRX‐induced MDA elevation Histologically, testicular lesions were recovered by HaE HaE promoted spermatogenesis	[168]
			Organ coefficient				
			Measurements of hormones and enzymes				
			Testis histopathological and sperm analysis				
				
				
*Pfaffia glomerata*	HaE of roots	Swiss mice (male)	Sexual behavior experiment	100, 200, and 400 mg/kg for 42 days	Sildenafil citrate	Daily intake of HaE did not affect the BW and caused no morphometric differences in organs such as the uterus, placenta, and ovaries HaE at a concentration of 200 mg/kg ↑ pregnancy rates in females and fertility in males HaE ↑ ROS levels Mn ↑ at concentrations of 200 and 400 mg/kg of HeE All concentrations of HaE caused germ cell damage ↓ Daily sperm production and elongated spermatids	[186]
			Fertility rates				
			Oxidative stress				
			Mineral quantification in the testis				
			Cell viability				
			Histopathology				
			Sperm evaluation				
			Morphometry				
**Analgesic activity**							
*Alternanthera brasiliana*	AqE aerial part	Wistar rats (male)	Acetic acid‐induced abdominal contractions	25, 50, 100, 200, and 400 mg/kg	Dipyrone	↓ Of contractions for the different []: 90.35%, 91.73%, 95.17%, 94.45%, and 96.55% All [] evaluated had a higher activity than dipyrone	[30]
*Alternanthera maritima*	EE of aerial parts	Swiss mice	Carrageenan‐induced paw edema	30, 100, and 300 mg/kg	—	EE at doses of 300 and 500 mg/kg prevents mechanical hyperalgesia The isolated compound (**72**) at doses of 0.3, 3, and 300 µg/paw prevents a significant ↓ in sensitivity in rats with carrageenan‐induced paw edema Compound **72** inhibited the hyperalgesic effects of TNF and significantly prevented the ↓ in the threshold sensitivity but did not inhibit the hyperalgesic effects of L‐DOPA	[34]
	72	Swiss mice	Carrageenan‐induced paw edema	1, 10, and 20 mg/kg or 3 µg/paw	—		
		Swiss mice	TNF‐ or L‐DOPA‐induced hyperalgesia	3 µg/paw	—		
*Alternanthera philoxeroides*	ME of whole plant	Swiss albino mice	Acetic acid‐induced constriction	50, 100, 200, and 400 mg/kg BW	Aspirin	↓ The number of constrictions in a dose‐dependent manner (%*I*_50 mg/kg_: 31 and %*I*_400 g/kg_: 44.8)	[39]
*Alternanthera sessilis*	EE of whole plant	Swiss albino mice	Hot plates test	250 and 500 mg/kg BW	Morphine	Inhibits the number of contortions by 37.28% (250 mg/kg) and 59.52% (500 mg/kg) the number of contortions Maximum reaction time 6.87 and 7.28 s at doses of 250 and 500 mg/kg EE ↑ pain threshold	[139]
		Swiss albino mice	Writhing test	250 and 500 mg/kg BW	Diclofenac sodium		
*Alternanthera sessilis*	ME of aerial parts	Swiss albino mice (male)	Abdominal writhing test	50, 100, 200, and 400 mg/kg BW	Aspirin	Dose‐dependent activity. ↓ Writhing for the different []: 27.6%, 37.9%, 41.4%, and 44.8%. The last three doses of ME showed greater activity than the control	[232]
*Alternanthera tenella* Colla	EE of whole plant	Swiss and C57bl6	Paw edema, mechanical hyperalgesia, and cold allodynia induced by carrageenan (acetone drop test)	30, 100, and 300 mg/kg	PRED	Four hours after carrageenan injection, EE and compound 43 inhibited edema by 54% (100 mg/kg) and 56% (10 mg/kg), respectively Three hours after carrageenan injection, EE and compound 43 inhibited mechanical hyperalgesia by 99% (300 mg/kg) and 100% (10 mg/kg), respectively	[67]
		Swiss	Knee edema and mechanical hyperalgesia induced by zymosan	100 mg/kg	PRED	Three hours after carrageenan injection, EE inhibited the cold response by 82% at 300 mg/kg, but the isolated compound had no effect A total of 4 and 6 h after zymosan injection, EE and compound 43 blocked mechanical hyperalgesia Four hours after zymosan injection, EE and compound 43 inhibited edema by 58% and 72%, respectively	
	43	Swiss and C57bl6	Paw edema, mechanical hyperalgesia, and cold allodynia induced (acetone drop test) by carrageenan	0.1, 1, and 10 mg/kg	PRED		
		Swiss	Knee edema and mechanical hyperalgesia induced by zymosan	1 mg/kg	PRED		
*Blutaparon portulacoides*	EE of stems	Swiss mice (male)	Paw edema, mechanical hyperalgesia	30, 100, or 300 mg/kg	Dexamethasone	EE ↓ the sensitivity to mechanical stimuli and reduced carrageenan‐induced mechanical hyperalgesia	[71]
		C57BL/6 mice (male)	Mechanical sensitivity and cold sensitivity	30 and 100 mg/kg	Dexamethasone	In mice treated with CFA, EE at a dose of 30 mg/kg inhibited mechanical sensitivity by 60% (6 days), 90% (16 days), and 77% (22 days) and inhibited cold sensitivity in a manner comparable to dexamethasone	
*Gomphrena celosioides*	AqE of leaves	Swiss albino mice	Hot plate test	100, 200, and 400 mg/kg	Morphine	Reaction time: 18.97 ± 0.47, 19.32 ± 1.14, 20.64 ± 0.51. At 400 mg/kg, activity was significant	[87]
			Acetic acid‐induced writhing movement test	100, 200, and 400 mg/kg	Paracetamol	Dose‐dependent activity. Number of writhing movements: 35.78 ± 3.2, 30.16 ± 1.67, 28.27 ± 2.11	
*Gomphrena celosioides*	EE of aerial part	Swiss mice	Paw edema, mechanical hyperalgesia, and cold allodynia induced (acetone drop test) by carrageenan	300, 700, or 1000 mg/kg	Dexamethasone	All doses ↓ carrageenan‐induced edema formation, with a ↑ inhibition of 61% ± 5%, 53% ± 6%, and 68% ± 5%, respectively All doses ↓ hyperalgesia, exhibited ↑ activity at 300 mg/kg with 91% ± 22% Allodynia ↓ at doses of 700 and 1000 mg/kg, with a ↑ inhibition of 58% ± 14% EE significantly ↓ leukocyte migration (58% ± 14%) but did not reduce protein extravasation into the pleural cavity EE ↓ hyperalgesia and leukocyte migration induced with zymosan, with inhibition rates of 52% ± 3% and 81% ± 4%, respectively EE did not significantly alter NO levels EE inhibits cell adhesion to the endothelium (40% ± 7%) and rolling cells (48% ± 6%) EE ↓ edema (25% ± 18%), and CFA‐induced hyperalgesia	[78]
		Swiss mice	Model of carrageenan‐induced pleurisy	300, 700, or 1000 mg/kg	Dexamethasone		
		Swiss mice	Leukocyte recruitment and mechanical model of zymosan	300 mg/kg	—		
		Swiss male mice	Zymosan‐induced peritonitis	300 mg/kg	Dexamethasone		
		Swiss male mice	In situ intravital microscopy analysis	300 mg/kg	Indomethacin		
		C57BL6 mice (male)	Paw edema and mechanical hyperalgesia induced by CFA	100 mg/kg	Dexamethasone		
*Pfaffia glomerata*	HaE of roots and rhizomes	Swiss albino mice (male)	Writhing test (acetic acid)	100, 200, and 300 mg/kg	Indomethacin	%*I* of writhing: 69.1%, 66.4%, and 74.1% for 100, 200, and 300 mg/kg, respectively Showed no effect in the hot plate test, indicating no analgesic effect on the central nervous system	[118]
		Wistar rats (male)	Hot plate test	100, 200, and 300 mg/kg	Morphine		
*Pfaffia glomerata*	HaE of roots	Swiss mice (female)	Acetic acid‐induced constriction	10–300 mg/kg	Indomethacin	Inhibits abdominal constriction by 78% ± 3% at 300 mg/kg and has an ID_50_ of 64.6 mg/kg ↓ Glutamate‐induced nociception in a dose‐dependent manner (ID_50_: 370.8) Antinociceptive activity is not ← by naloxone pre‐treatment Had no effect against nociceptive responses induced by spinal injections of NMDA, AMPA, and kainite but inhibited (32% ± 8%) nociceptive responses induced by spinal injection of *trans*‐ACPD ↓ The biting response was induced by TNF‐α but did not alter the biting response induced by IL‐1β	[231]
		Swiss mice (female)	Glutamate‐induced nociception	100–600 mg/kg	Morphine		
		Swiss mice (female) injected with acetic acid	Involvement of opioid system	300 mg/kg	Morphine		
		Swiss mice (female)	Involvement of glutamatergic system was	300 mg/kg	—		
**Angiogenesis**							
*Alternanthera brasiliana*	HaE of leaves	Wistar rats (male‐trichotomy was performed on the dorsum skin and a dermatological punch of 1.5 cm in diameter)	Histomorphometry and Western blotting	20% HaE of leaves in 2% carbopol gel	—	Favors angiogenesis	[195]
**Antiangiogenic activity**							
*Pfaffia paniculata*	ME of roots	Adult BALB/c mice (male) with corneal lesion	Histopathological study	250, 500, or 1000 mg/kg	—	1000 mg/kg significantly ↓ the number of new blood vessels formed in mouse cornea	[188]
**Anti‐arthritic activity**							
*Alternanthera bettzickiana*	EE of aerial parts	Wistar rats with CFA‐induced arthritis	Determination of physical parameters	250, 500, and 1000 mg/kg	Diclofenac sodium	%*I* of edema: 70.56% and 65.81% for 1000 and 500 mg/kg, respectively 1000 and 500 mg/kg reinstated the arthritis index and BW Significant ↓ in CRP, AST, ALP, ALT, RF, urea, creatinine, and BUN levels Improved [] of RBCs, Hb, WBCs, and ESR ↓ Pannus formation, synovial hyperplasia, inflammatory cell infiltration, bone erosion, bone resorption, joint deformation, soft tissues inflammation, and connective tissue alterations ↑ NF‐kB, IL‐4, IL‐10, I‐kB expression ↓ COX‐2, IL‐6, TNF‐α, IL‐1β expression, and MDA level Restores SOD and CAT values	[19]
			Arthritic Index				
			Hematological and biochemical parameters				
			Histopathological analysis				
			Radiographic assessment				
			RT‐PCR				
			ELISA				
*Alternanthera bettzickiana*	EE of aerial parts	Wistar rats with formaldehyde‐induced arthritis	Hematological and biochemical parameters	250, 500, 1000 mg/kg	Diclofenac sodium	%*I* of edema: 72.11%, 65.25%, and 56.62% for 1000, 500, and 250 mg/kg 500 and 1000 mg/kg significantly ↓ ALP, ALT, and AST levels ↓ Creatinine, urea, CRP, and RF levels 500 and 1000 mg/kg: Significant ↓ in TNF‐α and IL‐6 500 and 1000 mg/kg: ↑ SOD and CAT, and ↓ MDA and NO EE may be an optimal therapy for the treatment of rheumatoid arthritis	[20]
			Enzyme‐linked immunosorbent assay				
			Oxidative stress biomarkers				
**Anticancer activity**							
*Alternanthera brasiliana*	EaE of leaves	EAC bearing Swiss albino mice	Estimation of hematological parameters	200 and 400 mg/kg	5‑FU	↓ BW; doses of 200 and 400 mg/kg ↓ tumor volume, tumor weight, and viable cell count, whereas ↑ the non‐viable cell count Significantly ↑ survival rates (%ILS_200 mg/kg_: 53.33 and %ILS_400 mg/kg_:78.37) ← EAC‐induced changes in Hb, WBCs, and RBCs ↓ SGPT, SGOT, TGL, and ALP, restoring them to normal levels ↑ GSH, SOD, and CAT levels, while ↓ MDA levels Prevented the development of steatosis and lymphocyte accumulation in the liver, maintaining an almost normal liver histology	[190]
			Biochemical analysis	200 and 400 mg/kg	5‑FU		
			Histopathological study	200 and 400 mg/kg	5‑FU		
			Tumor growth response analysis	200 and 400 mg/kg	5‑FU		
			Determination of %ILS	200 and 400 mg/kg	5‑FU		
*Alternanthera sessilis*	Paste of leaves	Swiss mice induced with 3,4‐benzo[α]pyrene (male)	BW measurement	600 mg/g	Uninformed	Had no effect on mice or rats BW Did not significantly inhibit squamous cell carcinoma of the stomach in mice and did not prevent adenocarcinoma development	[228]
			Histopathological study				
		Wistar rats induced with 3′‐methyl‐4‐dimethylaminoazobenzene (male)	BW measurement	600 mg/g	Uninformed		
			Histopathological study				
*Alternanthera tenella* Colla	AqE of aerial parts	Swiss mice (male) injected with EAC cells	Tumor growth response analysis	5 or 50 mg/kg	—	↑ Survival time of mice 50 mg/kg inhibited viable tumor cell count by 59% 5 mg/kg ↓ BW after 8 days of tumor inoculation	[66]
			Determination of %ILS	5 or 50 mg/kg	—		
*Gomphrena celosioides*	Crude powder	DEN/HCB‐induced Albino Wistar rats (male)	Biochemical analysis	200 mg/kg	—	LPO GOT and GPT levels were significantly ↓, indicating antioxidant activity ALP, ACP, and GGT levels significantly ↓ to near normal values, possibly due to the regenerative capacity of liver cells Crude extract showed remarkable activity on SGOT	[133]
	EE of whole plant	DEN/HCB induced Albino Wistar rats (male)	Biochemical analysis	50 mg/kg	—		
*Gomphrena martiana*	Mixture of 10, 6, 7, and 17	S180 bearing BALB/c mice and Swiss mice	BW measurement	20 and 40 mg/kg	—	The mixture of flavonoids ↑ the survival of S180‐bearing mice by 40% The flavonoid mixture ↓ tumor growth in S180‐bearing mice by 21.6% at 40 mg/kg on Day 18 The flavonoid mixture at 20 mg/kg did not ↑ the survival in Ehr Ca‐bearing mice, with only a 10%–20% survival increase at 40 and 60 mg/kg Tumor inhibition in Ehr Ca‐bearing mice was 32% at 40 mg/kg on Day 18	[100]
			Determination of the %*I* of tumor ascites				
	Mixture of 10, 6, 7, and 17	Ehr Ca bearing BALB/c mice and Swiss mice	BW measurement	20, 40, and 60 mg/kg	—		
			Determination of the %*I* of tumor ascites				
*Pfaffia paniculata*	Powdered root	Ehr Ca bearing inbred BALB/cICB mice (male)	Ehrlich ascitic tumor growth	200 mg/kg	—	Significantly ↓ the Ehrlich ascitic volume but had no significant effect on total tumor cell count	[224]
*Pfaffia paniculata*	Powdered root	BALB/c mice treated with *N*‐nitrosodiethylamine	Liver macroscopic analysis	0.5%, 2%, or 10% by weight	—	Male mice treated with 10% powder showed a small incidence of macroscopic lesions, while mice treated with 2% did not show any lesions. Female mice treated with 0.5% and 2% showed a ↓ in macroscopic lesions ↓ Mean lesion number, mean area of the preneoplastic lesions, the % of area with lesions, lesions number by cm^2^, and total preneoplastic lesions ↓ Adenoma incidence in male mice, and adenomas disappeared in female mice	[226]
			Morphologic and morphometric analysis				
*Pfaffia paniculata*	EE of roots AF of roots	Ehr Ca bearing SWISS mice (male)	Survival time	50, 100, or 200 mg/kg/day	—	BuF 50 and 200 mg/kg ↑ survival, but AF and EE had no effect on survival BuFF had no effect on total ascitic volume, tumor cell count per mL, or total tumor cells collected	[248]
		
	BuF of roots	Ehr Ca bearing SWISS mice (male)	Survival time	50, 100, or 200 mg/kg/day	—		
			Ehrlich ascitic tumor growth	50 or 200 mg/kg/day	—		
*Pfaffia paniculata*	ME of roots	Ehr Ca bearing BALB/c mice	Macrophage activity	100, 250, and 500 mg/kg	—	500 mg/kg ↑ the spreading index of peritoneal macrophages and phagocytosis index Had no effect on H_2_O_2_ and NO production	[225]
*Pfaffia paniculata*	Powdered root	BALB/c mice (male) treated with *N*‐nitrosodiethylamine	Histopathological study	0%, 2%, and 10% of weight	—	0% and 10% ↑ relative liver weight Histopathological examination showed diffuse mononuclear inflammatory infiltrates and coagulation necrosis 0% and 10% ↑ cellular proliferation, but 2% ↓ cellular proliferation and PCNA‐positive nuclei 2% and 10% ↑ apoptosis Did not alter intercellular hepatocyte communication via gap junctions	[227]
			Immunohistochemical staining				
			Fluorescence microscopy				
			Alkaline comet assay				
			Western blot				
			Real‐time PCR				
**Antidiabetic and antihyperglycemic activity**							
*Alternanthera philoxeroides*	ME of whole plant	Swiss albino mice	OGTT	50, 100, 200, and 400 mg/kg BW	GLB	↓ Serum glucose levels in a dose‐dependent manner (%*I*_100 mg/kg_: 58.6 and %*I*_400 mg/kg_: 65.6) Doses equal to or greater than 100 mg/kg of ME showed greater activity than GLB	[39]
*Alternanthera sessilis* (red)	FH of EE of aerial parts	Male Sprague Dawley rats induced with STZ	OGTT	500 mg/kg	GLB	FH and AF did not show significant antihyperglycemic effect FEA showed a more significant hypoglycemic effect than GLB FEA ↓ blood glucose levels in the rats over 15 days but did not affect serum insulin levels FEA ↓ HOMA index and ↑ QUICKI index FEA ↓ triglyceride levels (↓ 42.04%) and free fatty acid levels (↓ 34.38%) in plasma but did not alter the triglyceride content FEA ↑ insulin levels and SOD activity in the pancreas	[236]
	AF of EE of aerial parts		OGTT	500 mg/kg	GLB		
	FEA of EE of aerial parts	Male Sprague Dawley rats induced with STZ	OGTT	500 mg/kg	GLB		
			Biochemical assay	250 mg/kg	Pioglitazone		
			Liver triglyceride assay	250 mg/kg	Pioglitazone		
			Pancreatic insulin and SOD assay	250 mg/kg	Pioglitazone		
			Insulin sensitivity indexes	250 mg/kg	Pioglitazone		
*Alternanthera sessilis*	Green leaf juice	Wistar rats (male)	OGTT	NA	—	Did not reduce starch‐ or glucose‐induced postprandial glycemic load	[211]
			OSTT	NA	—		
*Alternanthera sessilis*	ME of aerial parts	Swiss albino mice (male)	OGTT	50, 100, 200, and 400 mg/kg BW	GLB	Dose‐dependent activity. ↓ blood glucose levels by 22.9%, 30.7%, 45.4%, and 46.1%. The highest [] showed activity comparable to that of GLB	[232]
**Antihypertensive effect**							
*Alternanthera sessilis*	EE of whole plant	Sprague–Dawley albino rats	Measures of SDB, DBP, MABP	1–10 mg/kg	Verapamil	↓ SBP, DBP, and MABP Dose‐dependent hypotensive activity	[64]
*Gomphrena celosioides*	EE of aerial parts	Wistar rats (male)—the 1K1C method	Acute model of direct blood pressure measurement	30, 100, or 300 mg/kg diluted	—	EE at [] of 100 and 300 mg/kg ↓ MAP in a dose‐dependent manner, reducing PAM by 36.7 and 38.2 mm Hg, respectively EE acted as a diuretic EE ↑ the Na levels in the urine, while K and Cl levels in urine were similar to the control, and serum remained unchanged in all groups EE ↓ MAP in the 2K1C model, but the effect was lower than of enalapril EE inhibited ACE even more than enalapril, as well as ↓ serum aldosterone [] and TBARS, and ↑ serum nitrite The isolated mesenteric beds in the EE group showed ↓ contractility and reduced pressure ↑ after Phe administration, as well as ↑ relaxation after treatment with ACh and SNP The left ventricle was thinner in EE‐treated rats than in control, and EE did not affect heart, liver, or kidney weights	[80]
		Wistar rats (male)—the 2K1C method	Diuretic assessment	100 mg/kg	Enalapril		
			Blood pressure assessment				
			Urine and serum analysis				
			ACE activity, aldosterone, nitrite, and TBARS				
			Isolation of the mesenteric bed and assessment of vascular reactivity to Phe, ACh, and SNP				
			Organ weighing and histopathology				
					
**Anti‐inflammatory activity**							
*Alternanthera brasiliana*	Infusion of aerial part	Wistar rats (male)	Carrageenan‐induced pleurisy	200 and 400 mg/kg doses	Indomethacin	400 mg/kg significantly ↓ the number of lymphocytes ↓ Exudate by 19.8% (200 mg/kg) and 23.9% (400 mg/kg) ↓ Polymorphonuclear cells (47.5% and 48.02%) and ↑ mononuclear cells (72.13% and 55.74%)	[30]
*Alternanthera brasiliana*	EE of leaves	*Mus musculus* mice	Formalin test	25, 50, and 100 mg/kg	Indomethacin	EE at concentrations of 25, 50, and 100 mg/kg, ↓ the edematogenic process by 35.57%, 64.67%, and 64.17%, respectively	[26]
*Alternanthera brasiliana*	HaE of leaves	Wistar rats (male‐trichotomy was performed on the dorsum skin and a dermatological punch of 1.5 cm in diameter)	Histomorphometry	20% HaE of leaves in 2% carbopol gel	—	20% HaE leaf extract in 2% carbopol gel controls the recruitment of inflammatory cells at the wound site, according to histomorphometry and biochemical analysis 20% HaE leaf extract in 2% carbopol gel modulates inflammation by ↑ IL‐1β and ↓ TGF‐β1 levels HaE at 20% exhibited anti‐inflammatory activity in the acute phase of inflammation	[195]
			Biochemical analysis (MPO and NAG)	20% HaE of leaves in 2% carbopol gel			
			Western blotting	20% HaE of leaves in 2% carbopol gel			
*Alternanthera maritima*	EE of aerial parts	Swiss mice	Carrageenan‐induced paw edema	30, 100, and 300 mg/Kg	Dexamethasone	EE inhibits edema formation at 100 and 300 mg/kg (79%), whereas the isolated compound inhibits it at all [] tested, achieving the ↑ inhibition at 1 mg/kg (76%) EE ↓ leukocyte counts at 100 and 300 mg/kg, with %*I* of 65% and 68%, respectively The isolated compound ↓ leukocyte migration and protein extravasation at all doses, with the ↑ inhibition of leukocyte migration at a dose of 10 mg/kg (77%) and protein extravasation at 20 mg/kg (56%)	[34]
	72	Swiss mice	Carrageenan‐induced paw edema	1, 10, and 20 mg/Kg	Dexamethasone		
	EE of aerial parts	Swiss mice	Carrageenan‐induced pleurisy	30, 100, and 300 mg/Kg	Dexamethasone		
	72	Swiss mice	Carrageenan‐Induced Pleurisy	1, 10, and 20 mg/Kg	Dexamethasone		
*Alternanthera tenella* Colla	AqE of whole plant MHW	BALB/c mice (male) induced with carrageenan	Paw thickness measurement	200 or 400 mg/kg	Indomethacin	The extracts inhibit edema formation in a dose‐dependent manner AqE MCW and MHW showed greater inhibition of edema than indomethacin, with a %*I*_400 mg/kg_ of 61% and 56% at 3 h, respectively	[234]
	AqE of whole plant MCW		Paw thickness measurement	200 or 400 mg/kg	Indomethacin		
*Alternanthera tenella* Colla	EE of whole plant	Swiss mice	Zymosan‐induced articular inflammation	100 mg/kg	PRED	Four hours after carrageenan injection, EE and **43** inhibited edema by 54% at 100 and 1 mg/kg, respectively Four hours after zymosan injection, EE and **43** inhibited edema by 58% and 72%, respectively EE and 43 ↓ the total leukocyte count in synovial fluid by 65% and 61%, respectively EE and 43 blocked leukocyte migration and inhibited edema proteins by 54% and 72%, respectively Twenty‐four hours after CFA‐injection, EE and 43 inhibited MPO activity by 82.86% and 79.15%, respectively, and also inhibited NAG by 67.87% and 68.56%	[67]
			Carrageenan‐Induced Pleurisy	100 mg/kg	PRED		
		C57bl6 mice	MPO and NAG activity assay	100 mg/kg			
	43	Swiss mice	Zymosan‐induced articular inflammation	1 mg/kg	PRED		
			Carrageenan‐induced pleurisy	1 mg/kg	PRED		
		C57bl6 mice	MPO and NAG activity assay	100 mg/kg			
*Blutaparon portulacoides*	EE of aerial parts	Swiss mice (male)	Venom of *Bothrops jararacussu* induced paw edema	100, 250, or 500 mg/kg	Dexamethasone	EE at 250 and 500 mg/kg inhibits venom‐induced edema formation by 28.5% and 39% within 6 h, respectively EE also ↓ the edematogenic effect induced by BthTX‐I and BthTX‐II from 30 min to 6 h (end of experiment) EE had no effect on leukocyte migration induced by venom or BthTX‐II, but it significantly inhibited the leukocyte flux induced by BthTX‐I	[235]
			BthTX‐I‐ and BthTX‐II‐induced paw edema	500 mg/kg	Dexamethasone		
			Venom of *Bothrops jararacussu* induced pleurisy	500 mg/kg	Dexamethasone		
			BthTX‐I‐ and BthTX‐II‐induced pleurisy	500 mg/kg	Dexamethasone		
*Blutaparon portulacoides*	EE of stems	Swiss mice (female)	Carrageenan‐induced pleurisy	30, 100, 300, and 1000 mg/kg	Dexamethasone	Only at 1000 mg/kg was a 55% inhibition of leukocyte invasion in the pleura, and protein exudation was ↓ at doses of 300 and 100 mg/kg, as well as a ↓ in IL‐1β levels EE ↓ edema in a dose‐ and time‐dependent manner, achieving a 67% reduction at 4 h in mice injected with carrageenan, and EE ↓ edema in mice treated with CFA in a manner comparable to that of dexamethasone, reaching an 80% inhibition at Day 22	[71]
		Swiss mice (male)	Carrageenan‐induced paw edema	30, 100, or 300 mg/kg	Dexamethasone		
		C57BL/6 mice (male)	CFA	30 and 100 mg/kg	Dexamethasone		
		C57BL/6 mice (female)	BCG‐induced pleurisy	30 and 100 mg/kg	Isoniazid		
*Gomphrena celosioides*	AqE of leaves	Adult Sprague–Dawley rats and Swiss albino mice	Carrageenan‐induced edema	100, 200, and 400 mg/kg	Indomethacin	Dose‐dependent activity %*I* of edema: 27.97, 39.62, and 39.62 At 100 mg/kg, the effect was not significant	[87]
*Gomphrena celosioides*	EE of whole plant	Wistar albino rats	Carrageenan induced paw edema	200 mg/kg of BW	Diclofenac	↓ CRP levels, possibly due to the inhibition of inflammatory mediators Inhibition of edema	[77]
*Pfaffia glomerata*	HaE of roots and rhizomes	Wistar rats (male)	Carrageenan induced paw edema	100, 200, and 300 mg/kg	Dexamethasone	%*I* of edema: 46.3, 56.8, and 63.2 for 100, 200, and 300 mg/kg, respectively HaE does not inhibit cell migration ↓ Granulomatous tissue formation by 29%	[118]
			Granulomatous tissue assay	100 mg/kg	Dexamethasone		
*Pfaffia glomerata*	HaE of roots	Swiss mice (male)	Carrageenan induced paw edema	1, 10, 30, 100, or 300 mg/kg	Indomethacin	↓ Carrageenan‐induced paw edema in a dose‐dependent manner (ID_50_: 60.5 (dose oral) and 20.4 (dose intraperitoneal)) ↓ Edema induced by bradykinin and substance P, highlighting that 300 mg/kg of HaE completely ↓ edema at 120 min. These results suggest that HaE has antinociceptive activity ↓ Edema induced by histamine, serotonin, and LPS ↑ NO levels When the ↑ in NO levels is blocked with L‐NAME, a ↓ in anti‐edema activity is observed	[119]
		Swiss mice (male) with carrageenan‐induced edema	Evaluation of the influence of NO synthase and guanylate cyclase inhibition	300 mg/kg	Indomethacin		
		Swiss mice (male)	Bradykinin induced paw edema	1, 10, 30, 100, or 300 mg/kg	Indomethacin		
		Swiss mice (male)	Substance P induced paw edema	1, 10, 30, 100, or 300 mg/kg	Indomethacin		
		Swiss mice (male)	Serotonin induced paw edema	1, 10, 30, 100, or 300 mg/kg	Indomethacin		
		Swiss mice (male)	Histamine induced paw edema	1, 10, 30, 100, or 300 mg/kg	Indomethacin		
		Swiss mice (male)	LPS induced paw edema	1, 10, 30, 100, or 300 mg/kg	Indomethacin		
*Pfaffia paniculata*	ME of roots	Wistar rats (male)	TNBS induced intestinal inflammation	25, 50, 100, 200, or 400 mg/kg	Prednisolone	No ↓ macroscopic damage score in the preventive protocol, but at 200 mg/kg in the curative protocol, ↓ in gross damage score was observed Doses of 50 and 100 mg/kg ↓ microscopic damage score (↑ mucosal regeneration) ↓ Polymorphonuclear cell infiltration, fewer ulceration sites, ↓ dilated crypts, and depletion of goblet cell 200 mg/kg, ↓ MPO activity was observed, and doses of 50 or 200 mg/kg prevented glutathione depletion in the curative protocol ↓ IL‐1β, IFN‐γ, and C‐reactive protein levels At 200 mg/kg, ↓TNF‐α and IL‐6 levels It was not able to prevent TNBS‐induced intestinal inflammation but was able to ↓ colonic inflammation	[215]
*Pfaffia paniculata*	ME of roots	Male Wistar rats (male)	Intestinal inflammation assessment	25, 50, 100, and 200 mg/kg	—	At 200 mg/kg ↓ gross damage score, extent of injury, and MPO activity were observed At 25 mg/kg, ↓ Hsp70; 50 mg/kg, ↓ Mapk3 and ↑ Muc4; 100 mg/kg, ↑ Mapk1, Muc3, Muc4, and ↓ Mapk3; at 200 mg/kg, ↓ Mapk3 No effect was observed on the mRNA levels of heparanase, NF‐κB, Mapk6, Mapk9, Muc1, or Muc2 Inflammatory activity was related to the differential modulation of MAPKs and the expression and production of mucin	[120]
			Inflammatory mediator's analysis	25, 50, 100, and 200 mg/kg	—		
*Pfaffia townsendii*	EE of whole plant	Swiss mice (male)	Carrageenan‐induced paw edema	300 mg/kg	Dexamethasone	EE inhibits the formation of edema by 51.00% ± 11.0%, while compound 67 inhibited it by 75.4% ± 4.0% and compound 98 by 73.00% ± 4.0% EE inhibited leukocyte migration to the pleura by 69.2% ± 1.04%, while compounds 67 and 106 by 50.7% ± 1.03% and 59.4% ± 1.25%, respectively EE ↑ plasma leakage, whereas flavonoids ↓ plasma leakage The anti‐inflammatory activity of compound 67 was similar to that of dexamethasone	[127]
	67			1 mg/kg			
	106			1 mg/kg			
	EE of whole plant	Swiss mice (female)	Carrageenan‐induced pleurisy	300 mg/kg	Dexamethasone		
	67			1 mg/kg			
	106			1 mg/kg			
**Antimutagenic activity**							
*Pfaffia glomerata*	Commercial root dry extract	Wistar rats (*Rattus norvegicus*) treated with cyclophosphamide	Chromosomal aberration test	1.5 mg/mL simultaneous or pretreatment or post‐treatment	—	Significantly ↓ the % of damage induced by cyclophosphamide; damage was ↓ by 87% for simultaneous treatment, 98% for pretreatment, and 99% for post‐treatment	[112]
**Antispasmodic activity**							
*Gomphrena perennis*	HaE of aerial parts	Sprague–Dawley rats (female)	Carbachol concentration‐response (CCh‐CRCs)	0.14–28.1 mg extract/mL	—	HaE demonstrated antispasmodic activity through several mechanisms, highlighting the non‐competitive inhibition of Ca^2+^ influx. Significant values were obtained for the inhibition of CCh‐CRCs in a concentration‐dependent manner	[140]
			Calcium concentration‐response (Ca^2+^‐CRCs)				
			Relaxation response concentration (RRC)				
**Antioxidant activity**							
*Alternanthera brasiliana*	HaE of leaves	Wistar rats (male‐trichotomy was performed on the dorsum skin and a dermatological punch of 1.5 cm in diameter)	Biochemical analysis: TBARS and antioxidants—SH groups	20% HaE of leaves in 2% carbopol gel	—	Relevant antioxidant activity was observed on Day 2, probably to control oxidative damage	[195]
*Gomphrena celosioides*	EE of whole plant	Wistar albino rats	FRAP and TBARS assays	200 mg/kg of BW	Vitamin C	Significantly ↓ serum TBAR levels ↑ Serum levels total antioxidant capacity Significant ↓ in Fe^3+^ to ion Fe^2+^ ion activity	[77]
*Pfaffia glomerata*	FD of roots	C57BL/6 mice	Tissue oxidative induction	250, 25, and 2.5 µg/mL	Quercetin	FDs showed inhibitory activity against lipid peroxidation and the formation of ROS. In particular, only the FD of aerial parts, at the highest concentration, showed significant total antioxidant capacity	[167]
	FD of aerial part		Total antioxidant capacity				
			Lipid peroxidation				
			Sulfhydryl groups content				
			Reactive oxygen species (ROS)				
**Antiviral activity**							
*Alternanthera philoxeroides*	238	BALB/c mice inoculated HSV‐2 strain UW 264	Determination of clinical signs and viral shedding	0.3 or 0.6 mg	—	Dose‐dependent protection against HSV‐2 In the 3rd day of treatment, it ↓ the mean titers of virus shed by 33.33% Significantly suppressed herpetic lesions ↑ Survival by 40 and 60% for doses of 0.3 and 0.6 mg, respectively	[41]
**Cardioprotective activity**							
*Gomphrena celosioides*	AqE of stems, flowers, and leaves	Wistar albino rats induced with DOX	Biochemical study	200 and 500 mg/kg for 14 days	Resveratrol	AqE and EE ↓ the [] of ALT, AST, CK‐MB, cholesterol, and triglycerides in serum AqE and EE ↑ the [] of HDL‐C It ↓ the weight loss of the rat but did not affect the relative weight of the heart	[76]
			RW of the body and heart				
	EE of stems, flowers, and leaves.	Wistar albino rats induced with DOX	Biochemical study	200 and 500 mg/kg for 14 days	Resveratrol		
			RW of the body and heart				
*Gomphrena perennis*	HaE of aerial parts	Sprague–Dawley rats (female)	Langendorff method with control Krebs solution (Krebs‐C), Ischemia/reperfusion (I/R) model	Oral 25 mg HaE/kg/day and HaE at 0.1% v/v were perfused for 15 min prior to I/R	Krebs‐C	HaE has a cardioprotective effect due to its action on NO production, attributed to the presence of flavones in its composition	[140]
*Blutaparon portulacoides*	AqE of whole plant	SHRs and Wistar‐Kyoto rats (male)	Electrocardiography	30, 100, and 300 mg/kg	HCTZ	AqE prevents changes in the RW of the heart and left ventricle, as well as changes in the levels of MDA and NT AqE has significant diuretic and cardioprotective effects	[155]
			Blood pressure				
			Biochemical analysis				
			RW of organs, histopathology, and heart morphometry				
**Diuretic activity**							
*Gomphrena celosioides*	EE of aerial parts	Wistar rats (male)	Single‐dose model of diuretic assessment	30, 100, and 300 mg/kg	HCTZ	EE ↑ urine volume, comparable to HCTZ, DI_100 mg/kg_: 1.74 ± 0.25 and DI_300 mg/kg_: 1.86 ± 0.28 EE had a higher UNa value than HCTZ EE had no effect on urine K^+^, Ca, and Cl^−^ [], nor on pH, density, serum electrolytes, urea, and creatinine After pretreatment with L‐NAME, indomethacin, or HOE‐140, EE did not significantly promote diuresis or natriuresis During the 7 days of treatment, an ↑ in UV and UNa and ↓ aldosterone levels were observed	[81]
			Assessment of the involvement of the prostaglandin, bradykinin, and NO pathways	100 mg/kg	HCTZ		
			Urine and serum analysis Urine	100 mg/kg	HCTZ		
*Gomphrena perennis*	HaE of aerial parts	Sprague–Dawley rats (female)	Quantification of Na+ and K+ content urinary volumetric excretion (UVE %)	100 and 400 mg/kg	Amiloride	HaE did not show an ↑ in the total urine volume at the evaluated doses and did not cause a differences in ion excretion in the animal's urine	[140]
*Blutaparon portulacoides*	AqE of whole plant	SHRs and Wistar‐Kyoto rats (male and SHRs)	Biochemical analysis	30, 100, and 300 mg/kg	HCTZ	300 mg/kg of AqE prevents changes in renal sodium and chloride excretion, maintaining urinary volume and electrolyte elimination. AqE does not increase the potassium elimination, preventing the appearance of cramps and arrhythmia 300 mg/kg of AqE has diuretic effects in SHRs	[155]
**Gastrointestinal activity**							
*Alternanthera repens*	ME	CD1 strain mice (male)	Evaluation of the number of feces	50 and 100 mg/kg	Diphenoxylate	AqE (%*I*_100 mg kg_: 49 ± 6.9) and ME (%*I*_50 mg kg_: 37.9 ± 0.7) significantly ↓ castor oil‐induced diarrhea HeE and ClE did not show antidiarrheal activity ME modifies normal defecation (%*I*_100 mg/kg_: 59.6 ± 3.2) ME has dose‐dependent antidiarrheal activity at 12.5, 25, and 50 mg/kg, with highest effect at 50 mg/kg in mice with MgSO_4_‐induced diarrhea (%*I*:75.9) ME ↓ intestinal transit by 24% at 60 min, but at 90 min, intestinal transit is returned to 100%	[197]
	AqE or ClE or HeE or ME	CD1 strain mice (male) treated with castor oil or MgSO_4_	Evaluation of the antidiarrheal activity	50 and 100 mg/kg	Diphenoxylate		
	ME	CD1 strain mice (male) treated with castor oil	Evaluation of the antidiarrheal activity	12.5, 25, 50, and 100 mg/kg	Diphenoxylate		
	ME	Wistar rats (male) mice treated with castor oil	Effect on small intestinal transit	12.5, 25, 50, and 100 mg/kg	Diphenoxylate		
*Alternanthera repens*	AqE of aerial parts	Swiss mice (female)	Charcoal meal method	1–300 mg/kg	Atropine	The extracts ↓ gastrointestinal content and contain metabolites with antidiarrheal activity	[46]
	EE of aerial parts	Swiss mice (female)	Charcoal meal method	1–300 mg/kg	Atropine		
**Gastroprotective activity**							
*Gomphrena celosioides*	ME of leaves	Wistar rats (male) induced with indomethacin	Determination of gastric volumes, pH, acid outputs, ulcer score, and ulcer index Biochemical analysis	200, 500, and 800 mg/kg BW	Cimetidine	↑ pH in a dose‐dependent manner, and at 800 mg/kg this ↑ was more significant than that of cimetidine ↓ Acidity, gastric volume, ulcer index, ulcer score, and pepsin activity in a dose‐dependent manner, with these effects being more marked at 800 mg/kg of ME than cimetidine ↓ MDA levels and ↑ protein levels ME displays an antiulcerogenic effect related to its gastroprotective activity	[88]
*Gomphrena celosioides*	ME of leaves	Wistar rats (male) induced with acidified ethanol	Biochemical analysis	200, 400, and 800 mg/kg BW	Cimetidine	ME ↓ the increase in gastric volume and acid output, and ↑ the mucus content ↑ SOD, GSH, and GPX activities ↓ LPO and XO levels ME promotes the restoration of the epithelial layer, lamina propria, and submucosal layer in a dose‐dependent manner	[246]
			Histopathological study				
*Guilleminea densa*	HaE of leaves	Holtzman rats induced with ethanol (male)	Macroscopic analysis	200, 400, and 600 mg/kg	Ranitidine and sucralfate	Dose‐dependent gastroprotective effect (%*I* of gastric lesions_400 mg/kg_: 58,78 and (%*I* of gastric lesions_600 mg/kg_: 82,72) Significantly ← mucus depletion induced by ethanol at HaE [] of 400 and 600 mg/kg but had no effect on NP‐SG levels At 600 mg/kg, it inhibited gastric erosions, ulcers, acute inflammation infiltration, and focal bleeding development	[102]
			Determination of gastric mucus and NP‐SG				
			Histopathological study				
*Pfaffia glomerata*	AqE of roots and rhizomes	Wistar rats (female) with ulcers induced by restriction or ethanol or indomethacin	Determination of IMD	125, 250, 500, and 1000 mg/kg	Ranitidine	↓ Ethanol‐induced gastric lesion in a dose‐dependent manner, reducing them by over 90% At 250 and 1000 mg/kg, ↓ stress‐induced gastric lesion by 37.8% and 47.8%, respectively The AqE did not show gastric mucosa protective activity against indomethacin‐induced ulcers ↑ pH The ↓ in total acidity and gastric volume in rats with a pylorus ligation is associated with an ↑ in NO*_x_* content AqE has no effect on total acidity and gastric secretion volume in rats treated with bethanechol or pentagastrin At 2000 mg/kg, ↓ total acidity by 18.4% and gastric secretion volume by 53.2% in histamine‐injected rats	[164]
		Wistar rats (female) with pylorus ligation	Determination of gastric acid secretion	125, 250, 500, and 1000 mg/kg	—		
		Wistar rats (female) with pylorus ligation and administration of bethanechol, histamine or pentagastrin	Determination of gastric acid secretion	125, 250, 500, and 1000 mg/kg	—		
		Wistar rats (female) with pylorus ligation	Determination of nitric oxide production	1500 mg/kg	—		
**Hepatic activity**							
*Pfaffia glomerata*	HaE of roots	Swiss mice (male)	Biochemical analysis	100, 200, and 400 mg/kg	—	The HaE showed effects such as mineral content changes, antioxidant enzymes, and morphological modifications In general, HaE generated oxidative stress severe enough to induce liver damage	[185]
			Liver oxidative stress markers				
			Morphology				
			Histology and histopathology				
			Mineral and hepatic glycogen content				
**Hepatoprotective activity**							
*Alternanthera brasiliana* L.	HaE of leaves	BALB/c mice (male) induced with CCl_4_	Body weight, liver weight, and liver morphology	200 and 400 mg/kg	—	HaE restored the BW ↓ Liver index compared to CCl_4_ alone Restore ALT, AST, and ALP levels Restored liver size to normal, decreased the echogenic pattern, recovered nodular edge appearance, and ↓ the CBD dilation 400 mg/kg of HaE improvement hepatic architecture, showing minimal mononuclear cell infiltration and a ↓ number of mitotic figures HaE significantly ↓ MDA levels Restore GSH, GST, SOD, and vitamin C levels ↓ TNF‐α, IL‐1β, and IL‐18 levels 400 mg/kg of HaE significantly ↓ phospho–NF–kB (p65) and NLRP3 protein levels 400 mg/kg of HaE ↓ MMP‐2 and MMP‐9 levels and restores TIMP‐1 ↓ TGF‐β, α‐SMA, and *p*‐Smad2/3 protein levels HaE leaf extracts may serve as an herbal hepatoprotective agent	[208]
			Biochemical analysis (AST, ALT, and ALP)		—		
			Lipid peroxidation and antioxidant parameters		—		
			Histopathological study		—		
			Western blot analysis		—		
			qRT‐PCR analysis		—		
			ELISA		—		
*Alternanthera sessilis*	ME of whole plant	Wistar rats (male) induced with CCl_4_	Biochemical analysis (TBARS, GSH, CAT)	50, 200, and 250 mg/kg	Silymarin	At 250 mg/kg, ← the ↑ in serum SGPT, SGOT, and ALP levels Generates a ↓ in serum cholesterol and bilirubin levels It ↓ lipid oxidation, ↑ GSH levels, and improves CAT levels At higher doses, ← body degeneration (↓ necrosis and restores cellular integrity)	[51]
			Histopathological study				
*Gomphrena celosioides*	AqE of stems and leaves	Wistar rats induced with CCl_4_ (PT and TC)	Biochemical analysis (AST, ALT, ALP, BT, and CB)	500 mg/kg BW for 5 days	Silymarin	AqE ↓ AST, ALT, ALP, BT, and CB values, indicating a preventive (PT) and restorative (TC) effect With PT and CT, liver lesions are ↓ severe, with PT showing greater hepatoprotective activity	[90]
			Histopathological study				
*Gomphrena globosa* L.	AqE	C57BL/6 mice (male) with CCl_4_	Biochemical analysis (AST, ALT, ROS, and SOD)	100, 200, or 300 mg/kg	Bifendatatum	AqE ↓ serum AST, ALT levels, and hepatic MPO AqE improves hepatic total protein content It ↓ ROS and MDA levels and ↑ GSH, GSH‐Px, and SOD activities AqE improves liver injury in a dose‐dependent manner It activates Nrf2 protein expression and regulates Keap1 levels It activates GCLC, GCLM, HO‐1, and NQO1 protein expression It ↓ PI3K and mTOR phosphorylation, inhibits P62 protein expression, and activate LC3 II protein expression It promotes autophagy AqE alleviates CCl4‐induced chronic liver injury in mice by activating antioxidant signaling pathways and promoting autophagy	[249]
			Measurement of MPO, TP, MDA, GSH, and GSH‑Px				
			Histopathological studies				
			Western blot				
**Immunomodulatory activity**							
*Alternanthera tenella* Colla	AqE of aerial parts	Swiss mice (male) immunized with sheep RBC	Antibody assays	5 and 50 mg/kg	—	At 5 mg/kg, it ↓ anti‐SRBC IgM secreting cells prior to immunization, but 50 mg/kg has no effect on PFC count 50 mg/kg improves the production of IgM and IgG2a antibodies in mice stimulated with LPS It had no effect on the total number of nucleated spleen cells or spleen weight	[66]
		LPS stimulated mice (male)	Antibody assays	5 and 50 mg/kg	—		
*Alternanthera tenella* Colla	AqE of whole plant MHW	BALB/c mice (male) immunized with sheep RBC	Measurement of BW and lymphoid organs	50, 100, or 200 mg/kg BW	BALB/c mice (male) only immunized with sheep RBC	The extracts at 50, 100, and 200 mg/kg did not show significant differences in BW and lymphoid organ weight AqE MCW ↑ liver weight by 19.5% The extracts did not affect spleen cellularity AqE MHW and AqE MCW maintained cell viability at 88% and 90%, respectively Only AqE MCW at 100 mg/kg significantly ↑ PFC, suggesting the presence of immunomodulators AqE MCW ↑ anti‐SRBC IgM and IgG titers, but AqE MHW did not show any significant effect on antibody titers	[234]
			Splenic cellularity				
			PFC assay				
			Antibody assays				
	AqE of whole plant MCW	BALB/c mice (male) immunized with sheep RBC	Measurement of BW and lymphoid organs	50, 100, or 200 mg/kg BW	BALB/c mice (male) only immunized with sheep RBC		
			Splenic cellularity				
			PFC assay				
			Antibody assays				
**Neuropharmacological activity**							
*Alternanthera brasiliana*	Infusion of aerial part	Wistar rats (male)	Open field test	100, 200, and 400 mg/kg	—	There was no effect on latency time in the first rectangle or the number of crossings 100 mg/kg ↑ the number of rearings, while 200 mg/kg ↓ the number of fecal boluses	[30]
			Elevated plus maze	100, 200, and 400 mg/kg	—		
*Alternanthera brasiliana*	ME of leaves	Swiss albino mice (male)	Hole board test	100, 300, and 600 mg/kg	Diazepam	ME at 300 and 600 mg/kg significantly ↑ the number of times and duration of the mice that poke their heads It ↑ rearing, assisted rearing, and the number of squares traveled, showing comparable or superior activity to diazepam It ↑ in the number of entries into the open arm but ↓ entries into the close arm It ↑ time spent in lighted box, crossing numbers, and transfer latency, while ↓ time spent in the dark box It ↓ locomotor activity in a dose‐dependent manner (CNS depressant effect) ME has anxiolytic activity It protected mice from PTZ‐induced seizures in a dose‐dependent manner, achieving maximum protection of 66.66% ME at 600 mg/kg ↓ the latency of maximal electroshock‐induced seizures, but had no effect on seizure incidence	[24]
			Open field test	100, 300, and 600 mg/kg	Diazepam		
			Elevated plus maze test	100, 300, and 600 mg/kg	Diazepam		
			Light/dark exploration test	100, 300, and 600 mg/kg	Diazepam		
			Locomotor Activity test	100, 300, and 600 mg/kg	Diazepam		
		PTZ injected Swiss albino mice (male)	Chemoshock convulsion	100, 300, and 600 mg/kg	Diazepam		
		Swiss albino mice (male)	Maximal electroshock induced convulsion	100, 300, and 600 mg/kg	Diazepam		
*Alternanthera philoxeroides*	EE of whole plants	ICR‐OVX mice (female)	FST	250 and 500 mg/kg	17β‐Estradiol	It ↓ immobility time in FST and TST It did not alter locomotor activity in OVX mice It ↓ serum corticosterone levels in a dose‐dependent manner It normalized the expression of CREB and BDNF	[38]
			TST				
			LAT				
			Corticosterone ELISA				
			RT‐PCR				

*Alternanthera philoxeroides*	EE of whole plant	ICR‐OVX mice (female)	NORT	250 and 500 mg/kg/day	17β‐Estradiol	It significantly improved the acquisition and retrieval of reference memory, ↓ by OVX, in a dose‐dependent manner It significantly improves discrimination performance in NORT (enhanced recognition memory) It restored spatial working memory deficits induced by OVX It significantly ↑ the % of spontaneous alternation It ↓ significant MDA values, indicating antioxidant activity It ↓ IL‐1β, IL‐6, and TNF‐α mRNA expressions It normalized the expression of the PI3K and AKT genes Metabolomic analysis showed that 500 mg/kg of EE had better effects against OVX‐induced alterations, and the most relevant metabolites in the study were responsible for the galactose metabolic pathway	[141]
			Y‐Maze Task	250 and 500 mg/kg/day	17β‐Estradiol		
			MWMT	250 and 500 mg/kg/day	17β‐Estradiol		
			Locomotor Activity Test	250 and 500 mg/kg/day	17β‐Estradiol		
			Lipid peroxidation of the brain	250 and 500 mg/kg (mice)	17β‐Estradiol (mice)		
			Bradford's method (brain)	250 and 500 mg/kg (mice)	17β‐Estradiol (mice) and BSA (testes)		
			RT‐PCR	250 and 500 mg/kg (mice)	17β‐Estradiol (mice)		
			NMR‐metabolomic analysis	250 and 500 mg/kg (mice)	17β‐Estradiol (mice)		
*Alternanthera philoxeroides*	EE of whole plant	ICR mice (male)	Behavioral assessment	250 and 500 mg/kg/day	Vitamin E	EE contributed to the maintenance of short‐term and long‐term memory in behavioral tests The two EE concentrations evaluated were able to restore SOD and CAT levels to healthy conditions EE activity regulates the expression of mTERT, mTRF1, and mTRF2, leading to a delay in telomere shortening	[142]
			Biochemical assay				
			Determination of CAT and SOD activities				
			qPCR				
*Alternanthera sessilis*	EE of whole plant	Swiss Albino mice	Pentobarbital‐induced sleeping time	250 and 500 mg/kg	Caffeine	It ↑ the time required for the onset of sleep by 188.70% and 377.49% and ↓ its duration by 12.70% and 23.08%, at doses of 250 and 500 mg/kg, respectively Locomotion ↑, but after reaching maximum activity, the stimulating effect gradually ↓ with passage of time	[139]
			Open field test	250 and 500 mg/kg	—		
			Hole cross test	250 and 500 mg/kg	—		
*Iresine celosia*	EE of aerial parts	ICR mice (male) treated with LPS	Open field test	30 or 100 mg/kg	—	100 mg/kg significantly ↓ LPS‐induced activated microglia and number of S100β‐positive cells It ↑ ambulation in open field tests and improved T‐turn and T‐LA Inhibition of microglia and astrocytes alleviated behavioral dysfunction	[104]
			Pole test				
			Immunohistochemistry and image analysis				
*Pfaffia glomerata*	EE of roots and rhizomes	Wistar rats (male)	Open field test	500 mg/kg	Diazepam	Rats in the open‐field test exhibited behavior similar to those treated with diazepam 500 mg/kg ↓ sleep latency and ↑ sleeping time, but 1000 mg/kg had no effect on latency and sleep duration 500 mg/kg ↑ latency and a ↓ in the duration of the first convulsion, but 1000 mg/kg had no effect It ↓ entries in enclosed arms 500 and 1000 mg/kg ↓ step‐down latency in a []‐dependent manner 1500 mg/kg showed a tendency to ↑ the memory retention, but this effect was not statistically significant No antidepressant effect was observed	[245]
			Elevated plus maze test	500 mg/kg	Diazepam		
			Step‐down inhibitory avoidance task	100, 500, 1000, and 1500 mg/kg	Diazepam		
			FST	500 mg/kg	Imipramine hydrochloride		
		Swiss mice (male)	Pentobarbital‐induced sleeping time	500 and 1000 mg/kg	Diazepam		
			Pentylenetetrazole‐induced convulsions	500 and 1000 mg/kg	Diazepam		
*Pfaffia glomerata*	HaE of roots	Albino mice (male)	Spontaneous movement	10 and 100 mg/kg	—	It causes abdominal contraction when administered intraperitoneally It ↓ motor activity and stereotypy At 10 mg/kg, it ↓ scaling behavior and grooming 100, 200, and 1000 mg/kg caused ruffled fur 100 mg/kg ↓ sleep time but did not ← memory retention damage caused by scopolamine Old rats treated with HaE performed similarly to young rats in the discrimination test, demonstrating that HaE improved acquisition and retention of determined behavior HaE partially reversed age‐associated memory deficit. It ↓ BW	[116]
			Rota‐rod	10 and 100 mg/kg	—		
			Potentiation of sodium pentobarbital sleeping time	10 and 100 mg/kg	—		
		Albino mice treated with scopolamine	Passive avoidance test	100 mg/kg	—		
		Young and old Wistar rats (male)	Passive avoidance test	100 mg/kg	—		
		Old Wistar rats	Right–left discrimination test	100 mg/kg	—		
*Pfaffia glomerata*	HF of ME of roots	C57BL/6J mice (male) with acute stress	Open‐field test	3, 10, or 30 mg/kg	—	Avoid ↑ motor function of mice (↓ stress‐related behavior) It ↓ depressive‐like behaviors At 30 mg/kg, it ↓ the time spent in closed arms after Day 2 and gradually ↓ the time spent in the open arms of the maze. At 10 mg/kg, it ↓ immobility time after Day 2 (protective effect in anxiety development) It did not show any effect in the rotation test It prevented the ↓ of SOD and GPx activity in the cortex and striatum, but not in the hippocampus. It restores CAT activity in the striatum, but not in the cortex or hippocampus	[114]
			Elevated plus maze test	3, 10, or 30 mg/kg	—		
			FST	3, 10, or 30 mg/kg	—		
			Rotation test	3, 10, or 30 mg/kg	Diazepam		
			Biochemical analysis of tissues	3, 10, or 30 mg/kg	—		
**Wound‐healing activity**							
*Alternanthera brasiliana*	Ointment of ME of leaves	Sprague Dawley rats (excision wound model)	Wound area contraction measures	5%	Himax	ME ointment completely contracted the excision wound (100%), showing greater activity than Himax Mice treated with ME ointment had a granulation tissue completely filled with epidermal cells covered by a thick layer of keratin ME ointment generated a tensile strength of the healing tissue of 4.861 ± 0.664, indicating wound‐healing activity	[244]
			Histopathological study				
		Sprague Dawley rats (incision wound model)	Wound area contraction measures	5%	Himax		
*Alternanthera brasiliana*	Ointment of ME of leaves	Sprague Dawley rats (dermal burn wound)	Wound area contraction measures	5%	Himax	On Day 8, it ↓ the wound area by 92.13%, showing greater activity than Himax (72%) It ↑ protein and hydroxyproline content It ↑ CAT, GSH, and SOD levels in granulation tissue It ↑ Vitamin C levels ME has good wound‐healing activity, as histopathological studies showed collagen fiber deposition and a keratin layer, indicating tissue recovery and regeneration	[250]
			Biochemical estimations				
			Histopathological study				
*Alternanthera brasiliana*	Ointment of ME of leaves	Immunocompromised Sprague Dawley rats with HC	Wound area contraction measures	2.5%, 5.0%, and 7.5%	Himax	An ointment with 5% ME achieved 77.10% wound contraction on Day 8, outperforming Himax (60%) It ↑ GSH, CAT, and SOD levels, as well as protein content in granulation tissues At 5%, the highest levels of hydroxyproline and vitamin C were observed, exceeding those of Himax Mice treated with ME showed abundant collagen fibers, fibroblast proliferation, angiogenesis, and development of basement membrane beneath the necrotic debris	[251]
			Biochemical estimations				
			Histopathological Study				
*Alternanthera brasiliana*	Ointment of ME of leaves	Aged Sprague Dawley rats (aged wound model)	Wound area contraction measures	5%	—	The % of wound contraction ↑ to 97.62 ± 0.14 on Day 21, indicating wound‐healing activity It ↑ the content of collagen, elastin, and hydroxyproline by 16.33% ± 0.42%, 6.67 ± 0.42%, and 64.67 ± 3.83 (mg/g) on Day 21, respectively Granulation tissues showed abundant collagen fibers and re‐epithelization	[252]
			Biochemical estimations				
			Histopathological Study				
*Alternanthera brasiliana*	HaE of leaves	Wistar rats (male‐trichotomy was performed on the dorsum skin and a dermatological punch of 1.5 cm in diameter)	Re‑epithelialization analysis	20% HaE of leaves in 2% carbopol gel	—	According to histomorphometry analysis, the extract did not induce a significant fibroblast proliferation According to biochemical studies, the collagen formation ↑ on the 2nd day. Additionally, they observed that it stimulated wound healing, as it gradually reduced collagen III levels, and after Day 21, collagen I increased	[195]

Abbreviations: ACh, acetylcholine; AF, aqueous fraction; ALT, alanine aminotransferase; AST, aspartate aminotransferase; CAT, catalase; CFA, Freund's complete adjuvant; DBP, diastolic blood pressure; EE, ethanolic extracts; ELISA, enzyme‐linked immunosorbent assay; FD, fraction of DCM; FEA, fraction of ethyl acetate; FH, fraction of hexane; GLB, glibenclamide; HCTZ, hydrochlorothiazide; MABP, mean arterial blood pressure; MCW, made with cold water; ME, methanolic extracts; MHW, made with hot water; MPO, myeloperoxidase; NA, not applicable; OGTT, oral glucose tolerance test; PFC, plaque forming cells; RBC, red blood cells; ROS, reactive oxygen species; SNP, sodium nitroprusside; SOD, superoxide dismutase.

SCHEME 7Anticancer activity assays.

**Figure:**
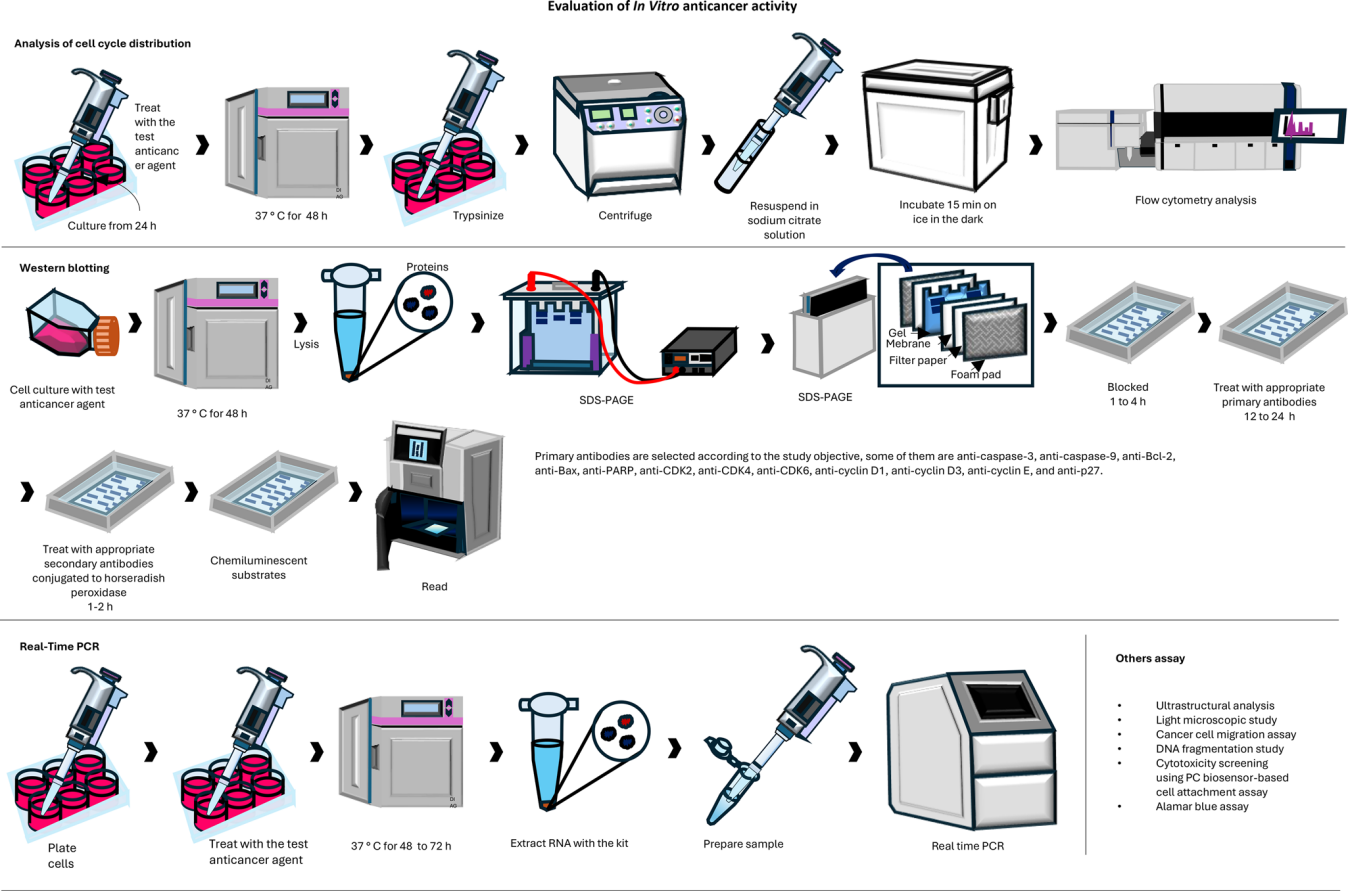

**Figure:**
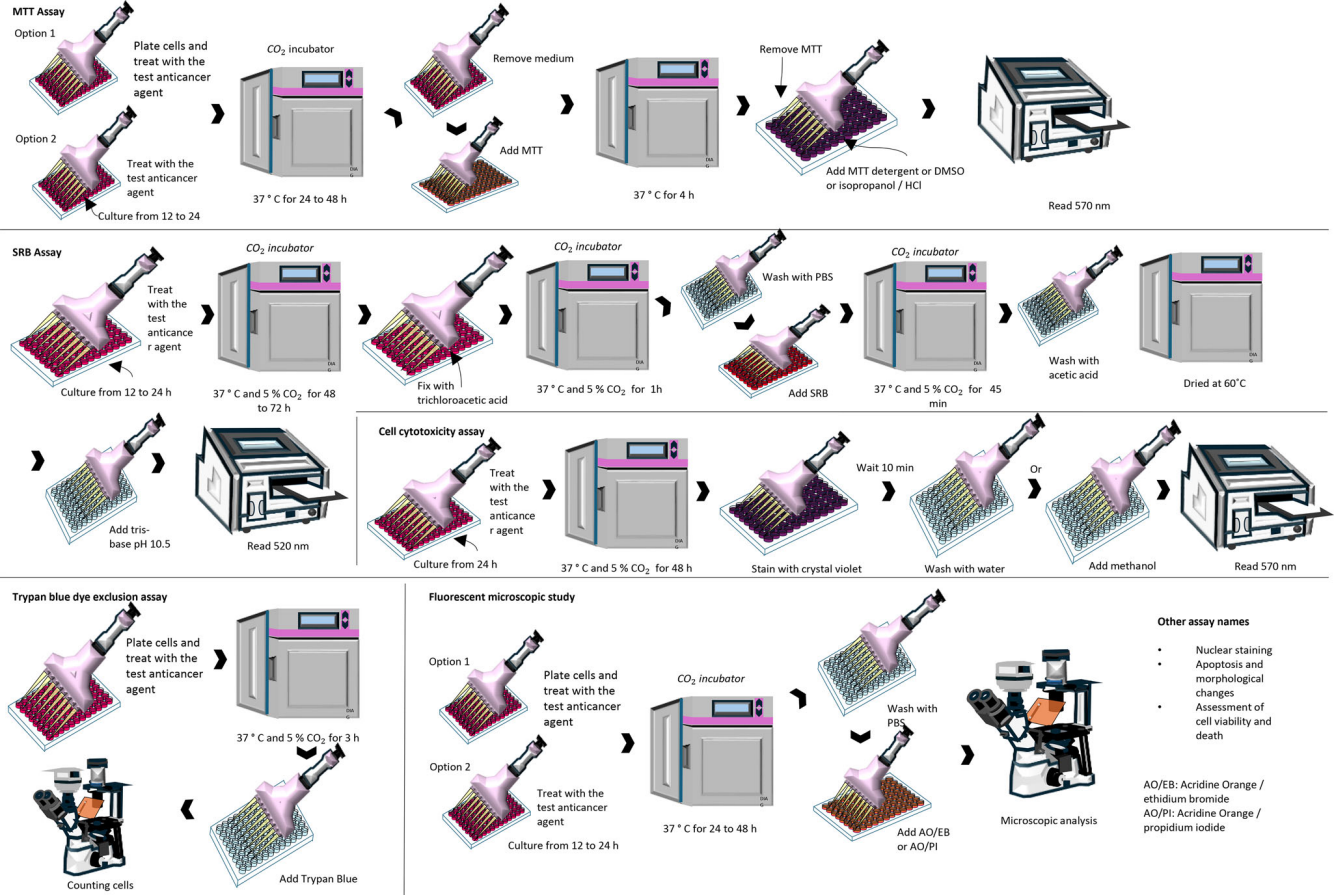

### 5.1 Antimicrobial Activity

Infections caused by fungi and bacteria are responsible for the development of serious diseases and over fifty thousand deaths per year [96]. Additionally, many microorganisms have developed resistance to existing drugs, posing a risk to public health and presenting a challenge for the pharmaceutical and healthcare industries, with economic implications [11, 14, 89, 129, 223].

In addition to the development of drug resistance by microorganisms, developing and underdeveloped countries lack access to medications to treat infections, leading to increased mortality rates. Therefore, the search for new broad‐spectrum antimicrobials with low toxicity derived from natural sources is necessary [14, 89, 223].

To date, 46 articles have evaluated the antimicrobial activity of members of 5 genera of this subfamily. Twenty‐seven articles report the presence or absence of antibacterial activity, evaluating a total of 98 bacterial strains; 14 articles discuss antifungal potential, evaluating 19 yeast strains and 8 fungal strains; 6 articles report on antiparasitic activity against *T. cruzi* and *L. amazonensis*; and 2 articles discuss antiviral potential of *A. philoxeroides*, evaluating its activity against 13 viruses. These studies examined the potential of approximately 46 extracts, 16 fractions, 6 mixtures of compounds, and 73 isolated compounds (**6, 7, 9, 10, 17, 20, 24, 25, 33, 34, 39, 40, 43, 50, 51, 54, 58, 60, 70, 71, 72, 80, 88, 92, 93, 94, 95, 100, 101, 106, 107, 108, 109, 156, 159, 226, 238, 274, 275, 298, 299, 300, 301, 304, 305, 309, 310, 311, 313, 324, 325, 327, 337, 338, 339, 340, 341, 342, 343, 344, 345, 346, 354, 356, 357, 358, 359, 360, 361, 411, 416, 417, 449**). These studies cover only 3.54% of the Gomphrenoideae members (*A. bettzickiana*, *A. brasiliana*, *A. caracasana* HBK, *A. dentata*, *A. littoralis* P. Beauv., *A. philoxeroides*, *A. pungens*, *A. repens*, *A. sessilis* (Linn.), *A. tenella* Colla, *B. portulacoides*, *G. agrestis*, *G. boliviana*, *G. celosioides*, *G. globosa*, *G. martiana*, *H. eriantha*, *I. herbstii*, *P. glomerata*, and *P. paniculata*). Schemes 4 and 5 summarize the main methodologies, and Table S1 presents the results obtained.

Kumar and his research group are currently the only ones who have studied the antibacterial activity of *Alternanthera dentata*, demonstrating that AgNPs of AqE of leaves are active against the four microorganisms evaluated [225]. Research by Zavala and collaborators showed that the ME of *A. repens* was not active against bacteria and yeasts [216].

Bhattacherjee and his research group are currently the only ones who have studied the antibacterial activity of *A. philoxeroides*. They demonstrated that fraction X of leaf ME exhibits remarkable activity, as it was able to inhibit *Escherichia coli* and *Micrococcus luteus* with relatively small MICs (11.23 ± 0.11 and 16.23 ± 0.23 µg/mL, respectively) and large ZI, when compared with the results obtained for the AuNPs of AqE from *A. bettzickiana*, the subfractions of FEaMc and extracts of *A. brasiliana*, ME of *A. sessilis*, EE and PEE from *G. boliviana*, extracts from *G. celosioides*, AcE from *G. globosa*, compounds (**20**, **24**, **25**, **34, 43**, **72**, **88**, **410**, **411**), and mixtures of compounds (**411** and **386**), (**396** and **411**), (**415** and **416**), (**394** and **414**) [26, 27, 32, 75, 82, 89, 142, 141, 173, 226].

*A. sessilis* was studied by four research groups, three of which used nanoparticles. Niraimathi et al. and Kabeerdass et al. demonstrated that the AgNPs of AqE of leaves are active against bacteria, especially gram‐negative strains [60, 227]. Venkatraman and collaborators showed that the ZnONPs of leaves possess antibacterial activity and suggested that the mechanism of action involves membrane destruction, leading to metabolic dysfunction and protein excretion by the bacteria [228]. Ullah and collaborators reported similar results, demonstrating that the ME of the whole plant presented activity against 10 of the 12 microorganisms evaluated [226].

*G. celosioides* was studied by five research groups. Moura's results for *E. coli* differed from those obtained by some authors; he reported that the EE of aerial parts and compounds **156** and **417** did not show activity against this strain. However, Dosumu and Omokhua‐Uyi reported that this strain was sensitive to AcE, EaE, and compound **449** [82, 89, 151]. Another interesting discovery was made by Dosumu, who concludes that the higher activity observed in EaE was due to synergistic relationships between molecules [82]. However, this information cannot be extrapolated, because extracts sometimes present less activity due to antagonistic relationships between molecules, as observed in the study by Sporna‐Kucab in *G. globosa* [173]. Additionally, Dosumu also reported that ME has antifungal activity similar to that of tioconazole [82]. This finding is particularly relevant because there are currently very few antifungal agents available on the market, highlighting the need for new broad‐spectrum antifungals. In this context, Abalaka et al. also obtained interesting results, demonstrating that AuNPs of leaf extract exhibited activity similar to CAM and streptomycin [229].

Rahamouz Haghighi and Sharafi in 2024 published the first article of the antibacterial activity of *H. eriantha*, demonstrating that it was active against the five microorganisms evaluated, having the same effect as gentamicin on *S. aureus* [230].

Dipangar and Murugan obtained results similar to Bhattacherjee with AgNPs of AqE from leaves of *I. herbstii*, which inhibited three gram‐negative bacteria and two gram‐positive with MICs between 6.25 and 50 µg/mL [231].

*A. brasiliana* was studied by six different researchers, two of whom studied both antibacterial and antifungal activity. In summary, Johann, Andreaza, and Coutinho reported that the HeE and EE of the whole plant, the HaE of aerial parts, and the EE of leaves did not show relevant activity against the tested microorganisms [23, 26, 232]. Araújo obtained similar results when evaluating AqE of leaves, showing that the extract did not show activity against four microorganisms, had clinically irrelevant activity against four microorganisms, and showed activity only against *Mycobacterium smegmatis* (MIC: 15.6 µg/mL) and *C. albicans* (MIC: 31.2 µg/mL) [27]. These results are consistent with those obtained by Marchete in 2021, who indicated that the HaE of the leaves has weak activity against *P. aeruginosa*, *S. aureus* ATCC 25923, oxacillin‐resistant *S. aureus*, and *E. coli* ATCC 25922 [193]. In contrast, Trapp reported that the FEaMc (1:1) and three sub‐fractions showed activity against *B. subtilis* and *M. luteus*, and that compounds **340**, **341**, **342**, **344**, **346**, **354**, **358**, **359**, and **361** showed activity against *B. subtilis*, *M. luteus*, *E. coli*, and *P. aeruginosa* [32]. The difference in results could be attributed to the extraction methods used.

Dominguez et al. in 2022 published the only article that discusses the effect of glycolic extract of the roots of *P. paniculata* on mixed‐species biofilms of *C. albicans* and *S. mutans*, *S. aureus*, or *P. aeruginosa*. Their main results reported biofilm inhibition to varying degrees [233].

Another important discovery was made by the research groups of Nagalingman and Andreazza, who concluded that the effect of the extract on microorganisms can be potentiated when used with drug delivery systems such as nanoparticles or in combination with other methods such as photosensitization [21, 23].

To date, the trypanocidal and leishmanicidal effects have been evaluated in *A. littoralis* Beauv. ex Moq., *B. portulacoides*, *G. agrestis*, and *P. glomerata*, all of which showed antiparasitic activity [2, 8, 117, 151, 177, 234]. The greatest activity was observed in compound **324** isolated from *A. littoralis*, with IC_50_ values of 0.23 and 0.16 mM for *T. cruzi* and *L. amazonensis*, respectively. Additionally, it should be noted that compounds **226** and FH from the HaE of *P. glomerata* showed high activity against *T. cruzi*, with IC_50_ values of 44.78 and 47.86 µg/mL, respectively [8, 177].

Antiviral activity has only been evaluated in vitro and in vivo for *A. philoxeroides*, and the results were promising, as that compound **238** showed in vitro activity against HSV‐1, HSV‐2, human cytomegalovirus, measles virus, and mumps virus. In a genital herpes model in mice (in vivo) caused by HSV‐2, compound **238** was effective, indicating that this compound could be a candidate for an anti‐herpes agent. Additionally, compounds **59, 40**, and **60** significantly blocked the secretion of HBsAg in HepG2.2.15 cells [40, 41].

### 5.2 Antioxidant Activity

Antioxidant compounds play a crucial role in protecting the human body against free radicals, including reactive oxygen species (ROS), which can lead to various pathological conditions. These conditions include Alzheimer's disease, anemia, arthritis, asthma, atherosclerosis, cancer, cataracts, liver cirrhosis, diabetes, neurological disorders, Parkinson's disease, cardiovascular diseases, hypertension, hypotension, ischemia, inflammation, Down syndrome, neurodegeneration, and the aging process. Therefore, antioxidant compounds are important in the treatment and prevention of these diseases [14, 77, 151, 224].

In recent years, there has been a growing interest in the antioxidant potential of plants. This interest persists despite the availability of synthetic drugs on the market, as concerns about their safety and toxicity continue to be significant.

To date, 32 articles have discussed the antioxidant potential of the Gomphrenoideae subfamily members, with 2 studies conducted using in vivo models. These studies described the antioxidant potential of 3.54% of the subfamily members (*A. bettzickiana*, *A. brasiliana*, *Alternanthera flavescens*, *A. littoralis* P. Beauv., *A. paronychioides*, *A. philoxeroides*, *A. pungens*, *A. sessilis* (Linn.), *A. tenella* Colla, *G. celosioides*, *G. globosa*, *Gomphrena haageana* K, *I. angustifolia*, *I. herbstii*, *P. glomerata*, *P. paniculata*, *P. townsendii*), evaluating 47 extracts, 22 fractions, and 14 compounds (**24, 25, 34, 43, 50, 67, 70, 88, 106, 129, 274–275, 324, 325**). Additionally, two extracts contained in nanoparticles, a green leaf juice, a commercial preparation, and a flower infusion were assessed. Scheme 6 summarizes the main methodologies, and Table S2 summarizes the results obtained.

*A. brasiliana* was evaluated by five different research groups. Pereira et al. evaluated the antioxidant potential of EE, BuF, DF, and FEA using the DPPH assay, but only FEA exhibited radical scavenging activity, which was dose‐dependent [235]. Marchete et al. showed that the HaE of leaves has antioxidant activity, evidenced by an increase in the scavenging capacity of DPPH, FRAP, and ABTS free radicals [193]. Paliwal et al. obtained similar results to Marchete et al., showing that HaE of leaves had higher activity than ClE and ME of leaves [236].

The antioxidant potential of *A. philoxeroides* was evaluated by five different research groups. In general, fraction X of the ME of leaves showed better activity against DPPH and ABTS radicals compared to the HdE of the tender stem, shoots, and leaves, as well as the EE of the whole plant. The IC_50_ values were 33.94 ± 3.45 µg/mL for DPPH and 60.76 ± 4.31 µg/mL for ABTS [132, 135, 136, 141, 237].

Two research groups demonstrated that the antioxidant potential of *A. pungens* was low [16, 238].

The antioxidant potential of *A. sessilis* was evaluated by eight different authors. The best results for DPPH activity were obtained with the FEA of the ME of leaves (EC_50_: 10.81 ± 0.29 µg/mL), followed by the CF of the ME of callus (EC_50_: 34.12 ± 0.67 µg/mL), ME of aerial parts (IC_50_: 35.39 µg/mL), BuF of the ME of leaves (EC_50_: 35.71 ± 1.24 µg/mL), AF of the ME leaves (EC_50_: 35.96 ± 1.28 µg/mL), FEA of the ME of callus (EC_50_: 43.87 ± 0.39 µg/mL), and BuF of the ME of callus (EC_50_: 57.11 ± 0.13 µg/mL). The other extracts evaluated showed IC_5O_, EC_50_, or SC_50_ values greater than 80 µg/mL [49, 55, 57, 60, 195, 196, 238]. Additionally, Mohd Hazli and Muniandy evaluated the EE of the stem of *A. sessilis*, obtaining IC_50_ values of >1000 µg/mL and 782 ± 29.9 µg/mL, respectively [55, 57]. Despite the similarity in the results, discrepancies were observed that could be due to differences in the growth conditions of each plant.

The ethanolic extract of *G. celosioides* has been studied in vitro and in vivo. According to the in vitro study, the EE of leaves eliminates the DPPH radicals more efficiently than Trolox. Additionally, in the in vivo rat model, it is capable of reducing TBARS levels and increasing the total antioxidant ability in serum [77, 194, 239].

The antioxidant potential of *G. globosa* has been studied by four research groups. The extracts, commercial preparations, and floral infusions demonstrated antioxidant activity in various assays, including DPPH, FRAP, TBARS, and radical scavenging activities involving O_2_^−^ and NO. However, they exhibited high values in EC_50_ and IC_50_, in most cases exceeding 500 µg/mL [73, 98, 150, 153].

Dipankar and Murugan, in their study, evaluated the antioxidant potential of EE of *I. herbstii* and AgNPs synthesized using the AqE of the leaves, concluding that the NPs potentially enhance the antioxidant activity of the extract [231].

Regarding *H. eriantha* (Poir.) Pedersen, it has been reported to have low antioxidant potential [215]. However, in that publication, the plant is cited as *P. paniculata*, which is a synonym of *H. eriantha* according to GBIF (https://www.gbif.org/es/species/101306355).

In 2018, Corrêa et al. conducted an analysis on various extracts and compounds derived from the whole plant of *P. townsendii* Pedersen. The study encompassed hexanoic and ethanolic extracts, as well as the hexane, dichloromethane, and hydroalcoholic phases. Additionally, the research included an examination of compounds **67** and **106**, both individually and in combination. The results demonstrated that the mixture of compounds **67** and **106** exhibited the highest DPPH radical scavenging activity, with an EC_50_ value of 3.7 µg/mL. Individually, compound **67** showed significant activity with an EC_50_ of 4.9 µg/mL, whereas compound **106** alone had an EC_50_ of 83.2 ± 2 µg/mL. Remarkably, when compound **106** was combined with compound **67**, there was a 95.55% reduction in the EC_50_ value, indicating an enhanced activity likely stemming from a synergistic interaction between the two compounds. Notably, the HeE and the hexane phase both displayed the lowest DPPH activity, with EC_50_ values exceeding 200 µg/mL [127].

### 5.3 Anticancer Activity

Cancer is one of the main causes of morbidity and mortality worldwide, characterized by irregular cell growth triggered by genetic or environment stimuli. Currently, chemotherapy remains a primary treatment option; however, it often leads to adverse effects, including the development of cancer cell lines resistant to multiple medications, leading to chemotherapy failure. Therefore, one strategy to combat cancer is the search for bioactive compounds with antiproliferative and antitumoral activities [131, 240].

Currently, 33 articles discuss the in vivo and in vitro anticancer potential of 28 extracts, 16 fractions, 5 NPs, and 22 isolated compounds (**35, 36, 243, 244, 245, 246, 247, 255, 256, 257, 258, 259, 260, 261, 262, 263, 264, 269, 270, 455, 456, 457**), 3 mixtures of active molecules, and a paste of leaves and plant powder obtained from 3.13% of the members of Gomphrenoideae (*A. bettzickiana*, *A. brasiliana*, *A. flavescens*, *A. philoxeroides*, *A. sessilis*, *A. tenella*, *G. celosioides*, *G. elegans* mart., *G. globosa*, *G. macrocephala*, *G. martiana*, *H. erianthos* (Synm. *Hydrangea paniculata*, Synm. *P. paniculata*), *I. diffusa*, *I. herbstii*). Ten articles discuss their activity in vivo models, and 26 discuss their activity in vitro models. Scheme 7 summarizes the main methodologies, and Table 10 and Table S3 summarize the results obtained.

In vitro activity was evaluated across a range of cancer cell lines. These included four leukemia cell lines (HL60, MT‐1, MT‐2, MK‐1); four colon cancer cell lines (Caco‐2, HT‐29, HCT‐8, HCT116); three pancreatic cancer cell lines (Panc‐1, MIA PaCa‐2, Capan‐1); two prostate cancer cell lines (PC3, Human LNCaP); two breast cancer cell lines (MCF‐7 cell, MDA‐MB‐435); two cervical cancer lines (HeLa, KB); two cholangiocarcinoma cells (KKU‐100, KKU‐213); two skin cancer cell lines (B16F10, B‐16); one line of lung cancer (A549); one Ehrlich ascites carcinoma cell line (EAC cell); one liver cancer cell line (Hep‐G2); one human neuroblastoma cell line (SK‐N‐SH); a cell line glioblastoma (SF‐295); and one oral cavity carcinoma cell line (HSC‐2 cells).

The ME of aerial parts of *A. brasiliana* and *A. flavescens* Kunth. were evaluated against Caco‐2 and HT‐29 cell lines. In both cell lines, the extract of *A. brasiliana* was more effective, with the lowest IC_50_ values: 252.9 ± 5.7 µg/mL for Caco‐2 and 160.3 ± 8.5 µg/mL for HT‐29 [31]. Regarding HCT‐8, it was evaluated only in *G. elegans*, and it was found that HeE from leaves caused the highest percentage of lethality (101.16%), followed by FnH (%*L*: 100), AqE, and FnB from leaves with 99.68% lethality, and ClE with %*L* of 79.08%. The other 12 extracts and 8 fractions showed a lethality percentage lower than 30% [156, 241]. The HCT11‐6 cell line was evaluated only with the ME of roots of *H. paniculata*, which generated a decline in cell viability [230]. These findings suggest that these four plants exhibit cytotoxic activity against colon cancer cell lines.

The ME of the aerial parts of *A. brasiliana* and *A. flavescens*, and the pfaffosidic fraction of *H. paniculata* (Syn. *H. eriantha* (Poir.) Pedersen), were evaluated against the Hep‐G2 cell line. It was shown that both the extracts and the fraction exhibited activity against this cell line, with the pfaffosidic fraction presenting the highest activity, reducing more than 50% of the cells at a concentration of 100 µg/mL [31, 121].

Five compounds isolated from *A. philoxeroides*, AuNPs from the AqE of *A. sessilis* leaves, the EE from the whole *A. sessilis* plant, AgNPs of the AqE of *I. herbstii* leaves, and the ME of the whole *G. globosa* plant were evaluated against HeLa cells. The results indicated that compounds **455**, **456**, **457,** and **35** isolated from *A. philoxeroides* exhibited the highest activity against this cell line, followed by AgNPs of *I. herbstii*. On the other hand, the EE of *A. sessilis* showed low activity, and *G. globosa* showed no activity [37, 62, 231, 242].

The ME of the whole *G. globosa* plant was evaluated against leukemia cell lines MT‐1, MT‐2, and MK‐1, and the isolated compounds from *A. philoxeroides* (**261–264**) were tested against HL60 cell line. The results showed that *G. globosa* exhibited no cytotoxic activity, whereas all the compounds from *A. philoxeroides* were active against the HL60 cell line. Compound **264** presented the highest activity with an IC_50_ value of 45.93 µg/mL, whereas compound **263** had the lowest activity, with an IC_50_ of 271.45 µg/mL [37, 243].

Only one study has evaluated the potential of this subfamily against pancreatic cancer cell lines, obtaining remarkable results. Overall, the ME, PEF, and CF of *A. sessilis* leaves at a concentration of 100 µg/mL reduced survival percentages to less than 2% in Panc‐1 cells. Among these, CF presented the highest activity, and the authors also reported that it obtained IC_50_ values of 13.08 ± 10.40 µg/mL for MIA PaCa‐2 and 34.92 ± 2.20 µg/mL for Capan‐1 [244].

The isolated compounds from *I. diffuse* (**269** and **270**) were tested against the PC3 and human LNCaP prostate cancer cell lines, and the AgNPs from AqE of the leaves of *A. sessilis* were tested against the PC3 line. It was found that compounds **269** and **270** showed activity against LNCaP cells. In contrast, the AgNPs of *A. sessilis* showed remarkable activity inhibiting 94.11% of PC3 cells at 25 µg/mL [165, 245].

Currently, seven studies have been conducted to find compounds with cytotoxic activity against breast cancer cell lines. In summary, it was found that MCF‐7 cells are sensitive to the AgNPs (IC_50_: 3.043 µL/mL and 99% of inhibition at 25 µg/mL) and ZnONPs (IC_50_: 210 µg/mL) of *A. sessilis*, but not to the AuNPs of this same plant. In this cell line, the AgNPs of *A. tenella* also showed activity with an IC_50_ of 42.5 µg/mL. Additionally, the BE of *P. paniculata* showed cytotoxic activity [70, 124, 184, 228, 246]. Regarding the MDA‐MB‐435 cell line, different extracts and fractions of *G. elegans* Mart. were evaluated. It was shown that AqE and FnB at a concentration of 100 µg/mL generated a lethality percentage of 97.39, whereas HeE and FnH reached lethality percentages of 96.35. However, the other 13 extracts and 8 fractions at this same concentration obtained lethality percentages lower than 50% [155, 241].

Two studies have evaluated the cytotoxic potential against skin cancer cell lines. In this context, the compounds **255**, **256**, **257**, **258**, **259**, and **260**, isolated from *P. paniculata*, were tested against the B‐16 cell line. Compound **260** presented the highest activity with an IC_50_ of 30 µg/mL, and the compound with the lowest activity was **259**, with an IC_50_ of 120 µg/mL [125, 164]. In contrast, the ME of the whole plant of *G. globosa* was inactive against B16F10 [247].

A total of 6 of the 10 in vivo studies aimed to find a bioactive substance for treating Ehrlich carcinoma. Remarkably, the EaE of the leaves of *A. brasiliana*, the AqE of the aerial parts of *A. tenella* Colla, the BuF from *P. paniculata* root, and 20 and 60 mg/kg of the mixture of flavonoids (**6, 7, 10, 17**) isolated from *G. martiana* increased the survival of mice with EAC. However, only the extract of *A. brasiliana* and *A. tenella* reduced viable cells. Additionally, the mixture of flavonoids inhibited tumor formation by 32%, and the root powder of *P. paniculata* and the EaE of *A. brasiliana* reduced the volume of EAC [66, 100, 185, 207]. It should also be noted that Pinello et al. [210] evaluated the anticancer potential of the ME of *P. paniculata* root, focusing on macrophage activity. They found that the ME increased peritoneal macrophages and phagocytosis, suggesting that the anticancer activity of *P. paniculata* may result from the stimulation of macrophages, natural killer cells, and cytotoxic T lymphocytes [210]. In conclusion, the extracts of *A. brasiliana* and *A. tenella* have antitumor activity, the mixture of compounds has moderate cytotoxic activity, and the root powder and BuF of *P. paniculata* have an antineoplastic effect.

Da Silva et al. [208, 211] demonstrated that *P. paniculata* root powder has anti‐hepatocarcinogenic properties, reducing liver lesions and adenoma in mice. The chemopreventive effect is attributed to the inhibition of cell proliferation and an increase in apoptotic processes [208, 211]. However, *A. sessilis* exhibited no activity against squamous cell adenocarcinoma of the stomach in mice [206].

### 5.4 Analgesic and Antinociceptive Activity

Pain, a sensory perception, often represents the primary symptom in the diagnosis of various diseases. Considered a global public health issue, the search for treatments to alleviate or control pain is crucial. Among these treatments, the use of medicinal herbs and their compounds stands out [190, 191, 248, 249].

It should be noted that many medications commonly used to treat pain have unwanted side effects, including respiratory depression, drowsiness, decreased gastrointestinal motility, nausea, gastric ulcers, hepatotoxicity, and various disorders of the autonomic nervous and endocrine systems. Furthermore, many of them do not reduce pain in all treated individuals [191, 248, 249]. This highlights the need to search for new bioactive compounds with analgesic activity that lacks side effects.

Currently, 11 articles discuss the analgesic potential of eleven extracts and 2 isolated compounds from 7 members of the Gomphrenoideae subfamily. The results are described in Table 10. In the acetic acid‐induced abdominal contractions assay, extracts from *A. brasiliana*, *A. philoxeroides, A. sessilis, G. celosioides*, and *P. glomerata* all showed a reduction in the number of contractions. Notably, the AqE of *A. brasiliana* and the ME of *A. sessilis* exhibited better activity than dipyrone and aspirin, respectively [30, 39, 87, 118, 133, 204, 205]. In the carrageenan‐induced paw edema assay, the EE of *A. maritima* (Mart.) A.St.‐Hil. (Synm. *A. littoralis* Beauv. ex Moq.), *A. tenella, B. portulacoides, G. celosioides*, and compounds **43** and **72** showed inhibition of mechanical hyperalgesia induced by carrageenan. Compound **43** achieved 100% inhibition of hyperalgesia [34, 67, 71, 78].

In the hot plate test, the EE of the whole plant of *A. sessilis* and the AqE of *G. celosioides* increased the reaction time, indicating an analgesic effect on the central nervous system. However, the HaE of the roots and rhizomes of *P. glomerata* showed no effect, indicating the absence of analgesic activity [87, 118, 133].

In the carrageenan‐induced cold allodynia test, the EE of the whole plant of *A. tenella* Colla and the EE of the aerial parts of *G. celosioides* inhibited the response to cold, with the EE of the *A. tenella* showing the highest activity. However, compound **43** did not show any effect in this test. These same extracts and compound **43** were evaluated in the zymosan‐induced articular inflammation assay, where all reduced mechanical hyperalgesia and inhibited edema [67, 78].

### 5.5 Anti‐Inflammatory Activity

Inflammatory diseases, such as asthma, rheumatoid arthritis, psoriasis, autoimmune diseases, and severe autoinflammatory diseases, develop due to the overproduction of pro‐inflammatory mediators. For this reason, their inhibition has therapeutic value in the development of anti‐inflammatory agents [73, 250].

Over time, GCs have been used to treat various inflammatory disorders characterized by their effectiveness, but their chronic use causes undesirable adverse effects, such as skin atrophy, inhibition of wound healing, osteoporosis, obesity, hyperglycemia, and glaucoma [71, 250].

It should be noted that the anti‐inflammatory compounds caffeic acid, ferulic acid, vanillic acid, and catechin have been isolated from *G. celosioides* [78].

To date, 21 studies have been carried out to evaluate the anti‐inflammatory activity of extracts, fractions, infusions, isolated compounds, and commercial preparations of 2.30% of the members of the Gomphrenoideae subfamily, with 7 studies conducted using in vitro models and fifteen using in vivo models. Tables 7 and 10 provide detailed information on these studies.

Regarding in vitro activity, extracts of *A. sessilis*, *G. celosioides*, *G. globosa*, and *Gomphrena haageana* Klotzsch have been shown to reduce NO levels [56, 73, 98, 194]. Additionally, the extract of *G. celosioides* and *A. sessilis* reduces COX‐2 expression levels [56, 194]. The EE of *A. sessilis* also reduces the viability of RAW 264.7 cells, proinflammatory cytokines, and PGE2, as well as prevents the activation of the NF‐κB pathway. However, none of the extracts of *G. celosioides* affect RAW 264.7 cell viability [194].

The in vivo anti‐inflammatory activity was evaluated in most of the studies through edema or pleuritis induced by carrageenan. The results showed that the extracts of *A. maritima* [34]*, A. tenella* Colla [213]*, B. portulacoides* [71]*, G. celosioides* [77, 87]*, P. glomerata* [118, 119], and *P. townsendii* [127], as well as compound **72** isolated from *A. maritima* [34], and compounds **67** and **106** isolated from *P. townsendii* [127], exhibited inhibition of edema formation. The best activity was observed in compounds **67** and **106**, which at a concentration of 1 mg/kg, were able to inhibit edema by 75.4% ± 4.0% and 73.00% ± 4.0%, respectively [127]. This was followed by the activity of the HaE of *P. glomerata*, which showed an ID_50_ of 20.4 mg/kg for the intraperitoneal dose and an ID_50_ of 60.5 mg/kg for the oral dose [119]. The least efficient extract was that of *G. celosioides*, which, at a concentration of 400 mg/kg, only achieved a 39.62% inhibition of edema [87].

Regarding the anti‐inflammatory activity in the carrageenan‐induced pleuritis model, it was observed that the extracts of *A. tenella* Colla [67]*, B. portulacoides* [71]*, P. townsendii* [127], compound **72** isolated from *A. maritima* [34], and compounds **67** and **106** isolated from *P. townsendii* [127] reduced leukocyte migration to the pleura. Compound **72** showed the greatest inhibition at a concentration of 10 mg/kg (%*I*: 77) [34]. Compounds **67** and **106** also showed high activity, with an inhibition of 50.7% ± 1.03% and 59.4% ± 1.25%, respectively, at a concentration of 1 mg/kg [127]. In this test, it was also observed that compound **72** and the extract of *B. portulacoides* reduced protein extravasation [34, 71]. The EE of *A. maritima* [34] and *A. tenella* Colla [67], as well as compound **43** [67], reduced the number of leukocytes, with the extract of *A. maritima* showing the best activity (%*I*: 68) [34, 67]. Additionally, the AqE of *A. brasiliana* reduced the number of lymphocytes, polymorphonuclear cells, and exudate [30]. These results suggest that both the extracts and the isolated compounds have anti‐inflammatory activity.

It should be noted that the EE of leaves from *A. brasiliana* also reduced the formalin‐induced edematogenic process [26]. The EE of the whole plant of *A. tenella* Colla and compound **43** inhibited the formation of zymosan‐induced edema [67]. The EE of *B. portulacoides* inhibited *Bothrops jararacussu* venom, BthTX‐I, and BthTX‐II‐induced edema formation but did not affect leukocyte migration [214]. HaE from roots of *P. glomerata* reduced edema induced by bradykinin, substance P, histamine, serotonin, and LPS [119], and the ME of *P. paniculata* roots reduced colonic and intestinal inflammation, the latter being related to the modulation of the expression and production of MAPKs and mucin [120, 215].

### 5.6 Antidiabetic Activity and Antihyperglycemic Activity

Diabetes is a disease that is spreading rapidly throughout the world, and every year the number of people suffering from this disease increases. In 2016, the WHO reported that 400 million people were affected by diabetic disorder, and in 2017, the IDF reported that 425 million people had diabetes mellitus. It is estimated that by 2045, the number of people with diabetes will rise to 629 million. Additionally, diabetes can lead to the development of other diseases, including cardiovascular, kidney, and eye diseases, as well as stroke and lower limb amputation [15, 204].

Currently, diabetes can be treated with insulin or hypoglycemic agents, but both can cause side effects such as hypoglycemia, weight gain, gastrointestinal upset, nausea, diarrhea, liver dysfunction, jaundice, and heart failure [15].

It should be noted that around 400 plant species and their metabolites are used to treat diabetes mellitus worldwide. The antioxidant potential of medicinal plants is a key factor in reducing the incidence of diabetic complications [15].

Currently, eight studies have evaluated the antidiabetic and/or antihyperglycemic potential of five extracts, fourteen fractions, five compounds, and a green juice, representing 0.83% of the members of the Gomphrenoideae subfamily (Tables 8 and 10).

In the in vitro studies, the inhibition of α‐glucosidase was evaluated for fraction X of *A. philoxeroides*, the green leaf juice of *A. sessilis*, and eight fractions of the latter plant. The results demonstrated that all the evaluated substances had α‐glucosidase inhibitory activity. The most potent activity was observed in fraction X of *A. philoxeroides* (IC_50_: 52.41 ± 5.22 µg/mL), whereas the weakest activity was found in the FH of the ME of *A. sessilis* leaves (EC_50_: 6.31 ± 1.70 mg/mL) [141, 195, 196]. It was also described that *A. paronychioides* has a preventive action on diabetic glucotoxicity [35], and among the five compounds isolated from *G. celosioides* (**162**, **166**, **187**, **190**, **271**), compound **190** showed a significant improvement in glucose uptake and the highest inhibitory effect on PTP1B, indicating its potential for use in the prevention and treatment of Type 2 diabetes [84].

As for the in vivo studies, they were generally analyzed using the OGTT. Notably, the ME of the entire *A. philoxeroides* plant [39], the ME of aerial parts of *A. sessilis* [204], and the FEA of the EE from the aerial parts of *A. sessilis* [212] reduced glucose levels in a manner comparable to GLB. It should also be noted that neither the green juice nor HF and FA of *A. sessilis* reduced glucose levels [195, 212].

### 5.7 Hepatoprotective Activity

The liver is responsible for regulating several important metabolic functions, and injury to this organ can disrupt these functions [251]. Liver damage typically involves oxidative stress and is characterized by a progressive evolution from steatosis to chronic hepatitis, fibrosis, and cirrhosis [252].

Currently, liver diseases are treated with corticosteroids, vaccines, and antivirals. However, these treatments often cause side effects, particularly with chronic or subchronic use. This highlights the necessity of finding new phytotherapeutic drugs that are both safe and more effective [252].

To date, only four in vivo studies on the hepatoprotective activity of members of this subfamily have been conducted (Table 10). The HaE from the leaves of *A. brasiliana* L. [236], the ME from the entire *A. sessilis* plant [51], the AqE from the stems and leaves of *G. celosioides* [90], and AqE from *G. globosa* L. [218]. All studies showed an improvement in the antioxidant profile and a reduction in liver damage [51, 90].

### 5.8 Other Activities

Other activities have been studied in members of the Gomphrenoideae subfamily, as shown in Tables 9 and 10. An analysis of some of these activities will be presented below.

#### 5.8.1 Neurological Activity

To date, 14 investigations have been carried out to evaluate the potential of fourteen extracts, 10 fractions, 6 compounds, and an infusion of aerial parts of 7 members of this subfamily. Among the isolated compounds of *A. philoxeroides* (**5, 35, 36, 41, 79**), compound **35** exhibited the highest inhibitory activity against MAO‐A (IC_50_: 0.00046 ± 0.04 µM), higher than that of Clorgyline. Compound **36** showed the highest inhibitory activity against MAO‐B (IC_50_: 0.00022 ± 0.12 µM) and inhibited the formation of toxic Aβ plaques (%*I*: 81.96 ± 2.14), exhibiting greater activity than curcumin in the latter. It should be noted that all these compounds demonstrated greater inhibition of MAO‐A and MAO‐B than the EE of this plant. These results are significant because monoamine oxidases are enzymes related to cognitive dysfunction and depression. Therefore, it can be suggested that these flavonoids have antidepressant activity. Furthermore, these five flavonoids play an important role in the search for anti‐Alzheimer's compounds, since preventing Aβ aggregation is one of the objectives for the development of therapeutic strategies. In general, these flavone derivatives exhibit an antidementia effect [38, 135]. Additionally, the EE of the entire *A. philoxeroides* plant was evaluated in an in vivo model, showing that it improves recognition, spatial working, and reference memory in ovariectomized mice, indicating its potential to prevent senile dementia [135].

The inhibition of AChE and BChE was also evaluated in the EE of *A. philoxeroides*, but this extract did not exert a significant effect on these neurotransmitters [135]. A similar result was previously obtained by Silva and collaborators, who evaluated two extracts (AqE and EB) and a commercial preparation of inflorescences of *G. globosa* L. and found that none of these substances were capable of inhibiting AChE [98].

Kim (2019) conducted an in vivo and in vitro study to determine whether the EE of the aerial parts of *Iresine celosia* L. (Syn. of *I. diffusa* Humb. & Bonpl. ex Willd.) had an anti‐neuroinflammatory effect. In the in vitro study, the extract reduced cytokine levels and inflammatory mediators in the microglia, partly due to the inhibition of the MAPKs/NF‐κB signaling pathway. In the in vivo study, the researchers concluded that the EE improves behavioral dysfunctions caused by neuroinflammation in mice. These results suggest that the extract is a potential therapeutic agent for treating neuroinflammation associated with neurodegenerative diseases such as Parkinson's, Alzheimer's, and Huntington's [104].

The aqueous and methanol extracts of *I. herbstii* were shown to affect the CNS by interacting with dopamine and serotonin receptors, suggesting their potential for treating diseases such as Parkinson's and schizophrenia [108, 201]. Regarding *A. brasiliana*, researchers found that the infusion of its aerial parts increases exploratory activity but has no effect on anxiety [30]. Meanwhile, the ME exhibited anxiolytic, sedative, and anticonvulsant effects [24]. In 2014, Mondal reported that the EE of *A. sessilis* has central stimulant activity [133]. It is also worth noting that *P. glomerata* was studied by three different research groups, which found that the EE of its roots and rhizomes did not have robust effects on depression and anxiety [219]. The HaE of the root exhibited stimulating effects, improved the acquisition and retention of behaviors, and partially reversed age‐related memory deficits [116]. Finally, the HF of ME of root was found to reduce stress and depressive behaviors and protect against anxiety development, possibly by maintaining antioxidant defenses and reducing oxidative damage [114, 219].

#### 5.8.2 Gastrointestinal Activity

Eight articles are currently discussed the gastrointestinal activity of 14 extracts and 8 fractions of 5 members of the Gomphrenoideae subfamily. It has been reported that the aqueous, methanolic, and hexanolic extracts of *A. repens* (Syn. *A. sessilis* (L.) R.Br. ex DC. and *A. pungens* Kunth), as well as six fractions of the ME, exhibit spasmolytic activity according to in vitro studies [216]. The AqE, EE, and ME of this plant also demonstrate antidiarrheal activity in vivo models [46, 47, 216]. Similarly, Saquib and Janbaz reported that the EE of *A. sessilis* has spasmolytic activity based on in vitro study results [64]. Additionally, it was reported that ME from the leaves of *G. celosioides* has an antiulcerogenic effect (both preventive and curative), which is likely related to its antioxidant activity [88, 217]. The HaE of *Guilleminea densa* (Humb. & Bonpl. ex Schult.) Moq. leaves have been shown to have a gastroprotective effect. It can inhibit gastric lesions induced by indomethacin, ethanol, cold immobilization, or stress. Additionally, it increases gastric mucus, demonstrating an enhancing effect on the protective mucosal barrier. Finally, it inhibits the presence of ulcers, erosive gastritis, acute inflammation infiltration, and focal hemorrhage [102]. Freitas reported in 2004 that the AqE from roots and rhizomes of *P. glomerata* exerts a protective effect on the gastric mucosa [159].

#### 5.8.3 Wound‐Healing Activity

To date, there are only six articles discussing the wound‐healing potential of members of the Gomphrenoideae subfamily. Four of these studies focused on the healing potential of the ME of *A. brasiliana* leaves by the Barua research group. Among the most relevant results obtained by this group is that the ME has a positive effect on wound contraction, fibroblast deposition, and angiogenesis. It also has pro‐healing activity in burn wounds, reducing the wound area more efficiently than Himax. Additionally, it increases endogenous antioxidant activity and angiogenesis. Similarly, it was shown that ME maintains its healing potential in wounds of old or immunocompromised mice [202, 220, 221, 222]. Additionally, the healing potential of the EE of *A. sessilis* stems was studied in an in vitro model, revealing that the EE can enhance the progression of wound closure in normal and diabetic fibroblast cells, as well as in keratinocytes. This suggests that it has the potential to be used in late healing stages of diabetic patients [57].

## 6 Toxicology of Gomphrenoideae

Members of the Gomphrenoideae subfamily are widely used in traditional medicine to treat different ailments. To date, only 37 articles have reported the toxicity of 2.35% of the members of this subfamily (Table 11). It has been reported that the *n*HE and HE of *A. bettzickiana* possess mutagenic activity and toxicity against the BHK‐21 cell line [17]. Additionally, the AuNPs of AqE of leaves of this same plant have shown toxicity against zebrafish embryos at concentrations higher than 25 µM [21]. Regarding *A. brasiliana*, it was found that the ME and EaE of leaves do not present toxicity in mice at the maximum dose evaluated (5 and 2 g/kg, respectively) [24, 185, 202, 220, 221, 222]. The HaE of the aerial parts did not present toxicity against murine macrophages at the evaluated concentration (20 µg/mL) [193], but the EE of leaves showed low toxicity against flies [26], and the AqE of leaves showed an LC_50_ of 500 µg/mL for *Artemia salina* [27]. The ME of *A. philoxeroides* did not show toxicity [39], whereas the AqE and EE extracts of the aerial parts of *A. repens* showed toxicity against mice (LD_50_: 3.4782 and 4.0639, respectively) [46]. On the contrary, the ME of the aerial parts and the EE of the stem of *A. sessilis* did not show toxicity up to the doses evaluated in an in vivo and in vitro model, respectively [57, 204].

**TABLE 11:** Cytotoxicity of the Gomphrenoideae subfamily.

Species	Extract(s)/Compounds	Assay method	Model	Dose	Positive control	Activity	References
*Alternanthera bettzickiana*	*n*HE of whole plant	MTT assays	BHK‐21	Uninformed	—	IC_50_: 493 µg/mL for HE and IC_50_: 456 µg/mL for *n*HE. Cytotoxic activity is dependent on concentration. *n*HE exhibited greater activity than HE Both extracts have dose‐dependent mutagenic potential. The enzyme activation system ↑ the mutagenicity in HE, whereas ↓ it in *n*HE *n*HE: MIx_150 mg/mL_: 33.7 and 45.29 for TA100 and TA102, respectively HE: MIx_15 mg/Ml_: 21.78 TA100 and MIx_150 mg/mL_: 12.30 for TA102	[17]
		Ames reverse mutation assay	*Salmonella typhimurium* TA‐100 and TA‐102	0.015, 0.15, 1.5, 15, and 150 mg/mL	Sodium Azide and H_2_O_2_		
	HE of whole plant	MTT assays	BHK‐21	Uninformed	—		
		Ames reverse mutation assay	*S. typhimurium* TA‐100 and TA‐102	0.015, 0.15, 1.5, 15, and 150 mg/mL	Sodium Azide and H_2_O_2_		
*Alternanthera bettzickiana*	AuNPs of AqE of leaves	Toxicity analysis	Zebrafish (*Danio rerio*) embryo model	12, 25, and 50 µM	—	No toxicity was observed up to 25 µM At 50 µM, it strongly inhibited hatching, affected tail formation, and dark material was observed in the intestinal tract	[21]
*Alternanthera bettzickiana*	EE of aerial parts	Acute oral toxicity study	Wistar rats	2000 mg/kg	—	No mortality was observed, but slight behavioral changes such as convulsions and tremors occurred, along with ↓ somatomotor activity. It did not affect BW or cause organ injury. No significant changes for hemoglobin, RBCS, ESR, TLC, neutrophils, HCT, MCHC, MCH, MCV, LDL, VLDL, cholesterol, HDL, triglycerides, AST, ALT, proteins, globulin, albumin, and A/G ratio. ↑ platelet count and ↓ alkaline phosphatase and protein levels. It was concluded that the extract was safe	[20]
*Alternanthera brasiliana*	ME of leaves	Acute toxicity	Albino mice	2.0 g/kg BW	—	The extract is safe up to 5 g/kg. Showed no changes in motor activity or behavior at a concentration of 2 g/kg	[202]
		Determination of LD_50_	Albino mice	Different [] up to 5 g/kg	—		
*Alternanthera brasiliana*	HaE of aerial parts	Toxicity test	Murine macrophages	20 µg/mL	—	No apparent cytotoxic effects were observed in murine macrophages	[232]
*Alternanthera brasiliana*	ME of leaves	Acute toxicity	Swiss albino mice	100, 200, 400, 800, 100, and 2000 mg/kg	—	The extract is safe up to 2000 mg/kg	[220]
			Sprague Dawley rats				
*Alternanthera brasiliana*	ME of leaves	Determination of LD_50_	Swiss albino mice	2.0 g/kg BW	—	The extract is safe up to 2000 mg/kg	[221, 222]
			Sprague Dawley rats	2.0 g/kg BW	—		
		Acute toxicity	Swiss albino mice	2.0 g/kg BW	—		
			Sprague Dawley rats	2.0 g/kg BW	—		
*Alternanthera brasiliana*	ME of leaves	Determination of LD_50_	Swiss albino mice (male)	2000 mg/kg	—	The extract is safe up to 2000 mg/kg, showed no changes in motor activity or behavior	[24]
		Acute toxicity	Swiss albino mice (male)	2000 mg/kg	—		
		Gross effect	Swiss albino mice (male)	2000 mg/kg	—		
*Alternanthera brasiliana*	EaE of leaves	Acute toxicity	Swiss albino mice	2000 mg/kg	—	LD_50_: >2000 mg/kg. Until the evaluated dose, no gross behavior changes or mortality were observed	[185]
		Determination of LD_50_	Swiss albino mice	Uninformed	—		
*Alternanthera brasiliana*	EE of leaves	Viability of flies	*Drosophila melanogaster*	10, 20, and 40 µg/mL	—	EE exhibited low toxicity, as mortality significantly differed from the control only after 24 h, without a dose‐dependent response. After 48 h, EE killed more than 50% of the flies	[26]
		Locomotor assay	*Drosophila melanogaster*	10, 20, and 40 µg/mL	—		
*Alternanthera brasiliana*	AqE of leaves	Determination of LC_50_	*Artemia salina*	[] up to 1000 µg/mL		LC_50_: 500 µg/mL	[27]
*Alternanthera brasiliana*	HaE of leaves	MTT assays	RAW 264.7 and L929	3.9–500.0 µg/mL	Doxorubicin	IC_50_: 297.5 ± 22.8 µg/mL for RAW 264.7 and IC_50_: 340.7 ± 42.4 µg/mL for L929	[193]
*Alternanthera littoralis*	EE of aerial parts	Biometric parameters	Swiss mice of both genders	100 and 1000 mg/kg	—	The extract had no effect on weight, number of implantations, live and dead fetuses, resorptions, fetal viability, resorption rate, post‐implantation loss rate, placental index, weight adequacy for gestational age, head‐to‐tail distance, or urogenital distance in males and females Malformations detected: hyperextension of the forelimbs, unilateral hyperflexion of the hindlimb, curly tail, gastroschisis, hydrocephaly, hydronephrosis, femur agenesis, and reduced ossification of skull bones EE did not change the frequency of micronuclei	[253]
		Reproductive parameters and embryofetal development					
		Micronucleus (MN) levels in peripheral blood					
		Splenic phagocytosis					
*Alternanthera philoxeroides*	ME of whole plant	Acute toxicity test	Swiss albino mice	100, 200, 300, 600, 800, 1000, 2000, and 3000 mg/kg BW	—	ME not cause changes in behavior or mortality; therefore, it is considered safe up to the evaluated dose	[39]
*Alternanthera philoxeroides*	ME extract	Swiss albino mice (female)	Body weight and relative organ weight	250, 500, and 1000 mg/kg	—	ME did not cause changes in BW or organ weight or cause architectural or degenerative changes No changes were observed in erythropoiesis, morphology, or osmotic fragility of RBC. No significant changes were observed in leukocyte counts except for neutrophils, which decreased markedly. No changes were observed in platelet count and platelet indices ME is not toxic	[132]
			Histopathological analyses		—		
			Hematological analyses		—		
*Alternanthera repens*	AqE of aerial parts	Determination of LD_50_	Swiss mice (female)	0.25–8 g/kg	—	LD_50_: 3.4782 and 4.0639 for AqE and EE, respectively. On the basis of LD_50_ values, the extracts are slightly toxic	[46]
	EE of aerial parts	Determination of LD_50_	Swiss mice (female)	0.25–8 g/kg	—		
*Alternanthera sessilis*	ME of aerial parts	Acute toxicity test	Swiss albino mice (male)	100, 200, 300, 600, 800, 1000, 2000, and 3000 mg/kg BW	—	Non‐toxic up to the evaluated dose	[204]
*Alternanthera sessilis*	EE of stem	MTT assays	NHDF cells	15.62, 31.25, 62.5, 125, 250, and 500 µg/mL	—	Non‐toxic up to the evaluated dose	[56]
			HDF‐D cells				
			HaCaT cells				
*Alternanthera sessilis*	ME of whole plant	Acute toxicity test	Female albino rats	250, 1000, and 2500 mg/kg BW	—	Results not reported	[51]
*Alternanthera sessilis*	AgNPs of leaves	Toxic effect of AgNPs against non‐target organisms	*Poecilia reticulata*	Uninformed	Silver nitrate	The AgNPs complex was not toxic to *Poecilia reticulata*	[158]
*Blutaparon portulacoides*	EE of stems	Acute toxicity test	Female Wistar rats	2000 mg/kg	—	No signs or symptoms of acute and clinical oral pathophysiology were observed with EE use	[71]
*Blutaparon portulacoides*	AqE of whole plant	Acute toxicity	Female Wistar rats	2000 mg/kg	—	LD_50_: >2000 mg/kg No significant behavioral or physiological changes were induced in female rats	[152]
*Gomphrena celosioides*	AqE of leaves	Preliminary acute toxicity study	Adult Sprague–Dawley rats	20, 40, 80, 160, 320, and 640 mg/kg	—	Non‐toxic up to evaluated dose	[87]
*Gomphrena celosioides*	HeE of whole plant	Brine shrimp toxicity assay	Brine shrimp nauplii	1000, 100, and 10 ppm	Podophylotoxin	LC_50_ (µg/mL) of HeE: 52.146 LC_50_ (µg/mL) of 449: 110.654 LC_50_ (µg/mL) of ME: 77.978 The LC_50_ values of HeE and ME suggest the presence of cytotoxic and/or insecticidal compounds. The LC_50_ indicates weak cytotoxicity	[82]
	449						
	ME of whole plant						
*Gomphrena celosioides*	AcE and AqE of flowers, leaves, twigs, and whole plant	MTT assay	Vero monkey kidney cells	Range of 0.03–1 mg/mL	—	All extracts showed low toxicity	[89]
*Gomphrena celosioides*	EE of aerial parts	Acute oral toxicity	Adult Wistar rats	2000 mg/kg	—	LD_50_: >2000 mg/kg EE from aerial parts is safe, as no rats exhibited clinical signs of toxicity. Histopathological studies showed no hepatotoxicity, nephrotoxicity, or hematotoxicity	[254]
		Subacute oral toxicity		75, 150, or 300 mg/kg	—		
*Gomphrena celosioides*	EE of aerial parts	Teratogenesis and genotoxicity	Pregnant mice (*Mus musculus*)	100, 1000, and 2000 mg/kg	—	Not alter the final weight, weight gain, uterine weight, or net weight gain Not affect the numbers of implantations, live fetuses, dead fetuses, or resorptions Not change fetal weight, placental weight, or the placental index The frequency of malformations (external, visceral) did not differ between the EE‐treated and control groups EE ↑ the frequency of abnormal sternum and fibula rotation over the tibia EE did not change the frequency of micronuclei These results suggest that daily doses up to 2000 mg/kg are not maternotoxic	[255]
		Biological tests	Pregnant mice (*Mus musculus*)	100, 1000, and 2000 mg/kg	—		
		Biometric parameters	Pregnant mice (*Mus musculus*)	100, 1000, and 2000 mg/kg	—		
		Reproductive performance and embryofetal development	Pregnant mice (*Mus musculus*)	100, 1000, and 2000 mg/kg	—		
		Micronucleus in peripheral blood					
		Splenic phagocytosis					
*Gomphrena globosa* var. *albiflora* (white amaranth)	HE of flowers	SRB assay	PLP2 cells	Uninformed	—	Non‐toxic up to the evaluated dose GI_50_ (µg/mL): >400	[73]
*Gomphrena haageana* K. (red amaranth)	HE of flowers	SRB assay	PLP2 cells	Uninformed	—	Non‐toxic up to the evaluated dose GI_50_ (µg/mL): >400	[73]
*Gomphrena* sp. (pink globe amaranth)	HE of flowers	SRB assay	PLP2 cells	Uninformed	—	Non‐toxic up to the evaluated dose GI_50_ (µg/mL): >400	[73]
*Gomphrena globosa*	HaE of flowers	Alamar blue and neutral red tests	HaCaT cells and BJ cells	50, 250, and 500 µg/mL	—	It did not affect BJ cell viability but ↓ HaCat cell viability at high concentrations	[153]
*Hebanthe eriantha*	ME of roots	Determination of LC_50_	*Artemia salina*	Range of 7.81–1000 µg/mL	Potassium dichromate	MEs showed toxicity in *A. salina*, with lethality ranging from 26.7% to 60% at concentrations of 31.25–1000 µg/mL	[230]
*Pfaffia glomerata*	HaE of roots	Acute toxicity test	Wistar rats (male)	3 g/kg	—	No behavioral changes or deaths were observed in rats (LD_50_: >3 g/kg)	[116]
		Determination of LD_50_					
*Pfaffia glomerata*	AqE of aerial parts	Cytotoxicity assay	J774 cell line	1, 10, and 100 µg/mL	—	Non‐toxic up to the evaluated dose	[234]
*Pfaffia glomerata*	Commercial root dry extract	Cytotoxic assay	Wistar rats	0.15, 1.5, and 15 mg/mL	—	No cytotoxic effects or mutagenic potentials were observed	[112]
		Chromosomal aberration test	Wistar rats	0.15, 1.5, and 1.5 mg/mL	Cyclophosphamide		
*Pfaffia glomerata*	FD of roots	MTT assay	BMDM (bone marrow‐derived macrophage)	250, 25, 2.5, and 0.25 µg/mL	—	Cytotoxicity was dose‐dependent, with lower cell viability at higher concentrations and higher viability for the concentrations of 2.5 and 0.25 µg/mL, maintaining viability >65%	[162]
	FD of aerial part	MTT assay	BMDM (bone marrow‐derived macrophage)	250, 25, 2.5, and 0.25 µg/mL	—		
*Pfaffia paniculata*	Powdered roots	ALT activity	Inbred BALB/cICB mice	200 and 400 mg/kg	—	Histopathological changes were not observed in the liver, kidney, or spleen. No changes in BW and ALT activities were detected	[207]
		Histopathological study					
*Pfaffia paniculata*	BuF of roots	BW measurement	SWISS mice (male)	200 mg/kg	—	↓ Weight gain on Days 13 and 15. No hepatic or renal toxicity was observed, according to histopathological analysis and levels of ALT, AST, ^γ^‐GT, urea, and creatinine	[209]
		Biochemical analysis					
		Histopathological study					
*Pfaffia paniculata*	ME of roots	Histopathological study	Adult BALB/c mice (male)	250, 500, and 1000 mg/kg	—	No histopathological alterations were observed in the liver, kidney, lung, brain, eye, or cerebellum at the studied dose. Only a tendency toward ↓ BW was observed at a [] of 1000 mg/kg	[183]

Abbreviations: AqE, aqueous extract; EaE, ethyl acetate extract; EE, ethanolic extracts; HaE, hydroalcoholic extract; HE, hydromethanolic extracts; ME, methanolic extracts; *n*HE, *n*‐hexane extracts.

The EE of *B. portulacoides* and the AqE of *G. celosioides* did not show toxicity in rats up to the dose evaluated [71, 87], but the HeE and ME of *G. celosioides* showed weak cytotoxicity against brine shrimp or nauplii, and the AcE and AqE extracts of this same plant showed low toxicity against Vero cells of monkey kidneys [82, 89]. On the other hand, the HE of *G. haageana* and *G. globosa* flowers did not show toxicity against PLP2 cells [73]. Additionally, the HaE of *G. globosa* flowers did not show toxicity against BJ cells but did against HaCat cells [153]. Regarding *P. glomerata* and *P. paniculata*, no toxic effects were observed up to the doses evaluated in rats (3 g/kg) and mice (1 g/kg), respectively [112, 116, 183, 207, 209]. It was also reported that the commercial extract of the root of *P. glomerata* has no cytotoxic effect on the J774 cell line, nor mutagenic effects [234].

It can be concluded that the extracts, fractions, and commercial preparations of the studied members of this subfamily do not present toxicity, or it is very low. Furthermore, toxicological safety evaluations are essential for the application or use of plants and the development of new drugs. Therefore, it is necessary to evaluate the toxicity of the other members of this subfamily.

## 7 Relationship Among Traditional Uses, Biological Activity, and Chemical Profile

Different parts or even the whole plant of the members of Gomphrenoideae have been used in traditional medicine to treat bacterial, fungal, and viral infections, parasitic diseases, diabetes, cancer, hypertension, inflammatory diseases, gastrointestinal diseases, and liver damage. They have also been used as antioxidants, analgesics, and diuretics, as well as to treat other pathologies. Therefore, during the last decades, they have been studied at the laboratory level, mainly to verify their antimicrobial, antioxidant, anticancer, antidiabetic, hepatoprotective, gastroprotective, diuretic, and insecticidal properties. An attempt to compare traditional use with results from the laboratory is described as follows:

***Alternanthera bettzickiana***: Out of the 17 traditional uses, only 4 have been evaluated at the laboratory level, confirming its antimicrobial, antioxidant, and cytotoxic (against the A549 cell line) activities. Additionally, two in vitro studies showed antiarthritic potential. The results obtained to date under laboratory conditions confirm only 23.53% of the traditional uses; therefore, it is necessary to confirm the other traditional uses.

***Alternanthera brasiliana***: To date, only 10 of the 25 traditional uses of this plant have been evaluated, confirming its anticancer potential against the A549, CaCo‐2, HT‐29, and Hep‐G2 cell lines, as well as its positive effect in mice bearing EAC. In addition, its use in the treatment of infections was confirmed, because it has been reported to have activity against some bacteria and yeasts. Its anti‐inflammatory, analgesic, anticonvulsant, anxiolytic, and immunomodulatory activities were also confirmed, as well as its great potential in wound healing.

***Alternanthera flavescens***: None of its traditional uses have been evaluated under laboratory conditions, but its antioxidant and anticancer activities have been evaluated.

***Alternanthera littoralis* P. Beauv**. (*Alternanthera maritima* (Mart.) St. Hil.): Currently, its two traditional uses have been evaluated under laboratory conditions, confirming that this plant has antifungal activity against yeasts and anti‐inflammatory activity. Although its traditional uses do not include antioxidant, antiparasitic, and immunomodulatory activities, these properties were evidenced through pharmacological studies.

***Alternanthera paronychioides***: Of the seven uses in traditional medicine, only antidiabetic activity has been evaluated under laboratory conditions through in vitro tests, suggesting that this plant has antidiabetic properties as well as antioxidant activity.

***Alternanthera philoxeroides***: Of the 21 traditional uses, 4 have been evaluated under laboratory conditions, confirming that this plant has antiviral activity (e.g., anti‐herpes, anti‐measles), potential antidiabetic activity (inhibits α‐glycosidase), analgesic, and antidepressant effects. Additionally, this plant has been reported to have antibiotic, antioxidant, cytotoxic (against HeLa cells), anticoagulant, and cardioprotective activities, as well as an antidementia and memory‐enhancing effect in mice.

***Alternanthera porrigens* Kuntze**: None of its traditional uses have been confirmed under laboratory conditions.

***Alternanthera pungens* Kunth**: None of its traditional uses have been confirmed under laboratory conditions. However, some articles report that this plant has antitumor activity, but it does not have antifungal activity, nor is it a significant antioxidant.

***Alternanthera repens*** (*Alternanthera caracasana)*: Of the nine traditional uses, only its antidiarrheal activity has been evaluated and confirmed under laboratory conditions. It should be noted that this plant has also been reported to have no activity against *C. albicans*.

***Alternanthera sessilis***: To date, only 11 of the 63 traditional uses have been evaluated under laboratory conditions, confirming that it has antiasthmatic, antidiarrheal, antidiabetic, hypotensive, hepatoprotective, analgesic, wound healing, anti‐inflammatory, antioxidant, and antimicrobial (against bacteria) activity. It has also been shown to be cytotoxic against HeLa, Panc‐1, MIA, PaCa‐2, Capan‐1, PC3, L929, and MCF‐7 cells and to have antiallergic and CNS‐stimulating properties.

***Alternanthera tenella***: Of the 18 uses in traditional medicine, only the anti‐inflammatory, analgesic, and antimicrobial activities have been confirmed by pharmacological studies. Additionally, it has been reported to also have antioxidant, anticancer, and immunomodulatory properties.

***Blutaparon portulacoides***: Of the two traditional uses, studies confirm its potential to treat vulvovaginitis under laboratory conditions. Additionally, it has also been reported to have antibiotic, antiparasitic, anti‐inflammatory, and analgesic properties, as well as diuretic and cardioprotective effects.

***Gomphrena agrestis***: There are no reports of traditional uses but has been evaluated for antimicrobial, antifungal, and antiparasitic activity.

***Gomphrena arborescens* L.f**.: Under laboratory conditions, none of the traditional uses have been confirmed.

***Gomphrena boliviana***: Four traditional uses have been reported, of which only antimicrobial activity has been confirmed under laboratory conditions.

***Gomphrena celosioides***: Of the 22 traditional uses, 9 have been evaluated through pharmacological studies, confirming that *G. celosioides* has the potential to be used in the prevention and treatment of Type 2 diabetes, as well as in the treatment of gastric lesions, to counteract renovascular hypertension, and to treat infections (antibiotic and antifungal activities). It also has hepatoprotective, analgesic, anti‐inflammatory, and immunomodulatory properties. Additionally, it has been reported to have cardioprotective and anticarcinogenic activity.

***Gomphrena elegans***: Its use in traditional medicine has not been reported, but pharmacological studies have shown that it has cytotoxicity against the HCT‐8, SF‐295, and MDA‐MB‐435 cell lines, as well as potential as an insecticide against *Aedes aegypti*.

***Gomphrena globosa***: To date, only 2 of its 20 traditional uses have been evaluated under laboratory conditions, which report that this plant has antibacterial, antifungal, and antioxidant activities, although these are considered weak. Assays have shown no cytotoxic activity against the MT‐1, MT‐2, B16F10, HeLa, and MK‐1 cell lines, nor any sun protection effect or AChE inhibition. However, it has anti‐inflammatory and anti‐collagenase properties.

***Gomphrena haageana* K**.: No traditional uses have been reported, but it has been found to have antioxidant and anti‐inflammatory properties.

***Gomphrena macrocephala***: Its two common uses have not been evaluated by pharmacological studies, but two compounds isolated from this plant have been reported to show cytotoxic activity against HSC‐2 cells.

***Gomphrena martiana***: Of the eight traditional uses, only its activity in the treatment of infections has been confirmed by antimicrobial studies, indicating that it has antibacterial and antifungal activity. Additionally, its activity against the KB cell line has been evaluated, showing moderate cytotoxicity against it. It has also been shown to have a beneficial effect on mice bearing S‐180 cells or EAC.

***Gomphrena virgata* Mart**.: Its five traditional uses have not yet been confirmed by pharmacological studies, but it has been reported that this plant can inhibit the proliferation of lymphocytes.

***Guilleminea densa***: Only one of the three traditional uses was evaluated by a pharmacological study, which showed that the plant has a gastroprotective effect, inhibiting gastric lesions and preventing the reduction of mucus induced by toxic agents, confirming its use in the treatment of gastric ulcers.

***Iresine difusa***: Of the 12 traditional uses, only the anticancer potential has been evaluated under laboratory conditions. It was reported to have cytotoxic activity against the human LNCaP cell line, but not against PC3. Additionally, in vitro and in vivo studies have shown that this plant can be used to treat diseases related to neuroinflammation.

***Iresine herbstii***: Of the 13 traditional uses, only 2 have been investigated pharmacologically. It was found to have anticancer potential, showing cytotoxic activity against the HeLa cell line. But no significant wound‐healing activity. Although its traditional uses do not mention antibiotic, antioxidant, or neurological activity, in vitro studies suggest that this plant has high antibacterial and antioxidant activities as well as has a beneficial effect on the CNS.

***Pfaffia glomerata***: Of the 22 known traditional uses, only 7 have been evaluated in the laboratory, confirming its anti‐inflammatory, analgesic, and stimulant properties. It also reduces stress and depressive behaviors and protects against the development of anxiety but has shown variable antioxidant activity. Other studies indicate that this plant has antiparasitic activity against *Leishmania braziliensis* and *L. amazonensis*, and its fractions have shown activity against *T. cruzi*. Additionally, this plant has demonstrated inhibition of melanogenesis.

***Pfaffia paniculata***: Of the 13 traditional uses, only its anticancer activity (in vitro and in vivo) and anti‐inflammatory properties have been confirmed through pharmacological studies. It was also observed that it did not show antitumor activity in EAC‐bearing mice.

***Pfaffia townsendii***: Of the four traditional uses reported, only the anti‐inflammatory activity has been confirmed through an in vivo study. Although its traditional uses do not include antioxidant activity, an in vitro study showed that it has antioxidant potential.

***Tidestromia oblongifolia***: Its traditional use as an analgesic has not been confirmed by pharmacological studies, highlighting that, to date, no study has been carried out to evaluate its biological potential.

From the above, it can be concluded that only 21.18% of the traditional uses reported for the members of this subfamily have been evaluated under laboratory conditions, whereas 78.2% remain untested. These data are relevant because they suggest a promising field of study, as most studies have shown that the plants of this subfamily indeed have biological activity consistent with their traditional uses. The traditional uses of these plants justify multidisciplinary research, which may include determination of biological activity, identification of chemical profiles, correlation between phytochemical profiles and biological activity, comparative studies of extracts, fractions, isolated compounds, and drug delivery, as well as comparative studies between varieties, plants collected from different locations under varying environmental conditions, and in vitro systems.

## 8 Gomphrenoideae Subfamily: Perspectives and Research Directions

Despite significant progress in understanding the biotechnological and chemical potential of the Gomphrenoideae subfamily, many research opportunities remain. These opportunities can be categorized into the following key areas of focus.

### 8.1 Evaluation of the Chemical Profile and Activity of Species That Remain Unstudied

Over 80% of species have not yet been studied in terms of their chemical profiles and biological activities, presenting a valuable opportunity to discover new chemical structures and significant biological activities that could be of interest to the pharmaceutical industry.

### 8.2 In Vitro Cultures: A Sustainable and Efficient Strategy in Biotechnological Studies

In the sections on pharmacological activity and phytochemical, it is evident that the same species can exhibit multiple chemical profiles, which, in turn, modulate their biological activity. This variation occurs because the phytochemical profile of plants is influenced by various biotic and abiotic factors, including climatic conditions, UV radiation exposure, soil characteristics, nutrient availability, and interactions with other organisms, such as microorganisms.

In this context, a viable alternative is the use of in vitro plant tissue cultures, which allow for the production of genetically identical plants or callus under controlled growth conditions. The use of in vitro cultures not only standardizes growth conditions but also optimizes the production process and helps prevent ecosystem degradation.

### 8.3 Co‐Cultures of Plants and Endophytic Microorganisms

Endophytic microorganisms play a crucial role in the phytochemistry of plants in natura. In this context, three key theories should be considered: (1) Endophytic microorganisms are responsible for producing certain chemical compounds; (2) microorganisms function as elicitors in the plant and stimulate the production of secondary metabolites; and (3) the interaction between the plant and the microorganism is necessary for the synthesis of specific compounds, which cannot be synthesized in their absence. This could be because endophytes act as stimulants, provide essential precursors for synthesis, or vice versa.

In this context, the use of in vitro plant tissue cultures in co‐culture with one or more endophytic microorganisms represents a valuable approach, which also allow for a better understanding of plant‐microorganism interactions.

### 8.4 Endophytic Microorganisms as a Source of Secondary Metabolites

Endophytic microorganisms are sometimes responsible for producing certain plant secondary metabolites, making them a sustainable alternative that minimizes environmental impact, shortens production times, and allows for process optimization.

Additionally, microorganisms are recognized as a promising source of bioactive molecules due to their metabolic plasticity, which enables them to mimic the metabolism of their host.

## 9 Conclusion

The members of the Gomphrenoideae subfamily (Amaranthaceae) have been used in traditional medicine around the world since ancient times. Although some members of this subfamily have been studied at the pharmacological and phytochemical levels, many others remain unstudied, presenting opportunities for research. Additionally, the literature review reveals a direct relationship between the traditional uses of these plants and the biological activity exhibited by the extracts, fractions, and compounds studied. There is also a correlation between the chemical profile and the types of compounds present and the observed biological activity. Demonstrating the importance of understanding the pharmacological activity of plants to harness this knowledge for the development of new drugs.

Research focused on discovering bioactive compounds can contribute to the development of drugs with fewer side effects and greater effectiveness than current options. Currently, drugs for treating pain, inflammation, cancer, diabetes, microbial infections, and oxidative stress‐related conditions are known for their side effects and limited effectiveness. This highlights the need to discover new drugs.

In general, it can be concluded that some phytochemicals found in the subfamily Gomphrenoideae can be used in drug development. Studying the members that have not yet been researched could lead to the discovery of new chemical compounds and the potential development of new drug.

Currently, 512 compounds have been isolated, including 173 phenolic compounds, 95 terpenoids, 87 lipid compounds, 62 alkaloids, and 95 other types of compounds, with phenolic compounds being the most abundant. These different extracts, fractions, and isolated compounds have been associated with various biological activities, such as antioxidant, analgesic, antibacterial, antifungal, antiparasitic, anticancer, antitumor, anti‐inflammatory, antidiabetic, antiarthritis, cardioprotective, healing, diuretic, gastroprotective, hepatoprotective, radioprotective, and hypertension and blood pressure management. The most extensively studied biological activities to date are anticancer, antimicrobial, and antioxidant activities. In conclusion, these plants represent a promising source of bioactive molecules with low or no toxicity.

## Author Contributions

**Dayanna Isabel Araque Gelves**: bibliographic survey, writing and revision of text, design of graphical abstract, design of chemical structures and tables. **Giulia Cristina Andreoli de Souza**: bibliographic survey, writing and revision of text, design of graphical abstract, design of chemical structures and tables. **Alvaro Jose Hernandez Tasco**: bibliographic survey, writing and revision of text, design of graphical abstract, design of chemical structures and tables. **Marcos Jose Salvador**: bibliographic survey, writing and revision of text, design of graphical abstract, design of chemical structures and tables.

## Conflicts of Interest

The authors declare no conflicts of interest.

## Supporting information



Supporting Information

## Data Availability

Data sharing is not applicable to this article as no new data were created or analyzed in this study.
