# Supplementary material for: Ethnomedicinal Uses, Phytochemistry, Pharmacological Activities, and Toxicology of the Subfamily Gomphrenoideae (Amaranthaceae): A Comprehensive Review
Source: Chem Biodivers. 2025 May 24;22(9):e202500530. doi: 10.1002/cbdv.202500530 (PMC12435438; doi:10.1002/cbdv.202500530)
Supplement: Supplementary file 1 — Supporting Information [file CBDV-22-e202500530-s001.docx]

**Supplementary Table 1.** Antimicrobial activity of the Gomphrenoideae subfamily

| Species | Extract(s)/ No. compounds | | Assay method | | Dose | | Positive control | | Strain | Activity | | Effects/mechanisms | References | |
| --- | --- | --- | --- | --- | --- | --- | --- | --- | --- | --- | --- | --- | --- | --- |
|  |  |  |  |  |  |  |  |  |  | MIC (μg/mL) | ZI (mm) |  |  | |
| **Antibacterial activity** | | | | | | | | | | | | | | |
| *Alternanthera bettzickiana* | Crude AuNPs of AqE of leaves | | Well diffusion method | | 10, 20, 30, and 40 µl and for | | Ciprofloxacin | | *B. subtilis MTCC 441* | - | 10 ± 0.17 | Crude AuNPs only showed activity against *B. subtilis*.  The AuNPs showed activity against all the strains.  The optimized AuNPs showed the highest activity, especially against *M. luteus*, *P. aeruginosa,* and *E. aerogenes.* | [21] | |
|  |  |  |  |  |  |  |  |  | *E. aerogenes MTCC 111* | - | 05 ± 0.00 |  |  |  |
|  |  |  |  |  |  |  |  |  | *M. luteus MTCC 106* | - | 05 ± 0.17 |  |  |  |
|  |  |  |  |  |  |  |  |  | *P. aeruginosa MTCC 841* | - | 05 ± 0.00 |  |  |  |
|  |  |  |  |  |  |  |  |  | *S. typhi MTCC-734* | - | 05 ± 0.17 |  |  |  |
|  |  |  |  |  |  |  |  |  | *S. aureus MTCC 3940* | - | 05 ± 0.17 |  |  |  |
|  | AuNPs of AqE of leaves | | Well diffusion method and MIC | | 10, 20, 30, and 40 µl and | | Ciprofloxacin | | *B. subtilis MTCC 441* | - | 14 ± 0.15 |  |  |  |
|  |  |  |  |  |  |  |  |  | *E. aerogenes MTCC 111* | - | 0.6 ± 0.15 |  |  |  |
|  |  |  |  |  |  |  |  |  | *M. luteus MTCC 106* | - | 22 ± 0.44 |  |  |  |
|  |  |  |  |  |  |  |  |  | *P. aeruginosa MTCC 841* | - | 14 ± 0.58 |  |  | |
|  |  |  |  |  |  |  |  |  | *S. typhi MTCC-734* | - | 16 ± 0.44 |  |  | |
|  |  |  |  |  |  |  |  |  | *S. aureus MTCC 3940* | - | 16 ± 0.88 |  |  | |
|  | Optimized AuNPs of AqE of leaves | | Well diffusion method | | 10, 20, 30, and 40 µl and for | | Ciprofloxacin | | *B. subtilis MTCC 441* | - | 14 ± 0.43 |  |  | |
|  |  |  |  |  |  |  |  |  | *E. aerogenes MTCC 111* | - | 24 ± 0.17 |  |  | |
|  |  |  |  |  |  |  |  |  | *M. luteus MTCC 106* | - | 30 ± 0.33 |  |  | |
|  |  |  |  |  |  |  |  |  | *P. aeruginosa MTCC 841* | - | 28 ± 0.33 |  |  | |
|  |  |  |  |  |  |  |  |  | *S. typhi MTCC-734* | - | 17 ± 0.13 |  |  | |
|  |  |  |  |  |  |  |  |  | *S. aureus MTCC 3940* | - | 19 ± 0.33 |  |  | |
|  |  | |  | |  | |  | |  | MIC (mg/mL) | ZI (mm) |  |  | |
| *Alternanthera brasiliana* | HeE of whole plant | | Agar-well diffusion method (well technique with double layers) and method of microdilution | | Uninformed | | Bacitracin | | *S. aureus ATCC 14458* | 25.0 | - | HeE and EE at concentrations of 2.5 and 25 mg/kg respectively, did not show activity in microorganisms in the absence of irradiation, but in the presence of diode laser irradiation, HeE and EE showed biocidal activity against *S. aureus* and *S. epidermis* respectively, as well as EE ↓ the viability of *S. aureus* by 99.86% and HeE ↓ the viability of *S. epidermis* by 99.97%. | [23] | |
|  |  |  |  |  |  | |  | | *S. epidermidis ATCC 12228* | 25.0 | - |  |  |  |
|  |  |  |  |  |  | |  | |  |  |  |  |  |  |
|  | EE of whole plant | |  |  | Uninformed | | Bacitracin | | *S. aureus ATCC 14458* | 50.0 | - |  |  |  |
|  |  |  |  |  |  | |  | | *S. epidermidis ATCC 12228* | 50.0 | - |  |  |  |
|  |  | |  | |  | |  | |  | %V of MI^-^ | %V of MI^+^ |  |  |  |
|  | HeE of whole plant | | Photosensitization assays | | 2.5 mg/mL | | Methylene blue and bacitracin | | *S. aureus ATCC 14458* | 100 | 0.00 |  |  |  |
|  |  |  |  | |  | |  |  | *S. epidermidis ATCC 12228* | 100 | 0.03 |  |  |  |
|  | EE of whole plant | | Photosensitization assays | | 25 mg/mL | | Methylene blue and bacitracin | | *S. aureus ATCC 14458* | 100 | 0.14 |  |  |  |
|  |  |  |  |  |  |  |  |  | *S. epidermidis ATCC 12228* | 100 | 0.00 |  |  |  |
|  |  | |  | |  | |  | |  | MIC (μg/mL) | ZI (mm) |  |  | |
| *Alternanthera brasiliana* | FEaMc (4:1), or FEaMc (1:1), or FEaMcM (4:1:0.1) or FM | | Microdilution broth  method | | 50, 100, 250, 500 µg/mL | | Tetracycline and norfloxacin | | *B. subtilis ATCC 6623* | NDNS | | Only FEaMc (1:1) showed activity against gram-positive bacteria, but Gram-negative bacteria were resistant to all fractions.  Of the 7 subfractions, only 3 showed activity against Gram-positive bactéria with a MIC of 50 µg/mL.  Compounds 340, 341, 342, 344, 346, 354, 358, 359, and 361 showed activity against the studied strains. | [32] | |
|  |  |  |  |  |  |  |  |  | *E. coli ATCC 25922* | NDNS | |  |  |  |
|  |  |  |  |  |  |  |  |  | *M. luteus ATCC 9341* | NDNS | |  |  |  |
|  |  |  |  |  |  |  |  |  | *P. aeruginosa ATCC 15442* | NDNS | |  |  |  |
|  |  |  |  |  |  |  |  |  | *S. aureus ATCC 25923* | NDNS | |  |  |  |
|  | 7 sub-fractions of FEaMc (1:1) | | Microdilution broth  method | | 50, 100, 250, 500 µg/mL | | Tetracycline and norfloxacin | | *B. subtilis ATCC 6623* | 50 | |  |  |  |
|  |  |  |  |  |  |  |  |  | *M. luteus ATCC 9341* | 50 | |  |  |  |
|  |  |  |  |  |  |  |  |  | *S. aureus ATCC 25923* | 50 | |  |  |  |
|  | 337, 338, 339, 340, 341, 342, 343, 344, 345, 346, 354, 356, 357, 358, 359, 360, 361 | | Microdilution broth  method | | 50 µg/mL | | Tetracycline and norfloxacin | | *B. subtilis ATCC 6623* | NDNS | |  |  | |
|  |  |  |  |  |  |  |  |  | *M. luteus ATCC 9341* | NDNS | |  |  | |
|  |  |  |  |  |  |  |  |  | *S. aureus ATCC 25923* | NDNS | |  |  | |
| *Alternanthera brasiliana* | EE of leaves | | Microdilution broth  method | | 0.5-512 μg/mL | | Gentamicin | | *E. coli MDR* | ≥1024 μg/mL | | EE with clinically irrelevant antimicrobial activity, but it has a synergistic effect with gentamicin since it manages to lower the MIC value of gentamicin. | [26] | |
|  |  |  |  |  |  |  |  |  | *P. aeruginosa MDR* | ≥1024 μg/mL | |  |  |  |
|  |  |  |  |  |  |  |  |  | *S. aureus MDR* | ≥1024 μg/mL | |  |  |  |
|  |  |  | Evaluation of the modification of antibiotic activity (Gentamicin) | | 0.6 - 2500 μg/mL | | Gentamicin | | *E. coli MDR* | NDNS | |  |  |  |
|  |  | |  |  |  |  |  |  | *P. aeruginosa MDR* | NDNS | |  |  |  |
|  |  | |  |  |  |  |  |  | *S. aureus MDR* | NDNS | |  |  |  |
|  |  | |  | |  | |  | |  | MIC (μg/mL) | MBC (μg/mL) |  |  | |
| *Alternanthera brasiliana* | AqE of leaves | | Microdilution broth  method | | 3.9 – 2000 μg/mL | | Amoxicillin | | *E. faecalis* ATCC 6057 | 2000 | NE | Showed no activity against: *B. subtilis* 16-UFPEDA, *E. coli* ATCC 25922, *S. aureus* 731-UFPEDA, and *E. aerogenes* 739-UFPEDA.  The best activity was against *M. smegmatis*. | [27] | |
|  |  |  |  |  |  |  | Erythromycin | | *M. luteus* ATTC 2225 | 2000 | NE |  |  |  |
|  |  |  |  |  |  |  | Amoxicillin | | *M. smegmatis* 71-UFPEDA | 15.6 | 1000 |  |  |  |
|  |  |  |  |  |  |  | Ciprofoxacin | | *P*. *aeruginosa* 39-UFPEDA | 1000 | NE |  |  |  |
|  |  |  |  |  |  |  | Ciprofoxacin | | *P*. *aeruginosa* 736-UFPEDA | 2000 | NE |  |  |  |
|  |  |  |  |  |  |  | Amoxicillin | | *S. aureus* ATCC 6538 | 2000 | NE |  |  |  |
| *Alternanthera brasiliana* | HaE of leaves | | Microdilution broth  method | |  | |  | | *P. aeruginosa* | > 16 | 16.0 mg/mL | Weak antimicrobial activity. | [193] | |
|  |  |  |  |  |  |  |  |  | *S. aureus* ATCC 25923 | 2.0 mg/mL | 4.0 mg/mL |  |  |  |
|  |  |  |  |  |  |  |  |  | *S. aureus* oxacillin resistant | > 16 | 16.0 mg/mL |  |  |  |
|  |  |  |  |  |  |  |  |  | *E. coli* ATCC 25922 | 4.0 mg/mL | 8.0 mg/mL |  |  |  |
|  |  | |  | |  | |  | |  | MIC (μg/mL) | ZI (mm) |  |  | |
| *Alternanthera caracasana HBK* | ME of aerial part | | Disk diffusion test | | Uninformed | | CAM | | *B. subtilis* |  | - | None of the extracts showed activity against *S. boydii* ATCC 8700, *E. coli* ATCC 25922, *E. agglomerans* ATCC 27155, *S. typhi* ATCC 19430, *V. cholerae* CDC V12*, V. cholerae* INDRE 206*, V. cholerae, E. aerogenes,* and *P. aeruginosa.*  HeE and ClE did not show activity against the evaluated microorganisms.  EaE was the extract with the largest zones of inhibition.  *V. cholera* was the microorganism most sensitive to 7-methoxycumarin (**159**). | [33] | |
|  |  |  |  |  |  |  |  |  | *S. aureus* ATCC 12398 |  | 10.07 ± 0.57 |  |  |  |
|  |  |  |  |  |  |  |  |  | *S. epidermidis* |  | 12.00 ± 1.0 |  |  |  |
|  |  |  |  |  |  |  |  |  | *S. lutea* |  | - |  |  |  |
|  |  |  |  |  |  |  |  |  | *V. cholerae* No-01 ATCC 35971 |  | - |  |  |  |
|  | AcE of aerial parts | | Disk diffusion test | | Uninformed | | CAM | | *B. subtilis* |  | 8.00 ± 0.50 |  |  |  |
|  |  |  |  |  |  |  |  |  | *S. aureus* ATCC 12398 |  | 12.67 ± 1.15 |  |  |  |
|  |  |  |  |  |  |  |  |  | *S. epidermidis* |  | 12.67 ± 0.58 |  |  |  |
|  |  |  |  |  |  |  |  |  | *S. lutea* |  | 9.33 ± 1.15 |  |  |  |
|  |  |  |  |  |  |  |  |  | *V. cholerae* No-01 ATCC 35971 |  | 8.00 ± 0.50 |  |  |  |
|  | EaE of aerial parts | | Disk diffusion test | | Uninformed | | CAM | | *B. subtilis* |  | 9.00 ± 1.00 |  |  |  |
|  |  |  |  |  |  |  |  |  | *S. aureus* ATCC 12398 |  | 13.67 ± 1.15 |  |  |  |
|  |  |  |  |  |  |  |  |  | *S. epidermidis* |  | 13.67 ± 0.58 |  |  |  |
|  |  |  |  |  |  |  |  |  | *S. lutea* |  | 11.00 ± 0.50 |  |  |  |
|  |  |  |  |  |  |  |  |  | *V. cholerae* No-01 ATCC 35971 |  | 8.00 ± 0.50 |  |  |  |
|  |  |  |  |  |  |  |  | |  | MIC (mg/mL) | MBC (mg/mL) |  |  |  |
|  | 159 | | MIC and MBC | | - | | - | | *B. subtilis* | 0.75 | >1.0 |  |  |  |
|  |  | |  |  |  | |  | | *S. aureus* ATCC 12398 | 0.5 | >1.0 |  |  |  |
|  |  | |  |  |  | |  | | *S. epidermidis* | 0.75 | >1.0 |  |  |  |
|  |  | |  |  |  | |  | | *S. lutea* | 0.75 | >1.0 |  |  |  |
|  |  | |  |  |  | |  | | *V. cholerae* No-01 ATCC 35971 | 0.5 | 1.0 |  |  |  |
|  |  | |  | |  | |  | |  | MIC (μg/mL) | ZI (mm) |  |  | |
| *Alternanthera dentata* | AgNPs of AqE of leaves | | Agar diffusion method | | 20, 40, 60, 80 and 100 µg/ml | | - | | *E. faecalis* | - | NDNS | AgNPs were active against all the microorganisms evaluated. | [255] | |
|  |  |  |  |  |  |  |  |  | *E. coli* | - | NDNS |  |  |  |
|  |  |  |  |  |  |  |  |  | *K. pneumonia* | - | NDNS |  |  |  |
|  |  |  |  |  |  |  |  |  | *P. aeruginosa* | - | NDNS |  |  |  |
| *Alternanthera philoxeroides* | Fraction X of ME of leaves | | Disk diffusion test | | 20, 40 and 60 µg/ml | | Streptomycin disk | | *E. coli* | 11.23 ± 0.11 | 52.14 ± 0.18 (60 µg/ml) | Dose-dependent antimicrobial activity. | [141] | |
|  |  |  |  |  |  |  |  |  | *M. luteus* | 16.23 ± 0.23 | 34 ± 0.31(60 µg/ml) |  |  |  |
| *Alternanthera repens* | ME | | Disk diffusion test | | 2,5 mg | | Ampicillin and CAM | | *S. lutea* ATCC 40322 |  | NE | ME did not show activity against the microorganisms tested. | [216] | |
|  |  | |  | |  | |  |  | *B. vulgaris ATCC 6059* |  | NE |  |  | |
|  |  | |  | |  | |  |  | *Staph. aureus ATCC 6538P* |  | NE |  |  | |
|  |  | |  | |  | |  |  | *E. coli ETECS 1552* |  | NE |  |  | |
| *Alternanthera sessilis (Linn.)* | AgNPs of AqE of leaves | | Baur et al.’s method | | 100 µg/ml | | Uninformed | | *E. coli* | Uninformed | | Microorganisms sensitive to AgNPs | [60] | |
|  |  |  |  |  |  |  |  |  | *Staph. aureus* | Uninformed | |  |  |  |
| *Alternanthera sessilis* | ME of the whole plant | | Disc diffusion method | | 500 µg/disc | | Kanamycin | | *B. megaterium* |  | 6 | *S. paratyphi*, *S. typi*, *E. coli,* and *S. lutea* were strongly inhibited. | [245] | |
|  |  |  |  |  |  |  |  |  | *B. subtilis* |  | 7 |  |  |  |
|  |  |  |  |  |  |  |  |  | *E. coli* |  | 16 |  |  |  |
|  |  |  |  |  |  |  |  |  | *P. aeruginosa* |  | 6 |  |  |  |
|  |  |  |  |  |  |  |  |  | *S. paratyphi* |  | 21 |  |  |  |
|  |  |  |  |  |  |  |  |  | *S. typhi* |  | 20 |  |  |  |
|  |  |  |  |  |  |  |  |  | *S. boydii* |  | 6 |  |  |  |
|  |  |  |  |  |  |  |  |  | *S. lutea* |  | 13 |  |  |  |
|  |  |  |  |  |  |  |  |  | *S. dysenteriae* |  | 0 |  |  |  |
|  |  |  |  |  |  |  |  |  | *S. aureus* |  | 6 |  |  |  |
|  |  |  |  |  |  |  |  |  | *V. mimicus* |  | 0 |  |  |  |
|  |  |  |  |  |  |  |  |  | *V. parahemolyticus* |  | 6 |  |  |  |
| *Alternanthera sessilis* | AgNPs of AqE of leaves | | Agar-well  diffusion method | | 10, 40 and 60 μg | | Clarithromycin | | *Escherichia coli* |  | 60 μg: 12 | Gram negative microorganisms were more sensitive than gram positive. | [227] | |
|  |  |  |  |  |  |  |  |  | *S. aureus* |  | 60 μg: 11 |  |  |  |
|  |  |  |  |  |  |  |  |  | *P. aeruginosa* |  | 60 μg: 9 |  |  |  |
|  |  |  |  |  |  |  |  |  | *K. pneumoniae* |  | 60 μg: 12 |  |  |  |
|  |  |  |  |  |  |  |  |  | *K. oxytoca* |  | 60 μg: 13 |  |  |  |
|  |  |  |  |  |  |  |  |  | *A. baumanii* |  | 60 μg: 13 |  |  |  |
| *Alternanthera sessilis* | ZnONPs of leaves | | Microdilution broth  method (MIC) | | 1000 to 31.25 μg/mL | | MHB | | *E. coli* ATCC 25922 | 125 | Uninformed | In a dark test, ZnONPs showed antibacterial activity.  The mechanism of action of the nanoparticle may be related to the Zn²⁺ ions released by the ZnONPs complex. | [228] | |
|  |  |  |  |  |  |  |  |  | *B. subtilis* ATCC 23857 | 125 | Uninformed |  |  |  |
|  |  |  |  |  |  |  |  |  | *S. aureus* NCTC 6571 | >1000 | Uninformed |  |  |  |
|  |  |  |  |  |  |  |  |  | *P. aeruginosa* ATCC 27853 | 250 | Uninformed |  |  |  |
|  |  |  |  |  |  | |  |  | *P. aeruginosa* PAC023 | NDNS | Uninformed |  |  |  |
|  |  |  |  | |  | |  | | *P. aeruginosa* PAC041 | NDNS | Uninformed |  |  |  |
|  |  |  |  | |  | |  | | *P. aeruginosa* PAC032 | 250 | Uninformed |  |  |  |
|  |  |  |  | |  | |  | | *P. aeruginosa* PAC045 | 500 | Uninformed |  |  |  |
|  |  |  | Photo‑bactericidal effect | | Uninformed | | Uninformed | | *E. coli* ATCC 25922 | Uninformed | Uninformed | The ZnONPs complex caused a ↓ in the integrity of the bacterial membrane and was more active against *E. coli*. |  |  |
|  |  |  |  |  |  | |  |  | *B. subtilis* ATCC 23857 | Uninformed | Uninformed |  |  |  |
|  |  |  | Generation of ROS | | 125 and 250 μg/mL | | Uninformed | | *E. coli* ATCC 25922 | Uninformed | Uninformed | It was found that for *E. coli* and *B. subtilis*, treatment with ZnONPs under light conditions and at [] of 125 and 250 μg/mL caused a ↑ in the production of ROS. As for the dark condition, [] of 250 μg/mL was able to ↓ the production of ROS.  It was found that the action of the ZnONPs complex can vary from species to species. |  |  |
|  |  | |  |  |  |  |  |  | *B. subtilis* ATCC 23857 | Uninformed | Uninformed |  |  |  |
|  |  | |  |  |  |  |  |  | *P. aeruginosa* PAC023 | Uninformed | Uninformed |  |  |  |
|  |  | |  |  |  |  |  |  | *P. aeruginosa* PAC032 | Uninformed | Uninformed |  |  |  |
|  |  | |  |  |  |  |  |  | *P. aeruginosa* PAC041 | Uninformed | Uninformed |  |  |  |
|  |  | |  |  |  |  |  |  | *P. aeruginosa* PAC045 | Uninformed | Uninformed |  |  |  |
|  |  | | Detection of bacterial protein leakage | | 125 and 250 μg/mL | | BSA | | *E. coli* ATCC 25922 | Uninformed | Uninformed | It has been suggested that the mechanism of action of the ZnONPs complex is the destruction of the membrane and, consequently, the destruction of metabolic functions and the excretion of proteins by the bacteria. |  |  |
|  |  | |  |  |  |  |  |  | *B. subtilis* ATCC 23857 | Uninformed | Uninformed |  |  |  |
|  |  | |  |  |  |  |  |  | *P. aeruginosa* PAC041 | Uninformed | Uninformed |  |  |  |
|  |  | |  |  |  |  |  |  | *P. aeruginosa* PAC045 | Uninformed | Uninformed |  |  |  |
| *Alternanthera tenella* Colla | Different extracts of the whole plant or callus | | Agar-well  diffusion method (well technique in double layer) | | 5.0 mg/mL | | Gentamicin disk, and bacitracin. | | Gram-positive (fi fteen strains) and Gram-negative bacteria (four strains) | NDNS | NDNS | The organic extracts and EE from a callus culture and whole plant were considerably active against the microorganisms evaluated, but the AqE did not show appreciable activity.  None of the extracts evaluated showed activity against *E. faecalis* ATCC 10541, *E. coli* (strains ATCC 10538 and ec 26.1),  *P. aeruginosa* (strains ATCC 27853 and 290D).  None of the isolated compounds showed activity against *E. coli* ec 26.1, *P. aeruginosa* ATCC 27853 y *P. aeruginosa* 290D.  24 was the compound with the highest antimicrobial spectrum, since it was able to inhibit the growth of 16 of the 19 microorganisms evaluated. | [142] | |
|  |  |  |  |  |  |  |  |  |  | NDNS | NDNS |  |  | |
|  | 411 | | Agar-well  diffusion method (well technique in double layer) | | 50 and 500 μg/mL | | Gentamicin disk, and bacitracin. | | *S. mutans* ATCC 25175 | 500 | 7 |  |  | |
|  |  |  |  |  |  |  |  |  | *S. mutans* Fab 3 | 500 | 6 |  |  | |
|  |  |  |  |  |  |  |  |  | *S. mutans* 11.1 | >500 | 9 |  |  | |
|  |  |  |  |  |  |  |  |  | *S. mutans* 9.1 | 500 | 8 |  |  | |
|  |  |  |  |  |  |  |  |  | *S. mutans* 9.31 | 500 | 6 |  |  | |
|  |  |  |  |  |  |  |  |  | *S. mutans* 11.22.1 | 500 | 6 |  |  | |
|  |  |  |  |  |  |  |  |  | *S. sobrinus* 180.3 | 500 | 6 |  |  | |
|  |  |  |  |  |  |  |  |  | *E. coli ATCC 10538* | >500 | 6 |  |  | |
|  | 411 and 386 | | Agar-well  diffusion method (well technique in double layer) | | 50 and 500 μg/mL | | Gentamicin disk, and bacitracin. | | *K. rhizophila* | >500 | 7 |  |  | |
|  |  |  |  |  |  |  |  |  | *S. aureus* ATCC 6538 | >500 | 7 |  |  | |
|  |  |  |  |  |  |  |  |  | *S. aureus penicillinase* + (7+) | 500 | 6 |  |  | |
|  |  |  |  |  |  |  |  |  | *S. mutans* ATCC 25175 | 100 | 7 |  |  | |
|  |  |  |  |  |  |  |  |  | *S. mutans* Fab 3 | 100 | 6 |  |  | |
|  |  |  |  |  |  |  |  |  | *S. mutans* 11.1 | >500 | 7 |  |  | |
|  |  |  |  |  |  |  |  |  | *S. mutans* 9.1 | 500 | 8 |  |  | |
|  |  |  |  |  |  |  |  |  | *S. mutans* 9.31 | 500 | 7 |  |  | |
|  |  |  |  |  |  |  |  |  | *S. mutans* 11.22.1 | 500 | 8 |  |  | |
|  |  |  |  |  |  |  |  |  | *S. sobrinus* 180.3 | 100 | 7 |  |  | |
|  |  |  |  |  |  | |  |  | *E. coli ATCC 10538* | >500 | 7 |  |  | |
|  | 396 and 411 | | Agar-well  diffusion method (well technique in double layer) | | 50 and 500 μg/mL | | Gentamicin disk, and bacitracin. | | *K. rhizophila* | >500 | 8 |  |  | |
|  |  |  |  |  |  |  |  |  | *S. aureus* ATCC 6538 | >500 | 8 |  |  | |
|  |  |  |  |  |  |  |  |  | *S. aureus penicillinase* + (7+) | 500 | 6 |  |  | |
|  |  |  |  |  |  |  |  |  | *S. mutans* ATCC 25175 | 500 | 7 |  |  | |
|  |  |  |  |  |  |  |  |  | *S. mutans* Fab 3 | 500 | 7 |  |  | |
|  |  |  |  |  |  |  |  |  | *S. mutans* 11.1 | >500 | 7 |  |  | |
|  |  |  |  |  |  |  |  |  | *S. mutans* 9.1 | 500 | 9 |  |  | |
|  |  |  |  |  |  |  |  |  | *S. mutans* 9.31 | 500 | 6 |  |  | |
|  |  |  |  |  |  |  |  |  | *S. mutans* 11.22.1 | 500 | 9 |  |  | |
|  |  |  |  |  |  |  |  |  | *S. sobrinus* 180.3 | 100 | 9 |  |  | |
|  |  |  |  |  |  |  |  |  | *E. coli ATCC 10538* | >500 | 6 |  |  | |
|  | 415 and 416 | | Agar-well  diffusion method (well technique in double layer) | | 50 and 500 μg/mL | | Gentamicin disk, and bacitracin. | | *S. aureus* ATCC 6538 | >500 | 7 |  |  | |
|  |  |  |  |  |  |  |  |  | *S. aureus penicillinase* + (7+) | >500 | 6 |  |  | |
|  |  |  |  |  |  |  |  |  | *S. mutans* 11.1 | 500 | 6 |  |  | |
|  |  |  |  |  |  |  |  |  | *S. mutans* 9.1 | >500 | 7 |  |  | |
|  |  |  |  |  |  |  |  |  | *S. mutans* 11.22.1 | 500 | 6 |  |  | |
|  |  |  |  |  |  |  |  |  | *S. sobrinus* 180.3 | 100 | 6 |  |  | |
|  |  |  |  |  |  |  |  |  | *E. coli ATCC 10538* | >500 | 7 |  |  | |
|  | 416 | | Agar-well  diffusion method (well technique in double layer) | | 50 and 500 μg/mL | | Gentamicin disk, and bacitracin. | | *S. aureus* ATCC 6538 | >500 | 7 |  |  | |
|  |  |  |  |  |  |  |  |  | *S. aureus penicillinase* + (7+) | >500 | 6 |  |  | |
|  |  |  |  |  |  |  |  |  | *S. epidermis 6ep* | >500 | 6 |  |  | |
|  |  |  |  |  |  |  |  |  | *S. mutans* 11.1 | 500 | 6 |  |  | |
|  |  |  |  |  |  |  |  |  | *S. mutans* 9.1 | >500 | 7 |  |  | |
|  |  |  |  |  |  |  |  |  | *S. mutans* 11.22.1 | 500 | 7 |  |  | |
|  |  |  |  |  |  |  |  |  | *S. sobrinus* 180.3 | 100 | 6 |  |  | |
|  |  |  |  |  |  |  |  |  | *E. coli ATCC 10538* | >500 | 7 |  |  | |
|  | 394 and 414 | | Agar-well  diffusion method (well technique in double layer) | | 50 and 500 μg/mL | | Gentamicin disk, and bacitracin. | | *S. aureus* ATCC 6538 | >500 | 6 |  |  | |
|  |  |  |  |  |  |  |  |  | *S. mutans* Fab 3 | 500 | 6 |  |  | |
|  |  |  |  |  |  |  |  |  | *S. mutans* 9.1 | 100 | 7 |  |  | |
|  |  |  |  |  |  |  |  |  | *S. mutans* 11.22.1 | 500 | 7 |  |  | |
|  |  |  |  |  |  |  |  |  | *S. sobrinus* 180.3 | 500 | 6 |  |  | |
|  |  |  |  |  |  |  |  |  | *E. coli ATCC 10538* | >500 | 7 |  |  | |
|  | 20 | | Agar-well  diffusion method (well technique in double layer) | | 50 and 500 μg/mL | | Gentamicin disk, and bacitracin. | | *S. aureus* ATCC 6538 | 100 | 7 |  |  | |
|  |  | |  |  |  |  |  |  | *S. aureus* ATCC 25923 | 100 | 6 |  |  | |
|  |  | |  |  |  |  |  |  | *S. aureus* ATCC 25213 | 100 | 6 |  |  | |
|  |  | |  |  |  |  |  |  | *S. aureus penicillinase* + (7+) | 500 | 6 |  |  | |
|  |  | |  |  |  |  |  |  | *S. epidermis 6ep* | 100 | 6 |  |  | |
|  |  | |  |  |  |  |  |  | *S. mutans* 11.1 | 500 | 7 |  |  | |
|  |  | |  |  |  |  |  |  | *S. mutans* 9.1 | 500 | 6 |  |  | |
|  |  | |  |  |  |  |  |  | *S. mutans* 9.31 | 100 | 6 |  |  | |
|  |  | |  |  |  |  |  |  | *S. mutans* 11.22.1 | 500 | 7 |  |  | |
|  |  | |  |  |  |  |  |  | *S. sobrinus* 180.3 | 50 | 6 |  |  | |
|  |  | |  |  |  |  |  |  | *E. faecalis ATCC 10541* | 500 | 6 |  |  | |
|  |  | |  |  |  |  |  |  | *E. coli ATCC 10538* | >500 | 9 |  |  | |
|  | 25 | | Agar-well  diffusion method (well technique in double layer) | | 50 and 500 μg/mL | | Gentamicin disk, and bacitracin. | | *S. aureus* ATCC 6538 | 100 | 6 |  |  | |
|  |  |  |  |  |  |  |  |  | *S. aureus* ATCC 25213 | 100 | 6 |  |  | |
|  |  |  |  |  |  |  |  |  | *S. aureus penicillinase* + (7+) | 500 | 6 |  |  | |
|  |  |  |  |  |  |  |  |  | *S. aureus penicillinase* - (8-) | 100 | 7 |  |  | |
|  |  |  |  |  |  |  |  |  | *S. epidermis 6ep* | 100 | 6 |  |  | |
|  |  |  |  |  |  |  |  |  | *S. mutans* 11.1 | 500 | 6 |  |  | |
|  |  |  |  |  |  |  |  |  | *S. mutans* 9.1 | 100 | 6 |  |  | |
|  |  |  |  |  |  |  |  |  | *S. mutans* 9.31 | 500 | 6 |  |  | |
|  |  |  |  |  |  |  |  |  | *S. mutans* 11.22.1 | 500 | 6 |  |  | |
|  |  |  |  |  |  |  |  |  | *S. sobrinus* 180.3 | 50 | 7 |  |  | |
|  |  |  |  |  |  |  |  |  | *E. faecalis ATCC 10541* | 500 | 7 |  |  | |
|  |  |  |  |  |  | |  | | *E. coli ATCC 10538* | >500 | 9 |  |  | |
|  | 24 | | Agar-well  diffusion method (well technique in double layer) | | 50 and 500 μg/mL | | Gentamicin disk, and bacitracin. | | *K. rhizophila* | 500 | 7 |  |  | |
|  |  |  |  |  |  |  |  |  | *S. aureus* ATCC 6538 | 100 | 6 |  |  | |
|  |  |  |  |  |  |  |  |  | *S. aureus* ATCC 25923 | 100 | 6 |  |  | |
|  |  |  |  |  |  |  |  |  | *S. aureus* ATCC 25213 | 100 | 7 |  |  | |
|  |  |  |  |  |  |  |  |  | *S. aureus penicillinase* + (7+) | 100 | 7 |  |  | |
|  |  |  |  |  |  |  |  |  | *S. aureus penicillinase* - (8-) | 100 | 6 |  |  | |
|  |  |  |  |  |  |  |  |  | *S. epidermis 6ep* | 100 | 8 |  |  | |
|  |  |  |  |  |  |  |  |  | *S. mutans* ATCC 25175 | 500 | 6 |  |  | |
|  |  |  |  | |  |  |  |  | *S. mutans* Fab 3 | 50 | 6 |  |  | |
|  |  |  |  | |  |  |  |  | *S. mutans* 11.1 | 100 | 6 |  |  | |
|  |  |  |  | |  |  |  | | *S. mutans* 9.1 | >500 | 6 |  |  | |
|  |  |  |  | |  | |  | | *S. mutans* 9.31 | 100 | 6 |  |  | |
|  |  | |  | |  | |  | | *S. mutans* 11.22.1 | 500 | 6 |  |  | |
|  |  | |  | |  | |  | | *S. sobrinus* 180.3 | 100 | 7 |  |  | |
|  |  | |  | |  | |  | | *E. faecalis ATCC 10541* | 100 | 7 |  |  | |
|  |  | |  | |  | |  | | *E. coli ATCC 10538* | >500 | 7 |  |  | |
|  | 88 | | Agar-well  diffusion method (well technique in double layer) | | 50 and 500 μg/mL | | Gentamicin disk, and bacitracin. | | *S. aureus* ATCC 6538 | 50 | 6 |  |  | |
|  |  |  |  |  |  |  |  |  | *S. aureus* ATCC 25923 | 100 | 7 |  |  | |
|  |  |  |  |  |  |  |  |  | *S. aureus penicillinase* + (7+) | 500 | 6 |  |  | |
|  |  |  |  |  |  |  |  |  | *S. mutans* 11.1 | >500 | 6 |  |  | |
|  |  |  |  |  |  |  |  |  | *S. mutans* 9.31 | >500 | 6 |  |  | |
|  |  |  |  |  |  |  |  |  | *S. mutans* 11.22.1 | >500 | 9 |  |  | |
|  |  |  |  |  |  |  |  |  | *E. coli ATCC 10538* | >500 | 9 |  |  | |
|  | 72 | | Agar-well  diffusion method (well technique in double layer) | | 50 and 500 μg/mL | | Gentamicin disk, and bacitracin. | | *S. aureus* ATCC 6538 | 500 | 6 |  |  | |
|  |  |  |  |  |  |  |  |  | *S. aureus* ATCC 25923 | 500 | 6 |  |  | |
|  | 43 | | Agar-well  diffusion method (well technique in double layer) | | 50 and 500 μg/mL | | Gentamicin disk, and bacitracin. | | *S. aureus* ATCC 6538 | 500 | 6 |  |  | |
|  |  |  |  |  |  |  |  |  | *S. aureus* ATCC 25923 | 500 | 6 |  |  | |
|  | 34 | | Agar-well  diffusion method (well technique in double layer) | | 50 and 500 μg/mL | | Gentamicin disk, and bacitracin. | | *S. aureus* ATCC 6538 | 500 | 6 |  |  | |
|  |  |  |  |  |  |  |  |  |  |  |  |  |  | |
|  |  |  |  |  |  |  |  |  | *S. aureus* ATCC 25923 | 500 | 6 |  |  | |
| *Blutaparon portulacoides* | EE of aerial parts | | Well diffusion method in double layer | | 5000 µg/ml | | Gentamicin | | *E. coli ATCC 10538* | - | 0 | The AqE of aerial parts and roots did not show antibacterial activity against any of the evaluated strains.  EE of aerial part was active against *S. aureus* (strain 7+ penicillinase producer), 4 strains of *S. mutans*, and *S. sobrinus* and EE of roots showed activity against *S. aureus* 7+, *S. mutans* 9.1 and S. sobrinus.  78 was active against 2 strains of *S. aureus*, 4 strains of *S. mutans* and *S. sobrinus*, with MICs ranging from 20 to 1250 µg/ml.  The mixture of acyl steryl glycosides (**395, 412**) was active against 2 strain of *S. aureus*, *S. epidermidis*, *E. coli*, *S. mutans* 9.1 and *S. sobrinus*; with MICs ranging from 50 and 500 µg/mL. | [151] | |
|  |  |  |  |  |  | |  | | *E. faecalis ATCC 10541* | - | 0 |  |  |  |
|  |  | |  |  |  | |  | | *M. luteus ATCC 9341* | - | 0 |  |  | |
|  |  | |  |  |  | |  | | *P. aeruginosa ATCC 27853* | - | 0 |  |  | |
|  |  | |  |  |  | |  | | *S. aureus ATCC 6538* | - | 0 |  |  | |
|  |  | |  |  |  | |  | | *S. aureus ATCC 25923* | - | 0 |  |  | |
|  |  | |  |  |  | |  | | *S. aureus 7+* | - | 15 |  |  | |
|  |  | |  |  |  | |  | | *S. aureus 8–* | - | 0 |  |  | |
|  |  | |  |  |  | |  | | *S. epidermis 6ep* | - | 0 |  |  | |
|  |  | |  |  |  | |  | | *S. mutans ATCC 25175* | - | 0 |  |  | |
|  |  | |  |  |  | |  | | *S. mutans 11.1* | - | 8 |  |  | |
|  |  | |  |  |  | |  | | *S. mutans 9.1* | - | 9 |  |  | |
|  |  | |  |  |  | |  | |  |  |  |  |  | |
|  |  | |  |  |  | |  | | *S. mutans 9.3* | - | 9 |  |  | |
|  |  | |  |  |  | |  | | *S. mutans 11.22.1* | - | 11 |  |  | |
|  |  | |  |  |  | |  | | *S. sobrinus 180.3* | - | 7 |  |  | |
|  | EE of roots | | Well diffusion method in double layer | | 5000 µg/ml | | Gentamicin | | *E. coli ATCC 10538* | - | 0 |  |  | |
|  |  | |  |  |  | |  | | *E. faecalis ATCC 10541* | - | 0 |  |  | |
|  |  | |  |  |  | |  | | *M. luteus ATCC 9341* | - | 0 |  |  | |
|  |  | |  |  |  | |  | | *P. aeruginosa ATCC 27853* | - | 0 |  |  | |
|  |  | |  |  |  | |  | | *S. aureus ATCC 6538* | - | 0 |  |  | |
|  |  | |  |  |  | |  | | *S. aureus ATCC 25923* | - | 0 |  |  | |
|  |  | |  |  |  | |  | | *S. aureus 7+* | - | 8 |  |  | |
|  |  | |  |  |  | |  | | *S. aureus 8–* | - | 0 |  |  | |
|  |  | |  |  |  | |  | | *S. epidermis 6ep* | - | 0 |  |  | |
|  |  | |  |  |  | |  | | *S. mutans ATCC 25175* | - | 0 |  |  | |
|  |  | |  |  |  | |  | | *S. mutans 11.1* | - | 0 |  |  | |
|  |  | |  |  |  | |  | | *S. mutans 9.1* | - | 8 |  |  | |
|  |  | |  |  |  | |  | | *S. mutans 9.3* | - | 0 |  |  | |
|  |  | |  |  |  | |  | | *S. mutans 11.22.1* | - | 0 |  |  | |
|  |  | |  |  |  | |  | | *S. sobrinus 180.3* | - | 7 |  |  | |
|  | 80 | | Well diffusion method in double layer and MIC | | 2500 µg/ml | | Gentamicin | | *E. coli ATCC 10538* | - | 0 |  |  | |
|  |  | |  |  |  | |  | | *E. faecalis ATCC 10541* | - | 0 |  |  | |
|  |  | |  |  |  | |  | | *M. luteus ATCC 9341* | - | 0 |  |  | |
|  |  | |  |  |  | |  | | *P. aeruginosa ATCC 27853* | - | 0 |  |  | |
|  |  | |  |  |  | |  | | *S. aureus ATCC 6538* | 1250 | 6 |  |  | |
|  |  | |  |  |  | |  | | *S. aureus ATCC 25923* | - | 0 |  |  | |
|  |  | |  |  |  | |  | | *S. aureus 7+* | 160 | 12 |  |  | |
|  |  | |  |  |  | |  | | *S. aureus 8–* | - | 0 |  |  | |
|  |  | |  |  |  | |  | | *S. epidermis 6ep* | - | 0 |  |  | |
|  |  | |  |  |  | |  | | *S. mutans ATCC 25175* | 80 | 9 |  |  | |
|  |  | |  |  |  | |  | | *S. mutans 11.1* | 80 | 10 |  |  | |
|  |  | |  |  |  | |  | | *S. mutans 9.1* | 40 | 8 |  |  | |
|  |  | |  |  |  | |  | | *S. mutans 9.3* | - | 0 |  |  | |
|  |  | |  |  |  | |  | | *S. mutans 11.22.1* | 20 | 7 |  |  | |
|  |  | |  |  |  | |  | | *S. sobrinus 180.3* | 160 | 6 |  |  | |
|  | 395 and 412 | | Well diffusion method in double layer and MIC | | 2500 µg/ml | | Gentamicin | | *E. coli ATCC 10538* | 50 | 9 |  |  | |
|  |  | |  |  |  | |  | | *E. faecalis ATCC 10541* | - | 0 |  |  | |
|  |  | |  |  |  | |  | | *M. luteus ATCC 9341* | - | 0 |  |  | |
|  |  | |  |  |  | |  | | *P. aeruginosa ATCC 27853* | - | 0 |  |  | |
|  |  | |  |  |  | |  | | *S. aureus ATCC 6538* | - | 0 |  |  | |
|  |  | |  |  |  | |  | | *S. aureus ATCC 25923* | 50 | 7 |  |  | |
|  |  | |  |  |  | |  | | *S. aureus 7+* | 50 | 7 |  |  | |
|  |  | |  |  |  | |  | | *S. aureus 8–* | - |  |  |  | |
|  |  | |  |  |  | |  | | *S. epidermis 6ep* | 500 | 6 |  |  | |
|  |  | |  |  |  | |  | | *S. mutans ATCC 25175* | - | 0 |  |  | |
|  |  | |  |  |  | |  | | *S. mutans 11.1* | - | 0 |  |  | |
|  |  | |  |  |  | |  | | *S. mutans 9.1* | 500 | 6 |  |  | |
|  |  | |  |  |  | |  | | *S. mutans 9.3* | - | 0 |  |  | |
|  |  | |  | |  | |  | | *S. mutans 11.22.1* | - | 0 |  |  | |
|  |  | |  | |  | |  | | *S. sobrinus 180.3* | 500 | 6 |  |  | |
| *Blutaparon portulacoides* | EE of stems | | Resazurin Microtiter Assay Plate | | 0.98–250 μg/mL | | Isoniazid | | *M. tuberculosis ATCC27294* | 123.4 | - | EE was not active against *E. aerogenes* and *S. saprophyticus*, but it proved to be efficient against *M. tuberculosis, B. cepacian* and *S. typhimurium* | [71] | |
|  |  | | Plate microdilution method | | Variable concentration | | - | | *B. cepacia (ATCC25416)* | 1000 | - |  |  | |
|  |  | |  |  |  |  |  |  | *E. aerogenes ATCC13048* | - | - |  |  | |
|  |  | |  |  |  |  |  |  | *S. typhimurium ATCC14028* | 1000 | - |  |  | |
|  |  | |  |  |  |  |  |  | *S. saprophyticus*  *ATCC15305* | - | - |  |  | |
|  |  | |  | |  | |  | |  | MIC mg/mL (mM) | ZI (mm) |  |  | |
| *Gomphrena agrestis* | EE of Whole plant | | Modified agar-well diffusion method (well technique in double layer) and MIC | | For ZI: 1 mg/mL  For MIC: range 0.02 to 0.5 mg/ml | | Bacitracine | | *P. aeruginosa ATTC 27853* |  | 7 | They were evaluated against 19 bacteria, but the EE and the isolated compounds showed activity only against 5 or 4 bacteria.  None showed activity against the following strains: *M. luteus* ATTC 9341; *Staph. Aureus stains:* ATCC 6538, ATTC 25213 and penicilinase - (8-); *Strept. mutans* strains: ATTC 25175, Fab 3, 11.1, 9.1, and 11.22.1; *Strept. sobrinus* 180.3; *E. faecalis* ATTC 10541; E. coli ATTC 10538 and ec 26.1 | [2] | |
|  |  | |  |  |  |  |  | | *P. aeruginosa 290D* |  | 6 |  |  |  |
|  |  | |  |  |  |  |  | | *Staph. aureus ATTC 25923* |  | 8 |  |  |  |
|  |  | |  |  |  |  |  | | *Staph. epidermidis 6epi* |  | 6 |  |  |  |
|  |  | |  |  |  |  |  | | *Staph. epidermidis epiC* |  | 6 |  |  |  |
|  | 327 | | Modified agar-well diffusion method (well technique in double layer) and MIC | | For ZI: 1 mg/mL  For MIC: range 0.02 to 0.5 mg/ml | | Bacitracine | | *P. aeruginosa ATTC 27853* | 0.5 (1.1) | 7 |  |  |  |
|  |  |  |  |  |  |  |  | | *P. aeruginosa 290D* | 0.5 (1.1) | 6 |  |  |  |
|  |  |  |  |  |  |  |  | | *Staph. aureus ATTC 25923* | 0.1 (0.2) | 8 |  |  |  |
|  |  |  |  |  |  |  |  | | *Staph. epidermidis 6epi* | 0.5 (1.1) | 6 |  |  |  |
|  |  |  |  |  |  |  |  | | *Staph. epidermidis epiC* | 0.5 (1.1) | 6 |  |  |  |
|  | 33 | | Modified agar-well diffusion method (well technique in double layer) and MIC | | For ZI: 1 mg/mL  For MIC: range 0.02 to 0.5 mg/ml | | Bacitracine | | *P. aeruginosa ATTC 27853* | 0.5 (1.0) | 6 |  |  |  |
|  |  |  |  |  |  |  |  | | *P. aeruginosa 290D* | 0.5 (1.0) | 6 |  |  |  |
|  |  |  |  |  |  |  |  | | *Staph. aureus ATTC 25923* | > 0.5 (1.0) | 6 |  |  |  |
|  |  |  |  |  |  |  |  | | *Staph. epidermidis 6epi* | 0.1 (0.2) | 7 |  |  |  |
|  |  |  |  |  |  |  |  | | *Staph. epidermidis epiC* | 0.5 (1.0) | 6 |  |  |  |
|  | 106 | | Modified agar-well diffusion method (well technique in double layer) and MIC | | For ZI: 1 mg/mL  For MIC: range 0.02 to 0.5 mg/ml | | Bacitracine | | *P. aeruginosa ATTC 27853* | > 0.5 (0.8) | 6 |  |  |  |
|  |  | |  |  |  |  |  | | *Staph. aureus ATTC 25923* | 0.5 (0.8) | 6 |  |  |  |
|  |  | |  |  |  |  |  | | *Staph. aureus penicilinase + (7+)* | 0.5 (0.8) | 6 |  |  |  |
|  |  | |  |  |  |  |  | | *Staph. epidermidis (6epi)* | > 0.5 (0.8) | 6 |  |  |  |
|  |  | |  | |  | |  | |  | MIC (µg/mL) | ZI (mm) |  |  | |
| *Gomphrena boliviana* | EE of whole plant | | Agar dilution technique | | 4000, 2000, 1000, 500, 250 and 125 µg/mL | |  | | *C. butyricum* | 4000 |  | None of the extracts showed activity against: *E. aerogenes*, *S. newport*, *S. oranienburg*, *E. coli* B, *E. coli* K 12, *K. pneumoniae*, *S. marcescens*, *P. aeruginosa* and *P. vulgaris,* and additionally EE did not show activity against *B. subtilis* and PEE against S. faecalis.  PEE showed strong activity against: *C. tetanii*, *C. sporogenes* and *M. phlei*. | [75] | |
|  |  |  |  |  |  |  |  | | *C. sporogenes* | 4000 |  |  |  |  |
|  |  |  |  |  |  |  |  | | *C. tetanii* | 1000 |  |  |  |  |
|  |  |  |  |  |  |  |  | | *M. luteus* | 2000 |  |  |  |  |
|  |  |  |  |  |  |  |  | | *M. phlei* | 250 |  |  |  |  |
|  |  |  |  |  |  |  |  | | *Staph. aureus ATTC* *12600* | 2000 |  |  |  |  |
|  |  |  |  |  |  |  |  | | *S. faecalis ATCC 19433* | 2000 |  |  |  |  |
|  | PEE of whole plant | | Agar dilution technique | | 4000, 2000, 1000, 500, 250 and 125 µg/mL | |  | | *B. subtilis* | 1000 |  |  |  |  |
|  |  |  |  |  |  |  |  | | *C. butyricum* | 1000 |  |  |  |  |
|  |  |  |  |  |  |  |  | | *C. sporogenes* | 250 |  |  |  |  |
|  |  |  |  |  |  |  |  | | *C. tetanii* | 250 |  |  |  |  |
|  |  |  |  |  |  |  |  | | *M. luteus* | 500 |  |  |  |  |
|  |  |  |  |  |  |  |  | | *M. phlei* | <50 |  |  |  |  |
|  |  |  |  |  |  |  |  | | *Staph. aureus ATTC* *12600* | 1000 |  |  |  |  |
| *Gomphrena boliviana and Gomphrena martiana* | 10 | | Agar dilution technique | | 100, 75, 50, 30, 20, 10 µg/mL | | Streptomycin sulfate | | *M. phlei* | 20 |  | All compounds showed strong activity against *M. phlei.* | [75] | |
|  |  |  |  |  |  |  |  |  | *Staph. aureus ATTC 12600* | 2000 |  |  |  |  |
|  |  |  |  |  |  |  |  |  | *S. faecalis ATCC 19433* | 2000 |  |  |  |  |
|  | 6 | |  |  |  |  |  |  | *M. phlei* | 15 |  |  |  |  |
|  |  |  |  |  |  |  |  | | *Staph. aureus ATTC* *12600* | 500 |  |  |  |  |
|  |  |  |  | |  |  |  | | *S. faecalis ATCC 19433* | 2000 |  |  |  |  |
|  | 7 | |  | |  | |  | | *M. phlei* | 15 |  |  |  |  |
|  |  |  |  | |  | |  | | *Staph. aureus ATTC* *12600* | 500 |  |  |  |  |
|  |  |  |  | |  | |  | | *S. faecalis ATCC 19433* | 2000 |  |  |  |  |
|  | 17 | |  | |  | |  | | *M. phlei* | 20 |  |  |  |  |
|  |  |  |  | |  | |  | | *Staph. aureus ATTC* *12600* | 2000 |  |  |  |  |
|  |  |  |  | |  | |  | | *S. faecalis ATCC 19433* | 2000 |  |  |  |  |
|  | 9 | |  | |  | |  | | *M. phlei* | 75 |  |  |  |  |
|  |  |  |  | |  | |  | | *Staph. aureus ATTC* *12600* | 2000 |  |  |  |  |
|  |  |  |  |  |  |  |  | | *S. faecalis ATCC 19433* | 2000 |  |  |  |  |
|  |  |  |  |  |  |  |  |  |  |  |  |  |  |  |
| *Gomphrena celosioides* | EE of aerial parts | | Kirby-Bauer method | | 0.2 mg/disc | | - | | *S. typhi ATCC 19430* |  | 7.3 ± 0.5 | The extract and the isolated compounds did not show activity against: *E. coli* ATCC 8739, *P. mirabilis* ATCC 15290, and *P. aeruginosa* ATCC 15442 | [157] | |
|  |  |  |  |  |  | |  | | *S. aureus ATCC 12598* |  | 7.6 ± 2.5 |  |  |  |
|  | 156 | | Kirby-Bauer method | | 0.2 mg/disc | | - | | *S. typhi ATCC 19430* |  | 7.3 ± 0.5 |  |  |  |
|  |  |  |  |  |  | |  | | *S. aureus ATCC 12598* |  | 7.3 ± 2.5 |  |  |  |
|  | 417 | | Kirby-Bauer method | | 0.2 mg/disc | | - | | *S. typhi ATCC 19430* |  | 7.3 ± 0.5 |  |  |  |
|  |  |  |  |  |  | |  | | *S. aureus ATCC 12598* |  | 7.6 ± 2.5 |  |  |  |
|  |  | |  | |  | |  | |  | MIC (mg/mL) | ZI (mm) |  |  | |
| *Gomphrena celosioides* | EaE of Whole planta | | Agar cup diffusion method | | 12.5 mg/mL | | Ampicilin | | *B. subtilis* |  | 14 ± 0.4 | The EaE presented greater activity than the isolated compound and ME.  The ↑ activity of the extracts may be due to synergistic activities between the compounds and to a ↑ amount of OH. | [82] | |
|  |  |  |  |  |  |  |  | | *E. coli* NCTC9001 |  | 12 ± 0.4 |  |  |  |
|  |  |  |  |  |  |  |  | | *P. aeruginosa* NCTC6750 |  | 12 ± 0.7 |  |  |  |
|  |  |  |  |  |  |  |  | | *S. typhi* ATCC14028 |  | 12 ± 0.3 |  |  |  |
|  |  |  |  |  |  |  |  | | *Staph. aureus* NCTC6571 |  | 13 ± 0.3 |  |  |  |
|  | ME of Whole planta | | Agar cup diffusion method | | 12.5 mg/mL | | Ampicilin | | *B. subtilis* |  | 13 ± 0.2 |  |  |  |
|  |  |  |  |  |  | |  | | *E. coli* NCTC9001 |  | 12 ± 0.3 |  |  |  |
|  |  |  |  |  |  | |  | | *P. aeruginosa* NCTC6750 |  | 12 ± 0.1 |  |  |  |
|  |  |  |  |  |  | |  | | *S. typhi* ATCC14028 |  | 12 ± 0.3 |  |  |  |
|  |  |  |  |  |  | |  | | *Staph. aureus* NCTC6571 |  | 13 ± 0.2 |  |  |  |
|  | 449 | | Agar cup diffusion method | | 25 µg/mL | | Ampicilin | | *B. subtilis* |  | 11 ± 0.7 |  |  |  |
|  |  |  |  |  |  |  |  | | *E. coli* NCTC9001 |  | 9 ± 0.2 |  |  |  |
|  |  |  |  |  |  |  |  | | *P. aeruginosa* NCTC6750 |  | 10 ± 0.5 |  |  |  |
|  |  |  |  |  |  |  |  | | *S. typhi* ATCC14028 |  | 9 ± 0.2 |  |  |  |
|  |  |  |  |  |  |  |  | | *Staph. aureus* NCTC6571 |  | 10 ± 0.4 |  |  |  |
| *Gomphrena celosioides* | AuNPs of extract of leaves | | Disc diffusion method | | Uninformed | | CAM and Streptomycin | | *P. pneumonia* |  | 17.56 ±0.30 | The activity was comparable to that of the positive controls. | [229] | |
|  |  |  |  |  |  |  |  |  | *S. typhi* |  | 14.67 ±0.30 |  |  |  |
|  |  |  |  |  |  |  |  |  | *Staph. aureus* |  | 16.65 ±0.50 |  |  |  |
|  |  | |  | |  | |  | |  | MIC  (mg/mL) | TAA (ml/g) |  |  | |
| *Gomphrena celosioides* | AcE of flower | | The broth microdilution test, bacteriostatic activity,  MIC, proliferation assay, biofilm inhibition assay | | Range from 2.5 to 0.04 mg/mL | | Ciprofloxacin | | *E. aerogenes*  ATCC 35029 | 0.16 | 25 | The aqueous extracts did not show bacteriostatic activity.  The AcE of leaves and mixture of aerial parts showed dose-dependent activity for *E. aerogenes* and *E. coli.*  All extracts tested inhibited biofilm formation above 50 %. | [89] | |
|  |  |  |  |  |  |  |  |  | *E. coli* ATCC 25218 | 0.08 | 50 |  |  |  |
|  |  |  |  |  |  |  |  |  | *K. pneumoniae* ATCC 700603 | 0.08 | 50 |  |  |  |
|  |  |  |  |  |  |  |  |  | *S. aureus* ATCC 11632 | 0.16 | 25 |  |  |  |
|  | AcE of leaves | |  |  |  |  |  |  | *E. aerogenes*  ATCC 35029 | 0.16 | 181.25 |  |  |  |
|  |  |  |  |  |  |  |  |  | *E. coli ATCC 25218* | 0.16 | 181.25 |  |  |  |
|  |  |  |  |  |  |  |  |  | *K. pneumoniae* ATCC 700603 | 0.16 | 181.25 |  |  |  |
|  |  |  |  |  |  |  |  |  | *S. aureus* ATCC 11632 | 0.16 | 181.25 |  |  |  |
|  | AcE of twigs | |  |  |  |  |  |  | *E. aerogenes*  ATCC 35029 | 0.08 | 175 |  |  |  |
|  |  |  |  |  |  |  |  |  | *E. coli* ATCC 25218 | 0.16 | 87.50 |  |  |  |
|  |  |  |  |  |  |  |  |  | *K. pneumoniae* ATCC 700603 | 0.04 | 350 |  |  |  |
|  |  |  |  |  |  |  |  |  | *S. aureus* ATCC 11632 | 0.31 | 45.16 |  |  |  |
|  | AcE of leaves, flowers and twigs combined | |  |  |  |  |  |  | *E. aerogenes*  ATCC 35029 | 0.08 | 1512.25 |  |  |  |
|  |  |  |  |  |  |  |  |  | *E. coli ATCC 25218* | 0.08 | 1512.25 |  |  |  |
|  |  |  |  |  |  |  |  |  | *K. pneumoniae* ATCC 700603 | 0.08 | 1512.25 |  |  |  |
|  |  |  |  |  |  |  |  |  | *S. aureus* ATCC 11632 | 0.08 | 1512.25 |  |  |  |
|  | AqE of flower | |  |  |  |  |  |  | *E. aerogenes*  ATCC 35029 | >2.5 | NA |  |  |  |
|  |  |  |  |  |  |  |  |  | *E. coli ATCC 25218* | >2.5 | NA |  |  |  |
|  |  |  |  |  |  |  |  |  | *K. pneumoniae* ATCC 700603 | >2.5 | NA |  |  |  |
|  |  |  |  |  |  |  |  |  | *S. aureus* ATCC 11632 | >2.5 | NA |  |  |  |
|  | AqE of leaves | |  |  |  |  |  |  | *E. aerogenes*  ATCC 35029 | >2.5 | NA |  |  |  |
|  |  |  |  |  |  |  |  |  | *E. coli ATCC 25218* | >2.5 | NA |  |  |  |
|  |  |  |  |  |  |  |  |  | *K. pneumoniae* ATCC 700603 | >2.5 | NA |  |  |  |
|  |  |  |  |  |  |  |  |  | *S. aureus* ATCC 11632 | >2.5 | NA |  |  |  |
|  | AqE of twigs | |  |  |  |  |  |  | *E. aerogenes*  ATCC 35029 | >2.5 | NA |  |  |  |
|  |  |  |  |  |  |  |  |  | *E. coli ATCC 25218* | >2.5 | NA |  |  |  |
|  |  |  |  |  |  |  |  |  | *K. pneumoniae* ATCC 700603 | >2.5 | NA |  |  |  |
|  |  |  |  |  |  |  |  |  | *S. aureus* ATCC 11632 | >2.5 | NA |  |  |  |
|  | AqE of leaves, flowers and twigs combined | |  |  |  |  |  |  | *E. aerogenes*  ATCC 35029 | >2.5 | NA |  |  |  |
|  |  |  |  |  |  |  |  |  | *E. coli ATCC 25218* | >2.5 | NA |  |  |  |
|  |  |  |  |  |  |  |  |  | *K. pneumoniae* ATCC 700603 | >2.5 | NA |  |  |  |
|  |  |  |  |  |  |  |  |  | *S. aureus* ATCC 11632 | >2.5 | NA |  |  |  |
|  |  | |  | |  | |  | |  | MIC (mg/mL) | MBC (mg/mL) |  |  | |
| *Gomphrena globosa* | AcE of flowers | | Broth microdilution method (MIC, and MBC) | | Uninformed | | Ciprofloxacin | | *B. bronchiseptica ATCC 4617* | 1.5 | 3.0 | AcE showed moderate and good activity against Gram positive bacteria, and weak activity against Gram negative bacteria.  Gram-positive pathogens were shown to be more susceptible than gram-negative pathogens to the action of the F1-F7.  The fractions showed greater activity than AcE, and the isolated compounds showed greater activity than the fractions. | [173] | |
|  |  |  |  |  |  |  |  |  | *B. cereus ATCC 10876* | 1.5 | >3.0 |  |  |  |
|  |  |  |  |  |  |  |  |  | *B. subtilis ATCC 6633* | 1.5 | 3.0 |  |  |  |
|  |  |  |  |  |  |  |  |  | *E. cloaceae* | 1.5 | >3.0 |  |  |  |
|  |  |  |  |  |  |  |  |  | *E. coli ATCC 25922* | 1.5 | 3.0 |  |  |  |
|  |  |  |  |  |  |  |  |  | *E. coli ATCC 35218* | 3.0 | > 3.0 |  |  |  |
|  |  |  |  |  |  |  |  |  | *K. pneumoniae ATCC 13883* | 1.5 | 3.0 |  |  |  |
|  |  |  |  |  |  |  |  |  | *M. luteus ATCC 10240* | 0.775 | 1.5 |  |  |  |
|  |  |  |  |  |  |  |  |  | *P. aeruginosa ATCC 27853* | 1.5 | 3.0 |  |  |  |
|  |  |  |  |  |  |  |  |  | *P. mirabilis ATCC 12453* | 1.5 | >3.0 |  |  |  |
|  |  |  |  |  |  |  |  |  | *S. aureus ATCC 25923* | 0.187 | 3.0 |  |  |  |
|  |  |  |  |  |  |  |  |  | *S. aureus ATCC 43300* | 0.75 | 3.0 |  |  |  |
|  |  |  |  |  |  |  |  |  | *S. aureus ATCC 6538* | 1.5 | 3.0 |  |  |  |
|  |  |  |  |  |  |  |  |  | *S. epidermidis ATCC 12228* | 1.5 | 3.0 |  |  |  |
|  |  |  |  |  |  |  |  |  | *S. typhimurium ATCC 14028* | 3.0 | > 3.0 |  |  |  |
|  |  |  |  |  |  |  |  |  | *S. sonnei* | 1.5 | 3.0 |  |  |  |
|  | 303, 308 and unknown pigments. (F1) | | Broth microdilution method (MIC, and MBC) | | Uninformed | | Ciprofloxacin | | *B. bronchiseptica ATCC 4617* | 1.5 | 3.0 |  |  | |
|  |  |  |  |  |  |  |  |  | *B. cereus ATCC 10876* | 1.5 | >3.0 |  |  | |
|  |  |  |  |  |  |  |  |  | *B. subtilis ATCC 6633* | 0.75 | 3.0 |  |  | |
|  |  |  |  |  |  |  |  |  | *E. cloaceae* | 3.0 | 3.0 |  |  | |
|  |  |  |  |  |  |  |  |  | *E. coli ATCC 25922* | 3.0 | 3.0 |  |  | |
|  |  |  |  |  |  |  |  |  | *E. coli ATCC 35218* | 3.0 | 3.0 |  |  | |
|  |  |  |  |  |  |  |  |  | *K. pneumoniae ATCC 13883* | 3.0 | 3.0 |  |  | |
|  |  |  |  |  |  |  |  |  | *M. luteus ATCC 10240* | 0.094 | 0.75 |  |  | |
|  |  |  |  |  |  |  |  |  | *P. aeruginosa ATCC 27853* | 3.0 | 3.0 |  |  | |
|  |  |  |  |  |  |  |  |  | *P. mirabilis ATCC 12453* | 3.0 | 3.0 |  |  | |
|  |  |  |  |  |  |  |  |  | *S. aureus ATCC 25923* | 3.0 | 3.0 |  |  | |
|  |  |  |  |  |  |  |  |  | *S. aureus ATCC 43300* | 3.0 | 3.0 |  |  | |
|  |  |  |  |  |  |  |  |  | *S. aureus ATCC 6538* | 1.5 | 3.0 |  |  | |
|  |  |  |  |  |  |  |  |  | *S. epidermidis ATCC 12228* | 1.5 | >3.0 |  |  | |
|  |  |  |  |  |  |  |  |  | *S. typhimurium ATCC 14028* | 3.0 | >3.0 |  |  | |
|  |  |  |  |  |  |  |  |  | *S. sonnei* | 0.75 | 3.0 |  |  | |
|  | 311 and 312 in a ratio 6.5**:**3.5. (F2) | | Broth microdilution method (MIC, and MBC) | | Uninformed | | Ciprofloxacin | | *B. bronchiseptica ATCC 4617* | 1.5 | 3.0 |  |  | |
|  |  |  |  |  |  |  |  |  | *B. cereus ATCC 10876* | 3.0 | >3.0 |  |  | |
|  |  |  |  |  |  |  |  |  | *B. subtilis ATCC 6633* | 1.5 | 3.0 |  |  | |
|  |  |  |  |  |  |  |  |  | *E. cloaceae* | 3.0 | >3.0 |  |  | |
|  |  |  |  |  |  |  |  |  | *E. coli ATCC 25922* | 3.0 | >3.0 |  |  | |
|  |  |  |  |  |  |  |  |  | *E. coli ATCC 35218* | 3.0 | >3.0 |  |  | |
|  |  |  |  |  |  |  |  |  | *K. pneumoniae ATCC 13883* | 3.0 | >3.0 |  |  | |
|  |  |  |  |  |  |  |  |  | *M. luteus ATCC 10240* | 0.187 | 1.5 |  |  | |
|  |  |  |  |  |  |  |  |  | *P. aeruginosa ATCC 27853* | 3.0 | 3.0 |  |  | |
|  |  |  |  |  |  |  |  |  | *P. mirabilis ATCC 12453* | 3.0 | >3.0 |  |  | |
|  |  |  |  |  |  |  |  |  | *S. aureus ATCC 25923* | 3.0 | >3.0 |  |  | |
|  |  |  |  |  |  |  |  |  | *S. aureus ATCC 43300* | 3.0 | >3.0 |  |  | |
|  |  |  |  |  |  |  |  |  | *S. aureus ATCC 6538* | 3.0 | >3.0 |  |  | |
|  |  |  |  |  |  |  |  |  | *S. epidermidis ATCC 12228* | 3.0 | >3.0 |  |  | |
|  |  |  |  |  |  |  |  |  | *S. typhimurium ATCC 14028* | 3.0 | >3.0 |  |  | |
|  |  |  |  |  |  |  |  |  | *S. sonnei* | 1.5 | 3.0 |  |  | |
|  | 299, 301, 311, 312, 305 and 310 in a ratio 0.7:0.2:2.1:0.6:3.8:2.6. (F3) | | Broth microdilution method (MIC, and MBC) | | Uninformed | | Ciprofloxacin | | *B. bronchiseptica ATCC 4617* | 1.5 | 3.0 |  |  | |
|  |  |  |  |  |  |  |  |  | *B. cereus ATCC 10876* | 3.0 | >3.0 |  |  | |
|  |  |  |  |  |  |  |  |  | *B. subtilis ATCC 6633* | 1.5 | 3.0 |  |  | |
|  |  |  |  |  |  |  |  |  | *E. cloaceae* | 3.0 | >3.0 |  |  | |
|  |  |  |  |  |  |  |  |  | *E. coli ATCC 25922* | 3.0 | >3.0 |  |  | |
|  |  |  |  |  |  |  |  |  | *E. coli ATCC 35218* | 3.0 | 3.0 |  |  | |
|  |  |  |  |  |  |  |  |  | *K. pneumoniae ATCC 13883* | 3.0 | >3.0 |  |  | |
|  |  |  |  |  |  |  |  |  | *M. luteus ATCC 10240* | 0. 375 | 1.5 |  |  | |
|  |  |  |  |  |  |  |  |  | *P. aeruginosa ATCC 27853* | 3.0 | 3.0 |  |  | |
|  |  |  |  |  |  |  |  |  | *P. mirabilis ATCC 12453* | 3.0 | >3.0 |  |  | |
|  |  |  |  |  |  |  |  |  | *S. aureus ATCC 25923* | 3.0 | >3.0 |  |  | |
|  |  |  |  |  |  |  |  |  | *S. aureus ATCC 43300* | >3.0 | >3.0 |  |  | |
|  |  |  |  |  |  |  |  |  | *S. aureus ATCC 6538* | 3.0 | 3.0 |  |  | |
|  |  |  |  |  |  |  |  |  | *S. epidermidis ATCC 12228* | 0.75 | 3.0 |  |  | |
|  |  |  |  |  |  |  |  |  | *S. typhimurium ATCC 14028* | 3.0 | >3.0 |  |  | |
|  |  |  |  |  |  |  |  |  | *S. sonnei* | 0.75 | 3.0 |  |  | |
|  | 309, 301, 305 and 310 in a ratio 1.9:1.6:4.3:2.2. (F4) | | Broth microdilution method (MIC, and MBC) | | Uninformed | | Ciprofloxacin | | *B. bronchiseptica ATCC 4617* | 0.75 | 1.5 |  |  | |
|  |  |  |  |  |  |  |  |  | *B. cereus ATCC 10876* | 1.5 | 3.0 |  |  | |
|  |  |  |  |  |  |  |  |  | *B. subtilis ATCC 6633* | 1.5 | 3.0 |  |  | |
|  |  |  |  |  |  |  |  |  | *E. cloaceae* | 3.0 | 3.0 |  |  | |
|  |  |  |  |  |  |  |  |  | *E. coli ATCC 25922* | 3.0 | 3.0 |  |  | |
|  |  |  |  |  |  |  |  |  | *E. coli ATCC 35218* | 3.0 | >3.0 |  |  | |
|  |  |  |  |  |  |  |  |  | *K. pneumoniae ATCC 13883* | 3.0 | 3.0 |  |  | |
|  |  |  |  |  |  |  |  |  | *M. luteus ATCC 10240* | 0.094 | 0.094 |  |  | |
|  |  |  |  |  |  |  |  |  | *P. aeruginosa ATCC 27853* | 3.0 | 3.0 |  |  | |
|  |  |  |  |  |  |  |  |  | *P. mirabilis ATCC 12453* | 3.0 | >3.0 |  |  | |
|  |  |  |  |  |  |  |  |  | *S. aureus ATCC 25923* | 3.0 | 3.0 |  |  | |
|  |  |  |  |  |  |  |  |  | *S. aureus ATCC 43300* | 3.0 | 3.0 |  |  | |
|  |  |  |  |  |  |  |  |  | *S. aureus ATCC 6538* | 3.0 | 3.0 |  |  | |
|  |  |  |  |  |  |  |  |  | *S. epidermidis ATCC 12228* | 3.0 | 3.0 |  |  | |
|  |  |  |  |  |  |  |  |  | *S. typhimurium ATCC 14028* | 3.0 | >3.0 |  |  | |
|  |  |  |  |  |  |  |  |  | *S. sonnei* | 1.5 | 1.5 |  |  | |
|  | 305 and 310 in a ratio 6.8:3.2. (F5) | | Broth microdilution method (MIC, and MBC) | | Uninformed | | Ciprofloxacin | | *B. bronchiseptica ATCC 4617* | 1.5 | 3.0 |  |  | |
|  |  |  |  |  |  |  |  |  | *B. cereus ATCC 10876* | 1.5 | 3.0 |  |  | |
|  |  |  |  |  |  |  |  |  | *B. subtilis ATCC 6633* | 3.0 | 3.0 |  |  | |
|  |  |  |  |  |  |  |  |  | *E. cloaceae* | 3.0 | 3.0 |  |  | |
|  |  |  |  |  |  |  |  |  | *E. coli ATCC 25922* | 3.0 | 3.0 |  |  | |
|  |  |  |  |  |  |  |  |  | *E. coli ATCC 35218* | 3.0 | >3.0 |  |  | |
|  |  |  |  |  |  |  |  |  | *K. pneumoniae ATCC 13883* | 3.0 | 3.0 |  |  | |
|  |  |  |  |  |  |  |  |  | *M. luteus ATCC 10240* | 0.187 | 0.187 |  |  | |
|  |  |  |  |  |  |  |  |  | *P. aeruginosa ATCC 27853* | 3.0 | 3.0 |  |  | |
|  |  |  |  |  |  |  |  |  | *P. mirabilis ATCC 12453* | 3.0 | >3.0 |  |  | |
|  |  |  |  |  |  |  |  |  | *S. aureus ATCC 25923* | 3.0 | 3.0 |  |  | |
|  |  |  |  |  |  |  |  |  | *S. aureus ATCC 43300* | 3.0 | 3.0 |  |  | |
|  |  |  |  |  |  |  |  |  | *S. aureus ATCC 6538* | 3.0 | 3.0 |  |  | |
|  |  |  |  |  |  |  |  |  | *S. epidermidis ATCC 12228* | 3.0 | >3.0 |  |  | |
|  |  |  |  |  |  |  |  |  | *S. typhimurium ATCC 14028* | 3.0 | >3.0 |  |  | |
|  |  |  |  |  |  |  |  |  | *S. sonnei* | 1.5 | 3.0 |  |  | |
|  | 298, 300, 304, 309, 305 and 310 in a ratio 0.7:0.4:1.5:3.0:1.8:2.6. (F6) | | Broth microdilution method (MIC, and MBC) | | Uninformed | | Ciprofloxacin | | *B. bronchiseptica ATCC 4617* | 0.187 | 0.75 |  |  | |
|  |  |  |  |  |  |  |  |  | *B. cereus ATCC 10876* | 0.75 | 1.5 |  |  | |
|  |  |  |  |  |  |  |  |  | *B. subtilis ATCC 6633* | 0.75 | 1.5 |  |  | |
|  |  |  |  |  |  |  |  |  | *E. cloaceae* | 1.5 | 1.5 |  |  | |
|  |  |  |  |  |  |  |  |  | *E. coli ATCC 25922* | 1.5 | 1.5 |  |  | |
|  |  |  |  |  |  |  |  |  | *E. coli ATCC 35218* | 1.5 | 1.5 |  |  | |
|  |  |  |  |  |  |  |  |  | *K. pneumoniae ATCC 13883* | 1.5 | 1.5 |  |  | |
|  |  |  |  |  |  |  |  |  | *M. luteus ATCC 10240* | 0.094 | 0.187 |  |  | |
|  |  |  |  |  |  |  |  |  | *P. aeruginosa ATCC 27853* | 0.75 | 1.5 |  |  | |
|  |  |  |  |  |  |  |  |  | *P. mirabilis ATCC 12453* | 1.5 | 1.5 |  |  | |
|  |  |  |  |  |  |  |  |  | *S. aureus ATCC 25923* | 1.5 | 1.5 |  |  | |
|  |  |  |  |  |  |  |  |  | *S. aureus ATCC 43300* | 1.5 | 3.0 |  |  | |
|  |  |  |  |  |  |  |  |  | *S. aureus ATCC 6538* | 1.5 | 1.5 |  |  | |
|  |  |  |  |  |  |  |  |  | *S. epidermidis ATCC 12228* | 1.5 | 3.0 |  |  | |
|  |  |  |  |  |  |  |  |  | *S. typhimurium ATCC 14028* | 1.5 | 1.5 |  |  | |
|  |  |  |  |  |  |  |  |  | *S. sonnei* | 0.75 | 0.75 |  |  | |
|  | 304 and 309 in a ratio 5.8:4.2. (F7) | | Broth microdilution method (MIC, and MBC) | | Uninformed | | Ciprofloxacin | | *B. bronchiseptica ATCC 4617* | 1.5 | 3.0 |  |  | |
|  |  |  |  |  |  |  |  |  | *B. cereus ATCC 10876* | 1.5 | >3.0 |  |  | |
|  |  |  |  |  |  |  |  |  | *B. subtilis ATCC 6633* | 1.5 | 3.0 |  |  | |
|  |  |  |  |  |  |  |  |  | *E. cloaceae* | 1.5 | >3.0 |  |  | |
|  |  |  |  |  |  |  |  |  | *E. coli ATCC 25922* | 1.5 | 3.0 |  |  | |
|  |  |  |  |  |  |  |  |  | *E. coli ATCC 35218* | 1.5 | 3.0 |  |  | |
|  |  |  |  |  |  |  |  |  | *K. pneumoniae ATCC 13883* | 1.5 | >3.0 |  |  | |
|  |  |  |  |  |  |  |  |  | *M. luteus ATCC 10240* | 1.5 | 3.0 |  |  | |
|  |  |  |  |  |  |  |  |  | *P. aeruginosa ATCC 27853* | 1.5 | 3.0 |  |  | |
|  |  |  |  |  |  |  |  |  | *P. mirabilis ATCC 12453* | 1.5 | >3.0 |  |  | |
|  |  |  |  |  |  |  |  |  | *S. aureus ATCC 25923* | 0.094 | 1.5 |  |  | |
|  |  |  |  |  |  |  |  |  | *S. aureus ATCC 43300* | 0.187 | 3.0 |  |  | |
|  |  |  |  |  |  |  |  |  | *S. aureus ATCC 6538* | 1.5 | 3.0 |  |  | |
|  |  |  |  |  |  |  |  |  | *S. epidermidis ATCC 12228* | 1.5 | 3.0 |  |  | |
|  |  |  |  |  |  |  |  |  | *S. typhimurium ATCC 14028* | 3.0 | 3.0 |  |  | |
|  |  |  |  |  |  |  |  |  | *S. sonnei* | 1.5 | 3.0 |  |  | |
|  | 303/308 | | Broth microdilution method (MIC, and MBC) | | Uninformed | | Ciprofloxacin | | *B. bronchiseptica ATCC 4617* | 1.5 | 1.5 |  |  | |
|  |  |  |  |  |  |  |  |  | *B. cereus ATCC 10876* | >1.0 | >1.0 |  |  | |
|  |  |  |  |  |  |  |  |  | *B. subtilis ATCC 6633* | 1.0 | >1.0 |  |  | |
|  |  |  |  |  |  |  |  |  | *E. coli ATCC 25922* | 1.5 | 1.5 |  |  | |
|  |  |  |  |  |  |  |  |  | *M. luteus ATCC 10240* | >1.0 | >1.0 |  |  | |
|  |  |  |  |  |  |  |  |  | *P. aeruginosa ATCC* *27853* | 1.5 | 1.5 |  |  | |
|  |  |  |  |  |  |  |  |  | *S. aureus ATCC 6538* | >1.0 | >1.0 |  |  | |
|  |  |  |  |  |  |  |  |  | *S. aureus ATCC 25923* | >1.0 | >1.0 |  |  | |
|  |  |  |  |  |  |  |  |  | *S. aureus ATCC 43300* | >1.0 | >1.0 |  |  | |
|  |  |  |  |  |  |  |  |  | *S. epidermidis ATCC 12228* | >1.0 | >1.0 |  |  | |
|  |  |  |  |  |  |  |  |  | *S. sonnei* | 0.75 | 3 |  |  | |
|  |  |  |  |  |  |  |  |  | *S. typhimurium ATCC 14028* | 1.5 | 3.0 |  |  | |
|  | 298 | | Broth microdilution method (MIC, and MBC) | | Uninformed | | Ciprofloxacin | | *B. bronchiseptica ATCC 4617* | 0.25 | 1.0 |  |  | |
|  |  |  |  |  |  |  |  |  | *B. cereus ATCC 10876* | 1.0 | >1.0 |  |  | |
|  |  |  |  |  |  |  |  |  | *B. subtilis ATCC 6633* | 0.5 | 1.0 |  |  | |
|  |  |  |  |  |  |  |  |  | *E. coli ATCC 25922* | 0.75 | 1.5 |  |  | |
|  |  |  |  |  |  |  |  |  | *M. Luteus ATCC 10240* | 0.5 | >1.0 |  |  | |
|  |  |  |  |  |  |  |  |  | *P. aeruginosa ATCC* *27853* | 0.75 | 1.5 |  |  | |
|  |  |  |  |  |  |  |  |  | *S. aureus ATCC 6538* | 1.0 | >1.0 |  |  | |
|  |  |  |  |  |  |  |  |  | *S. aureus ATCC 25923* | 1.0 | >1.0 |  |  | |
|  |  |  |  |  |  |  |  |  | *S. aureus ATCC 43300* | 1.0 | >1.0 |  |  | |
|  |  |  |  |  |  |  |  |  | *S. epidermidis ATCC 12228* | 1.0 | >1.0 |  |  | |
|  |  |  |  |  |  |  |  |  | *S. sonnei* | 0.38 | 3.0 |  |  | |
|  |  |  |  |  |  |  |  |  | *S. typhimurium ATCC 14028* | 0.75 | 3.0 |  |  | |
|  | 300 | | Broth microdilution method (MIC, and MBC) | | Uninformed | | Ciprofloxacin | | *B. bronchiseptica ATCC 4617* | 0.25 | 1.0 |  |  | |
|  |  |  |  |  |  |  |  |  | *B. cereus ATCC 10876* | 0.25 | >2.0 |  |  | |
|  |  |  |  |  |  |  |  |  | *B. subtilis ATCC 6633* | 0.5 | 0.5 |  |  | |
|  |  |  |  |  |  |  |  |  | *E. coli ATCC 25922* | Nd | Nd |  |  | |
|  |  |  |  |  |  |  |  |  | *M. Luteus ATCC 10240* | 0.25 | 2.0 |  |  | |
|  |  |  |  |  |  |  |  |  | *P. aeruginosa ATCC* *27853* | 0.25 | 1.0 |  |  | |
|  |  |  |  |  |  |  |  |  | *S. aureus ATCC 6538* | 0.5 | 1.0 |  |  | |
|  |  |  |  |  |  |  |  |  | *S. aureus ATCC 25923* | Nd | Nd |  |  | |
|  |  |  |  |  |  |  |  |  | *S. aureus ATCC 43300* | Nd | Nd |  |  | |
|  |  |  |  |  |  |  |  |  | *S. epidermidis ATCC 12228* | 0.25 | 1.0 |  |  | |
|  |  |  |  |  |  |  |  |  | *S. sonnei* | 0.25 | 1.0 |  |  | |
|  |  |  |  |  |  |  |  |  | *S. typhimurium ATCC 14028* | Nd | Nd |  |  | |
|  | 299 | | Broth microdilution method (MIC, and MBC) | | Uninformed | | Ciprofloxacin | | *B. bronchiseptica ATCC 4617* | 0.5 | 1.0 |  |  | |
|  |  |  |  |  |  |  |  |  | *B. cereus ATCC 10876* | 1.0 | >1.0 |  |  | |
|  |  |  |  |  |  |  |  |  | *B. subtilis ATCC 6633* | 1.0 | >1.0 |  |  | |
|  |  |  |  |  |  |  |  |  | *E. coli ATCC 25922* | 1.5 | 3.0 |  |  | |
|  |  |  |  |  |  |  |  |  | *M. luteus ATCC 10240* | 1.0 | >1.0 |  |  | |
|  |  |  |  |  |  |  |  |  | *P. aeruginosa ATCC* *27853* | 1.5 | 1.5 |  |  | |
|  |  |  |  |  |  |  |  |  | *S. aureus ATCC 6538* | >1.0 | >1.0 |  |  | |
|  |  |  |  |  |  |  |  |  | *S. aureus ATCC 25923* | >1.0 | >1.0 |  |  | |
|  |  |  |  |  |  |  |  |  | *S. aureus ATCC 43300* | >1.0 | >1.0 |  |  | |
|  |  |  |  |  |  |  |  |  | *S. epidermidis ATCC 12228* | >1.0 | >1.0 |  |  | |
|  |  |  |  |  |  |  |  |  | *S. sonnei* | 0.75 | 3 |  |  | |
|  |  |  |  |  |  |  |  |  | *S. typhimurium ATCC 14028* | 1.5 | >3.0 |  |  | |
|  | 301 | | Broth microdilution method (MIC, and MBC) | | Uninformed | | Ciprofloxacin | | *B. bronchiseptica ATCC 4617* | 0.5 | 1.0 |  |  | |
|  |  |  |  |  |  |  |  |  | *B. cereus ATCC 10876* | 1.0 | >1.0 |  |  | |
|  |  |  |  |  |  |  |  |  | *B. subtilis ATCC 6633* | 1.0 | >1.0 |  |  | |
|  |  |  |  |  |  |  |  |  | *E. coli ATCC 25922* | 1.5 | 3.0 |  |  | |
|  |  |  |  |  |  |  |  |  | *M. Luteus ATCC 10240* | 1.0 | >1.0 |  |  | |
|  |  |  |  |  |  |  |  |  | *P. aeruginosa ATCC* *27853* | 1.5 | 1.5 |  |  | |
|  |  |  |  |  |  |  |  |  | *S. aureus ATCC 6538* | >1.0 | >1.0 |  |  | |
|  |  |  |  |  |  |  |  |  | *S. aureus ATCC 25923* | Nd | Nd |  |  | |
|  |  |  |  |  |  |  |  |  | *S. aureus ATCC 43300* | Nd | Nd |  |  | |
|  |  |  |  |  |  |  |  |  | *S. epidermidis ATCC 12228* | >1.0 | >1.0 |  |  | |
|  |  |  |  |  |  |  |  |  | *S. sonnei* | 0.75 | 3.0 |  |  | |
|  |  |  |  |  |  |  |  |  | *S. typhimurium ATCC 14028* | Nd | Nd |  |  | |
|  | 311 | | Broth microdilution method (MIC, and MBC) | | Uninformed | | Ciprofloxacin | | *B. bronchiseptica ATCC 4617* | 0.25 | 2.0 |  |  | |
|  |  |  |  |  |  |  |  |  | *B. cereus ATCC 10876* | 0.5 | >2.0 |  |  | |
|  |  |  |  |  |  |  |  |  | *B. subtilis ATCC 6633* | 0.25 | 1.0 |  |  | |
|  |  |  |  |  |  |  |  |  | *E. coli ATCC 25922* | 0.75 | 1.5 |  |  | |
|  |  |  |  |  |  |  |  |  | *M. luteus ATCC 10240* | 0.25 | 1.0 |  |  | |
|  |  |  |  |  |  |  |  |  | *P. aeruginosa ATCC* *27853* | 0.5 | 1.0 |  |  | |
|  |  |  |  |  |  |  |  |  | *S. aureus ATCC 6538* | 0.5 | 2.0 |  |  | |
|  |  |  |  |  |  |  |  |  | *S. aureus ATCC 25923* | 1.0 | >1.0 |  |  | |
|  |  |  |  |  |  |  |  |  | *S. aureus ATCC 43300* | 1.0 | >1.0 |  |  | |
|  |  |  |  |  |  |  |  |  | *S. epidermidis ATCC 12228* | 0.25 | >2.0 |  |  | |
|  |  |  |  |  |  |  |  |  | *S. sonnei* | 0.25 | 1.0 |  |  | |
|  |  |  |  |  |  |  |  |  | *S. typhimurium ATCC 14028* | 0.75 | >3.0 |  |  | |
|  | 313 | | Broth microdilution method (MIC, and MBC) | | Uninformed | | Ciprofloxacin | | *B. bronchiseptica ATCC 4617* | 0.25 | 0.5 |  |  | |
|  |  |  |  |  |  |  |  |  | *B. cereus ATCC 10876* | 0.25 | >2.0 |  |  | |
|  |  |  |  |  |  |  |  |  | *B. subtilis ATCC 6633* | 0.25 | 0.5 |  |  | |
|  |  |  |  |  |  |  |  |  | *E. coli ATCC 25922* | Nd | Nd |  |  | |
|  |  |  |  |  |  |  |  |  | *M. luteus ATCC 10240* | 0.25 | 2.0 |  |  | |
|  |  |  |  |  |  |  |  |  | *P. aeruginosa ATCC* *27853* | 0.25 | 0.5 |  |  | |
|  |  |  |  |  |  |  |  |  | *S. aureus ATCC 6538* | 0.5 | 2.0 |  |  | |
|  |  |  |  |  |  |  |  |  | *S. aureus ATCC 25923* | Nd | Nd |  |  | |
|  |  |  |  |  |  |  |  |  | *S. aureus ATCC 43300* | Nd | Nd |  |  | |
|  |  |  |  |  |  |  |  |  | *S. epidermidis ATCC 12228* | 0.25 | 1.0 |  |  | |
|  |  |  |  |  |  |  |  |  | *S. sonnei* | 0.25 | 1.0 |  |  | |
|  |  |  |  |  |  |  |  |  | *S. typhimurium ATCC 14028* | Nd | Nd |  |  | |
|  | 304 | | Broth microdilution method (MIC, and MBC) | | Uninformed | | Ciprofloxacin | | *B. bronchiseptica ATCC 4617* | 0.125 | 0.5 |  |  | |
|  |  |  |  |  |  |  |  |  | *B. cereus ATCC 10876* | 0.25 | >2.0 |  |  | |
|  |  |  |  |  |  |  |  |  | *B. subtilis ATCC 6633* | 0.25 | 0.5 |  |  | |
|  |  |  |  |  |  |  |  |  | *E. coli ATCC 25922* | 1.5 | 3.0 |  |  | |
|  |  |  |  |  |  |  |  |  | *M. luteus ATCC 10240* | 0.25 | 2.0 |  |  | |
|  |  |  |  |  |  |  |  |  | *P. aeruginosa ATCC* *27853* | 0.25 | 0.5 |  |  | |
|  |  |  |  |  |  |  |  |  | *S. aureus ATCC 6538* | 0.5 | 1.0 |  |  | |
|  |  |  |  |  |  |  |  |  | *S. aureus ATCC 25923* | >1.0 | >1.0 |  |  | |
|  |  |  |  |  |  |  |  |  | *S. aureus ATCC 43300* | >1.0 | >1.0 |  |  | |
|  |  |  |  |  |  |  |  |  | *S. epidermidis ATCC 12228* | 0.25 | 1.0 |  |  | |
|  |  |  |  |  |  |  |  |  | *S. sonnei* | 0.25 | 1.0 |  |  | |
|  |  |  |  |  |  |  |  |  | *S. typhimurium ATCC 14028* | 1.5 | >3.0 |  |  | |
|  | 309 | | Broth microdilution method (MIC, and MBC) | | Uninformed | | Ciprofloxacin | | *B. bronchiseptica ATCC 4617* | 0.5 | 1.0 |  |  | |
|  |  |  |  |  |  |  |  |  | *B. cereus ATCC 10876* | 0.5 | >2.0 |  |  | |
|  |  |  |  |  |  |  |  |  | *B. subtilis ATCC 6633* | 0.25 | 1.0 |  |  | |
|  |  |  |  |  |  |  |  |  | *E. coli ATCC 25922* | Nd | Nd |  |  | |
|  |  |  |  |  |  |  |  |  | *M. luteus ATCC 10240* | 0.5 | >2.0 |  |  | |
|  |  |  |  |  |  |  |  |  | *P. aeruginosa ATCC* *27853* | 0.5 | 1.0 |  |  | |
|  |  |  |  |  |  |  |  |  | *S. aureus ATCC 6538* | 0.5 | >2.0 |  |  | |
|  |  |  |  |  |  |  |  |  | *S. aureus ATCC 25923* | Nd | Nd |  |  | |
|  |  |  |  |  |  |  |  |  | *S. aureus ATCC 43300* | Nd | Nd |  |  | |
|  |  |  |  |  |  |  |  |  | *S. epidermidis ATCC 12228* | 0.5 | 2.0 |  |  | |
|  |  |  |  |  |  |  |  |  | *S. sonnei* | 0.5 | >2.0 |  |  | |
|  |  |  |  |  |  |  |  |  | *S. typhimurium ATCC 14028* | Nd | Nd |  |  | |
|  | 305 | | Broth microdilution method (MIC, and MBC) | | Uninformed | | Ciprofloxacin | | *B. bronchiseptica ATCC 4617* | 0.25 | 1.0 |  |  | |
|  |  |  |  |  |  |  |  |  | *B. cereus ATCC 10876* | 0.5 | >2.0 |  |  | |
|  |  |  |  |  |  |  |  |  | *B. subtilis ATCC 6633* | 0.25 | 1.0 |  |  | |
|  |  |  |  |  |  |  |  |  | *E. coli ATCC 25922* | 0.75 | 1.5 |  |  | |
|  |  |  |  |  |  |  |  |  | *M. luteus ATCC 10240* | 0.25 | >2.0 |  |  | |
|  |  |  |  |  |  |  |  |  | *P. aeruginosa ATCC* *27853* | 0.5 | 1.0 |  |  | |
|  |  |  |  |  |  |  |  |  | *S. aureus ATCC 6538* | 0.5 | >2.0 |  |  | |
|  |  |  |  |  |  |  |  |  | *S. aureus ATCC 25923* | >1.0 | >1.0 |  |  | |
|  |  |  |  |  |  |  |  |  | *S. aureus ATCC 43300* | 1.0 | >1.0 |  |  | |
|  |  |  |  |  |  |  |  |  | *S. epidermidis ATCC 12228* | 0.5 | 2.0 |  |  | |
|  |  |  |  |  |  |  |  |  | *S. sonnei* | 0.5 | >2.0 |  |  | |
|  |  |  |  |  |  |  |  |  | *S. typhimurium ATCC 14028* | 1.5 | >3.0 |  |  | |
|  |  |  |  |  |  |  |  |  |  |  |  |  |  |  |
|  | 310 | | Broth microdilution method (MIC, and MBC) | | Uninformed | | Ciprofloxacin | | *B. bronchiseptica ATCC 4617* | 0.5 | 1.0 |  |  | |
|  |  |  |  |  |  |  |  |  | *B. cereus ATCC 10876* | 0.5 | >2.0 |  |  | |
|  |  |  |  |  |  |  |  |  | *B. subtilis ATCC 6633* | 0.5 | 2.0 |  |  | |
|  |  |  |  |  |  |  |  |  | *E. coli ATCC 25922* | 1.5 | 1.5 |  |  | |
|  |  |  |  |  |  |  |  |  | *M. luteus ATCC 10240* | 0.5 | 2.0 |  |  | |
|  |  |  |  |  |  |  |  |  | *P. aeruginosa ATCC* *27853* | 0.5 | 1 |  |  | |
|  |  |  |  |  |  |  |  |  | *S. aureus ATCC 6538* | 1.0 | 2.0 |  |  | |
|  |  |  |  |  |  |  |  |  | *S. aureus ATCC 25923* | 1.0 | >1.0 |  |  | |
|  |  |  |  |  |  |  |  |  | *S. aureus ATCC 43300* | >1.0 | >1.0 |  |  | |
|  |  |  |  |  |  |  |  |  | *S. epidermidis ATCC 12228* | 0.5 | 2.0 |  |  | |
|  |  |  |  |  |  |  |  |  | *S. sonnei* | 0.5 | 2.0 |  |  | |
|  |  |  |  |  |  |  |  |  | *S. typhimurium ATCC 14028* | 1.5 | >3.0 |  |  | |
| *Gomphrena globose L* | EaE of whole plant | | Broth microdilution  method | | Uninformed | | Ceftriaxone sodium | | *P. aeruginosa* | 1.02 |  | EaE and four of the isolated compounds showed activity against *P. aeuruginsa*, with Kaempferol obtaining the lowest MIC, which was lower than that of the positive control.  In accordance with time-kill curves assays, all four compounds inhibited *P. aeruginosa* at the 2XMIC concentration at 12 h.  The SEM analysis allowed to observe that P. aeruginosa treated with 2xMIC was severely broken, causing leakage of its internal contents. | [144] | |
|  | 94 | | Broth microdilution  method | | Uninformed | | Ceftriaxone sodium | | *P. aeruginosa* | ND |  |  |  | |
|  | 95 | | Broth microdilution  method | | Uninformed | | Ceftriaxone sodium | | *P. aeruginosa* | ND |  |  |  | |
|  | 92 | | Broth microdilution  method | | Uninformed | | Ceftriaxone sodium | | *P. aeruginosa* | ND |  |  |  | |
|  | 20 | | Broth microdilution  Method, Time-Kill Curves and SEM | | Uninformed | | Ceftriaxone sodium | | *P. aeruginosa* | 0.008 |  |  |  | |
|  | 109 | | Broth microdilution  Method, Time-Kill Curves and SEM | | Uninformed | | Ceftriaxone sodium | | *P. aeruginosa* | 0.256 |  |  |  | |
|  | 108 | | Broth microdilution  Method, Time-Kill Curves and SEM | | Uninformed | | Ceftriaxone sodium | | *P. aeruginosa* | 0.128 |  |  |  | |
|  | 107 | | Broth microdilution  Method, Time-Kill Curves and SEM | | Uninformed | | Ceftriaxone sodium | | *P. aeruginosa* | 0.256 |  |  |  | |
|  | 51 | | Broth microdilution  method | | Uninformed | | Ceftriaxone sodium | | *P. aeruginosa* | ND |  |  |  | |
|  | 71 | | Broth microdilution  method | | Uninformed | | Ceftriaxone sodium | | *P. aeruginosa* | ND |  |  |  | |
|  | 50 | | Broth microdilution  method | | Uninformed | | Ceftriaxone sodium | | *P. aeruginosa* | ND |  |  |  | |
|  | 93 | | Broth microdilution  method | | Uninformed | | Ceftriaxone sodium | | *P. aeruginosa* | ND |  |  |  | |
|  | 100 | | Broth microdilution  method | | Uninformed | | Ceftriaxone sodium | | *P. aeruginosa* | ND |  |  |  | |
|  | 101 | | Broth microdilution  method | | Uninformed | | Ceftriaxone sodium | | *P. aeruginosa* | ND |  |  |  | |
|  |  | |  | |  | |  | |  | MIC (μg/mL) | ZI (mm) |  |  | |
| *Gomphrena martiana* | EE of whole plant | | Agar dilution technique | | 4000, 2000, 1000, 500, 250 and 125 µg/mL | |  | | *C. butyricum* | 4000 |  | None of the extracts showed activity against: *E. aerogenes*, *S. newport*, *S. oranienburg*, *E. coli* B, *E. coli* K 12, *K. pneumoniae*, *S. marcescens*, *P. aeruginosa* and *P. vulgaris,* and additionally EE did not show activity against *B. subtilis.*  PEE showed strong activity against: *C. tetanii*, *C. sporogenes* and *M. phlei*. | [75] | |
|  |  |  |  |  |  |  |  | | *C. sporogenes* | 4000 |  |  |  |  |
|  |  |  |  |  |  |  |  | | *C. tetanii* | 1000 |  |  |  |  |
|  |  |  |  |  |  |  |  | | *M. luteus* | 2000 |  |  |  |  |
|  |  |  |  |  |  |  |  | | *M. phlei* | 250 |  |  |  |  |
|  |  |  |  |  |  |  |  | | *Staph. aureus ATTC* *12600* | 2000 |  |  |  |  |
|  |  |  |  |  |  |  |  | | *S. faecalis ATCC 19433* | 2000 |  |  |  |  |
|  | PEE of whole plant | | Agar dilution technique | | 4000, 2000, 1000, 500, 250 and 125 µg/mL | |  | | *B. subtilis* | 1000 |  |  |  |  |
|  |  |  |  |  |  |  |  | | *C. butyricum* | 1000 |  |  |  |  |
|  |  |  |  |  |  |  |  | | *C. sporogenes* | 250 |  |  |  |  |
|  |  |  |  |  |  |  |  | | *C. tetanii* | 250 |  |  |  |  |
|  |  |  |  |  |  |  |  | | *M. luteus* | 500 |  |  |  |  |
|  |  |  |  |  |  |  |  | | *M. phlei* | <50 |  |  |  |  |
|  |  |  |  |  |  |  |  | | *Staph. aureus ATTC* *12600* | 500 |  |  |  |  |
|  |  |  |  |  |  |  |  | | *S. faecalis ATCC 19433* | 1000 |  |  |  |  |
| *Hebanthe eriantha* | ME of roots | | Agar disc diffusion method | | 10 to 100 mg/mL | | Gentamicin | | *E. coli* ATCC 10536 | >1000 | 9.5 ± 0.5 | The MEs showed interesting antibacterial activity, with greater inhibitory activity against *S. aureus* and *P. vulgaris*. In particular, the effect against *S. aureus* was the same compared to the Gentamicin control. | [230] | |
|  |  |  |  |  |  |  |  | | *K. pneumoniae* ATCC 10031 | 1000 | 12.5 ± 2.5 |  |  |  |
|  |  |  |  |  |  |  |  | | *P. vulgaris* PTCC 1182 | 500 | 16.0 ± 0.5 |  |  |  |
|  |  |  |  |  |  |  |  | | *S. paratyphi* ATCC 5702 | >1000 | 10.0 ± 1.2 |  |  |  |
|  |  | |  | |  | |  | | *S. aureus* ATCC 29737 | 500 | 17.5 ± 1.5 |  |  |  |
| *Iresine herbstii* | AgNPs of AqE of leaves | | Agar well-diffusion method and broth micro-dilution method | | 50, 100, 150, 200 and 250 µg/mL | | kanamycin,  norfloxacin and ciprofloxacin | | *E. faecalis* | 12.5 | 15.0 ± 0.0 | Activity against *S. aureus* and *E. faecalis* was higher than positive controls and the MIC against *E. coli* was lower than that of kanamycin. | [231] | |
|  |  |  |  |  |  |  |  |  | *E. coli* | 6.25 | 15.7 ± 0.6 |  |  |  |
|  |  |  |  |  |  |  |  |  | *K. pneumoniae* | 50 | 13.7 ± 0.6 |  |  |  |
|  |  |  |  |  |  |  |  |  | *P. aeruginosa* | 12.5 | 12.3 ± 0.6 |  |  |  |
|  |  |  |  |  |  |  |  |  | *S. aureus* | 12.5 | 14.8 ± 0.3 |  |  |  |
| **Antifungal activity** | | | | | | | | | | | | | | |
|  | |  | |  | |  | |  |  | MIC (µg/mL) | ZI (mm) |  |  | |
| *Alternanthera brasiliana* | | HaE of aerial parts | | Broth microdilution method | | 1000 to 7.8 µg/mL | | Amphotericin B | *P. brasiliensis ATCC MYA826* | >1000 |  | All strains of *P. brasiliensis* were resistant to HaE. | [232] | |
|  |  |  |  |  |  |  |  |  | *P. brasiliensis ATCC 32069* | >1000 |  |  |  |  |
|  |  |  |  |  |  |  |  |  | *P. brasiliensis Pb18* | >1000 |  |  |  |  |
|  | |  | |  | |  | |  |  | MIC (mg/mL) | ZI (mm) |  |  | |
| *Alternanthera brasiliana* | | HeE of whole plant | | Agar-well diffusion method (well technique with double layers) and method of microdilution | | Uninformed | | Ketoconazole | *C. dubliniensis ATCC 778157* | 25.0 | - | HeE (2.5 mg / kg) and EE (25 mg / kg) were not active against C. dubliniensis without photosensitizing, but when the strains were photosencitized, the EE extract completely inhibited growth, and HeE reduced viability by 99.98%. | [23] | |
|  |  | EE of whole plant | |  |  | Uninformed | | Ketoconazole | *C. dubliniensis ATCC 778157* | 50.0 | - |  |  |  |
|  |  |  | |  | |  | |  |  | %V of MI^-^ | %V of MI^+^ |  |  |  |
|  |  | HeE of whole plant | | Photosensitization assays | | 2.5 mg/mL | | Methylene blue and ketoconazole | *C. dubliniensis ATCC 778157* | 100 | 0.02 |  |  |  |
|  |  | EE of whole plant | | Photosensitization assays | | 25 mg/mL | | Methylene blue and ketoconazole | *C. dubliniensis ATCC 778157* | 100 | 0 |  |  |  |
|  | |  | |  | |  | |  |  | MIC (μg/mL) | ZI (mm) |  |  | |
| *Alternanthera brasiliana* | | AqE of leaves | | Broth microdilution method | | 3.9 – 2000 μg/mL | | Ketoconazole | *C. albicans 1007-UFPEDA* | 31.2 |  |  | [27] | |
|  | |  | |  | |  | |  |  | MIC (mg/mL) | ZI (mm) |  |  | |
| *Alternanthera caracasana HBK* | | AcE, ClE, EaE, HeE, and ME of aerial part | | Disk diffusion test | | Uninformed | | CAM | *C. albicans* |  | NE | None of the extracts showed activity against *C. albicans* | [33] | |
| *Alternanthera maritima* | | EE of aerial parts | | Agar-well diffusion method (well technique  with double layers) and MIC | | 100 mg/mL | | Ketoconazole | *C. dubliniensis ATCC 777* | 50 | - | The extracts at 25 mg/mL were not active against *C. dubliniensis* without photosencilization, but when the strains were photosenbilized, the extracts generated a ↓ in viability of more than 98%. | [138] | |
|  |  |  |  |  |  |  | |  | *C. dubliniensis ATCC 778157* | 50 | - |  |  |  |
|  |  | HeE of aerial parts | |  |  | 100 mg/mL | | Ketoconazole | *C. dubliniensis ATCC 777* | 50 | - |  |  |  |
|  |  |  |  |  |  |  | |  | *C. dubliniensis ATCC 778157* | 50 | - |  |  |  |
|  |  |  | |  | |  | |  |  | %V of MI^-^ | %V of MI^+^ |  |  |  |
|  |  | EE of aerial parts | | Photosensitization assays | | 25 mg/mL | | Methylene blue and ketoconazole | *C. dubliniensis ATCC 777* | 100 % | 1,18 % |  |  |  |
|  |  |  |  |  |  |  | |  | *C. dubliniensis ATCC 778157* | 100 % | 1,84 % |  |  |  |
|  |  | HeE of aerial parts | | Photosensitization assays | | 25 mg/mL | | Methylene blue and ketoconazole | *C. dubliniensis ATCC 777* | 100 % | 1.09 % |  |  |  |
|  |  |  |  |  | |  | |  | *C. dubliniensis ATCC 778157* | 100 % | 0.19 % |  |  |  |
|  |  |  | |  | |  | |  |  | MIC (μg/mL) | ZI (mm) |  |  | |
| *Alternanthera pungens* | | AqE of whole plant | | Agar tube dilution method | | 6.25, 12.5, 25 and 50 mg/mL | | Fluconazole | *A. flavus* | - | No activity | The extracts showed weak activity against *A. niger* and only at high concentrations was observed activity against *Mucor sp.* | [16] | |
|  |  |  |  |  |  |  |  |  | *A. niger* | - | NDNS |  |  |  |
|  |  |  |  |  |  |  |  |  | *Mucor sp* | - | NDNS |  |  |  |
|  |  | ME of whole plant | | Agar tube dilution method | | 6.25, 12.5, 25 and 50 mg/mL | | Fluconazole | *A. flavus* | - | No activity |  |  |  |
|  |  |  |  |  |  |  |  |  | *A. niger* | - | NDNS |  |  |  |
|  |  |  |  |  |  |  |  |  | *Mucor sp* | - | NDNS |  |  |  |
|  |  | nHE of whole plant | | Agar tube dilution method | | 6.25, 12.5, 25 and 50 mg/mL | | Fluconazole | *A. flavus* | - | No activity |  |  |  |
|  |  |  |  |  |  |  |  |  | *A. niger* | - | NDNS |  |  |  |
|  |  |  |  |  |  |  |  |  | *Mucor sp* | - | NDNS |  |  |  |
| *Alternanthera repens* | | ME | | Disk diffusion test | | 2,5 mg | | Nystatin | *C. albicans* |  | NE | ME has no activity. | [216] | |
| *Alternanthera tenella* Colla | | Different extracts of the whole plant | | Agar-well  diffusion method (well technique in double layer) | | 5.0 mg/mL | | Ketoconazole | Yeasts (seven strains) and dermatophytes (four strains) | NDNS | NDNS | The organic extracts from a callus culture and whole plant were considerably active against the microorganisms evaluated, but the AqE did not show appreciable activity.  None of the extracts evaluated showed activity against *C. albicans* (strains ATCC 1023, 64548 and cas), and *C. parapsilosis* ATCC 22019. | [142] | |
|  | | Different extracts of callus | |  |  | 5.0 mg/mL | | Ketoconazole | Yeasts (seven strains) and dermatophytes (four strains) | NDNS | NDNS |  |  | |
|  | | 411 | | Agar-well  diffusion method (well technique in double layer) | | 50 and 500 μg/mL | | Ketoconazole | *T. rubrum* Tr 5 | 500 | T |  |  | |
|  | | 411 and 386 | | Agar-well  diffusion method (well technique in double layer) | | 50 and 500 μg/mL | | Ketoconazole | *C. albicans* ATCC 1023 | >500 | 7 |  |  | |
|  | |  |  |  |  |  |  |  | *C. albicans* cas | >500 | 6 |  |  | |
|  | |  |  |  |  |  |  |  | *T. rubrum* Tr 5 | 500 | T |  |  | |
|  | | 396 and 411 | | Agar-well  diffusion method (well technique in double layer) | | 50 and 500 μg/mL | | Ketoconazole | *C. albicans* ATCC 1023 | >500 | 9 |  |  | |
|  | |  |  |  |  |  |  |  | *C. albicans* cas | >500 | 7 |  |  | |
|  | |  |  |  |  |  |  |  | *T. rubrum* Tr 5 | >500 | T |  |  | |
|  | | 415 and 416 | | Agar-well  diffusion method (well technique in double layer) | | 50 and 500 μg/mL | | Ketoconazole | *C. albicans* cas | >500 | 6 |  |  | |
|  | |  |  |  |  |  |  |  | *C. krusei* ATCC 6258 | 500 | 7 |  |  | |
|  | |  |  |  |  |  |  |  | *T. rubrum* Tr 19 | 500 | 7 |  |  | |
|  | |  |  |  |  |  |  |  | *T, mentagrophytes* Tm 9 | 500 | 7 |  |  | |
|  | | 416 | | Agar-well  diffusion method (well technique in double layer) | | 50 and 500 μg/mL | | Ketoconazole | *C. albicans* cas | >500 | 6 |  |  | |
|  | |  |  |  |  |  |  |  | *C. krusei* ATCC 6258 | 500 | 7 |  |  | |
|  | |  |  |  |  |  |  |  | *T. rubrum* Tr 19 | 500 | T |  |  | |
|  | |  |  |  |  |  | |  | *T, mentagrophytes* Tm 9 | >500 | T |  |  | |
|  | | 394 and 414 | | Agar-well  diffusion method (well technique in double layer) | | 50 and 500 μg/mL | | Ketoconazole | *C. albicans* cas | >500 | 6 |  |  | |
|  | |  |  |  |  |  |  |  | *C. glabrata* ATCC 90030 | >500 | 8 |  |  | |
|  | |  |  |  |  |  |  |  | *C. krusei* ATCC 6258 | 500 | 7 |  |  | |
|  | |  |  |  |  |  |  |  | *T. rubrum* Tr 5 | 100 | 10 |  |  | |
|  | |  |  |  |  |  |  |  | *T. rubrum* Tr 19 | 500 | 7 |  |  | |
|  | |  |  |  |  |  |  |  | *T, mentagrophytes* Tm 9 | >500 | 7 |  |  | |
|  | | 20 | | Agar-well  diffusion method (well technique in double layer) | | 50 and 500 μg/mL | | Ketoconazole | *C. glabrata* ATCC 90030 | 500 | 8 |  |  | |
|  | |  |  |  |  |  |  |  | *C. krusei* ATCC 6258 | 500 | 7 |  |  | |
|  | |  |  |  |  |  |  |  | *T. rubrum* Tr 5 | 500 | 8 |  |  | |
|  | |  |  |  |  |  |  |  | *T. rubrum* Tr 19 | 500 | 8 |  |  | |
|  | |  |  |  |  |  |  |  | *T, mentagrophytes* Tm 9 | 500 | 7 |  |  | |
|  | | 25 | | Agar-well  diffusion method (well technique in double layer) | | 50 and 500 μg/mL | | Ketoconazole | *C. glabrata* ATCC 90030 | 500 | 9 |  |  | |
|  | |  |  |  |  |  |  |  | *C. krusei* ATCC 6258 | 500 | 7 |  |  | |
|  | |  |  |  |  |  |  |  | *T. rubrum* Tr 5 | 500 | 20 |  |  | |
|  | |  |  |  |  |  |  |  | *T. rubrum* Tr 19 | 500 | 8 |  |  | |
|  | |  |  |  |  |  |  |  | *T, mentagrophytes* Tm 9 | 500 | 7 |  |  | |
|  | |  |  |  |  |  |  |  | *T, mentagrophytes* Tm 17 | 500 | 8 |  |  | |
|  | | 24 | | Agar-well  diffusion method (well technique in double layer) | | 50 and 500 μg/mL | | Ketoconazole | *C. glabrata* ATCC 90030 | 500 | 6 |  |  | |
|  | |  |  |  |  |  | |  | *C. krusei* ATCC 6258 | 500 | 6 |  |  | |
|  | |  |  |  |  |  | |  | *C. parapsilosis* ATCC 22019 | >500 | 6 |  |  | |
|  | |  |  |  |  |  | |  | *T. rubrum* Tr 5 | 500 | 7 |  |  | |
|  | |  |  |  |  |  | |  | *T, mentagrophytes* Tm 9 | 500 | 7 |  |  | |
|  | | 88 | | Agar-well  diffusion method (well technique in double layer) | | 50 and 500 μg/mL | | Ketoconazole | *C. glabrata* ATCC 90030 | 100 | 7 |  |  | |
|  | |  | |  |  |  |  |  | *C. parapsilosis* ATCC 22019 | 100 | 7 |  |  | |
|  | |  | |  |  |  |  |  | *T. rubrum* Tr 5 | 500 | 7 |  |  | |
|  | |  | |  |  |  |  |  | *T, mentagrophytes* Tm 9 | 100 | 7 |  |  | |
|  | |  | |  |  |  |  |  | *T, mentagrophytes* Tm 17 | 500 | 6 |  |  | |
|  | | 43 | | Agar-well  diffusion method (well technique in double layer) | | 50 and 500 μg/mL | | Ketoconazole | *C. parapsilosis* ATCC 22019 | >500 | 6 |  |  | |
|  | | 72 | | Agar-well  diffusion method (well technique in double layer) | | 50 and 500 μg/mL | | Ketoconazole | *C. parapsilosis* ATCC 22019 | >500 | 6 |  |  | |
|  | | 34 | | Agar-well  diffusion method (well technique in double layer) | | 50 and 500 μg/mL | | Ketoconazole | *C. parapsilosis* ATCC 22019 | >500 | 6 |  |  | |
| *Gomphrena agrestis* | | EE or 327 or 33, or 106 | | Modified agar-well diffusion method (well technique in double layer) | | 1 mg/ml | | Ketoconazole | *C. albicans* ATTC 1023 | No activity | |  | [2] | |
|  |  |  |  |  |  |  |  |  | *C. albicans* cas | No activity | |  |  |  |
|  |  |  |  |  |  |  |  |  | *C. albicans* ct | No activity | |  |  |  |
| *Gomphrena boliviana* | | EE of whole plant | | Agar dilution technique | | 4000, 2000, 1000, 500, 250 and 125 µg/mL | |  | *A. niger* | 2000 |  | The two extracts showed weak activity against *A. niger*.  EE showed moderate activity against *C. albicans* and *S. cerevisiae.* | [75] | |
|  |  |  |  |  |  |  |  |  | *C. albicans* | 250 |  |  |  |  |
|  |  |  |  |  |  |  |  |  | *S. cerevisiae* | 250 |  |  |  |  |
|  |  | PEE of whole plant | | Agar dilution technique | | 4000, 2000, 1000, 500, 250 and 125 µg/mL | |  | *A. niger* | 2000 |  |  |  |  |
|  |  |  |  |  |  |  |  |  | *C. albicans* | 2000 |  |  |  |  |
|  |  |  |  |  |  |  |  |  | *S. cerevisiae* | 1000 |  |  |  |  |
| *Gomphrena boliviana and Gomphrena martiana* | | 10 | | Agar dilution technique | | 125 – 4000 µg/mL | | - | *A. niger* | *500* |  | The compounds inhibited the formation of conidia of *A. niger* but without affecting growth. | [75] | |
|  |  |  |  |  |  |  |  |  | *C. albicans* | *500* |  |  |  |  |
|  |  |  |  |  |  |  |  |  | *S. cerevisiae* | *1000* |  |  |  |  |
|  |  | 6 | | Agar dilution technique | | 125 – 4000 µg/mL | | - | *A. niger* | *1000* |  |  |  |  |
|  |  |  |  |  |  |  |  |  | *C. albicans* | *500* |  |  |  |  |
|  |  |  |  |  |  |  |  |  | *S. cerevisiae* | *250* |  |  |  |  |
|  |  | 7 | | Agar dilution technique | | 125 – 4000 µg/mL | | - | *A. niger* | *1000* |  |  |  |  |
|  |  |  |  |  |  |  |  |  | *C. albicans* | *500* |  |  |  |  |
|  |  |  |  |  |  |  |  |  | *S. cerevisiae* | *250* |  |  |  |  |
|  |  | 17 | | Agar dilution technique | | 125 – 4000 µg/mL | | - | *A. niger* | *500* |  |  |  |  |
|  |  |  |  |  |  |  |  |  | *C. albicans* | *500* |  |  |  |  |
|  |  |  |  |  |  |  |  |  | *S. cerevisiae* | *1000* |  |  |  |  |
|  |  | 9 | | Agar dilution technique | | 125 – 4000 µg/mL | | - | *A. niger* | *1000* |  |  |  |  |
|  |  |  |  |  |  |  |  |  | *C. albicans* | *1000* |  |  |  |  |
|  |  |  |  |  |  |  |  |  | *S. cerevisiae* | *1000* |  |  |  |  |
| *Gomphrena celosioides* | | ME of Whole planta | | Agar cup diffusion method | | 12.5 mg/ml | | Tioconazole | *A. niger* |  | 17 ±0.5 | The activity of the ME was comparable to that of the positive control | [82] | |
|  |  |  |  |  |  |  |  |  | *C. albicans* NCTC 7534 |  | 20 ± 0.3 |  |  |  |
|  |  |  |  |  |  |  |  |  | Tricophyton species |  | 14 ± 0.1 |  |  |  |
|  | |  | |  | |  | |  |  | MIC (mg/mL) | MFC (mg/mL) |  |  | |
| *Gomphrena globosa L* | | AcE of flowers | | Broth microdilution method | | Uninformed | | Fluconazole | *C. albicans ATCC 2091* | *1.5* | 1.5 | The AcE showed mild activity towards yeast.  All fractions showed activity against yeast with MIC of 0.75-1.5.  The fractions showed greater activity than AcE, and the isolated compounds showed greater activity than the fractions. | [173] | |
|  |  |  |  |  |  |  |  |  | *C. albicans*  *ATCC 10231* | *1.5* | 1.5 |  |  |  |
|  |  |  |  |  |  |  |  |  | *C. parapsilosis ATCC 22019* | *1.5* | 3.0 |  |  |  |
|  |  | F1 | | Broth microdilution method | | Uninformed | | Fluconazole | *C. albicans ATCC 2091* | *1.5* | 3.0 |  |  |  |
|  |  |  |  |  |  |  |  |  | *C. albicans*  *ATCC 10231* | *1.5* | 3.0 |  |  |  |
|  |  |  |  |  |  |  |  |  | *C. parapsilosis ATCC 22019* | *1.5* | 3.0 |  |  |  |
|  |  | F2 | | Broth microdilution method | | Uninformed | | Fluconazole | *C. albicans ATCC 2091* | *1.5* | 1.5 |  |  |  |
|  |  |  |  |  |  |  |  |  | *C. albicans*  *ATCC 10231* | *1.5* | 3.0 |  |  |  |
|  |  |  |  |  |  |  |  |  | *C. parapsilosis ATCC 22019* | *0.75* | 3.0 |  |  |  |
|  |  | F3 | | Broth microdilution method | | Uninformed | | Fluconazole | *C. albicans ATCC 2091* | *1.5* | 3.0 |  |  |  |
|  |  |  |  |  |  |  |  |  | *C. albicans*  *ATCC 10231* | *1.5* | 3.0 |  |  |  |
|  |  |  |  |  |  |  |  |  | *C. parapsilosis ATCC 22019* | *0.75* | 3.0 |  |  |  |
|  |  | F4 | | Broth microdilution method | | Uninformed | | Fluconazole | *C. albicans ATCC 2091* | *1.5* | 1.5 |  |  |  |
|  |  |  |  |  |  |  |  |  | *C. albicans*  *ATCC 10231* | *0.75* | 1.5 |  |  |  |
|  |  |  |  |  |  |  |  |  | *C. parapsilosis ATCC 22019* | *1.5* | 1.5 |  |  |  |
|  |  | F5 | | Broth microdilution method | | Uninformed | | Fluconazole | *C. albicans ATCC 2091* | *1.5* | 3.0 |  |  |  |
|  |  |  |  |  |  |  |  |  | *C. albicans*  *ATCC 10231* | *1.5* | 3.0 |  |  |  |
|  |  |  |  |  |  |  |  |  | *C. parapsilosis ATCC 22019* | *1.5* | 3.0 |  |  |  |
|  |  | F6 | | Broth microdilution method | | Uninformed | | Fluconazole | *C. albicans ATCC 2091* | *0.75* | 0.75 |  |  |  |
|  |  |  |  |  |  |  |  |  | *C. albicans*  *ATCC 10231* | *0.75* | 1.5 |  |  |  |
|  |  |  |  |  |  |  |  |  | *C. parapsilosis ATCC 22019* | *0.75* | 1.5 |  |  |  |
|  |  | F7 | | Broth microdilution method | | Uninformed | | Fluconazole | *C. albicans ATCC 2091* | *0.75* | 0.75 |  |  |  |
|  |  |  |  |  |  |  |  |  | *C. albicans*  *ATCC 10231* | *0.75* | 1.5 |  |  |  |
|  |  |  |  |  |  |  |  |  | *C. parapsilosis ATCC 22019* | *0.75* | 1.5 |  |  |  |
|  | | 303/308 | | Broth microdilution method | | Uninformed | | Fluconazole | *C. albican ATCC 10231* | *0.75* | 1.5 |  |  | |
|  | |  |  |  |  |  |  |  | *C. glabrata ATCC 90030* | 1.5 | 1.5 |  |  | |
|  | |  |  |  |  |  |  |  | *C. krusei ATCC 14243* | *0.75* | 1.5 |  |  | |
|  | |  |  |  |  |  |  |  | *C. parapsilosis ATCC 22019* | *0.75* | 1.5 |  |  | |
|  | | 298 | | Broth microdilution method | | Uninformed | | Fluconazole | *C. albican ATCC 10231* | *0.38* | *0.75* |  |  | |
|  | |  |  |  |  |  |  |  | *C. glabrata ATCC 90030* | *0.75* | *0.75* |  |  | |
|  | |  |  |  |  |  |  |  | *C. krusei ATCC 14243* | *0.19* | *0.75* |  |  | |
|  | |  |  |  |  |  |  |  | *C. parapsilosis ATCC 22019* | *0.38* | *0.75* |  |  | |
|  | | 300 | | Broth microdilution method | | Uninformed | | Fluconazole | *C. albican ATCC 10231* | *0.125* | 0.25 |  |  | |
|  | |  |  |  |  |  |  |  | *C. glabrata ATCC 90030* | *Nd* | Nd |  |  | |
|  | |  |  |  |  |  |  |  | *C. krusei ATCC 14243* | *Nd* | Nd |  |  | |
|  | |  |  |  |  |  |  |  | *C. parapsilosis ATCC 22019* | *0.125* | 0.25 |  |  | |
|  | | 299 | | Broth microdilution method | | Uninformed | | Fluconazole | *C. albican ATCC 10231* | *0.75* | 1.5 |  |  | |
|  | |  |  |  |  |  |  |  | *C. glabrata ATCC 90030* | *0.75* | 1.5 |  |  | |
|  | |  |  |  |  |  |  |  | *C. krusei ATCC 14243* | *0.75* | 1.5 |  |  | |
|  | |  |  |  |  |  |  |  | *C. parapsilosis ATCC 22019* | *0.75* | 1.5 |  |  | |
|  | | 301 | | Broth microdilution method | | Uninformed | | Fluconazole | *C. albican ATCC 10231* | *0.75* | 1.5 |  |  | |
|  | |  |  |  |  |  |  |  | *C. glabrata ATCC 90030* | *Nd* | Nd |  |  | |
|  | |  |  |  |  |  |  |  | *C. krusei ATCC 14243* | *Nd* | Nd |  |  | |
|  | |  |  |  |  |  |  |  | *C. parapsilosis ATCC 22019* | *0.75* | 1.5 |  |  | |
|  | | 311 | | Broth microdilution method | | Uninformed | | Fluconazole | *C. albican ATCC 10231* | *0.125* | 0.25 |  |  | |
|  | |  |  |  |  |  |  |  | *C. glabrata ATCC 90030* | *0.75* | 0.75 |  |  | |
|  | |  |  |  |  |  |  |  | *C. krusei ATCC 14243* | *0.38* | 0.75 |  |  | |
|  | |  |  |  |  |  |  |  | *C. parapsilosis ATCC 22019* | *0.125* | 0.5 |  |  | |
|  | | 313 | | Broth microdilution method | | Uninformed | | Fluconazole | *C. albican ATCC 10231* | *0.25* | 0.5 |  |  | |
|  | |  |  |  |  |  |  |  | *C. glabrata ATCC 90030* | *Nd* | Nd |  |  | |
|  | |  |  |  |  |  |  |  | *C. krusei ATCC 14243* | *Nd* | Nd |  |  | |
|  | |  |  |  |  |  |  |  | *C. parapsilosis ATCC 22019* | *0.125* | 0.5 |  |  | |
|  | | 304 | | Broth microdilution method | | Uninformed | | Fluconazole | *C. albican ATCC 10231* | *0.125* | 0.25 |  |  | |
|  | |  |  |  |  |  |  |  | *C. glabrata ATCC 90030* | *1.5* | 3.0 |  |  | |
|  | |  |  |  |  |  |  |  | *C. krusei ATCC 14243* | *0.75* | 1.5 |  |  | |
|  | |  |  |  |  |  |  |  | *C. parapsilosis ATCC 22019* | *0.125* | 0.25 |  |  | |
|  | | 309 | | Broth microdilution method | | Uninformed | | Fluconazole | *C. albican ATCC 10231* | 0.25 | 0.5 |  |  | |
|  | |  |  |  |  |  |  |  | *C. glabrata ATCC 90030* | *Nd* | *Nd* |  |  | |
|  | |  |  |  |  |  |  |  | *C. krusei ATCC 14243* | *Nd* | *Nd* |  |  | |
|  | |  |  |  |  |  |  |  | *C. parapsilosis ATCC 22019* | 0.25 | 0.5 |  |  | |
|  | | 305 | | Broth microdilution method | | Uninformed | | Fluconazole | *C. albican ATCC 10231* | *0.38* | *0.75* |  |  | |
|  | |  |  |  |  |  |  |  | *C. glabrata ATCC 90030* | *0.75* | 3.0 |  |  | |
|  | |  |  |  |  |  |  |  | *C. krusei ATCC 14243* | *0.38* | *0.75* |  |  | |
|  | |  |  |  |  |  |  |  | *C. parapsilosis ATCC 22019* | *0.38* | 1.5 |  |  | |
|  | | 310 | | Broth microdilution method | | Uninformed | | Fluconazole | *C. albican ATCC 10231* | *0.38* | *0.75* |  |  | |
|  | |  |  |  |  |  |  |  | *C. glabrata ATCC 90030* | *0.75* | 1.5 |  |  | |
|  | |  |  |  |  |  |  |  | *C. krusei ATCC 14243* | *0.38* | 1.5 |  |  | |
|  | |  |  |  |  |  |  |  | *C. parapsilosis ATCC 22019* | *0.38* | 1.5 |  |  | |
|  | |  | |  | |  | |  |  | MIC (μg/mL) | ZI (mm) |  |  | |
| *Gomphrena martiana* | | EE of whole plant | | Agar dilution technique | | 4000, 2000, 1000, 500, 250 and 125 µg/mL | |  | *A. niger* | *2000* |  | The two extracts showed weak activity against *A. niger*.  EE showed moderate activity against *C. albicans* and *S. cerevisiae*. | [75] | |
|  |  |  |  |  |  |  |  |  | *C. albicans* | *250* |  |  |  |  |
|  |  |  |  |  |  |  |  |  | *S. cerevisiae* | *250* |  |  |  |  |
|  |  | PEE of whole plant | | Agar dilution technique | | 4000, 2000, 1000, 500, 250 and 125 µg/mL | |  | *A. niger* | *2000* |  |  |  |  |
|  |  |  |  |  |  |  |  |  | *C. albicans* | *2000* |  |  |  |  |
|  |  |  |  |  |  |  |  |  | *S. cerevisiae* | *1000* |  |  |  |  |
|  | |  | |  | |  | |  |  | *% I* | |  |  | |
| *Pfaffia paniculata* | | Glycolic extract of roots | | Biofilm formation and treatment | | 25, 50 and 100 mg/mL | |  | *C. albicans ATCC 18804 and S. mutans ATCC 35688* | *100 mg/mL for C. albicans: 100%* | | The extract has action  over mixed-species biofilms of *C. albicans* and *S. mutans*, *S. aureus* or *P. aeruginosa* after 24 h of treatment in different concentrations. | [233] | |
|  | |  | |  | |  | |  | *C. albicans ATCC 18804 and S. aureus ATCC 6538* | *100 mg/mL for C. albicans:* *48.4% and 100 mg/mL for S. aureus: 55.5%* | |  |  | |
|  | |  | |  | |  | |  | *C. albicans ATCC 18804 and E. faecalis ATCC 4083* | *100 mg/mL for C. albicans: 100%* | |  |  | |
|  | |  | |  | |  | |  | *C. albicans ATCC 18804 and P. aeruginosa ATCC 15442* | *50 and 100 mg/mL for P. aeruginosa: 100%* | |  |  | |
| **Antiparasitic activity** | | | | | | | | | | | | | |  |
|  | |  | |  | |  | |  |  | IC_50_ (mM) | | Analysis |  | |
| *Alternanthera littoralis* P. Beauv. | | 274 | | Trypanocidal assay | | 100, 250 and 500 mg/mL | | Crystal violet | *T. cruzi* | 0.61 (0.55-0.82) | | The extract and the compounds showed trypanocidal and leishmanicidal activity, being alternamine A (**324**) of the isolated compounds, the one with the highest activity against the two evaluated parasites. | [8] | |
|  |  |  |  | Leishmanicidal assay | | 20, 100 and 250 mg/mL | | Amphotericin B | *L. amazonensis* | >10 |  |  |  |  |
|  |  | 275 | | Trypanocidal assay | | 100, 250 and 500 mg/mL | | Crystal violet | *T. cruzi* | >10 |  |  |  |  |
|  |  |  |  | Leishmanicidal assay | | 20, 100 and 250 mg/mL | | Amphotericin B | *L. amazonensis* | >10 |  |  |  |  |
|  |  | 324 | | Trypanocidal assay | | 100, 250 and 500 mg/mL | | Crystal violet | *T. cruzi* | 0.23 (0.20-0.26) | |  |  |  |
|  |  |  |  | Leishmanicidal assay | | 20, 100 and 250 mg/mL | | Amphotericin B | *L. amazonensis* | 0.16 (0.13-0.20) | |  |  |  |
|  |  | 325 | | Trypanocidal assay | | 100, 250 and 500 mg/mL | | Crystal violet | *T. cruzi* | 0.82 (0.76-0.87) | |  |  |  |
|  |  |  |  | Leishmanicidal assay | | 20, 100 and 250 mg/mL | | Amphotericin B | *L. amazonensis* | >10 | |  |  |  |
|  |  |  | |  | |  | |  |  | (%) of viable trypomastigotes | |  |  |  |
|  |  | EE of aerial parts | | Trypanocidal assay | | 4 mg/mL | | Crystal violet | *T. cruzi* | 24.70 ± 3.38 | |  |  |  |
|  |  |  |  | Leishmanicidal assay | | 1 mg/mL | | Amphotericin B | *L. amazonensis* | 1.47 ± 0.02 | |  |  |  |
| *Blutaparon portulacoides* | | AqE (MCW) of aerial parts | | Trypanocidal assay | | 4000 µg/ml | | Gentian violet | *T. cruzi* | 63.2 | | The AqE of aerial part did not show activity, while both AqE of root showed a ↓ signicant in viability (greater than 90%) of amastigotes, and the AqE MCW appreciably ↓ the viability of the trimastigotes.  The EE of aerial parts is not toxic to *T. cruzi*, but it does ↓ the viability of amastigotes, while EE of root ↓ the viability of the two parasites.  80 does not significantly ↓ parasite viability.  The mixture of acyl steryl glycosides (**395, 412**) in all [] ↓ the viability of both parasites, and this activity is more pronounced in amastigotes of *L. amazonensis.* | [151] | |
|  | |  |  | Leishmanicidal assay | | 1000 µg/ml | | - | *L. amazonensis* | 68.9 | |  |  | |
|  | | AqE (MHW) of aerial parts | | Trypanocidal assay | | 4000 µg/ml | | Gentian violet | *T. cruzi* | 78.4 | |  |  | |
|  | |  |  | Leishmanicidal assay | | 1000 µg/ml | | - | *L. amazonensis* | 96.1 | |  |  | |
|  | | AqE (MCW) of roots | | Trypanocidal assay | | 4000 µg/ml | | Gentian violet | *T. cruzi* | 34.4 | |  |  | |
|  | |  |  | Leishmanicidal assay | | 1000 µg/ml | | - | *L. amazonensis* | 5.3 | |  |  | |
|  | | AqE (MHW) of roots | | Trypanocidal assay | | 4000 µg/ml | | Gentian violet | *T. cruzi* | 78.4 | |  |  | |
|  | |  |  | Leishmanicidal assay | | 1000 µg/ml | | - | *L. amazonensis* | 2.8 | |  |  | |
|  | | EE of aerial parts | | Trypanocidal assay | | 4000 µg/ml | | Gentian violet | *T. cruzi* | 99.2 | |  |  | |
|  | |  |  | Leishmanicidal assay | | 1000 µg/ml | | - | *L. amazonensis* | 44.3 | |  |  | |
|  | | EE of roots | | Trypanocidal assay | | 4000 µg/ml | | Gentian violet | *T. cruzi* | 47.2 | |  |  | |
|  | |  |  | Leishmanicidal assay | | 1000 µg/ml | | - | *L. amazonensis* | 44.3 | |  |  | |
|  | | 80 | | Trypanocidal assay | | 100, 250 and 500 µg/ml | | Gentian violet | *T. cruzi* | 70.0 | |  |  | |
|  | |  |  | Leishmanicidal assay | | 4, 84, and  500 µg/ml | | - | *L. amazonensis* | 83.8 | |  |  | |
|  | | 395 and 412 | | Trypanocidal assay | | 100, 250 and 500 µg/ml | | Gentian violet | *T. cruzi* | 24.9 at the highest [ ] | |  |  | |
|  | |  |  | Leishmanicidal assay | | 4, 84, and  500 µg/ml | | - | *L. amazonensis* | 2.9 at the highest [ ] | |  |  | |
| *Gomphrena agrestis* | | EE of whole plant | | Leishmanicidal assay | | 1 mg/mL | | Amphotericin B | *L. amazonensis* MPRO/BR/72/M 1841 | % V of *L. amazonensis*: 1.7 | | The isolated compounds did not appreciably interfere with parasite viability.  The lack of activity of the isolated compounds could be due to a loss of activity during the isolation procedure and / or synergism. | [2] | |
|  |  | 327 | | Leishmanicidal assay | | 0.02, 0.1 and 0.5 mg/mL | | Amphotericin B | *L. amazonensis* MPRO/BR/72/M 1841 | NDNS |  |  |  |  |
|  |  | 33 | | Leishmanicidal assay | | 0.02, 0.1 and 0.5 mg/mL | | Amphotericin B | *L. amazonensis* MPRO/BR/72/M 1841 | NDNS |  |  |  |  |
|  |  | 106 | | Leishmanicidal assay | | 0.02, 0.1 and 0.5 mg/mL | | Amphotericin B | *L. amazonensis* MPRO/BR/72/M 1841 | NDNS | |  |  |  |
| *Pfaffia glomerata* | | HaE of roots and rhizomes | | Trypanocidal Assays | | 1, 10 and 50 µg/mL | | Gentian violet | *T. cruzi* strain Y | IC_50_: 8.2 x 10^14^ | | Active against *L. braziliensis* but not against *T. cruzi.* | [117] | |
|  |  |  |  | Leishmanicidal assay | | 1, 10 and 50 µg/mL | | Anfotericine B | *L. braziliensis* | IC_50_: 168.6 µg/mL | |  |  |  |
| *Pfaffia glomerata* | | AqE of aerial part | | Leishmanicidal assay | | 1, 10 and 100 µg/mL | | Pentamidine | *L. amazonensis* | % I_100µg/mL_: 83.8 % | | Active against *L. amazonensis* | [234] | |
| *Pfaffia glomerata* | | HaE of roots | | Trypanocidal Assays | | 6.25 to 200 µg/mL | | Benznidazole | *T. cruzi* | IC_50_: 181.69 ± 47.93 | | Of the all fractions, FH showed the highest activity against *T. cruzi*. | [177] | |
|  | | FH of HaE | | Trypanocidal Assays | | 6.25 to 200 µg/mL | | Benznidazole | *T. cruzi* | IC_50_: 47.89 ± 5.87 | |  |  | |
|  | | CF of HaE | | Trypanocidal Assays | | 6.25 to 200 µg/mL | | Benznidazole | *T. cruzi* | IC_50_: 254.73 ± 68.36 | |  |  | |
|  | | Fraction 3 | | Trypanocidal Assays | | 6.25 to 200 µg/mL | | Benznidazole | *T. cruzi* | IC_50_: 136.67 ± 17.74 | |  |  | |
|  | | 226 | | Trypanocidal Assays | | 6.25 to 200 µg/mL | | Benznidazole | *T. cruzi* | IC_50_: 44.78 ± 7.83 | |  |  | |
| **Antiviral activity** | | | | | | | | | | | | | | |
| *Alternanthera philoxeroides* | | 238 | | Antiviral and cytotoxic assays | | 580 to 1100 µM | | ACV | Coxsackievirus type B-1-infected Vero cells | CC_50_: 870 µM IC_50_: > 250 µM and SIs: < 3 | | Has low cytotoxicity in the host cells.  Presented antiviral activity front enveloped viruses (HSV-1, HSV-2, HCMV, measles virus, and mumps virus, except for influenza virus and coronavirus), according to the selectivity indices.  Not interferes with the coupling of the virus to the surface of the host cells, and does not show effects marked in the process of penetration of virus.  Time- and concentration-dependent ↓ in infectivity.  ↓ cell-associated virus levels and levels of virus released in a []-dependent manner.  The reduction in the levels of released viruses was greater than that of cell-associated viruses.  There were no clear differences in the SDSPAGE patterns of these samples. | [41] | |
|  |  |  | |  | |  | |  | HCMV-infected MRC-5 cells | CC_50_: 1100 µM IC_50_: 15 and 24 µM and SIs: 73 and 46 | |  |  |  |
|  |  |  | |  | |  | |  | Human coronavirus- infected MRC-5 cells | CC_50_: 1100 µM IC_50_: > 250 µM and SIs: < 4 | |  |  |  |
|  |  |  | |  | |  | |  | HIV-infected HeLa cells | CC_50_: 840–880 µM IC_50_: > 250 µM and SIs: < 3 | |  |  |  |
|  |  |  | |  | |  | |  | HSV-1-Infected Vero cells | CC_50_: 870 µM IC_50_: 30 and 40 µM and SIs: 29 and 22 | |  |  |  |
|  |  |  | |  | |  | |  | HSV-2-infected Vero cells | CC_50_: 870 µM IC_50_: 29 and 32 µM and SIs: 30 and 27 | |  |  |  |
|  |  |  | |  | |  | |  | Human rhinovirus type 14-infected HeLa cells | CC_50_: 890 µM IC_50_: > 250 µM and SIs: < 4 | |  |  |  |
|  |  |  | |  | |  | |  | Influenza A virus infected MDCK cells | CC_50_: 580 µM IC_50_: > 250 µM and SIs: < 2 | |  |  |  |
|  |  |  | |  | |  | |  | Measles virus-infected Vero cells | CC_50_: 870 µM IC_50_: 35 and 32 µM and SIs: 25 and 27 | |  |  |  |
|  |  |  | |  | |  | |  | Mumps virus-infected Vero cells | CC_50_: 870 µM IC_50_: 35 and 43 µM and SIs: 25 and 20 | |  |  |  |
|  |  |  | |  | |  | |  | Poliovirus type 3-infected Vero cells | CC_50_: 870 µM IC_50_: > 250 µM and SIs: < 3 | |  |  |  |
|  |  |  | | Virus adsorption assay | | 25 and 125 µM | | - | HSV-2-infected Vero cells | NDNS | |  |  |  |
|  |  |  | | Virus penetration assay | | 25 and 125 µM | | Uninformed | HSV-2-infected Vero cells | NDNS | |  |  |  |
|  |  |  | | Virucidal assay | | 20, 100 and 200 µM | | - | HSV-2 | NDNS | |  |  |  |
|  |  |  | | Inhibition of virus release | | 25, 50, 100, and 200 µM | | - | HSV-2-infected Vero cells | % R_200 µM_: 93 and 75 in released viruses and cell-associated viruses, respectively. | |  |  |  |
|  |  |  | | SDS-PAGE analysis of protein | | 0, 10, and 100 µM | | - | HSV-2-infected Vero cells | NDNS | |  |  |  |
| *Alternanthera philoxeroides* | | 59 | | Inhibition Assay of HBsAg | | Uninformed | | - | HBV-infected HepG2.2.15 cells | IC_50_: 28.65 µM | | Compounds 59, 40, y 60 blocks the secretion of HBsAg in a dose-dependent manner, reaching to inhibit the secretion of HBsAg to 70%, 74,1 % and 67,3 % respectively, in non-toxic concentrations 129 µM (Luteolin glycosides) and 127 µM (Chrysoeriol glycosides). | [40] | |
|  | |  |  | Inhibition Assay of HBeAg | | Uninformed | | - | HBV-infected HepG2.2.15 cells | NE | |  |  |  |
|  | |  |  | MTT-Based | | Uninformed | | - | HBV-infected HepG2.2.15 cells | CC_50_: >519 µM | |  |  |  |
|  | | 40 | | Inhibition Assay of HBsAg | | Uninformed | | - | HBV-infected HepG2.2.15 cells | IC_50_: 22.20 µM | |  |  |  |
|  | |  |  | Inhibition Assay of HBeAg | | Uninformed | | - | HBV-infected HepG2.2.15 cells | NE | |  |  |  |
|  | |  |  | MTT-Based | | Uninformed | | - | HBV-infected HepG2.2.15 cells | CC_50_: >253 µM | |  |  |  |
|  | | 60 | | Inhibition Assay of HBsAg | | Uninformed | | - | HBV-infected HepG2.2.15 cells | IC_50_: 31.54 µM | |  |  |  |
|  | |  |  | Inhibition Assay of HBeAg | | Uninformed | | - | HBV-infected HepG2.2.15 cells | NE | |  |  |  |
|  | |  |  | MTT-Based | | Uninformed | | - | HBV-infected HepG2.2.15 cells | CC_50_: >519 µM | |  |  |  |
|  | | 58 | | Inhibition Assay of HBsAg | | Uninformed | | - | HBV-infected HepG2.2.15 cells | IC_50_: 11.39 µM | |  |  |  |
|  | |  |  | Inhibition Assay of HBeAg | | Uninformed | | - | HBV-infected HepG2.2.15 cells | IC_50_: 39.78 µM | |  |  |  |
|  | |  |  | MTT-Based | | Uninformed | | - | HBV-infected HepG2.2.15 cells | CC_50_: 60.10 µM | |  |  |  |
|  | | 39 | | Inhibition Assay of HBsAg | | Uninformed | | - | HBV-infected HepG2.2.15 cells | NE | |  |  |  |
|  | |  |  | Inhibition Assay of HBeAg | | Uninformed | | - | HBV-infected HepG2.2.15 cells | NE | |  |  |  |
|  | |  |  | MTT-Based | | Uninformed | | - | HBV-infected HepG2.2.15 cells | CC_50_: <21.81µM | |  |  |  |

**Supplementary Table 2.** Antioxidant activity of the Gomphrenoideae subfamily

| Species | Extract(s)/ compounds | Assay method | Dose | Positive control | Activity | | | References |
| --- | --- | --- | --- | --- | --- | --- | --- | --- |
|  |  |  |  |  | Values | | Analysis |  |
| *Alternanthera bettzickiana* | EE of aerial parts | FRAP assay | Uninformed | - | 455.24 ± 26.55 | | Dose-dependent free radical scavenging and reducing potential. | [19] |
|  |  | DPPH assay | Uninformed | Quercetin and ascorbic acid | IC_50_: 135.07 ± 21.36 | |  |  |
|  |  | ABTS assay | Uninformed | Ascorbic acid | IC_50_: 103.87 ± 5.77 | |  |  |
| *Alternanthera brasiliana* | EE | DPPH assay | Uninformed | Ascorbic acid | %S = 40 at 250 µg/mL | | Only FEA exhibited radical scavenging activity, which was dose dependent. No correlation between phenolic content and antioxidant activity. | [235] |
|  | BuF | DPPH assay | Uninformed | Ascorbic acid | %S = 40 at 250 µg/mL | |  |  |
|  | DF | DPPH assay | Uninformed | Ascorbic acid | %S = 40 at 250 µg/mL | |  |  |
|  | FEA | DPPH assay | Uninformed | Ascorbic acid | SC_50_: 163 µg/mL | |  |  |
| *Alternanthera brasiliana* | ME of Aerial part | DPPH assay | 0.5 to 512 μg/mL | Trolox | 49.1 ± 5.0 µg TE/mg | |  | [31] |
|  |  | ORAC assay | 6.4 µg/mL | Trolox | 281.8 ± 71.1 µg TE/mg | |  |  |
| *Alternanthera brasiliana* | AqE of leaves | ABTS assay | 0.2274 mg | Trolox | %I_120min_: 94.57 ± 0.29  TEAC_120min_: 2084.66 ± 7.07 | |  | [27] |
|  |  | Phosphomolybdenum assay | 1 mg | Ascorbic acid | %TAC_30min_: 38.60 ± 0.22 | |  |  |
| *Alternanthera brasiliana* | HaE of leaves | Modified H_2_O_2_  assay | 1.0–100.0 μg/mL | Gallic acid | ↑ % V_1μg/mL_: 36.01 ± 2.95  ↑ % V_10μg/mL_: 38.73 ± 2.15  ↑ % V_50μg/mL_: 57.30 ± 2.43  ↑ % V_100μg/mL_: 60.33 ± 1.54 | | HaE of leaves has antioxidant activity, evidenced by the ↑ in the scavenging capacity of DPPH, FRAP and ABTS free radicals. | [193] |
|  |  | DPPH assay | Uninformed | Quercetin and tocopherol | IC_50_: 41.2 ± 4.4 μg/mL | |  |  |
|  |  | FRAP assay | Uninformed | Quercetin and tocopherol | IC_50_: 19.9 ± 0.8 μg/mL | |  |  |
|  |  | ABTS assay | Uninformed | Quercetin and tocopherol | IC_50_: 45.9 ± 7.6 μg/mL | |  |  |
|  |  | NO assay | Uninformed | Quercetin and tocopherol | IC_50_: > 500 μg/mL | |  |  |
| *Alternanthera brasiliana* L | ClE of leaves | DPPH assay |  | Ascorbic acid | 16.25 ± 2.73 % | | HaE of leaves exhibited the highest free radical scavenging activity | [236] |
|  |  | ABTS assay |  | Ascorbic acid | 12.56 ± 2.55 % | |  |  |
|  | ME of leaves | DPPH assay |  | Ascorbic acid | 21.88 ± 2.87 % | |  |  |
|  |  | ABTS assay |  | Ascorbic acid | 23.12 ± 2.10 % | |  |  |
|  | HaE of leaves | DPPH assay |  | Ascorbic acid | IC_50_ = 1.050 ± 0.051 mg/ml | |  |  |
|  |  | ABTS assay |  | Ascorbic acid | IC_50_ = 0.595 ± 0.014 mg/ml | |  |  |
| *Alternanthera flavescens* | ME of Aerial part | DPPH Assay | 0.5 to 512 μg/mL | Trolox | 153.9 ± 21.3 | |  | [31] |
|  |  | ORAC assay | 6.4 µg/mL | Trolox | 628.2 ± 156.7 | |  |  |
| *Alternanthera littoralis* P. Beauv. | EE of aerial parts | ORAC assay | - | Quercetin and caffeic acid | 2560 ± 14.09 mmol of Trolox equivalents/g | | The EE and the isolated compounds showed antioxidant activity. EE presented the highest activity, possibly due to a synergism between the alkaloids, flavonoids and flavonoid glucosides that it contained. Of the isolated compounds, 275 presented the highest activity. | [8] |
|  | 274 | ORAC assay | - | Quercetin and caffeic acid | 0.85 ± 0.001 RTE | |  |  |
|  | 275 | ORAC assay | - | Quercetin and caffeic acid | 1.10 ± 0.001 RTE | |  |  |
|  | 324 | ORAC assay | - | Quercetin and caffeic acid | 0.42 RTE | |  |  |
|  | 325 | ORAC assay | - | Quercetin and caffeic acid | 0.65 ± 0.001 RTE | |  |  |
| *Alternanthera maritima* | EE of aerial parts | Cellular chemiluminescence assays | 6.25-100 μg/mL | - | IC_50_: 57.09 ± 4.27 | | BuF was six times more active than the EE in the cell-free assay.  EE also showed less activity than BuF in the assay in the presence of cells.  The compounds 24, 25, 50, and 129 showed significant activity in the cell-free assay, but in the chemiluminescence assay with cells, only 25 and 24 showed significant activity. | [134] |
|  | BuF of EE of aerial parts |  | 6.25-100 μg/mL | - | IC_50_: 30.45 ± 5.42 | |  |  |
|  | 25 |  | 3.1-50 μmol/L | - | IC_50_: 8.72 ± 0.78 | |  |  |
|  | 24 |  | 3.1-50 μmol/L | - | IC_50_: 6.32 ± 0.79 | |  |  |
|  | 129 |  | 3.1-50 μmol/L | - | IC_50_: 43.41 ± 1.46 | |  |  |
|  | 50 |  | 3.1-50 μmol/L | - | IC_50_: 37.41 ± 1.96 | |  |  |
|  | 43 |  | 3.1-50 μmol/L | - | IC_50_: >50 | |  |  |
|  | 70 |  | 3.1-50 μmol/L | - | IC_50_: >50 | |  |  |
|  | 34 |  | 3.1-50 μmol/L | - | IC_50_: >50 | |  |  |
|  | EE of aerial parts | Cell-free chemiluminescence assay | 6.25-100 μg/mL | - | IC_50_: 11.90 ± 0.44 | |  |  |
|  | BuF of EE of aerial parts |  | 6.25-100 μg/mL | - | IC_50_: 1.88 ± 0.05 | |  |  |
|  | 25 |  | 3.1-50 μmol/L | - | IC_50_: 7.31 ± 0.11 | |  |  |
|  | 24 |  | 3.1-50 μmol/L | - | IC_50_: 4.17 ± 0.13 | |  |  |
|  | 129 |  | 3.1-50 μmol/L | - | IC_50_: 5.22 ± 0.07 | |  |  |
|  | 50 |  | 3.1-50 μmol/L | - | IC_50_: 4.82 ± 0.12 | |  |  |
|  | 43 |  | 3.1-50 μmol/L | - | IC_50_: >50 | |  |  |
|  | 72 |  | 3.1-50 μmol/L | - | IC_50_: >50 | |  |  |
|  | 34 |  | 3.1-50 μmol/L | - | IC_50_: >50 | |  |  |
| *Alternanthera paronychioides* | ME | TEAC assay | Uninformed | - | 448.6 ± 0.1 µM TE/g | | EE presented the highest antioxidant activity. | [35] |
|  |  | ORAC assay | Uninformed | Trolox | 1217.4 ± 97.5 µM QE/g | |  |  |
|  | EE | TEAC assay | Uninformed | - | 470.6 ± 1.1 µM TE/g | |  |  |
|  |  | ORAC assay | Uninformed | Trolox | 5585.3 ± 108.7 µM QE/g | |  |  |
|  | AqE | TEAC assay | Uninformed | - | 295.8 ± 1.1 µM TE/g | |  |  |
|  |  | ORAC assay | Uninformed | Trolox | 657.3 ± 41.9 µM QE/g | |  |  |
| *Alternanthera philoxeroides* | Fraction X of ME of leaves | DPPH Assay | 20, 40 and 60 µg/mL | Trolox | % I_60µg/mL_: 67.9 and IC_50_: 33.94 ± 3.45 | | Dose-dependent inhibition of DPPH.  ME removes the free electrons from the meedium. | [141] |
|  |  | ABTS Assay | 20, 40 and 60 µg/mL | Trolox | % I_60µg/mL_: 51.2 and IC_50_: 60.76 ± 4.31 | |  |  |
| *Alternanthera philoxeroides* | HdE of tender stem, shoots and leaves | DPPH Assay | 200, 400 and 800 µL | Trolox | 0.14 ± 0.00 µmol TE/g FW | |  | [237] |
| *Alternanthera philoxeroides* | EE of whole plant | DPPH Assay | Uninformed | Ascorbic acid | IC_50_: 222.58 ± 0.080 µg/mL | |  | [135] |
|  |  | ABTS Assay | Uninformed | Trolox | IC_50_: 384.00 ± 0.36 µg/mL | |  |  |
| *Alternanthera philoxeroides* | ME of whole plant | DPPH Assay | Uninformed | Ascorbic acid | IC_50_: 116.63 μg/mL | | Significant antioxidant activity of ME.  ME has concentration-dependent iron and copper reducing activity. | [132] |
|  |  | NO radical scavenging assay | Uninformed | Ascorbic acid | IC_50_: 176.74 μg/mL | |  |  |
|  |  | CUPRAC | Uninformed | Ascorbic acid | - | |  |  |
|  |  | FRAP Assay | Uninformed | Ascorbic acid | - | |  |  |
| *Alternanthera philoxeroides* | EE of whole plant | ORAC assay | Uninformed | - | 563 μM TE/g | | The antioxidant activity shown by EE can be attributed to the composition of the extract with phenolic and flavonoid compounds. | [136] |
|  |  | FRAP Assay | Uninformed | - | 151.36 μM (TEAC)/g extract | |  |  |
| *Alternanthera pungens* | AqE of whole plant | DPPH Assay | 50, 100, 150, 200, 250 µg / mL | Ascorbic acid | 24.59%±1.59 | | The extracts showed low antioxidant activity, the methanolic extract being the one that has more activity. | [16] |
|  |  | Reducing power assay | 50, 100, 150, 200, 250 µg / mL | - | 0.168±0.12 - 0.091±0.132 | |  |  |
|  | ME of whole plant | DPPH Assay | 50, 100, 150, 200, 250 µg / mL | Ascorbic acid | Numerical data not shown | |  |  |
|  |  | Reducing power assay | 50, 100, 150, 200, 250 µg / mL | - | Numerical data not shown | |  |  |
|  | nHE of whole plant | DPPH Assay | 50, 100, 150, 200, 250 µg / mL | Ascorbic acid s | Numerical data not shown | |  |  |
|  |  | Reducing power assay | 50, 100, 150, 200, 250 µg / mL | - | Numerical data not shown | |  |  |
| *Alternanthera pungens* | HeE of leafy stems | DPPH Assay | Uninformed | Trolox | NE | | For the two antioxidant activity tests carried out, the extracts showed a low effect compared to the Trolox control. | [238] |
|  | DCM of leafy stems | DPPH Assay | Uninformed | Trolox | NE | |  |  |
|  | EaE of leafy stems | DPPH Assay | Uninformed | Trolox | IC_50_ (mg/mL): 0.92 ± 0.00 | |  |  |
|  | ME of leafy stems | DPPH Assay | Uninformed | Trolox | IC_50_ (mg/mL): 0.38 ± 0.02 | |  |  |
|  | HeE of leafy stems | ABTS Assay | Uninformed | Trolox | NE | |  |  |
|  | DCM of leafy stems | ABTS Assay | Uninformed | Trolox | NE | |  |  |
|  | EaE of leafy stems | ABTS Assay | Uninformed | Trolox | NE | |  |  |
|  | ME of leafy stems | ABTS Assay | Uninformed | Trolox | IC_50_ (mg/mL): 3.28 ± 0.02 | |  |  |
| *Alternanthera sessilis (Linn.)* | AgNPs of AqE of leaves | DPPH Assay | 100 µg / mL to 500 µg / mL | Gallic acid | IC_50_ (µg/mL): 300.6 | | Dose-dependent activity. | [60] |
| *Alternanthera sessilis* | Green leaf juice | ABTS Assay | NA | - | SC_50_: 14 ± 1 µg/mL | | It showed potent activity to reduce FeCl_3_ and scavenging H_2_O_2_. | [195] |
|  |  | DPPH Assay | NA | - | SC_50_: 87 ± 0.6 µg/mL | |  |  |
|  |  | FeCl_3_ reducing Assay | NA | - | SC_50_: 70 ± 3 mg/mL | |  |  |
|  |  | H_2_O_2_ radical scavenging activity | NA | - | %I: 92 ± 0.2 | |  |  |
|  |  | Prevention of H2O2 induced erythrocytes hemolysis | NA | Ascorbic acid | NE | |  |  |
| *Alternanthera sessilis* | HdE of tender stem, shoots and leaves | DPPH Assay | 200, 400 and 800 µL | Trolox | 4.06 ± 0.37 µmol TE/g FW | | Considerable antioxidant activity | [237] |
| *Alternanthera sessilis* | FH of ME of leaves | DPPH Assay | Uninformed | Quercetin | EC_50_ (µg/mL): 93.37 ± 5.64 | | All fractions showed dose-dependent activity.  Leaf fractions had stronger  DPPH scavenging activity than callus fractions. | [196] |
|  | CF of ME of leaves | DPPH Assay | Uninformed | Quercetin | EC_50_ (µg/mL): 115.51 ± 7.56 | |  |  |
|  | FEA of ME of leaves | DPPH Assay | Uninformed | Quercetin | EC_50_ (µg/mL): 10.81 ± 0.29 | |  |  |
|  | BuF of ME of leaves | DPPH Assay | Uninformed | Quercetin | EC_50_ (µg/mL): 35.71 ± 1.24 | |  |  |
|  | AF of ME of leaves | DPPH Assay | Uninformed | Quercetin | EC_50_ (µg/mL): 35.96 ± 1.28 | |  |  |
|  | FH of ME of callus | DPPH Assay | Uninformed | Quercetin | EC_50_ (µg/mL): 171.97 ± 3.53 | |  |  |
|  | CF of ME of callus | DPPH Assay | Uninformed | Quercetin | EC_50_ (µg/mL): 34.12 ± 0.67 | |  |  |
|  | FEA of ME of callus | DPPH Assay | Uninformed | Quercetin | EC_50_ (µg/mL): 43.87 ± 0.39 | |  |  |
|  | BuF of ME of callus | DPPH Assay | Uninformed | Quercetin | EC_50_ (µg/mL): 57.11 ± 0.13 | |  |  |
|  | AF of ME of callus | DPPH Assay | Uninformed | Quercetin | EC_50_ (µg/mL): 354.64 ± 29.12 | |  |  |
| *Alternanthera sessilis* | EE of stem | DPPH Assay | 0, 200, 400, 600, 800, 100 and 1200 µg/mL | Gallic acid | IC_50_ (µg/mL): 782 ± 29.9 | | Dose-dependent activity.  The activity of EE is 25 times less than gallic acid. | [57] |
| *Alternanthera sesillis* (red) | AqE of leaves | DPPH Assay | 0–1000 μg/mL | Gallic acid, rutin, ascorbic acid an BHT | IC_50_ (µg/mL): > 1000 | | Leaf extracts showed higher activity than stem extracts.  The ethanol extract of leaves showed the highest DPPH radical scavenging activity, but this activity was lower than that of the Gallic acid, rutin and ascorbic acid.  Leaf EE also showed the highest TEAC, followed by EaE, HeE and AqE.  The EE of leaves also showed the highest values of FRAP. | [55] |
|  |  | TEAC | 1 mg/mL |  | 0.28 ± 0.04 mmol TE/g | |  |  |
|  |  | FRAP assay | 1 mg/mL | FeSO_4_ | 0.23 ± 0.04 mmol Fe^2+^/g | |  |  |
|  | EE of leaves | DPPH Assay | 0–1000 μg/mL | Gallic acid, rutin, ascorbic acid an BHT | IC_50_ (µg/mL): 154.93 ± 14.99 | |  |  |
|  |  | TEAC | 1 mg/mL |  | 0.41 ± 0.03 mmol TE/g | |  |  |
|  |  | FRAP assay | 1 mg/mL | FeSO_4_ | 1.16 ± 0.06 mmol Fe^2+^/g | |  |  |
|  | EaE of leaves | DPPH Assay | 0–1000 μg/mL | Gallic acid, rutin, ascorbic acid an BHT | IC_50_ (µg/mL): > 1000 | |  |  |
|  |  | TEAC | 1 mg/mL |  | 0.39 ± 0.06 mmol TE/g | |  |  |
|  |  | FRAP assay | 1 mg/mL | FeSO_4_ | 0.46 ± 0.08 mmol Fe^2+^/g | |  |  |
|  | HeE of leaves | DPPH Assay | 0–1000 μg/mL | Gallic acid, rutin, ascorbic acid an BHT | IC_50_ (µg/mL): > 1000 | |  |  |
|  |  | TEAC | 1 mg/mL |  | 0.38 ± 0.12 mmol TE/g | |  |  |
|  |  | FRAP assay | 1 mg/mL | FeSO_4_ | 0.32 ± 0.12 mmol Fe^2+^/g | |  |  |
|  | AqE of stem | DPPH Assay | 0–1000 μg/mL | Gallic acid, rutin, ascorbic acid an BHT | IC_50_ (µg/mL): > 1000 | |  |  |
|  |  | TEAC | 1 mg/mL |  | 0.09 ± 0.01 mmol TE/g | |  |  |
|  |  | FRAP assay | 1 mg/mL | FeSO_4_ | - | |  |  |
|  | EE of stem | DPPH Assay | 0–1000 μg/mL | Gallic acid, rutin, ascorbic acid an BHT | IC_50_ (µg/mL): > 1000 | |  |  |
|  |  | TEAC | 1 mg/mL |  | 0.28 ± 0.04 mmol TE/g | |  |  |
|  |  | FRAP assay | 1 mg/mL | FeSO_4_ | 0.25 ± 0.06 mmol Fe^2+^/g | |  |  |
|  | EaE of stem | DPPH Assay | 0–1000 μg/mL | Gallic acid, rutin, ascorbic acid an BHT | IC_50_ (µg/mL): > 1000 | |  |  |
|  |  | TEAC | 1 mg/mL |  | 0.09 ± 0.02 mmol TE/g | |  |  |
|  |  | FRAP assay | 1 mg/mL | FeSO_4_ | 0.01 ± 0.01 mmol Fe^2+^/g | |  |  |
|  | HeE of stem | DPPH Assay | 0–1000 μg/mL | Gallic acid, rutin, ascorbic acid an BHT | IC_50_ (µg/mL): > 1000 | |  |  |
|  |  | TEAC | 1 mg/mL |  | 0.16 ± 0.01 mmol TE/g | |  |  |
|  |  | FRAP assay | 1 mg/mL | FeSO_4_ | - | |  |  |
| *Alternanthera sessilis* | ME of aerial parts | DPPH Assay | 1–100 µg/mL | Ascorbic acid | IC_50_: 35.39 µg/mL | | []-dependent DPPH and H_2_O_2_ scavenging activity.  The reducing power of ME was also []-dependent.  ME prevents the peroxidation of linoleic acid. | [49] |
|  |  | H_2_O_2_ radical scavenging activity | 10–100 µg/mL | Ascorbic acid | IC_50_: 22.74 µg/mL | |  |  |
|  |  | FRAP Assay | 10–200 µg/mL | - | NDNS | |  |  |
|  |  | FTC in Linoleic Acid System | 100 µg/mL | - | NDNS | |  |  |
| *Alternanthera sessilis* | AqE of whole plant | DPPH Assay | Uninformed | Ascorbic acid, Gallic acid, BHT and RUT. | IC_50_ (µg/mL): >1000 | | It was found that EE and its fractions showed greater antioxidant activity compared to AqE and its fractions. The antioxidant activity shown by this species is due to its high polyphenolic content. | [140] |
|  |  | FRAP Assay | Uninformed |  | 0.02 ± 0.01 mmol Fe^2+^/g | |  |  |
|  |  | TEAC | Uninformed |  | 0.03 ± 0.02 mmol TE/g | |  |  |
|  | EE of whole plant | DPPH Assay | Uninformed | Ascorbic acid, Gallic acid, BHT and RUT. | IC_50_ (µg/mL): 775.00 ± 20.26 | |  |  |
|  |  | FRAP Assay | Uninformed |  | 0.52 ± 0.03 mmol Fe^2+^/g | |  |  |
|  |  | TEAC | Uninformed |  | 0.18 ± 0.02 mmol TE/g | |  |  |
|  | FHa of AqE of whole plant (100-90%) | DPPH Assay | Uninformed | Ascorbic acid, Gallic acid, BHT and RUT. | IC_50_ (µg/mL): >1000 | |  |  |
|  |  | FRAP Assay | Uninformed |  | 0.04 ± 0.01 mmol Fe^2+^/g | |  |  |
|  |  | TEAC | Uninformed |  | 0.02 ± 0.01 mmol TE/g | |  |  |
|  | FHa of AqE of whole plant (90–30%) | DPPH Assay | Uninformed | Ascorbic acid, Gallic acid, BHT and RUT. | IC_50_ (µg/mL): >1000 | |  |  |
|  |  | FRAP Assay | Uninformed |  | 0.06 ± 0.00 mmol Fe^2+^/g | |  |  |
|  |  | TEAC | Uninformed |  | 0.03 ± 0.01 mmol TE/g | |  |  |
|  | FHa of AqE of whole plant (20–0%) | DPPH Assay | Uninformed | Ascorbic acid, Gallic acid, BHT and RUT. | NE | |  |  |
|  |  | FRAP Assay | Uninformed |  | 0.01 ± 0.00 mmol Fe^2+^/g | |  |  |
|  |  | TEAC | Uninformed |  | NE | |  |  |
|  | FHa of EE of whole plant  (0–50%) | DPPH Assay | Uninformed | Ascorbic acid, Gallic acid, BHT and RUT. | IC_50_ (µg/mL): >1000 | |  |  |
|  |  | FRAP Assay | Uninformed |  | 0.21 ± 0.02 mmol Fe^2+^/g | |  |  |
|  |  | TEAC | Uninformed |  | 0.15 ± 0.04 mmol TE/g | |  |  |
|  | FEtAc of EE of whole plant  (40:50:10%) | DPPH Assay | Uninformed | Ascorbic acid, Gallic acid, BHT and RUT. | IC_50_ (µg/mL): 159.43 ± 2.13 | |  |  |
|  |  | FRAP Assay | Uninformed |  | 1.93 ± 0.14 mmol Fe^2+^/g | |  |  |
|  |  | TEAC | Uninformed |  | 0.90 ± 0.13 mmol TE/g | |  |  |
|  | FEtAc of EE of whole plant  (30:20:50%) | DPPH Assay | Uninformed | Ascorbic acid, Gallic acid, BHT and RUT. | IC_50_ (µg/mL): 664.59 ± 13.24 | |  |  |
|  |  | FRAP Assay | Uninformed |  | 0.76 ± 0.07 mmol Fe^2+^/g | |  |  |
|  |  | TEAC | Uninformed |  | 0.26 ± 0.05 mmol TE/g | |  |  |
|  | FEtAc of EE of whole plant  (30:20:50%) | DPPH Assay | Uninformed | Ascorbic acid, Gallic acid, BHT and RUT. | IC_50_ (µg/mL): >1000 | |  |  |
|  |  | FRAP Assay | Uninformed |  | 0.6 ± 0.01 mmol Fe^2+^/g | |  |  |
|  |  | TEAC | Uninformed |  | 0.13 ± 0.01 mmol TE/g | |  |  |
| *Alternanthera tenella* Colla | (0–50%) | ORAC Assay | Uninformed | - | 848.24 µmol of TE/g | | The fractions showed greater activity than the EE.  The antioxidant activity of the EE and the fractions showed a correlation with the content of total phenols.  The radical scavenging activity by the isolated compounds was lower than that of quercetin. | [143] |
|  | FMW (35:65, v/v) | ORAC Assay | Uninformed | - | 4542.7 µmol of TE/g | |  |  |
|  | FMW (40:60, v/v) | ORAC Assay | Uninformed | - | 8416.6 µmol of TE/g | |  |  |
|  | FMW (45:65, v/v) | ORAC Assay | Uninformed | - | 4690.2 µmol of TE/g | |  |  |
|  | FMW (70:30, v/v) | ORAC Assay | Uninformed | - | 1007.2 µmol of TE/g | |  |  |
|  | 34 | ORAC Assay | Uninformed | Quercetin, Isoquercitrin, Caffeic and Chlorogenic acids | 0.72 relative TE | |  |  |
|  | 70 | ORAC Assay | Uninformed |  | 1.41 relative TE | |  |  |
|  | 43 | ORAC Assay | Uninformed |  | 1.92 relative TE | |  |  |
|  | 88 | ORAC Assay | Uninformed |  | 0.96 relative TE | |  |  |
| *Gomphrene celesoids* | Extract | DPPH Assay | Uniformed | Vitamin C | SC_50_: > 60 µg/mL | |  | [194] |
| *Gomphrena* *celosioides* | EE of leaves | FRAP Assay | Uninformed | - | 9.89 ± 0.06 μg GAE/mg dry wt | | EE scavenging DPPH more efficiently than trolox. | [229] |
|  |  | DPPH Assay | Uninformed | Trolox | NDNS | |  |  |
| *Gomphrena globosa* | AqE of inflorescences | DPPH Assay | 1.5- 3 µg/mL | - | IC_50_: 421.0 ± 11.4 | | All 3 extracts have dose-dependent activity.  The commercial preparation exhibits the highest radical scavenging activity of NO.  None of the extracts showed hypochlorous acid scavenging activity. | [98] |
|  |  | O_2_^-^ radical scavenging activity | 1- 4 µg/mL | - | IC_50_: 55.5 ± 23.9 | |  |  |
|  |  | NO radical scavenging assay | 2- 3.5 µg/mL | - | IC_50_: 1260.0 ± 50.5 | |  |  |
|  |  | Hypochlorous acid scavenging | Uninformed | - | NE | |  |  |
|  | EB of inflorescences | DPPH Assay | 1.5- 3 µg/mL | - | IC_50_: 427.6 ± 11.2 | |  |  |
|  |  | O_2_^-^ radical scavenging activity | 1- 4 µg/mL | - | IC_50_: 62.4 ± 6.3 | |  |  |
|  |  | NO radical scavenging assay | 2- 3.5 µg/mL | - | IC_50_: 1166.0 ± 16.7 | |  |  |
|  |  | Hypochlorous acid scavenging | Uninformed | - | NE | |  |  |
|  | Commercial preparation | DPPH Assay | 1.5- 3 µg/mL | - | IC_50_: 474.1 ± 44.8 | |  |  |
|  |  | O_2_^-^ radical scavenging activity | 1- 4 µg/mL | - | IC_50_: 82.6 ± 4.3 | |  |  |
|  |  | NO radical scavenging assay | 2- 3.5 µg/mL | - | IC_50_: 472.3 ± 31.3 | |  |  |
|  |  | Hypochlorous acid scavenging | Uninformed | - | NE | |  |  |
| *Gomphrena globosa* | Flower infusion | DPPH Assay | Uninformed | Trolox | EC_50_ (µg/mL): 4305 ± 74 | | The infusions have a high antioxidant activity (free radical scavenging activity, reducing power and inhibition of lipid peroxidation).  Better results were obtained in the infusion G. globosa (40%) + C. citratus (60%), since in all the tests except FRAP, a synergy relation was observed. | [150] |
|  |  | FRAP Assay | Uninformed | Trolox | EC_50_ (µg/mL): 916 ± 7 | |  |  |
|  |  | β-carotene/ linoleate assay | Uninformed | Trolox | EC_50_ (µg/mL): 4079 ± 31 | |  |  |
|  |  | TBARS Assay | Uninformed | Trolox | EC_50_ (µg/mL): 2496 ± 5 | |  |  |
|  | Infusion: *G. globosa* (25%) + *C. citratus* (75%) | DPPH Assay | Uninformed | Trolox | EC_50_ (µg/mL): 1927 ± 67 | |  |  |
|  |  | FRAP Assay | Uninformed | Trolox | EC_50_ (µg/mL): 1029 ± 20 | |  |  |
|  |  | β-carotene/ linoleate assay | Uninformed | Trolox | EC_50_ (µg/mL): 797 ± 30 | |  |  |
|  |  | TBARS Assay | Uninformed | Trolox | EC_50_ (µg/mL): 1126 ± 16 | |  |  |
|  | Infusion: *G. globosa* (40%) + *C. citratus* (60%) | DPPH Assay | Uninformed | Trolox | EC_50_ (µg/mL): 1651 ± 23 | |  |  |
|  |  | FRAP Assay | Uninformed | Trolox | EC_50_ (µg/mL): 942 ± 13 | |  |  |
|  |  | β-carotene/ linoleate assay | Uninformed | Trolox | EC_50_ (µg/mL): 505 ± 45 | |  |  |
|  |  | TBARS Assay | Uninformed | Trolox | EC_50_ (µg/mL): 1005 ± 64 | |  |  |
| *Gomphrena globosa* | HaE of flowers | DPPH Assay | 100 µg/mL | - | NDNS | | DPPH radical scavenging activity ↑ with time.  Dose-dependent ABTS antiradical activity.  Low intracellular antioxidant activity. | [153] |
|  |  | ABTS Assay | 10–500 µg/mL | - | % I_500µg/mL_: 100 | |  |  |
|  |  | ROS detection in BJ cells | 50, 250 and 500 µg/mL | H_2_O_2_ | Fluorescence was lower about 20–25% in relation to the control | |  |  |
|  |  | ROS detection in HaCaT cells | 50, 250 and 500 µg/mL | H_2_O_2_ |  |  |  |  |
| *Gomphrena globosa* var. albiflor (white amaranth) | HE of flowers | DPPH Assay | 10 mg/mL to 0.02 mg/ mL | Trolox | EC_50_ (µg/mL): 1.36 ± 0.03 | | The pink variety presented the best activity with the lowest EC_50_.  The TBARS test presented the best results in the three plants. | [73] |
|  |  | β-carotene/linoleate assay | 10 mg/mL to 0.02 mg/ mL | Trolox | EC_50_ (µg/mL): 1.47 ± 0.04 | |  |  |
|  |  | FRAP Assay | 10 mg/mL to 0.02 mg/ mL | Trolox | EC_50_ (µg/mL): 1.38 ± 0.03 | |  |  |
|  |  | TBARS Assay | 10 mg/mL to 0.02 mg/ mL | Trolox | EC_50_ (µg/mL): 0.57 ± 0.01 | |  |  |
| *Gomphrena haageana K.* (red amaranth) | HE of flowers | DPPH Assay | 10 mg/mL to 0.02 mg/ mL | Trolox | EC_50_ (µg/mL): 1.19 ± 0.06 | |  | [73] |
|  |  | β-carotene/linoleate assay | 10 mg/mL to 0.02 mg/ mL | Trolox | EC_50_ (µg/mL): 1.30 ± 0.04 | |  |  |
|  |  | FRAP Assay | 10 mg/mL to 0.02 mg/ mL | Trolox | EC_50_ (µg/mL): 0.88 ± 0.01 | |  |  |
|  |  | TBARS Assay | 10 mg/mL to 0.02 mg/ mL | Trolox | EC_50_ (µg/mL): 0.41 ± 0.01 | |  |  |
| *Gomphrena sp.* (pink globe amaranth) | HE of flowers | DPPH Assay | 10 mg/mL to 0.02 mg/ mL | Trolox | EC_50_ (µg/mL): 1.02 ± 0.01 | |  | [73] |
|  |  | β-carotene/linoleate assay | 10 mg/mL to 0.02 mg/ mL | Trolox | EC_50_ (µg/mL): 0.98 ± 0.06 | |  |  |
|  |  | FRAP Assay | 10 mg/mL to 0.02 mg/ mL | Trolox | EC_50_ (µg/mL): 0.84 ± 0.02 | |  |  |
|  |  | TBARS Assay | 10 mg/mL to 0.02 mg/ mL | Trolox | EC_50_ (µg/mL): 0.25 ± 0.03 | |  |  |
| *Iresine angustifolia* | EE of whole plant | ABTS Assay |  | Trolox | 324.70 ± 5.3 µM_TE_/mL | |  | [103] |
|  | AqE of whole plant | ABTS Assay |  | Trolox | 390.60 ± 2.6 µM_TE_/mL | |  |  |
| *Iresine herbstii* | AgNPs of AqE of leaves | DPPH Assay | 100, 200, 300, 400 and 500 µg/mL | Ascorbic acid | AgNPs showed higher DPPH radical scavenging activity and total antioxidant activity than EE.  AgNPs showed a reducing power better than that of ascorbic acid, as well as a higher level of total antioxidant activity. | | | [231] |
|  |  | FRAP Assay |  | Ascorbic acid |  |  |  |  |
|  |  | Total antioxidant capacity | 100, 400, 800 and 1200 µg/mL | Ascorbic acid |  |  |  |  |
|  | EE of leaves | DPPH Assay | 100, 200, 300, 400 and 500 µg/mL | Ascorbic acid |  |  |  |  |
|  |  | FRAP Assay |  | Ascorbic acid |  |  |  |  |
|  |  | Total antioxidant capacity | 100, 400, 800 and 1200 µg/mL | Ascorbic acid |  |  |  |  |
| *Pfaffia glomerata* | HaE of inflorescences | DPPH Assay | 25 to 1000 µg/ml | BHT | IC_50_: 120.23 ± 2.51 | HaE of inflorescences and stems had strong antioxidant activity according to DPPH.  HaE of inflorescences and stems had moderate antioxidant activity according to β-carotene/linoleate assay.  HaE of inflorescences had higher antioxidant activity in all assays. | | [179] |
|  |  | β-carotene/linoleate assay | 1000 µg/ml | BHT | %TAC: 50.4 ± 1.45 |  |  |  |
|  |  | Phosphomolybdenum method | 200 μg/ml | Ascorbic acid | 167.8 ± 0.32 mg AAE/g |  |  |  |
|  | HaE of stems | DPPH Assay | 50 to 1000 µg/ml | BHT | IC_50_: 297.54 ± 1.94 |  |  |  |
|  |  | β-carotene/linoleate assay | 1000 µg/ml | BHT | %TAC: 47.9 ± 0.91 |  |  |  |
|  |  | Phosphomolybdenum method | 200 μg/ml | Ascorbic acid | 137.6 ± 0.11 mg AAE/g |  |  |  |
|  | HaE of roots | DPPH Assay | 50o to 1000 µg/ml | BHT | IC_50_: 1007.29 ± 3.08 |  |  |  |
|  |  | β-carotene/linoleate assay | 1000 µg/ml | BHT | %TAC: 43.7 ± 0.84 |  |  |  |
|  |  | Phosphomolybdenum method | 200 μg/ml | Ascorbic acid | 123.2 ± 0.40 mg AAE/g |  |  |  |
| *Pfaffia glomerata* | ME of roots | DPPH Assay | 10 mg/mL | Ascorbic acid  Ascorbic acid | NE | The different extracts and fractions showed interesting antioxidant activity in the ORAC and DPPH assays, with the FD of aerial part and FD of root showing the highest activity. | | [162] |
|  |  | ORAC Assay | 100 μg/ mL and 10 mg/mL |  | > 1000 µM Trolox eq/g |  |  |  |
|  | ME of aerial part | DPPH Assay | 10 mg/mL | Ascorbic acid  Ascorbic acid | NE |  |  |  |
|  |  | ORAC Assay | 100 μg/ mL and 10 mg/mL |  | NE |  |  |  |
|  | FnH of roots | DPPH Assay | 10 mg/mL | Ascorbic acid | NE |  |  |  |
|  |  | ORAC Assay | 100 μg/ mL and 10 mg/mL | Ascorbic acid | > 1000 µM Trolox eq/g |  |  |  |
|  | FnH of aerial part | DPPH Assay | 10 mg/mL | Ascorbic acid | NE |  |  |  |
|  |  | ORAC Assay | 100 μg/ mL and 10 mg/mL | Ascorbic acid | NE |  |  |  |
|  | FD of roots | DPPH Assay | 10 mg/mL | Ascorbic acid | IC_50_: 2.8 µg/mL |  |  |  |
|  |  | ORAC Assay | 100 μg/ mL and 10 mg/mL | Ascorbic acid | > 1000 µM Trolox eq/g |  |  |  |
|  | FD of aerial part | DPPH Assay | 10 mg/mL | Ascorbic acid | IC_50_: 2.3 µg/mL |  |  |  |
|  |  | ORAC Assay | 100 μg/ mL and 10 mg/mL | Ascorbic acid | > 1000 µM Trolox eq/g |  |  |  |
|  | FEA of roots | DPPH Assay | 10 mg/mL | Ascorbic acid | NE |  |  |  |
|  |  | ORAC Assay | 100 μg/ mL and 10 mg/mL | Ascorbic acid | NE |  |  |  |
|  | FEA of aerial part | DPPH Assay | 10 mg/mL | Ascorbic acid | NE |  |  |  |
|  |  | ORAC Assay | 100 μg/ mL and 10 mg/mL | Ascorbic acid | > 1000 µM Trolox eq/g |  |  |  |
|  | FnB of roots | DPPH Assay | 10 mg/mL | Ascorbic acid | NE |  |  |  |
|  |  | ORAC Assay | 100 μg/ mL and 10 mg/mL | Ascorbic acid | > 1000 µM Trolox eq/g |  |  |  |
|  | FnB of aerial part | DPPH Assay | 10 mg/mL | Ascorbic acid | NE |  |  |  |
|  |  | ORAC Assay | 100 μg/ mL and 10 mg/mL | Ascorbic acid | NE |  |  |  |
|  | AF of roots | DPPH Assay | 10 mg/mL | Ascorbic acid | NE |  |  |  |
|  |  | ORAC Assay | 100 μg/ mL and 10 mg/mL | Ascorbic acid | NE |  |  |  |
|  | AF of aerial part | DPPH Assay | 10 mg/mL | Ascorbic acid | NE |  |  |  |
|  |  | ORAC Assay | 100 μg/ mL and 10 mg/mL | Ascorbic acid | NE |  |  |  |
| *Pfaffia glomerata* | FD of roots | ROS production in BMDM (Bone marrow-derived macrophage) | 250, 25, 2.5, and 0.25 μg/mL  250, 25, 2.5, and 0.25 μg/mL | - | NDNS | Both root and shoot FD ↓ ROS production in cells at all concentrations. | | [162] |
|  | FD of aerial part |  |  | - | NDNS |  |  |  |
| *Pfaffia paniculata* | ME of roots | Lipid peroxidation assay (rat brain membranes) | 1–1000 μM | Quercetin | IC_50_: 4.05 mg/mL | Weak antioxidant activity. | | [215] |
|  |  | DPPH Assay | 0 to 900 μg/mL | Gallic acid | EC_50_: 5.03 mg/mL |  |  |  |
| *Pfaffia townsendii* | HeE of whole plant | DPPH Assay | 100 to 1.6 µg/mL | Quercetin, trolox and cafeic acid | EC_50_: > 200 µg/mL | HeE and hexane partition showed low antioxidant activity according to the DPPH and ORAC tests.  But EE, the DCM phase and the hydroalcoholic phase showed high antioxidant activity. | | [127] |
|  |  | ORAC Assay |  |  | 13.0 µmol of TE/g |  |  |  |
|  | EE of whole plant | DPPH Assay | 100 to 1.6 µg/mL | Quercetin, trolox and cafeic acid | EC_50_: 62.6 µg/mL |  |  |  |
|  |  | ORAC Assay |  |  | 1555 µmol of TE/g |  |  |  |
|  | Hexane phase | DPPH Assay | 100 to 1.6 µg/mL | Quercetin, trolox and cafeic acid | EC_50_: > 200 µg/mL |  |  |  |
|  |  | ORAC Assay |  |  | 2461 µmol of TE/g |  |  |  |
|  | DCM phase | DPPH Assay | 100 to 1.6 µg/mL | Quercetin, trolox and cafeic acid | EC_50_: 45.6 µg/mL |  |  |  |
|  |  | ORAC Assay |  |  | 5641µmol of TE/g |  |  |  |
|  | Hydroalcoholic phase | DPPH Assay | 100 to 1.6 µg/mL | Quercetin, trolox and cafeic acid | EC_50_: 31.9 µg/mL |  |  |  |
|  |  | ORAC Assay |  |  | 4581µmol of TE/g |  |  |  |
|  | 67 | DPPH Assay | 100 to 1.6 µg/mL | Quercetin, trolox and cafeic acid | EC_50_: 4.9 µg/mL |  |  |  |
|  |  | ORAC Assay |  |  | 4.2 µmol of TE/g |  |  |  |
|  | 106 | DPPH Assay | 100 to 1.6 µg/mL | Quercetin, trolox and cafeic acid | EC_50_: 83.2 µg/mL |  |  |  |
|  |  | ORAC Assay |  |  | 0.8 µmol of TE/g |  |  |  |
|  | 67 and 106 | DPPH Assay | 100 to 1.6 µg/mL | Quercetin, trolox and cafeic acid | EC_50_: 3.7 µg/mL |  |  |  |
|  |  | ORAC Assay |  |  | 4.8 µmol of TE/g |  |  |  |

**Supplementary Table 3.** Anticancer activity of the Gomphrenoideae subfamily.

| Species | Extract(s)/ compounds | Assay method | Cell line | Dose | Positive control | Activity | | | References |
| --- | --- | --- | --- | --- | --- | --- | --- | --- | --- |
| *Alternanthera bettzickiana* | AuNPs of AqE of leaves | MTT assay | Cancer cell lines A549 | 1000 to 1.953 µg/mL | - | Cancer A549 cells showed a morphological change when treated with [] of 50-100 mM AuNPs.  AuNPs induce apoptotic death.  ↑ the activation of caspases, cytochrome C.  ↑ the level of Bax, Bcl-2 and p53.  ↓ CDC2, CDK2, CDK4, CDK6, COX-1, COX-2, dc25C, PGE2 and proteins cyclin A and B. | | | [21] |
|  |  |  |  |  |  |  |  |  |  |
|  |  | Light microscopic study | A549 | IC_50_ | - |  |  |  |  |
|  |  | Fluorescent microscopic study | A549 | Uninformed | - |  |  |  |  |
|  |  | DNA fragmentation study | Cancer cell lines A549 | Uninformed | - |  |  |  |  |
|  |  | Nuclear staining | Cancer cell lines A549 | 10, 25 and 50 µg/mL | - |  |  |  |  |
|  |  | Western blotting | Cancer cell lines A549 | 10, 25 and 50 µg/mL | - |  |  |  |  |
|  |  | Analysis of cell cycle distribution | Cancer cell lines A549 | IC_50_ | - |  |  |  |  |
|  |  | RT-PCR | Cancer cell lines A549 | 10, 25 and 50 µg/mL |  |  |  |  |  |
| *Alternanthera brasiliana* | EaE of leaves | Trypan blue dye exclusion method | EAC cells | 4, 8, 16, 32 and 64 µg/m | 5‑FU | IC_50_: 33.54 µg | | [] dependant cytotoxicity. | [185] |
|  |  | MTT assay | EAC cells | 4, 8, 16, 32 and 64 µg/m | 5‑FU | IC_50_: 33.69 µg  %I_64µg/mL_: 96.41 ± 2.56 | |  |  |
| *Alternanthera brasiliana* | ME of Aerial part | MTT assay | Caco-2 | 16 – 512 μg/mL | Vinorelbine ditartrate salt  hydrate | IC_50_: 252.9 ± 5.7 µg/mL | | | [31] |
|  |  |  | HT-29 |  |  | IC_50_: 160.3 ± 8.5 µg/mL | | |  |
|  |  |  | Hep-G2 |  |  | IC_50_: 281.1 ± 11.5 µg/mL | | |  |
| *Alternanthera* *flavescens* | ME of Aerial part | MTT assay | Caco-2 | 16 – 512 μg/mL | Vinorelbine ditartrate salt  hydrate | IC_50_: 300.1 ± 9.1 µg/mL | | | [31] |
|  |  |  | HT-29 |  |  | IC_50_: 203.4 ± 7.3 µg/mL | | |  |
|  |  |  | Hep-G2 |  |  | IC_50_: 79.4 ± 6.6 µg/mL | | |  |
| *Alternanthera*  *philoxeroides* | 36 | Uninformed | HeLa | 10 and 30 µg/mL | - | % I: 8.9 and 26.5 | | 456 and 455 showed significant effects on the growth of Hela cells. | [36] |
|  | 456 | Uninformed | HeLa | 10 and 30 µg/mL | - | % I: 40.7 and 72.1 | |  |  |
|  | 35 | Uninformed | HeLa | 10 and 30 µg/mL | - | % I: 49.0 and 55.9 | |  |  |
|  | 457 | Uninformed | HeLa | 10 and 30 µg/mL | - | % I: 28.9 and 58.2 | |  |  |
|  | 455 | Uninformed | HeLa | 10 and 30 µg/mL | - | % I: 52.9 and 72.2 | |  |  |
| *Alternanthera philoxeroides* | 261 | MTT assay | HL60 | Uninformed | - | IC_50_: 185.29 µg/ml | | All compounds showed cytotoxic activity | [37] |
|  |  |  | SK-N-SH | Uninformed | - | IC_50_: 51.00 µg/ml | |  |  |
|  | 262 | MTT assay | HL60 | Uninformed | - | IC_50_: 185.57 µg/ml | |  |  |
|  |  |  | SK-N-SH | Uninformed | - | IC_50_: 118.69 µg/ml | |  |  |
|  | 263 | MTT assay | HL60 | Uninformed | - | IC_50_: 271.45 µg/ml | |  |  |
|  |  |  | SK-N-SH | Uninformed | - | IC_50_: 60.60 µg/ml | |  |  |
|  | 264 | MTT assay | HL60 | Uninformed | - | IC_50_: 45.93 µg/ml | |  |  |
|  |  |  | SK-N-SH | Uninformed | - | IC_50_: 37.29 µg/ml | |  |  |
| *Alternanthera sessilis* | EE of whole plant | MTT assay | HeLa | 250 and 500 µg/mL | Cisplatin | %I cell _500µg/mL_: 40  %I cell _250µg/mL_: 20 | | Moderate cytotoxicity | [242] |
| *Alternanthera sessilis* | ME of leaves | PC biosensor-based cell attachment assay | Panc-1 | 100 μg/mL | DOX and Cur | Fraction of cell survival: 1.63 ± 0.41 | | CF induced more than 80% cell death in the biosensor-based assay.  CF induces cell death in more than 70%, in the three cell lines evaluated, but  limited selectivity. | [244] |
|  | PEF of leaves | PC biosensor-based cell attachment assay | Panc-1 | 100 μg/mL | DOX and Cur | Fraction of cell survival: 0.43 ± 0.14 | |  |  |
|  | CF of leaves | PC biosensor-based cell attachment assay | Panc-1 | 100 μg/mL | DOX and Cur | Fraction of cell survival: 0.04 ± 0.09 | |  |  |
|  |  | MTT assay | Panc-1 | 100 μg/mL | DOX and Cur | IC_50_: 27.19 ± 3.01 μg/mL | |  |  |
|  |  | MTT assay | MIA PaCa-2 | 100 μg/mL | DOX and Cur | IC_50_: 13.08 ± 10.40 μg/mL | |  |  |
|  |  | MTT assay | Capan-1 | 100 μg/mL | DOX and Cur | IC_50_: 34.92 ± 2.20 μg/mL | |  |  |
| *Alternanthera sessilis* | AgNPs of AqE of leaves | MTT assay | PC3 | 1.56, 3.12, 6.25, 12.5, and 25 μl/mL | - | IC_50_: 6.85 μg/mL  %I cell _25µg/mL_: 94.11 | | Dose-dependent cytotoxic activity. | [245] |
| *Alternanthera sessilis* | ME of whole plant | Lethality bioassay | Brine shrimp | 5, 10, and 20 µg/mL | Vincristine sulfate | LC_50_: 19.825 and LC_90_: 601.458 | | Can be considered as a candidate for an anticancer compound. | [226] |
| *Alternanthera sessilis* | AgNPs of AqE of aerial parts | MTT assay | MCF-7 cell | 1.56, 3.12, 6.25,  12.5, 25 lL/mL | Cisplatin | Induces apoptosis, dose-dependent cytotoxic activity (IC_50_: 3.043 µL/mL  %I cell _25µg/mL_: 99) and greater activity than Cisplatin. | | | [184] |
| *Alternanthera sessilis* | AuNPs of AqE of leaves | MTT assay | HeLa | 1–15 µg/ml | - | Dose-dependent cytotoxic activity.  The induction of apoptosis in cells is [] dependent.  ↑ the expression of Bax and ↓ the expression of Bid and Bcl-2.  ↑ the activity of caspase 8, 9 and 3. | | | [62] |
|  |  | AO/PI staining | HeLa | IC_50_ | - |  |  |  |  |
|  |  | Caspase 8, 9 and 3 activity assay | HeLa | Uninformed | - |  |  |  |  |
|  |  | Western blot analysis | HeLa | Uinformed | - |  |  |  |  |
| *Alternanthera sessilis* | AuNPs of AqE | MTT assay | MCF-7 cell | 12.5, 25, 50, 100, 200 µg/ml | - | Dose-dependent activity.  AuNPs did not show much anticancer potential (% I_200µg /kg_: 46.1) | | | [246] |
| *Alternanthera sessilis* | AgNPs of AqE of leaves | MTT assays | L 929 cell lines | 50, 100, 150, 200, and 250 µg/mL | - | Inhibition of L929 cells at a minimal concentration of 5.0 µg/mL | | | [227] |
| *Alternanthera sessilis* | ZnONPs of leaves | MTT assays | MCF-7 cell | 15.6, 31.25, 62.5, 125, 250 and 500 µg/mL | - | IC_50_: 210 μg/mL | Dose-dependent activity | | [228] |
|  |  | Apoptosis and morphological changes | MCF-7 cell | 210 μg/mL and  500 μg/mL | - | ZnONPs ZnONPs at the doses evaluated caused morphological changes, such as apoptotic condensed chromatin and distorted membranes of dead cells. | | |  |
|  |  | Detection of ROS | MCF-7 cell | 210 μg/mL and  500 μg/mL | - | ZnONPs was able to induce ROS production when compared to the control treatment. | | |  |
|  |  | DAPI staining | MCF-7 cell | 210 μg/mL and  500 μg/mL | - | ZnONPs caused nuclear morphological changes, such as nuclear condensation in their chromatin. | | |  |
|  |  |  |  |  |  |  | | |  |
| *Alternanthera tenella* | AgNPs of AqE of leaves | MTT assay | MCF-7 cell line | 25 to 100 µg/mL | - | % I: 98, 54 and 99 for AgNPs, FF and mixture of AgNPs + FF, respectively.  The cytotoxic activity of AgNPs is dose dependent (IC_50_: 42.5 µg/mL).  Apoptotic cells are observed at doses of 100 µg/mL.  Dose-dependent inhibition of cell migration. | | | [70] |
|  |  | AO/PI staining | MCF-7 cell line | Various [] | - |  |  |  |  |
|  |  | Cell migration assay | MCF-7 cell line | 5 to 20 µg/mL | - |  |  |  |  |
|  | FF of AqE of leaves | MTT assay | MCF-7 cell line | 25 to 100 µg/mL | - |  |  |  |  |
|  | AgNPs + FF | MTT assay | MCF-7 cell line | 25 to 100 µg/mL | - |  |  |  |  |
| *Gomphrena* *celosioides* | EE of leaves | SRB assay | KKU-100 (JCRB 1568) | 62.5–1,000 μg/mL | - | Dose and time dependent activity.  Slight inhibitory effects on cholangiocarcinoma cell growth. | | | [239] |
|  |  | SRB assay | KKU-213 (JCRB 1557) | 62.5–1,000 μg/mL | - |  |  |  |  |
| *Gomphrena elegans mart.* | AqE of leaves | MTT assay | HCT-8 | 100 µg/mL | DOX | % L: 99,68 | Only the AqE and HeE extracts from leaves showed a % L above 96% against the three cell lines, and the ClE from aerial stems showed an activity of 79.08% against HCT-8. The other extracts presented a% L below 48%. | | [156] |
|  |  |  | SF-295 |  |  | % L: 97,39 |  |  |  |
|  |  |  | MDA-MB-435 |  |  | % L: 97,39 |  |  |  |
|  | ClE of leaves | MTT assay | HCT-8 | 100 µg/mL | DOX | % L: 11,90 |  |  |  |
|  |  |  | SF-295 |  |  | % L: 23,60 |  |  |  |
|  |  |  | MDA-MB-435 |  |  | % L: - |  |  |  |
|  | HeE of leaves | MTT assay | HCT-8 | 100 μg/mL | DOX | % L: 101,16 |  |  |  |
|  |  |  | SF-295 |  |  | % L: 96,35 |  |  |  |
|  |  |  | MDA-MB-435 |  |  | % L: 96,35 |  |  |  |
|  | ME of leaves | MTT assay | HCT-8 | 100 μg/mL | DOX | % L: 0,43 |  |  |  |
|  |  |  | SF-295 |  |  | % L: 11,53 |  |  |  |
|  |  |  | MDA-MB-435 |  |  | % L: 33,80 |  |  |  |
|  | AqE of aerial stem | MTT assay | HCT-8 | 100 μg/mL | DOX | % L: 26,90 |  |  |  |
|  |  |  | SF-295 |  |  | % L: 18,38 |  |  |  |
|  |  |  | MDA-MB-435 |  |  | % L: 18,38 |  |  |  |
|  | ClE of aerial stem | MTT assay | HCT-8 | 100 μg/mL | DOX | % L: 79,08 |  |  |  |
|  |  |  | SF-295 |  |  | % L: 9,78 |  |  |  |
|  |  |  | MDA-MB-435 |  |  | % L: 9,78 |  |  |  |
|  | HeE of aerial stem | MTT assay | HCT-8 | 100 μg/mL | DOX | % L: 12,74 |  |  |  |
|  |  |  | SF-295 |  |  | % L: 24,38 |  |  |  |
|  |  |  | MDA-MB-435 |  |  | % L: 24,38 |  |  |  |
|  | ME of aerial stem | MTT assay | HCT-8 | 100 μg/mL | DOX | % L: 15,49 |  |  |  |
|  |  |  | SF-295 |  |  | % L: 16,43 |  |  |  |
|  |  |  | MDA-MB-435 |  |  | % L: 16,43 |  |  |  |
|  | AqE of submerged stems | MTT assay | HCT-8 | 100 μg/mL | DOX | % L: - |  |  |  |
|  |  |  | SF-295 |  |  | % L: 22,00 |  |  |  |
|  |  |  | MDA-MB-435 |  |  | % L: 47,35 |  |  |  |
|  | ClE of submerged stems | MTT assay | HCT-8 | 100 μg/mL | DOX | % L: 16,89 |  |  |  |
|  |  |  | SF-295 |  |  | % L: 19,33 |  |  |  |
|  |  |  | MDA-MB-435 |  |  | % L: 0,33 |  |  |  |
|  | HeE of submerged stems | MTT assay | HCT-8 | 100 μg/mL | DOX | % L: 11,61 |  |  |  |
|  |  |  | SF-295 |  |  | % L: 14,47 |  |  |  |
|  |  |  | MDA-MB-435 |  |  | % L: 5,69 |  |  |  |
|  | ME of submerged stems | MTT assay | HCT-8 | 100 μg/mL | DOX | % L: 11,28 |  |  |  |
|  |  |  | SF-295 |  |  | % L: 31,00 |  |  |  |
|  |  |  | MDA-MB-435 |  |  | % L: 23,74 |  |  |  |
|  | AqE of roots | MTT assay | HCT-8 | 100 μg/mL | DOX | % L: 3,57 |  |  |  |
|  |  |  | SF-295 |  |  | % L: 3,80 |  |  |  |
|  |  |  | MDA-MB-435 |  |  | % L: 3,11 |  |  |  |
|  | ClE of roots | MTT assay | HCT-8 | 100 μg/mL | DOX | % L: 16,11 |  |  |  |
|  |  |  | SF-295 |  |  | % L: 19,20 |  |  |  |
|  |  |  | MDA-MB-435 |  |  | % L: 19,51 |  |  |  |
|  | HeE of roots | MTT assay | HCT-8 | 100 μg/mL | DOX | % L: 16,85 |  |  |  |
|  |  |  | SF-295 |  |  | % L: 22,47 |  |  |  |
|  |  |  | MDA-MB-435 |  |  | % L: 13,96 |  |  |  |
|  | ME of roots | MTT assay | HCT-8 | 100 μg/mL | DOX | % L: 9,01 |  |  |  |
|  |  |  | SF-295 |  |  | % L: 1,13 |  |  |  |
|  |  |  | MDA-MB-435 |  |  | % L: 11,90 |  |  |  |
| *Gomphrena elegans Mart.* | FD of leaves | MTT assay | HCT-8 | 100 μg/mL | DOX | %L: 13.80 | The FnH of leaves presented a % L of 100% against HCT8, and 96.38% against MDA-MB-435 and SF-295. FnB presented a % L above 96% against the three cell lines. The rest of the fractions obtained a % L lower than 25%. | | [241] |
|  |  |  | MDA-MB-435 |  |  | %L: 19.17 |  |  |  |
|  |  |  | SF-295 |  |  | %L: 19.17 |  |  |  |
|  | FEA of leaves | MTT assay | HCT-8 | 100 μg/mL | DOX | %L: 0.91 |  |  |  |
|  |  |  | MDA-MB-435 |  |  | %L: 10.04 |  |  |  |
|  |  |  | SF-295 |  |  | %L: 10.04 |  |  |  |
|  | FHa of leaves | MTT assay | HCT-8 | 100 μg/mL | DOX | %L: 18.24 |  |  |  |
|  |  |  | MDA-MB-435 |  |  | %L: 10.43 |  |  |  |
|  |  |  | SF-295 |  |  | %L: 10.43 |  |  |  |
|  | FnB of leaves | MTT assay | HCT-8 | 100 μg/mL | DOX | %L: 99.68 |  |  |  |
|  |  |  | MDA-MB-435 |  |  | %L: 97.39 |  |  |  |
|  |  |  | SF-295 |  |  | %L: 97.39 |  |  |  |
|  | FnH of leaves | MTT assay | HCT-8 | 100 μg/mL | DOX | %L: 100.00 |  |  |  |
|  |  |  | MDA-MB-435 |  |  | %L: 96.35 |  |  |  |
|  |  |  | SF-295 |  |  | %L: 96.35 |  |  |  |
|  | FD of stems | MTT assay | HCT-8 | 100 μg/mL | DOX | %L: 9.78 |  |  |  |
|  |  |  | MDA-MB-435 |  |  | %L: 9.78 |  |  |  |
|  |  |  | SF-295 |  |  | %L: 9.78 |  |  |  |
|  | FEA of stems | MTT assay | HCT-8 | 100 μg/mL | DOX | %L: 16.43 |  |  |  |
|  |  |  | MDA-MB-435 |  |  | %L: 16.43 |  |  |  |
|  |  |  | SF-295 |  |  | %L: 16.43 |  |  |  |
|  | FHa of stems | MTT assay | HCT-8 | 100 μg/mL | DOX | %L: 19.95 |  |  |  |
|  |  |  | MDA-MB-435 |  |  | %L: 19.95 |  |  |  |
|  |  |  | SF-295 |  |  | %L: 19.95 |  |  |  |
|  | FnB of stems | MTT assay | HCT-8 | 100 μg/mL | DOX | %L: 18.38 |  |  |  |
|  |  |  | MDA-MB-435 |  |  | %L: 18.38 |  |  |  |
|  |  |  | SF-295 |  |  | %L: 18.38 |  |  |  |
|  | FnH of stems | MTT assay | HCT-8 | 100 μg/mL | DOX | %L: 24.38 |  |  |  |
|  |  |  | MDA-MB-435 |  |  | %L: 24.38 |  |  |  |
|  |  |  | SF-295 |  |  | %L: 24.38 |  |  |  |
| *Gomphrena globosa* | ME of whole plant | MTT assay | MT-1 | 0.1, 1, 10 and 100 μg/mL | - | ME not showed activity against any of the cell lines evaluated. | | | [243] |
|  |  |  | MT-2 |  |  |  |  |  |  |
| *Gomphrena globosa* | ME of whole plant | MTT assay | B16F10 | 1-100 μg/mL | - | ME not showed activity against any of the cell lines evaluated. | | | [247] |
|  |  |  | HeLa |  |  |  |  |  |  |
|  |  |  | MK-1 |  |  |  |  |  |  |
| *Gomphrena macrocephala* | 244 | MTT assay | HSC-2 cells | Uninformed | Etoposide | NE | The isolated triterpenes did not show cytotoxic activity at a [] of 100 µM, but their aglycones showed comparable activity with the etoposide. | | [99] |
|  | 243 | MTT assay | HSC-2 cells | Uninformed | Etoposide | NE |  |  |  |
|  | 245 | MTT assay | HSC-2 cells | Uninformed | Etoposide | NE |  |  |  |
|  | 246 | MTT assay | HSC-2 cells | Uninformed | Etoposide | IC_50_: 21 µM |  |  |  |
|  | 247 | MTT assay | HSC-2 cells | Uninformed | Etoposide | IC_50_: 21 µM |  |  |  |
| *Gomphrena martiana* | Mixure of 6, 7, 10 and 17 | Determination of protein level and cytotoxicity | KB cell | 1, 10 and 100 μg/mL | 6-Mercaptopurine | ED_50_: 27.5 μg/mL | Moderate cytotoxic activity | | [100] |
| *Hebanthe paniculata* | Pfaffosidic  fraction of roots | MTT Assay | HepG2 | 100 𝜇g/mL | - | % Viability at 72h: 69 % | Significantly ↓ the viability and cell proliferation  It ↓ cells in S phase and consequently ↑ the G_0_/G_1_ phase.  ↑ sub-G_1_ cell population inducing apoptosis via caspase-3, since it does not affect the DNA.  ↓ cyclin D1 and E, CDK4 and -2, and ↑ p27. | | [121] |
|  |  | Cell Cycle Analysis | HepG2 | 100 𝜇g/mL | - | - |  |  |  |
|  |  | Immunocytochemical detection of BrdU and activated Caspase-3 | HepG2 | 100 𝜇g/mL | - | PIx BrdU: 22 % (24h), 62 % (48h), and 36 % (72)  PIx Caspase 3: 51.03 % (24h), 70.58 % (48h) and 33.96 (72 h) |  |  |  |
|  |  | DNA Fragmentation assay | HepG2 | 100 𝜇g/mL | - | - |  |  |  |
|  |  | Western Blot Analysis | HepG2 | 100 𝜇g/mL | - | - |  |  |  |
|  |  | RT-PCR | HepG2 | 100 𝜇g/mL | - | - |  |  |  |
| *Hebanthe paniculata* | ME of roots | MTT assay | HCT116 | 15.625, 31.25, 62.5, 125, 250, and 500 μg/mL | 5-FU | Uninformed | A significant decline in the viability of the cells evaluated was obtained. The decline was time- and dose-dependent. | | [230] |
|  |  | MTT assay | AT1 | 15.625, 31.25, 62.5, 125, 250, and 500 μg/mL | 5-FU | IC50: 88.5 μg/mL at 72h |  |  |  |
| *Iresine diffusa* | 269 | Alamar blue assay | Human LNCaP | 12.5, 25, and 50 mM | - | The isolated compounds inhibit the growth of human LNCaP cells (AR-sensitive), but not that of PC3 (AR-Non-sensitive) | | | [165] |
|  |  |  | PC3 |  |  |  |  |  |  |
|  | 270 | Alamar blue assay | Human LNCaP | 12.5, 25, and 50 mM | - |  |  |  |  |
|  |  |  | PC3 |  |  |  |  |  |  |
| *Iresine herbstii* | AgNPs of AqE of leaves | Trypan blue dye exclusión method | HeLa cells | 25, 50, 100, 200 and 300 µg/mL | - | %V_300 µg/mL_: 88  LC_50_: 51 µg/mL | Dose-dependent cytotoxicity. | | [231] |
| *Pfafia paniculuta* | 255 | Inhibition test | B-16 | Uninformed | Uninformed | IC: 50 µg/ml | Pfaffosides F (260) was the one who presented the highest inhibitory activity | | [125,164] |
|  | 256 | Inhibition test | B-16 | Uninformed | Uninformed | Uninformed |  |  |  |
|  | 257 | Inhibition test | B-16 | Uninformed | Uninformed | IC: 100 µg/ml |  |  |  |
|  | 258 | Inhibition test | B-16 | Uninformed | Uninformed | IC: 70 µg/ml |  |  |  |
|  | 259 | Inhibition test | B-16 | Uninformed | Uninformed | IC: 120 µg/ml |  |  |  |
|  | 260 | Inhibition test | B-16 | Uninformed | Uninformed | IC: 30 µg/ml |  |  |  |
| *Pfaffia paniculata* | BE of roots | Cell cytotoxicity assay | MCF-7 cells | 100 to 900 µg/mL |  | ↓ cell growth in a dose-dependent manner.  BE exhibits cytotoxic activity.  At 1000 µg/mL, cytoplasmic vacuolization, cluster of round cells, bleb formation, and contraction of nucleus are observed.  ↓ BrdU positive cells.  500 and 1000 µg/mL cause alteration of the organelles of the nucleus and cytoplasm and absence of mitochondria. | | | [124] |
|  |  | AO/BE staining | MCF-7 cells | 500 and 1000 µg/mL | - |  |  |  |  |
|  |  | BrdU immunocytochemistry and cell proliferation | MCF-7 cells | 500 and 1000 µg/mL | - |  |  |  |  |
|  |  | Ultrastructural analysis | MCF-7 cells | 500 and 1000 µg/mL | - |  |  |  |  |
